# Sustainable Access to 5‐Amino‐Oxazoles and Thiazoles via Calcium‐Catalyzed Elimination‐Cyclization with Isocyanides

**DOI:** 10.1002/cssc.202100225

**Published:** 2021-03-03

**Authors:** Ashley J. Basson, Mark G. McLaughlin

**Affiliations:** ^1^ Department of Natural Sciences Manchester Metropolitan University Chester Street Manchester M1 5GD United Kingdom

**Keywords:** calcium catalysis, cyclisation, isocyanides, oxazoles, thiazoles

## Abstract

Herein, we report a sustainable, modular, rapid and high‐yielding transformation to afford densely functionalized 5‐aminooxazoles and thiazoles. The reaction is tolerant to a wide range of functional groups and is typically complete in under 30 min. Furthermore, the described transformation is inherently green in relation to the catalyst and solvent choice as well as producing environmentally benign alcoholic by‐products.

## Graphical abstract

**The future of Ca**: A novel, sustainable, modular and high yielding synthesis of medicinally important scaffolds has been realized. The reaction is tolerant of a range of functional groups and produces environmentally benign alcoholic by‐products. The rapid nature of the reaction, coupled with the modulatory of the methodology, means it should find use in a range of applications.

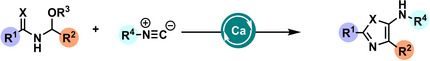

The advent of more sustainable synthetic methodology is of paramount importance to the future of manufacturing, healthcare and agriculture. The requirement to find reaction conditions that use more environmentally benign reagents is now a cornerstone of modern synthetic organic chemistry, with the introduction of the Twelve Principles of Green Chemistry cementing its importance within the community.^[1]^ This has led to seminal work describing the use of non‐precious transition metals and main group organometallics in synthesis,^[2]^ as well as the increase in the use of photo‐^[3]^ and electrochemistry^[4]^ to mediate important transformations. Furthermore, the use of organocatalysis^[5]^ and more sustainable radical initiators^[6]^ have been successfully employed to produce important intermediates and complex scaffolds alike.

Our enthusiasm for sustainable synthesis stems from our interest in the use of group 2 metals in synthesis.^[7]^ In particular, we are interested in accessing scaffolds which hold special interest to medicinal chemists,^[8]^ with our current work focusing on 5‐membered heterocyclic motifs.^[9]^ Oxazoles play an increasingly important role in the discovery of new therapeutics,^[10]^ from antibacterial agents targeting multiple ESKAPE pathogens^[11]^ to novel kinase inhibitors for the treatment of cancer.^[12]^ Unsurprisingly, much attention has been paid to their synthesis, including the classical Robinson‐Gabriel synthesis^[13]^ and Van‐Leusen reaction.^[14]^ More modern approaches using stoichiometric Lewis acids,^[15]^ and redox strategies^[16]^ have been successful in producing a range of substituted oxazoles. Of greater interest are methods employing at least one of the principles of green chemistry, including transition metal^[17]^ and photoredox catalysis^[18]^ as well as innovative strategies utilizing main group reagents.^[19]^

Owing to our interest in employing group 2 metal catalysts to generate reactive intermediates, we sought to establish a method to produce oxazole motifs bearing multiple functional handles, and in particular, oxazoles containing a 5‐amino group. This was borne out of the fact that although many elegant strategies have been described for multiply functionalized oxazoles,^[20]^ there are limited reports whereby the oxazole is formed directly using truly catalytic approaches (Scheme 1).

**Scheme 1:**
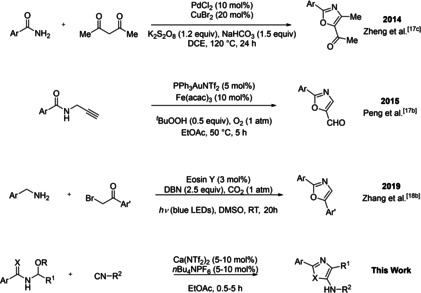
Previous closely related catalytic examples. DCE=1,2‐dichloroethane, DBN=1,5‐Diazabicyclonon‐5‐ene, DMSO=Dimethyl sulfoxide, Tf=Triflate

Our investigation therefore began by exploring the feasibility of this strategy, using **1** **a** as a model substrate. Upon treating **1** **a** with *tert*‐butylisocyanide in the presence of catalytic Ca(NTf_2_)_2_/*n*Bu_4_NPF_6_ ^[21]^ in DCE at 80 °C, we were pleased to discover that the reaction had gone to completion within 5 min, providing the desired oxazole in 83 % isolated yield (Table 1, entry 1). Increasing the reaction time to 15 min led to a slight increase in yield (Table 1, entry 3); however, running the reactions over a prolonged period of time resulted in a noticeable drop in yield and reproducibility (Table 1, entry 2). In an effort to increase the overall sustainability of the reaction, we proceeded to screen a range of solvents touted as being more green.^[22]^ Surprisingly, given the oxophilic nature of calcium,^[23]^ ethyl acetate turned out to be the best solvent in terms of yield, reproducibility and reaction time. Finally, in order to rule out acid‐based catalysis, we performed the reaction in the presence 2,6‐di‐*tert*‐butylpyridine, and found no reduction in reactivity (Table 1, entry 8).

**Table 1:** Reaction optimization.

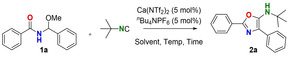					
Entry	Loading [mol %]	*T* [°C]	Solvent	*t*	Yield [%]
1	10	80	1,2‐DCE	5 min	83
2	1	80	1,2‐DCE	2 h	73
3	5	80	1,2‐DCE	15 min	86
4	5	80	EtOAc	30 min	99[a]
5	5	80	EtOAc	30 min	92[b]
6	5	80	MeCN	12 h	53[a]
7	5	80	acetone	12 h	0
8	5	80	EtOAc	30 min	96[a,c]

[a] NMR yield. [b] Isolated yield. [c] Reaction carried out in presence of 2,6‐di‐*tert*‐butyl pyridine.

With these conditions now in hand, we wanted to explore applicability and limitations of the reaction. We started by examining the substitution on the hemiaminal itself, as this is often less explored in the literature. As shown (Scheme 2), the reaction is tolerant a wide range of electronically diverse substrates. Electron‐withdrawing groups (**1** **b**–**1** **g**) worked well, providing the desired oxazole in good‐to‐excellent yields. Electron‐donating groups were also tolerated (**1** **h**–**1** **j**), once again affording the corresponding 5‐aminooxazole in low (**1** **j**) to very high yields. Furthermore, thiophene (**1** **k**) and naphthyl derivatives gave the desired product in useful yields. Unfortunately all attempts employing saturated derivatives failed under our reaction conditions. Analysis provided some insight, and in most cases, the major product was the bisamide derived from two equivalents of **1** **a**. We are currently investigating this limitation in reactivity, and will report our findings in due course.

**Scheme 2:**
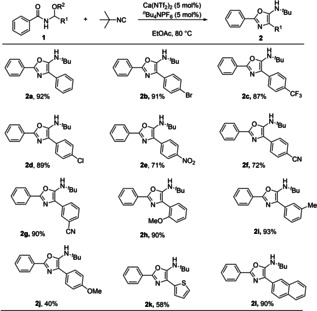
Substrate scope of functionalized hemiaminals.

We then turned our attention to assessing how derivatization of the amide portion of the starting materials affected the reaction (Scheme 3). This proved very successful, with the reaction providing the oxazole routinely in high yield, regardless of electronics (**3** **a**–**3** **f**). Heterocyclic substrates were also well tolerated (**3** **g**–**3** **h**), affording bidentate scaffolds in excellent yield.

**Scheme 3:**
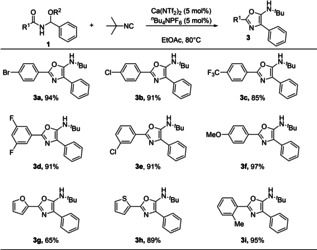
Substrate scope of functionalized amides.

To ensure that the methodology is fully modular, we then focused on varying the isocyanides used in the reaction. Employing **1** **a** as a model substrate, we assessed a range of isocyanides (Scheme 4), including electron withdrawing (**4** **a**–**4** **c**), electron donating (**4** **d**), mixed electronic (**4** **e**), benzyl (**4** **f**) and cyclohexyl (**4** **g**). This also proved fruitful, with a range of structurally diverse oxazoles being formed in good to excellent yields.

**Scheme 4:**
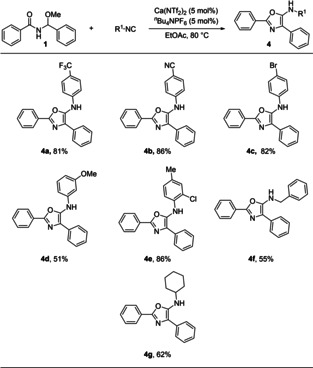
Varying the isocyanide.

Finally, we wanted to explore the possibility of extending this methodology to the synthesis of 5‐aminothiazoles, which have shown promise as a versatile building block in drug discovery and materials chemistry. The synthesis of 2‐aminothiazoles is well established, however their 5‐amino counterparts remain underexplored,^[24]^ with limited modular synthesis of these scaffolds reported. Moreover, the routes towards the structures are plagued with issues relating to sustainability, with toxic or environmentally harmful by‐products formed. To this end, we subjected a series of thioamide derivatives to our optimized conditions, which afforded the desired thiazoles in good to excellent yields (Scheme 5)

**Scheme 5:**
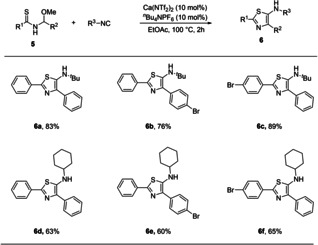
Thiazole Synthesis

Although the catalyst system is not recyclable in the traditional sense, we wanted to explore whether the system could be used in a sequential manner. We therefore decided to run the reaction to completion, followed by recharging the flask with more starting material. We observed minimal reduction in reactivity in the first three recharges; however, the reaction quickly plateaus after this. We reason that this could be due to catalyst poisoning because of increased concentration of amine nucleophile from the oxazole or production of catalytically inactive calcium bisalkoxides, derived from loss of iPrOH.

A plausible reaction mechanism is provided below. (Scheme 6). It is now well established that the active catalyst [CaPF_6_NTf_2_] **A** is formed,^[21a]^ which then reacts to produce the reactive *N*‐acyliminium ion **B**, and subsequent loss of the non‐coordinating PF_6_^−^ anion. This then reacts with the isocyanide to produce nitrillium ion **D**, which after cyclization (**E**) and aromatization provides the desired product with concomitant regeneration of the active catalyst.

**Scheme 6:**
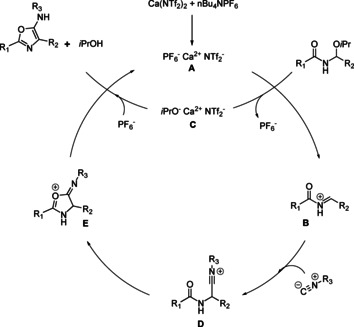
Plausible reaction mechanism.

In summary, we have developed a sustainable, modular and high yielding synthesis of 5‐aminooxazoles. The reaction is tolerant to a range of substituents and functional groups and is typically complete within 30 min. We have also extended the reaction to produce 5‐aminothiazoles in synthetically useful yields

## Experimental Section

### General procedure for the calcium‐catalyzed synthesis of 5‐aminooxazoles

To a 4 mL vial was added the corresponding *N*‐acyl‐*N*,*O*‐acetal **1** (1.0 equiv.) and isocyanide (1.2 equiv) in EtOAc (1 mL). *n*Bu_4_NPF_6_ (5 mol %) and Ca(NTf_2_)_2_ (5 mol %) was added, and the mixture was stirred at 80 °C until TLC analysis indicated complete conversion to the product. The mixture was concentrated and purified by flash column chromatography (EtOAc/hexane, 1 % NEt_3_) to afford the pure product.

### General procedure for the calcium‐catalyzed synthesis of 5‐aminothiazoles

To a 4 mL vial was added the corresponding *N*‐thioacyl‐*N*,*O*‐acetal **5** (1.0 equiv) and isocyanide (1.2 equiv) in EtOAc (1 mL). *n*Bu_4_NPF_6_ (10 mol %) and Ca(NTf_2_)_2_ (10 mol %) was added and the mixture was stirred at 100 °C until TLC analysis indicated complete conversion to the product. The mixture was concentrated and purified by flash column chromatography (EtOAc/hexane, 1 % NEt_3_) to afford the pure product.

## Conflict of interest

The authors declare no conflict of interest.

## Supporting information

As a service to our authors and readers, this journal provides supporting information supplied by the authors. Such materials are peer reviewed and may be re‐organized for online delivery, but are not copy‐edited or typeset. Technical support issues arising from supporting information (other than missing files) should be addressed to the authors.

Supplementary
