# Supplementary material for: Sustainable Access to 5‐Amino‐Oxazoles and Thiazoles via Calcium‐Catalyzed Elimination‐Cyclization with Isocyanides
Source: ChemSusChem. 2021 Mar 3;14(7):1696–9. doi: 10.1002/cssc.202100225 (PMC8048476; doi:10.1002/cssc.202100225)

ChemSusChem

Supporting Information

Sustainable Access to 5-Amino-Oxazoles and Thiazoles via Calcium-Catalyzed Elimination-Cyclization with Isocyanides

Ashley J. Basson and Mark G. McLaughlin*© 2021 The Authors. ChemSusChem published by Wiley-VCH GmbH. This is an open access article under the terms of the Creative Commons Attribution License, which permits use, distribution and reproduction in any medium, provided the original work is properly cited.

Contents

1. General Information 2
2. General Procedures 3
3. Synthesis of *N*-acyl-*N,O*-acetals 5
4. Synthesis of *N*-thioacyl-*N,O*-acetals 15
5. Calcium catalyzed synthesis of 5-aminooxazoles 17
6. Calcium catalyzed synthesis of 5-aminothiazoles 33
7. Catalyst Turnover Studies 37
8. COPIES OF SPECTRA 40

1. General Information

Solvents and reagents

All solvents were purchased from commercial sources and used without purification (HPLC or analytical grade). Anhydrous solvent was obtained from a The Solv™ Solvent Purification System. Standard vacuum line techniques were used and glassware was oven dried prior to use. Organic solvents were dried during workup using anhydrous Na_2SO_4. All calcium catalysed reactions where done without the need for anhydrous or air free conditions.

Purification and chromatography

Thin Layer Chromatography (TLC) was carried out using aluminium plates coated with 60 F254 silica gel. Plates were visualised using UV light (254 or 365 nm) or staining with 1% aq. $KMnO_4$, vanillin or ninhydrin. Normal-phase silica gel chromatography was carried out using either a Biotage Isolera One flash column chromatography system (LPLC) or traditional flash column chromatography using Geduran® Silica gel 60, 40–63 microns RE.

Characterisation

Infrared spectroscopy was carried out with a Nicolet® 380 FT/IR – Fourier Transform Infrared Spectrometer. Only the most significant frequencies have been considered during the characterization and selected absorption maxima (νmax) recorded in wavenumbers (cm^{-1}). NMR spectra were recorded using a JEOL® ECS-400 MHz spectrometer using the deuterated solvent stated. Chemical shifts (δ) quoted in parts per million (ppm) and referenced to the residual solvent peak. Multiplicities are denoted as s- singlet, d- doublet, t- triplet, q- quartet and quin- quintet and derivatives thereof (br denotes a broad resonance peak). Coupling constants recorded as Hz and round to the nearest 0.1 Hz. High Resolution Mass Spectrometry (HRMS) was recorded using an Agilent Technologies® 6540 Ultra-High-Definition (UHD) AccurateMass equipped with a time of flight (Q-TOF) analyser and the samples were ionised by ESI techniques and introduced through a high pressure liquid chromatography (HPLC) model Agilent Technologies® 1260 Infinity Quaternary LC system.

2. General Procedures

General Procedure A – Reported by Manolikakes[1]

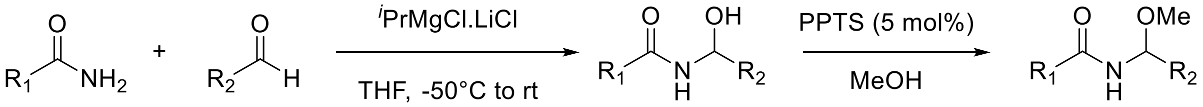

To a flame dried round bottom flask purged with argon was added amide (1.0 equiv.) and dry THF (2mmol/amide). The resulting solution was cooled to -50°C and *i*PrMgCl.LiCl (1.05 equiv.) was added dropwise. The resulting solution was then warmed to room temperature and stirred for 30 min. The mixture was then cooled to 0 °C and the corresponding aldehyde (1.1 equiv.) was added as a single portion. The reaction mixture was warmed to room temperature and stirred until TLC analysis indicated full consumption of amide. The mixture was quenched with sat. aq. $NaHCO_3$, the organic layer separated, and the aqueous layer extracted three times with DCM. The combined organic layers were dried with Na_2SO_4, filtered and concentrated to afford the crude hemiaminal. Pure hemiaminals were obtained by crystallisation from DCM:Hexane (9:1)

The crude hemiaminal was then dissolved in methanol (3mL/mmol) and PPTS (5 mol%) was added. The reaction mixture was stirred at room temperature until complete consumption of the hemiaminal was observed via TLC. The mixture was quenched using sat. aq. $NaHCO_3$. The organic layer was separated, and the aqueous layer was extracted three times with DCM. The combined organic layers were dried with Na_2SO_4, filtered, concentrated and purified by flash column chromatography (EtOAc:Hex, 1% NEt_3) to afford the pure product.

Yields are reported over two steps.

General Procedure B – Adapted literature procedure[2]

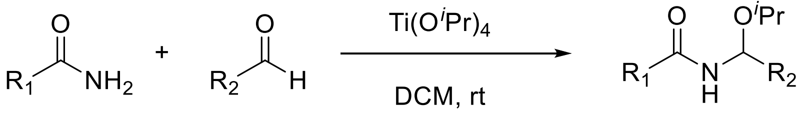

Amide (1.0 equiv.) and aldehyde (1.2 equiv.) were dissolved in anhydrous DCM (0.25M) under an argon atmosphere. $Ti(O^iPr)_4$ (1.5 equiv.) was added dropwise and the reaction was stirred at room temperature overnight. The reaction was then diluted with isopropanol and quenched through dropwise addition of a 0.5M K_2CO_3 solution. The resulting precipitate was then removed via slow filtration through Celite and washed 3 times with isopropanol. The solution was then concentrated, and the resulting solid was purified by flash column chromatography (EtOAc:Hex) to afford the pure product.

*when using benzaldehyde, excess could be removed by washing the solid with hexane prior to column chromatography which resulted in more efficient purification.

General Procedure C – Reported by Katritzky[3]

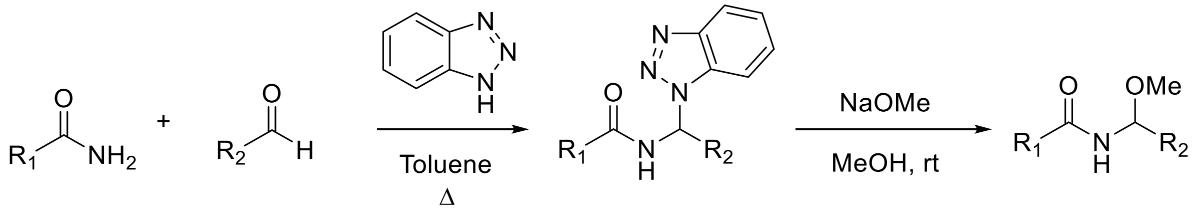

Amide (1 equiv.), aldehyde (1 equiv.) and benzotriazole (1 equiv.) were refluxed in toluene overnight in the presence of 4Å molecular sieves. The solution was cooled, concentrated and Et_2O (15 mL) was added and the resulting solid was filtered and recrystallized from cold methanol. The solid was then suspended in methanol, sodium methoxide (2 equiv.) was added and stirred overnight at room temperature. Water was added and the precipitated solid was collected by filtration.

General Procedure D – Synthesis of *N*-thioacyl-*N,O*-acetals – Adapted Procedure[4]

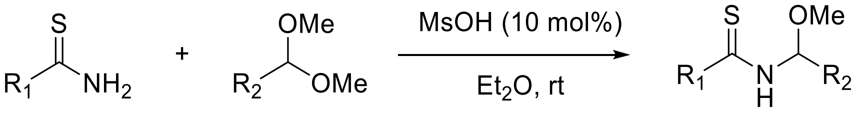

Thioamide (1 equiv.) and dimethyl acetal (2 equiv.) was dissolved in dry Et_2O (0.2 M) and MsOH (0.1 equiv.) was added. The reaction was stirred at room temperature overnight. The reaction was quenched with sat. aq. Na_2CO_3, and the aqueous layer was extracted three times with Et_2O. The combined organic layers were dried over Na_2SO_4, filtered and concentrated. The product was then purified by flash column chromatography (Hex: Et_2O gradient elution) to afford the pure product.

General Procedure E – Calcium catalyzed synthesis of 5-aminooxazoles

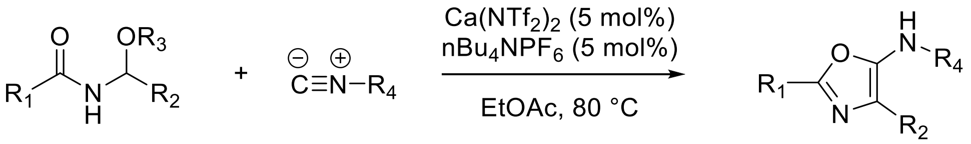

To a 4 mL vial was added the corresponding *N*-Acyl-N,O-acetal **1** (1.0 equiv.) and isocyanide (1.2 equiv.) in EtOAc (1 mL). *n*Bu_4NPF_6 (5 mol%) and $Ca(NTf_2)_2$ (5 mol%) was added and the mixture was stirred at 80°C until TLC analysis indicated complete conversion to the product. The mixture was concentrated and purified by flash column chromatography (EtOAc:Hex, 1% NEt_3) to afford the pure product.

General Procedure F – Calcium catalyzed synthesis of 5-aminothiazoles

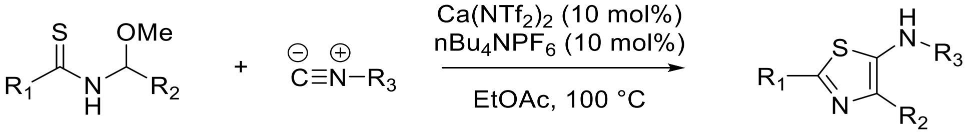

To a 4 mL vial was added the corresponding *N*-thioacyl-N,O-acetal **5** (1.0 equiv) and isocyanide (1.2 equiv) in EtOAc (1 mL). *n*Bu_4NPF_6 (10 mol%) and $Ca(NTf_2)_2$ (10 mol%) was added and the mixture was stirred at 100°C until TLC analysis indicated complete conversion to the product. The mixture was concentrated and purified by flash column chromatography (EtOAc:Hex, 1% NEt_3) to afford the pure product.

3. Synthesis of *N*-acyl-*N,O*-acetals

Aldehyde Variation

N-(Methoxy(phenyl)methyl)benzamide (1a)

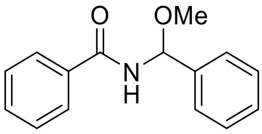

The title compound was prepared according to general procedure **A** from benzamide (1.00 g, 8.25 mmol), *i*PrMgCl.LiCl (6.7 mL, 8.67 mmol, 1.3M in THF) and benzaldehyde (0.93 mL, 9.10 mmol) in THF (20 mL) to afford the hemiaminal. Transacetalisation in MeOH (20 mL) and PPTS (88 mg, 0.35 mmol) followed by column chromatography (1:3 EtOAc:Hex, 1% NEt_3) afforded the pure product as a white solid (1.58 g, 79%)

RF (1:3 EtOAc:Hex): 0.33

^{1}H NMR (400 MHz, DMSO-d_6): δ 9.20 (d, *J* = 9.0 Hz, 1H), 8.04 – 7.89 (m, 2H), 7.59 – 7.53 (m, 1H), 7.51 – 7.45 (m, 4H), 7.42 – 7.36 (m, 2H), 7.36 – 7.30 (m, 1H), 6.28 (d, *J* = 9.0 Hz, 1H), 3.40 (s, 3H).

^{13}C NMR (101 MHz, DMSO-d_6): δ 166.9, 139.8, 133.8, 131.7, 128.3, 128.2, 128.0, 127.7, 126.4, 81.8, 55.3.

*Data in accordance with literature[1]

N-[(4-bromophenyl)(methoxy)methyl]benzamide (1b)

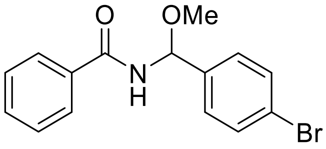

The title compound was prepared according to general procedure **A** from benzamide (400 mg, 3.30 mmol), *i*PrMgCl.LiCl (3.70 mL, 3.50 mmol, 0.94M in THF) and 4-bromo benzaldehyde (670 mg, 3.60 mmol) in THF (7 mL) to afford the hemiaminal. Transacetalisation in MeOH (5 mL) and PPTS (25 mg, 0.1 mmol) followed by column

chromatography (1:4 EtOAc:Hex, 1% NEt_3) afforded the pure product as a white solid (440 mg, 47%)

RF (1:3 EtOAc:Hex): 0.43

^{1}H NMR (400 MHz, DMSO-d_6): δ 9.22 (d, *J* = 8.9 Hz, 1H), 7.99 – 7.92 (m, 2H), 7.63 – 7.52 (m, 3H), 7.52 – 7.40 (m, 4H), 6.25 (d, *J* = 8.9 Hz, 1H), 3.39 (s, 3H).

^{13}C NMR (101 MHz, DMSO-d_6): δ 166.9, 139.3, 133.6, 131.8, 131.1, 128.7, 128.4, 127.7, 121.2, 81.2, 55.3.

*Data in accordance with literature[1]

_N_-{methoxy[4-(trifluoromethyl)phenyl]methyl}benzamide (1c)

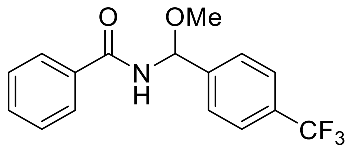

The title compound was prepared according to general procedure **A** from benzamide (300 mg, 2.50 mmol), *i*PrMgCl.LiCl (2 mL, 2.60 mmol, 1.3M in THF) and 4-(Trifluoromethyl)benzaldehyde (475 mg, 2.72 mmol) in THF (6 mL) to afford the hemiaminal. Transacetalisation in MeOH (4 mL) and PPTS (21 mg, 0.085 mmol) followed by column chromatography (1:4 EtOAc:Hex, 1% NEt_3) afforded the pure product as a white solid (365 mg, 53%)

RF (1:3 EtOAc:Hex): 0.42

IR ν_{max} (cm^{-1}): 3275, 3017, 2970, 1642, 1519, 1325,

HRMS (ESI) m/z: $[M – CH_4O]^+$ Calcd for $C_{15}H_{10}F_3NO$ 278.0793; Found 278.0796

^{1}H NMR (400 MHz, DMSO-d_6): δ 9.30 (d, *J* = 8.9 Hz, 1H), 7.98 (d, *J* = 7.4 Hz, 2H), 7.76 (d, *J* = 8.3 Hz, 2H), 7.71 (d, *J* = 8.2 Hz, 2H), 7.57 (t, *J* = 7.3 Hz, 1H), 7.49 (t, *J* = 7.5 Hz, 2H), 6.38 (d, *J* = 8.9 Hz, 1H), 3.44 (s, 3H).

^{13}C NMR (101 MHz, DMSO-d_6): δ 167.1, 144.4, 133.6, 131.8, 128.4, 128.6 (q, *J* = 31.6 Hz), 127. 8, 127.3, 125.2 (d, *J* = 3.6 Hz), 124.3 (q, *J* = 272.1 Hz), 81.1, 55.4.

^{19}F NMR (376 MHz, DMSO-d_6): 60.9

_N_-[(4-chlorophenyl)(methoxy)methyl]benzamide (1d)

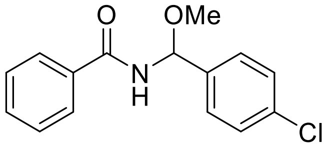

The title compound was prepared according to general procedure **A** from benzamide (300 mg, 2.50 mmol), *i*PrMgCl.LiCl (2 mL, 2.60 mmol, 1.3M in THF) and 4-chloro benzaldehyde (380 mg, 2.70 mmol) in THF (6 mL) to afford the hemiaminal. Transacetalisation in MeOH (4 mL) and PPTS (24 mg, 0.1 mmol) followed by column

chromatography (1:9 EtOAc:Hex, 1% NEt_3) afforded the pure product as a white solid (445 mg, 71%)

RF (1:3 EtOAc:Hex): 0.30

^{1}H NMR (400 MHz, DMSO-d_6): δ 9.24 (d, J = 8.9 Hz, 1H), 8.08 – 7.86 (m, 2H), 7.66 – 7.36 (m, 7H), 6.29 (d, J = 8.9 Hz, 1H), 3.40 (s, 3H).

^{13}C NMR (101 MHz, DMSO-d_6): δ 167.0, 138.9, 133.7, 132.6, 131.7, 128.3, 128.2, 127.8, 81.2, 55.3.

*Data in accordance with literature[1]

***N*-[methoxy(4-nitrophenyl)methyl]benzamide (1e)**

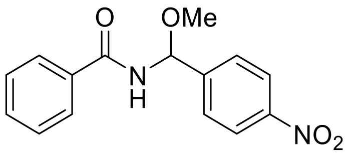

The title compound was prepared according to general procedure **A** from benzamide (300 mg, 2.50 mmol), *i*PrMgCl.LiCl (2 mL, 2.60 mmol, 1.3M in THF) and 4-nitrobenzaldehyde (410 mg, 2.70 mmol) in THF (6 mL) to afford the hemiaminal. Transacetalisation in MeOH (6 mL) and PPTS (23 mg, 0.092 mmol) followed by column chromatography (1:9 EtOAc:Hex, 1% NEt_3) afforded the pure product as a white solid (445 mg, 66%)

RF (1:3 EtOAc:Hex): 0.23

^{1}H NMR (400 MHz, DMSO-d_6): δ 9.36 (d, J = 8.8 Hz, 1H), 8.26 (d, J = 8.6 Hz, 2H), 7.97 (d, J = 7.4 Hz, 2H), 7.75 (d, J = 8.5 Hz, 2H), 7.57 (t, J = 7.2 Hz, 1H), 7.49 (t, J = 7.5 Hz, 2H), 6.41 (d, J = 8.8 Hz, 1H), 3.45 (s, 3H).

^{13}C NMR (101 MHz, DMSO-d_6): δ 167.1, 147.2, 147.1, 133.5, 131.9, 128.4, 127.8, 127.8, 123.4, 80.9, 55.5.

*Data in accordance with literature[1]

***N*-{(4-cyanophenyl)[(propan-2-yl)oxy]methyl}benzamide (1f)**

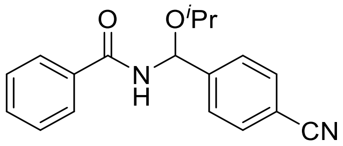

The title compound was prepared according to general procedure **B** from benzamide (250 mg, 2.10 mmol), 4-cyanobenzaldehyde (325 mg, 2.50 mmol) and Ti(O^{i}Pr)$_4$ (880 mg, 3.10 mmol) in DCM (7 mL). Purification by flash column chromatography (0 to 5% EtOAc:CycHex) afforded the pure product as a white solid (299 mg, 59%).

RF (1:5 EtOAc:Hex): 0.26

IR ν_{max} (cm^{-1}): 3375, 3267, 2974, 2231, 1645, 1487, 1048

HRMS (ESI) m/z: $[M – C_3H_8O]^+$ Calcd for $C_{15}H_{11}N_2O$ 235.0871; Found 235.0870

^1H NMR (400 MHz, DMSO-d_6): δ 9.30 (d, J = 8.9 Hz, 1H), 7.99 – 7.91 (m, 2H), 7.86 (d, J = 8.4 Hz, 2H), 7.66 (d, J = 8.1 Hz, 2H), 7.56 (t, J = 7.3 Hz, 1H), 7.48 (t, J = 7.4 Hz, 2H), 6.52 (d, J = 8.9 Hz, 1H), 3.94 (hept, J = 6.1 Hz, 1H), 1.21 (dd, J = 9.5, 6.1 Hz, 6H).

^{13}C NMR (101 MHz, DMSO-d_6): δ 166.7, 145.9, 133.5, 132.2, 131.8, 128.4, 127.7, 127.5, 118.8, 110.6, 77. 5, 68.9, 23.1, 21.6.

***N*-[(3-cyanophenyl)(methoxy)methyl]benzamide (1g)**

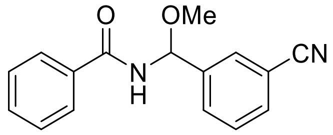

The title compound was prepared according to general procedure **A** from benzamide (300 mg, 2.50 mmol), *i*PrMgCl.LiCl (2.4 mL, 2.60 mmol, 1.097M in THF) and 3-Formylbenzonitrile (360 mg, 2.70 mmol) in THF (5 mL) to afford the hemiaminal. Transacetalisation in MeOH (5 mL) and PPTS (20 mg, 0.08 mmol) followed by column chromatography (3:10 EtOAc:Hex, 1% NEt_3) afforded the pure product as a white solid (350 mg, 64%)

RF (3:10 EtOAc:Hex): 0.48

IR ν_{max} (cm^{-1}): 3246, 2950, 2232, 1637, 1515, 1108, 1046

HRMS (ESI) m/z: $[M + Na]^+$ Calcd for $C_{16}H_{14}N_2O_2Na$ 289.0953; Found 289.0950

^1H NMR (400 MHz, DMSO-d_6): δ 9.19 (d, J = 9.0 Hz, 1H), 7.98 – 7.93 (m, 2H), 7.59 – 7.53 (m, 1H), 7.50 – 7.44 (m, 4H), 7.41 – 7.36 (m, 2H), 7.35 – 7.29 (m, 1H), 6.27 (d, J = 9.0 Hz, 1H), 3.39 (s, 3H).

^{13}C NMR (101 MHz, DMSO-d_6): δ 167.0, 141.3, 133.5, 131.9, 131.8, 131.5, 130.1, 129.6, 128.4, 127.8, 118.8, 111.2, 80.9, 55.4.

***N*-[methoxy(2-methoxyphenyl)methyl]benzamide (1h)**

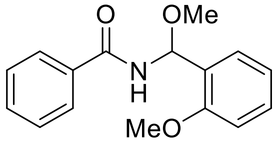

The title compound was prepared according to general procedure **C** from benzamide (1 g, 8.30 mmol), *o*-anisaldehyde (1.10 g, 8.30 mmol) and benzotriazole (980 mg, 8.30 mmol) in toluene (4 mL). The product (622 mg, 1.75 mmol) was then stirred in methanol (12 mL) with sodium methoxide (110 mg, 2.10 mmol) overnight to afford, after recrystilization, the title compound as a white solid (187 mg, 40%).

RF (1:5 EtOAc:Hex): 0.16

IR ν_{max} (cm^{-1}): 3315, 3073, 2940, 2837, 1638, 1517, 1488, 1242, 1027

HRMS (ESI) m/z: $[M + Na]^+$ Calcd for $C_{16}H_{17}NO_3Na$ 294.1106; Found 294.1101

^1H NMR (400 MHz, DMSO-d_6): δ 8.97 (d, *J* = 8.9 Hz, 1H), 7.91 (d, *J* = 7.2 Hz, 2H), 7.59 (dd, *J* = 7.6, 1.5 Hz, 1H), 7.54 (t, *J* = 7.3 Hz, 1H), 7.46 (t, *J* = 7.5 Hz, 2H), 7.36 – 7.29 (m, 1H), 7.06 – 6.90 (m, 2H), 6.47 (d, *J* = 9.0 Hz, 1H), 3.78 (s, 3H), 3.34 (s, 3H).

^{13}C NMR (101 MHz, DMSO-d_6): δ 166.6, 156.5, 134.0, 131.5, 129.4, 128.3, 127.7, 127.4, 127.2, 120.1, 111.0, 77.2, 55.6, 55.3.

***N*-{(3-methylphenyl)[(propan-2-yl)oxy]methyl}benzamide (1i)**

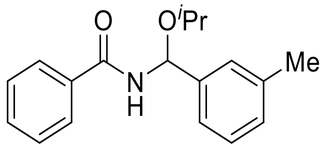

The title compound was prepared according to general procedure **B** from benzamide (250 mg, 2.10 mmol), *m*-Tolualdehyde (300 mg, 2.50 mmol) and Ti(O^{i}Pr)$_4$ (880 mg, 3.10 mmol) in DCM (8 mL). Purification by flash column chromatography (1:20 EtOAc:Hex) afforded the pure product as a white solid (195 mg, 33%).

RF (1:5 EtOAc:Hex): 0.45

IR ν_{max} (cm^{-1}): 3293, 2970, 1637, 1520, 1276, 1038

HRMS (ESI) m/z: $[M + Na]^+$ Calcd for $C_{18}H_{21}NNaO_2$ 306.1470; Found 306.1468

^1H NMR (400 MHz, DMSO-d_6): δ 9.15 (d, *J* = 9.0 Hz, 1H), 7.95 (d, *J* = 7.1 Hz, 2H), 7.59 – 7.52 (m, 1H), 7.50 – 7.44 (m, 2H), 7.34 – 7.20 (m, 3H), 7.17 – 7.09 (m, 1H), 6.43 (d, *J* = 8.9 Hz, 1H), 3.89 (hept, *J* = 6.1 Hz, 1H), 2.31 (s, 3H), 1.20 (dd, *J* = 8.9, 6.1 Hz, 6H).

^{13}C NMR (101 MHz, DMSO-d_6): δ 166.5, 140.5, 137.2, 133.8, 131.6, 128.4, 128.3, 128.0, 127.7, 126.9, 123.5, 78.0, 68.3, 23.2, 21.6, 21.1.

***N*-[methoxy(4-methoxyphenyl)methyl]benzamide (1j)**

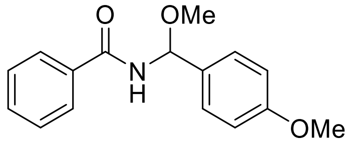

The title compound was prepared according to general procedure **C** from benzamide (1 g, 8.30 mmol), p-anisaldehyde (1.1 g, 8.30 mmol) and benzotriazole (980 mg, 8.30 mmol) in toluene (4 mL). The product (745 mg, 2.10 mmol) was then stirred in methanol (15 mL) with sodium methoxide (135 mg, 2.50 mmol) overnight to afford, after recrystilization, the title compound as a white solid (62 mg, 11%).

RF (1:5 EtOAc:Hex): 0.24

^{1}H NMR (400 MHz, DMSO-d_6): δ 9.12 (d, *J* = 8.9 Hz, 1H), 7.97 – 7.92 (m, 2H), 7.55 (t, *J* = 7.3 Hz, 1H), 7.47 (t, *J* = 7.5 Hz, 2H), 7.40 (d, *J* = 8.6 Hz, 2H), 6.21 (d, *J* = 9.0 Hz, 1H), 3.75 (s, 3H), 3.36 (s, 3H).

^{13}C NMR (101 MHz, DMSO-d_6): δ 166.8, 159.0, 133.8, 131.9, 131.6, 128.3, 127.7, 127.7, 113.5, 81.6, 55.1, 55.1.

*data in accordance with literature[3]

***N*-{[(propan-2-yl)oxy](thiophen-2-yl)methyl}benzamide (1k)**

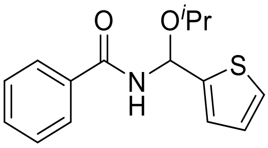

The title compound was prepared according to general procedure **B** from benzamide (250 mg, 2.10 mmol), 2-Thiophenecarboxaldehyde (280 mg, 2.50 mmol) and Ti(O^{i}Pr)$_4$ (880 mg, 3.10 mmol) in DCM (8 mL). Purification by flash column chromatography (0 to 5% EtOAc:Hex) afforded the pure product as a white solid (190 mg, 33%).

RF (1:5 EtOAc:Hex): 0.45

IR $ν_{max}$ (cm^{-1}): 3247, 2970, 1636, 1522, 1362, 1045

HRMS (ESI) m/z: [M + Na]$^+$ Calcd for $C_{15}H_{17}NNaO_2S$ 298.0878; Found 298.0881

^{1}H NMR (400 MHz, DMSO-d_6): δ 9.35 (d, *J* = 8.9 Hz, 1H), 7.91 (d, *J* = 7.1 Hz, 2H), 7.54 – 7.47 (m, 1H), 7.46 – 7.38 (m, 3H), 7.00 – 6.98 (m, 1H), 6.95 (dd, *J* = 5.0, 3.5 Hz, 1H), 6.59 (d, *J* = 8.3 Hz, 1H), 3.87 (hept, *J* = 6.1 Hz, 1H), 1.17 (d, *J* = 6.0 Hz, 3H), 1.11 (d, *J* = 6.2 Hz, 3H).

^{13}C NMR (101 MHz, DMSO-d_6): δ 166.5, 144.5, 133.6, 131.8, 128.3, 127.8, 126.8, 125.7, 124.6, 75.4, 68.9, 23.1, 21.6.

***N*-{(naphthalen-2-yl)[(propan-2-yl)oxy]methyl}benzamide (1l)**

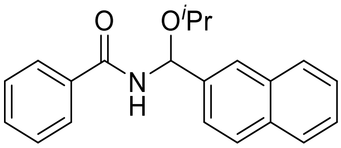

The title compound was prepared according to general procedure **B** from benzamide (290 mg, 2.40 mmol), 2-Naphthaldehyde (250 mg, 1.60 mmol) and Ti(O^{i}Pr)$_4$ (910 mg, 3.20 mmol) in DCM (7 mL). Purification by flash column chromatography (1:9 EtOAc:Hex) afforded the pure product as a white solid (128 mg, 25%).

RF (1:5 EtOAc:Hex): 0.39

IR $ν_{max}$ (cm^{-1}): 3298, 3056, 2696, 1638, 1517, 1365, 1072

HRMS (ESI) m/z: [M + Na]$^+$ Calcd for $C_{21}H_{21}NNaO_2$ 342.1470; Found 342.1468

^{1}H NMR (400 MHz, DMSO-d$_6$): δ 9.29 (d, *J* = 8.9 Hz, 1H), 8.01 – 7.87 (m, 6H), 7.62 (dd, *J* = 8.5, 1.7 Hz, 1H), 7.59 – 7.43 (m, 5H), 6.63 (d, *J* = 8.9 Hz, 1H), 3.97 (hept, *J* = 6.1 Hz, 1H), 1.25 (dd, *J* = 9.5, 6.1 Hz, 6H).

^{13}C NMR (101 MHz, DMSO-d$_6$): δ 66.6, 138.1, 133.8, 132.6, 132.6, 131.7, 128.3, 128.1, 127.8, 127.8, 127.5, 126.3, 126.2, 124.9, 124.8, 78.2, 68.5, 23.2, 21.7.

Amide Variation

4-bromo-*N*-{phenyl[(propan-2-yl)oxy]methyl}benzamide (1m)

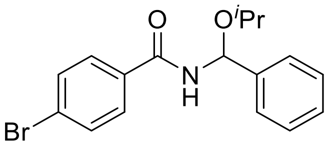

The title compound was prepared according to general procedure **B** from 4-bromobenzamide (662 mg, 3.30 mmol), benzaldehyde (421 mg, 4.00 mmol) and Ti(O^{i}Pr)$_4$ (1.40 g, 5.00 mmol) in DCM (10 mL). Purification by flash column chromatography (0 to 10% EtOAc:Hex) afforded the pure product as a white solid (188 mg, 16%).

RF (1:5 EtOAc:Hex): 0.52

IR ν_{max} (cm^{-1}): 3289, 2971, 1639, 1520, 1482, 1011, 696

HRMS (ESI) m/z: [M – C_3H_8O]$^+$ Calcd for $C_{14}H_{11}BrNO$ 288.0024; Found 288.0025

^{1}H NMR (400 MHz, DMSO-d$_6$): δ 9.28 (d, *J* = 8.9 Hz, 1H), 7.89 (d, *J* = 8.6 Hz, 2H), 7.69 (d, *J* = 8.6 Hz, 2H), 7.47 (d, *J* = 7.1 Hz, 2H), 7.37 (t, *J* = 7.3 Hz, 2H), 7.34 – 7.28 (m, 1H), 6.44 (d, *J* = 8.8 Hz, 1H), 3.90 (hept, *J* = 6.1 Hz, 1H), 1.20 (dd, *J* = 9.3, 6.1 Hz, 6H).

^{13}C NMR (101 MHz, DMSO-d$_6$): δ 165.7, 140.4, 132.9, 131.4, 129.9, 128.2, 127.9, 126.4, 125.4, 78.1, 68.5, 23.1, 21.6.

4-chloro-*N*-{phenyl[(propan-2-yl)oxy]methyl}benzamide (1n)

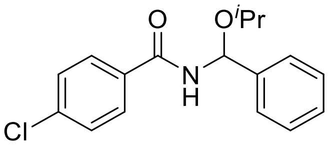

The title compound was prepared according to general procedure **B** from 4-chlorobenzamide (500 mg, 3.20 mmol), benzaldehyde (410 mg, 3.90 mmol) and Ti(O^{i}Pr)$_4$ (1.40 g, 4.80 mmol) in DCM (13 mL). Purification by flash column chromatography (0 to 10% EtOAc:Hex) afforded the pure product as a white solid (222 mg, 23%).

RF (1:5 EtOAc:Hex): 0.54

IR ν_{max} (cm^{-1}): 3263, 3064, 2974, 1635, 1532, 1486, 1031,850

HRMS (ESI) m/z: [M + C_3H_8O]$^+$ Calcd for $C_{17}H_{19}ClNO$ 244.0529; Found 244.0534

^{1}H NMR (400 MHz, DMSO-d_6): δ 9.30 (d, *J* = 8.9 Hz, 1H), 7.97 (d, *J* = 8.6 Hz, 2H), 7.55 (d, *J* = 8.6 Hz, 2H), 7.47 (d, *J* = 7.3 Hz, 2H), 7.38 (t, *J* = 7.3 Hz, 2H), 7.35 – 7.28 (m, 1H), 6.44 (d, *J* = 8.9 Hz, 1H), 3.89 (hept, *J* = 6.1 Hz, 1H), 1.20 (dd, *J* = 10.1, 6.1 Hz, 6H).

^{13}C NMR (101 MHz, DMSO-d_6): δ 165.6, 140.4, 136.5, 132.6, 129.7, 128.4, 128.1, 127.9, 126.4, 78.1, 68.56, 23.1, 21.6.

N-{phenyl[(propan-2-yl)oxy]methyl}-4-(trifluoromethyl)benzamide (1o)

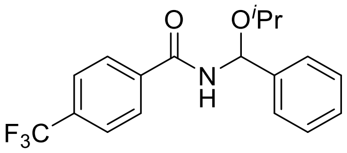

The title compound was prepared according to general procedure **B** from 4-(trifluoromethyl)benzamide (400 mg, 2.10 mmol), benzaldehyde (270 mg, 2.50 mmol) and Ti(O^{i}Pr)$_4$ (900 mg, 3.20 mmol) in DCM (9 mL). Purification by flash column chromatography (0 to 5% EtOAc:Hex) afforded the pure product as a white solid (206 mg, 30%).

RF (1:5 EtOAc:Hex): 0.51

IR ν_{max} (cm^{-1}): 3282, 2977, 1652, 1534, 1326, 1126, 859

HRMS (ESI) m/z: [M – C_3H_8O]$^+$ Calcd for $C_{15}H_{11}F_3NO$ 278.0793; Found 278.0792

^{1}H NMR (400 MHz, DMSO-d_6): δ 9.48 (d, *J* = 8.8 Hz, 1H), 8.13 (d, *J* = 8.0 Hz, 2H), 7.86 (d, *J* = 8.3 Hz, 2H), 7.48 (d, *J* = 7.2 Hz, 2H), 7.38 (t, *J* = 7.3 Hz, 2H), 7.35 – 7.29 (m, 1H), 6.46 (d, *J* = 8.8 Hz, 1H), 3.92 (hept, *J* = 6.0 Hz, 1H), 1.21 (dd, *J* = 9.9, 6.1 Hz, 6H).

^{13}C NMR (101 MHz, DMSO-d_6): δ 165.5, 140.3, 137.6, 131.4 (q, *J* = 31.8 Hz), 128.6, 128.2, 127.9, 126.4, 125.3 (d, *J* = 3.7 Hz), 124.0 (q, *J* = 272.9 Hz), 78.2, 68.6, 23.1, 21.6.

^{19}F NMR (376 MHz, DMSO-d_6): 61.27

3,5-difluoro-N-{phenyl[(propan-2-yl)oxy]methyl}benzamide (1p)

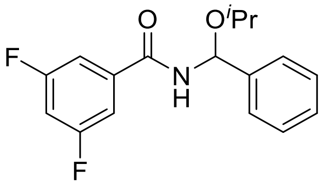

The title compound was prepared according to general procedure **B** from 3,5-difluorobenzamide (400 mg, 2.60 mmol), benzaldehyde (324 mg, 3.06 mmol) and Ti(O^{i}Pr)$_4$ (1.10 g, 3.80 mmol) in DCM (10 mL). Purification by flash column chromatography (0 to 5% EtOAc:Hex) afforded the pure product as a white solid (275 mg, 35%).

RF (1:5 EtOAc:Hex): 0.65

IR ν_{max} (cm^{-1}): 3264, 3063, 2970, 1654, 1534, 1330, 1119

HRMS (ESI) m/z: $[M – C_3H_8O]^+$ Calcd for $C_{14}H_{10}F_2NO$ 246.0730; Found 246.0729

^1H NMR (400 MHz, DMSO-d_6): δ 9.39 (d, J = 8.7 Hz, 1H), 7.68 (dd, J = 8.5, 2.3 Hz, 2H), 7.54 – 7.44 (m, 3H), 7.41 – 7.36 (m, 2H), 7.35 – 7.29 (m, 1H), 6.44 (d, J = 8.7 Hz, 1H), 3.89 (hept, J = 6.1 Hz, 1H), 1.20 (dd, J = 8.5, 6.1 Hz, 6H).

^{13}C NMR (101 MHz, DMSO-d_6): δ 163.9, 163.5 (d, J = 12.7 Hz), 161.0 (d, J = 12.6 Hz), 140.2, 137.2 (t, J = 8.4 Hz), 128.2, 128.0, 126.4, 111.2 (d, J = 7.2 Hz), 111.0 (d, J = 7.2 Hz), 107.2 (t, J = 25.9 Hz), 78.2, 68.6, 23.1, 21.6.

^{19}F NMR (376 MHz, DMSO-d_6): 108.86

3-chloro-*N*-{phenyl[(propan-2-yl)oxy]methyl}benzamide (1q)

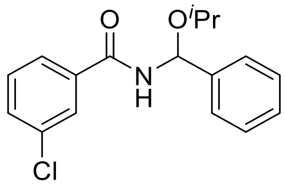

The title compound was prepared according to general procedure **B** from 3-chlorobenzamide (400 mg, 2.60 mmol), benzaldehyde (327 mg, 3.09 mmol) and Ti(O^{i}Pr)$_4$ (1.10 g, 3.90 mmol) in DCM (10 mL). Purification by flash column chromatography (0 to 5% EtOAc:Hex) afforded the pure product as a white solid (136 mg, 17%).

RF (1:5 EtOAc:Hex): 0.46

IR ν_{max} (cm^{-1}): 3290, 3031, 2970, 1644, 1523, 1030, 692

HRMS (ESI) m/z: $[M – C_3H_7O]^+$ Calcd for $C_{14}H_{11}ClNO$ 244.0529; Found 244.0533

^1H NMR (400 MHz, DMSO-d_6): δ 9.35 (d, J = 8.8 Hz, 1H), 8.01 (s, 1H), 7.92 (d, J = 7.8 Hz, 1H), 7.67 – 7.59 (m, 1H), 7.56 – 7.44 (m, 3H), 7.38 (t, J = 7.3 Hz, 2H), 7.32 (t, J = 7.1 Hz, 1H), 6.44 (d, J = 8.8 Hz, 1H), 3.90 (hept, J = 6.1 Hz, 1H), 1.20 (dd, J = 8.4, 6.2 Hz, 6H).

^{13}C NMR (101 MHz, DMSO-d_6): δ 165.2, 140.4, 135.7, 133.2, 131.5, 130.4, 128.2, 127.9, 127.5, 126.5, 126.4, 78.1, 68.5, 23.1, 21.6.

4-methoxy-*N*-{phenyl[(propan-2-yl)oxy]methyl}benzamide (1r)

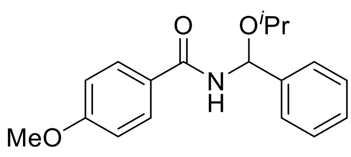

The title compound was prepared according to general procedure **B** from 4-methoxybenzamide (400 mg, 2.65 mmol), benzaldehyde (337 mg, 3.20 mmol) and Ti(O^{i}Pr)$_4$ (1.10 g, 4.00 mmol) in DCM (11 mL). Purification by flash column

chromatography (0 to 5% EtOAc:Hex) afforded the pure product as a white solid (206 mg, 26%).

RF (1:5 EtOAc:Hex): 0.29

IR ν_{max} (cm^{-1}): 3319, 3065, 2971, 1631, 1495, 1257, 849

HRMS (ESI) m/z: $[M – C_3H_8O]^+$ Calcd for $C_{15}H_{14}NO_2$ 240.1025; Found 240.1024

^{1}H NMR (400 MHz, DMSO-d_6): δ 9.04 (d, *J* = 9.0 Hz, 1H), 7.96 (d, *J* = 8.8 Hz, 2H), 7.46 (d, *J* = 7.2 Hz, 2H), 7.41 – 7.27 (m, 3H), 7.00 (d, *J* = 9.0 Hz, 2H), 6.45 (d, *J* = 9.0 Hz, 1H), 3.89 (hept, *J* = 8.0 Hz, 1H), 3.81 (s, 3H), 1.19 (dd, *J* = 11.4, 6.1 Hz, 6H).

^{13}C NMR (101 MHz, DMSO-d_6): δ 166.0, 161.9, 140.8, 129.6, 128.1, 127.8, 126.4, 125.9, 113.5, 77.9, 68.3, 55.4, 23.2, 21.7.

_N_-{phenyl[(propan-2-yl)oxy]methyl}furan-2-carboxamide (1s)

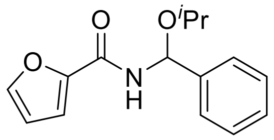

The title compound was prepared according to general procedure **B** from furan-2-carboxamide (400 mg, 3.60 mmol), benzaldehyde (460 mg, 4.32 mmol) and $Ti(O^iPr)_4$ (1.50 g, 5.40 mmol) in DCM (12 mL). Purification by flash column chromatography (0 to 5% EtOAc:Hex) afforded the pure product as a white solid (200 mg, 21%).

RF (1:9 EtOAc:Hex): 0.21

IR ν_{max} (cm^{-1}): 3224, 3047, 2969, 1646, 1531, 1024, 752

HRMS (ESI) m/z: $[M – C_3H_8O]^+$ Calcd for $C_{12}H_{10}NO_2$ 200.0712; Found 200.0711

^{1}H NMR (400 MHz, DMSO-d_6): δ 9.07 (d, *J* = 9.1 Hz, 1H), 7.87 (s, 1H), 7.46 (d, *J* = 7.3 Hz, 2H), 7.41 – 7.27 (m, 4H), 6.65 – 6.62 (m, 1H), 6.39 (d, *J* = 9.1 Hz, 1H), 3.99 – 3.79 (m, 1H), 1.19 (dd, *J* = 8.3, 6.2 Hz, 6H).

^{13}C NMR (101 MHz, DMSO-d_6): δ 157.9, 147.1, 145.7, 140.4, 128.2, 127.9, 126.4, 114.4, 111.9, 77.2, 68.3, 23.1, 21.6.

_N_-{phenyl[(propan-2-yl)oxy]methyl}thiophene-2-carboxamide (1t)

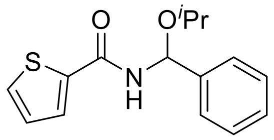

The title compound was prepared according to general procedure **B** from thiophene-2-carboxamide (400 mg, 3.15 mmol), benzaldehyde (400 mg, 3.80 mmol) and $Ti(O^iPr)_4$ (1.30 g, 4.70 mmol) in DCM (13 mL). Purification by flash column chromatography (0 to 5% EtOAc:Hex) afforded the pure product as a white solid (206 mg, 24%).

RF (1:5 EtOAc:Hex): 0.40

IR ν_{max} (cm^{-1}): 3330, 3090, 2969, 1625, 1533, 1030, 745

HRMS (ESI) m/z: $[M – C_3H_8O]^+$ Calcd for $C_{15}H_{18}NO_2S$ 216.0483; Found 216.0478

^{1}H NMR (400 MHz, DMSO-d_6): δ 9.25 (d, J = 9.0 Hz, 1H), 8.00 (d, J = 3.8 Hz, 1H), 7.81 (d, J = 5.0 Hz, 1H), 7.47 (d, J = 7.4 Hz, 2H), 7.38 (t, J = 7.4 Hz, 2H), 7.32 (t, J = 7.2 Hz, 1H), 7.18 – 7.14 (m, 1H), 6.41 (d, J = 9.0 Hz, 1H), 3.90 (hept, J = 6.1 Hz, 1H), 1.20 (dd, J = 8.0, 6.2 Hz, 6H).

^{13}C NMR (101 MHz, DMSO-d_6): δ 161.4, 140.4, 139.4, 131.8, 129.1, 128.2, 128.1, 127.9, 126.3, 77. 8, 68.4, 23.1, 21.6.

2-methyl-*N*-{phenyl[(propan-2-yl)oxy]methyl}benzamide (1u)

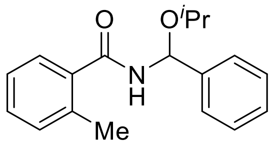

The title compound was prepared according to general procedure **B** from 2-methylbenzamide (250 mg, 1.85 mmol), benzaldehyde (236 mg, 2.22 mmol) and Ti(O^{i}Pr)$_4$ (790 mg, 2.80 mmol) in DCM (7 mL). Purification by flash column chromatography (0 to 5% EtOAc:Hex) afforded the pure product as a white solid (72 mg, 14%).

RF (1:5 EtOAc:Hex): 0.33

IR ν_{max} (cm^{-1}): 3260, 3065, 2969, 1646, 1516, 1041, 727

HRMS (ESI) m/z: $[M – C_3H_8O]^+$ Calcd for $C_{15}H_{14}NO$ 224.1075; Found 224.1069

^{1}H NMR (400 MHz, DMSO-d_6): δ δ 9.12 (d, J = 9.1 Hz, 1H), 7.48 (d, J = 7.4 Hz, 2H), 7.43 – 7.28 (m, 5H), 7.28 – 7.18 (m, 2H), 6.40 (d, J = 9.2 Hz, 1H), 4.05 – 3.94 (m, 1H), 2.35 (s, 3H), 1.23 (dd, J = 9.3, 6.2 Hz, 6H).

^{13}C NMR (101 MHz, DMSO-d_6): δ 169.4, 140.5, 136.7, 135.1, 130.4, 129. 5, 128.1, 127.8, 127.1, 126.3, 125.5, 77.5, 68.3, 23.2, 21.6, 19.5.

4. Synthesis of *N*-thioacyl-*N,O*-acetals

***N*-[methoxy(phenyl)methyl]benzenecarbothioamide (5a)**

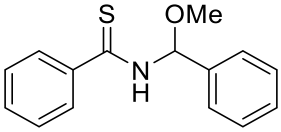

Title compound was prepared according to general procedure **D** from thiobenzamide (300 mg, 2.20 mmol), benzaldehyde dimethyl acetal (670 mg, 4.40 mmol) and MsOH (21 mg, 0.22 mmol) in Et_2O (11 mL). Work up and purification by flash column

chromatography (Hex to 5% Et_2O/Hex) afforded the pure product as a yellow solid (136 mg, 24%).

RF (1:5 EtOAc/Hex) = 0.52

IR ν_{max} (cm^{-1}): 3240, 2990, 2834, 1512, 1446, 1352, 1066, 743

HRMS (ESI) m/z: $[M – CH_4O]^+$ Calcd for $C_{14}H_{12}NS$ 226.0690; Found 226.0693

^1H NMR (400 MHz, DMSO-d_6): δ 11.01 (d, *J* = 7.8 Hz, 1H), 7.77 (d, *J* = 7.4 Hz, 2H), 7.58 – 7.46 (m, 3H), 7.46 – 7.28 (m, 5H), 6.88 (d, *J* = 7.9 Hz, 1H), 3.51 (s, 3H).

^{13}C NMR (101 MHz, DMSO-d_6): δ 199.5, 141.0, 138.5, 131.0, 128.5, 128.3, 128.0, 127.6, 126.4, 87.5, 56.2.

_N_-[(4-bromophenyl)(methoxy)methyl]benzenecarbothioamide (5b)

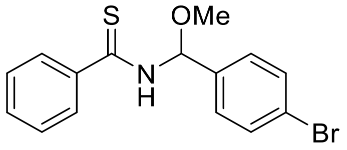

Title compound was prepared according to general procedure **D** from thiobenzamide (300 mg, 2.20 mmol), benzaldehyde dimethyl acetal (1.00 g, 4.40 mmol) and MsOH (21 mg, 0.022 mmol) in Et_2O (11 mL). Work up and purification by flash column chromatography (5% EtOAc/Hex, 1% NEt_3) afforded the pure product as a yellow solid (247 mg, 34%).

RF (1:5 EtOAc/Hex) = 0.43

IR ν_{max} (cm^{-1}): 3275, 3059, 3024, 1593, 1504, 1484, 1352, 1067, 688

HRMS (ESI) m/z: $[M – CH_3O]^+$ Calcd for $C_{14}H_{11}BrNS$ 303.9796; Found 303.9814

^1H NMR (400 MHz, DMSO-d_6): δ 11.00 (d, *J* = 7.7 Hz, 1H), 7.80 – 7.73 (m, 2H), 7.61 (d, *J* = 8.4 Hz, 2H), 7.51 (t, *J* = 7.3 Hz, 1H), 7.49 – 7.39 (m, 4H), 6.84 (d, *J* = 7.7 Hz, 1H), 3.51 (s, 3H).

^{13}C NMR (101 MHz, DMSO-d_6): δ 199.6, 140.8, 137.9, 131.3, 131.1, 128.7, 128.0, 127.6, 121.6, 86.9, 56.3.

4-bromo-_N_-[methoxy(phenyl)methyl]benzene-1-carbothioamide (5c)

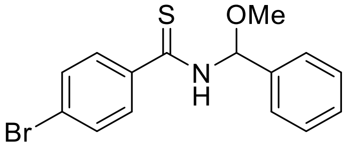

Title compound was prepared according to general procedure **D** from 4-bromothiobenzamide (514 mg, 2.40 mmol), benzaldehyde dimethyl acetal (724 mg, 4.80 mmol) and MsOH (23 mg, 0.024 mmol) in Et_2O (12 mL). Work up and purification by flash column chromatography (1% EtOAc/Hex, 1% NEt_3) afforded the pure product as a yellow oil which solidifies to a yellow solid (289 mg, 36%).

RF (1:5 EtOAc/Hex) = 0.52

IR ν_{max} (cm^{-1}): 3248, 3027, 2927, 1585, 1395, 1246, 1060, 827

HRMS (ES) m/z: $[M – CH_4O]^+$ Calcd for $C_{14}H_{11}BrNS$ 303.9796; Found 303.9801

^1H NMR (400 MHz, DMSO-d_6): δ 11.09 (d, *J* = 6.9 Hz, 1H), 7.70 (d, *J* = 8.6 Hz, 2H), 7.64 (d, *J* = 8.7 Hz, 2H), 7.53 – 7.47 (m, 2H), 7.44 – 7.31 (m, 3H), 6.83 (d, *J* = 7.1 Hz, 1H), 3.50 (s, 3H).

^{13}C NMR (101 MHz, DMSO-d_6): δ 198.0, 139.9, 138.3, 130.9, 129.6, 128.5, 128.3, 126.4, 124.7, 87.6, 56.3.

5. Calcium catalyzed synthesis of 5-aminooxazoles

Aldehyde Variation

N-(tert-butyl)-2,4-diphenyloxazol-5-amine (2a)

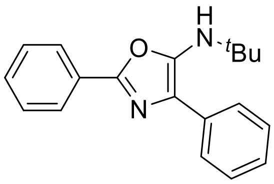

The title compound was prepared according to general procedure **E**, from **1a** (100 mg, 0.4 mmol), $Ca(NTf_2)_2$ (12 mg, 0.02 mmol), nBu_4NPF_6 (8 mg, 0.02 mmol) and tert-butyl isocyanide (38 mg, 0.5 mmol) in EtOAc (2 mL).

Following conversion to the product (1h) and flash column chromatography (1:5 EtOAc:Hex, 1% NEt_3) the pure product was obtained as a white solid (121 mg, 92%)

RF (3:1 EtOAc/Hex) = 0.73

^1H NMR (400 MHz, DMSO-d_6): δ 7.99 (d, *J* = 7.3 Hz, 2H), 7.96 – 7.92 (m, 2H), 7.57 – 7.45 (m, 3H), 7.44 – 7.38 (m, 2H), 7.28 – 7.20 (m, 1H), 5.44 (s, 1H), 1.30 (s, 9H).

^{13}C NMR (101 MHz, DMSO-d_6): δ 153.9, 148.7, 132.4, 129.8, 129.2, 128.3, 127.4, 126.3, 125.3, 125.1, 124.4, 53.2, 30.1.

*data in accordance with literature[5]

4-(4-bromophenyl)-N-(tert-butyl)-2-phenyloxazol-5-amine (2b)

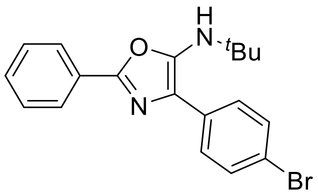

The title compound was prepared according to general procedure **E**, from **1b** (100 mg, 0.31 mmol), $Ca(NTf_2)_2$ (9 mg, 0.016 mmol), nBu_4NPF_6 (6 mg, 0.016 mmol) and tert-butyl isocyanide (31 mg, 0.38 mmol) in EtOAc (2 mL).

Following conversion to the product (30 min) and flash column chromatography (1:5 EtOAc/Hex, 1% NEt_3) the pure product was obtained as a white solid (105 mg, 91%)

RF (1:5 EtOAc/Hex, 1% NEt_3) = 0.43

^{1}H NMR (400 MHz, DMSO-d_6): δ 7.97 – 7.90 (m, 4H), 7.59 (d, *J* = 8.6 Hz, 2H), 7.56 – 7.44 (m, 3H), 5.58 (s, 1H), 1.30 (s, 9H).

^{13}C NMR (101 MHz, DMSO-d_6): δ 153.9, 149.1, 131.7, 131.2, 129.9, 129.2, 127.2, 125.1, 123.0, 118.99, 53.3, 30.0.

*data in accordance with literature[5]

***N*-tert-butyl-2-phenyl-4-[4-(trifluoromethyl)phenyl]-1,3-oxazol-5-amine (2c)**

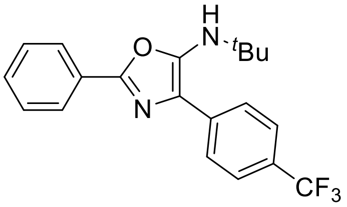

The title compound was prepared according to general procedure **E**, from **1c** (100 mg, 0.32 mmol), $Ca(NTf_2)_2$ (10 mg, 0.016 mmol), nBu_4NPF_6 (6 mg, 0.016 mmol) and tert-butyl isocyanide (32 mg, 0.39 mmol) in EtOAc (2 mL).

Following conversion to the product (1h 30 min) and flash column chromatography (1:9 EtOAc/Hex, 1% NEt_3) the pure product was obtained as a yellow solid (101 mg, 87%)

RF (1:9 EtOAc/Hex, 1% NEt_3) = 0.38

IR ν_{max} (cm^{-1}): 3259, 2972, 1635, 1323, 1103, 849

HRMS (ESI) m/z: $[M + H]^+$ Calcd for $C_{20}H_{20}F_3N_2O$ 361.1528; Found 361.1527

^{1}H NMR (400 MHz, DMSO-d_6): δ 8.13 (d, *J* = 8.2 Hz, 2H), 7.94 (d, *J* = 7.1 Hz, 2H), 7.74 (d, *J* = 8.4 Hz, 2H), 7.57 – 7.45 (m, 3H), 5.88 (s, 1H), 1.34 (s, 9H).

^{13}C NMR (101 MHz, DMSO-d_6): δ 153.5, 150.3, 136.6, 129.9, 129.2, 127.1, 125.9 (q, *J* = 31.8 Hz), 125.3, 125.3 (d, *J* = 3.9 Hz), 125.1, 124.5 (q, *J* = 271.7 Hz), 121.0, 53.3, 30.0.

^{19}F NMR (376 MHz, DMSO-d_6): 60.59

***N-tert*-butyl-4-(4-chlorophenyl)-2-phenyl-1,3-oxazol-5-amine (2d)**

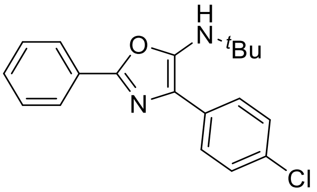

The title compound was prepared according to general procedure **E**, from **1d** (90 mg, 0.33 mmol), $Ca(NTf_2)_2$ (10 mg, 0.016 mmol), nBu_4NPF_6 (6 mg, 0.016 mmol) and tert-butyl isocyanide (33 mg, 0.39 mmol) in EtOAc (2 mL).

Following conversion to the product (15 min) and flash column chromatography (1:5 EtOAc/Hex, 1% NEt_3) the pure product was obtained as a pale yellow solid (95 mg, 89%)

RF (1:5 EtOAc/Hex, 1% NEt_3) = 0.38

IR ν_{max} (cm^{-1}): 3251, 3055, 2970, 2867, 1634, 1492

HRMS (ESI) m/z: $[M + H]^+$ Calcd for $C_{19}H_{20}ClN_2O$ 327.1264; Found 327.1259

^1H NMR (400 MHz, DMSO-d_6): δ 7.99 (d, *J* = 8.7 Hz, 2H), 7.95 – 7.90 (m, 2H), 7.56 – 7.43 (m, 5H), 5.56 (s, 1H), 1.30 (s, 9H).

^{13}C NMR (101 MHz, DMSO-d_6): δ 153.9, 149.0, 131.3, 130.5, 129.9, 129.2, 128.4, 127.2, 126.9, 125.1, 123.1, 53.3, 30.0.

_N_-tert-butyl-4-(4-nitrophenyl)-2-phenyl-1,3-oxazol-5-amine (2e)

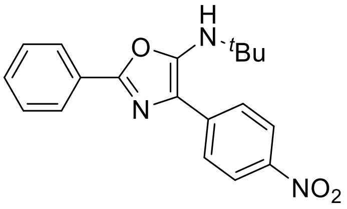

The title compound was prepared according to general procedure **E**, from **1e** (100 mg, 0.35 mmol), $Ca(NTf_2)_2$ (11 mg, 0.018 mmol), nBu_4NPF_6 (7 mg, 0.018 mmol) and tert-butyl isocyanide (35 mg, 0.42 mmol) in EtOAc (2 mL).

Following conversion to the product (1h 15 min) and flash column chromatography (1:9 EtOAc/Hex, 1% NEt_3) the pure product was obtained as a red solid (84 mg, 71%)

RF (1:9 EtOAc/Hex, 1% NEt_3) = 0.18

IR ν_{max} (cm^{-1}): 3395, 2980, 2909, 1590, 1321, 1216

HRMS (ESI) m/z: $[M + H]^+$ Calcd for $C_{19}H_{20}N_3O_3$ 338.1505; Found 338.1505

^1H NMR (400 MHz, DMSO-d_6): δ 8.25 (d, *J* = 9.0 Hz, 2H), 8.07 (d, *J* = 9.1 Hz, 2H), 7.93 (dd, *J* = 8.2, 1.3 Hz, 2H), 7.59 – 7.45 (m, 3H), 6.40 (s, 1H), 1.41 (s, 9H).

^{13}C NMR (101 MHz, DMSO-d_6): δ 152.8, 152.0, 144.1, 139. 6, 129.9, 129.3, 126.8, 125.0, 124.9, 123.9, 118.0, 53.4, 29.9.

4-[5-(_tert_-butylamino)-2-phenyl-1,3-oxazol-4-yl]benzonitrile (2f)

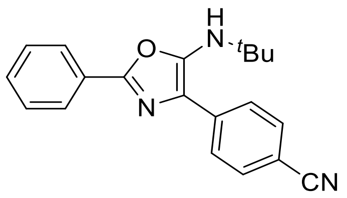

The title compound was prepared according to general procedure **E**, from **1f** (100 mg, 0.34 mmol), $Ca(NTf_2)_2$ (10 mg, 0.017 mmol), nBu_4NPF_6 (7 mg, 0.017 mmol) and tert-butyl isocyanide (34 mg, 0.41 mmol) in EtOAc (2 mL).

Following conversion to the product (30 min) and flash column chromatography (1:5 EtOAc/Hex, 1% NEt_3) the pure product was obtained as a white solid (78 mg, 72%)

RF (1:5 EtOAc/Hex, 1% NEt_3) = 0.16

IR ν_{max} (cm^{-1}): 3261, 3060, 2973, 2867, 2230, 1628

HRMS (ESI) m/z: $[M – CH_3]^+$ Calcd for $C_{19}H_{16}N_3O$ 302.1293; Found 302.1227

^1H NMR (400 MHz, DMSO-d_6): δ 8.05 (d, J = 8.4 Hz, 1H), 7.92 (d, J = 7.2 Hz, 1H), 7.84 (d, J = 8.4 Hz, 1H), 7.58 – 7.45 (m, 2H), 6.15 (s, 1H), 1.37 (s, 4H).

^{13}C NMR (101 MHz, DMSO-d_6): δ 153.0, 151.1, 137.3, 132.3, 129.9, 129.2, 126.9, 125.1, 125.0, 119.4, 119.3, 107.3, 53.3, 29.9.

3-[5-(*tert*-butylamino)-2-phenyl-1,3-oxazol-4-yl]benzonitrile (2g)

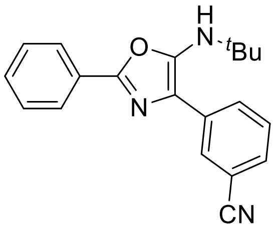

The title compound was prepared according to general procedure **E**, from **1g** (100 mg, 0.38 mmol), $Ca(NTf_2)_2$ (11 mg, 0.019 mmol), nBu_4NPF_6 (7 mg, 0.019 mmol) and tert-butyl isocyanide (38 mg, 0.45 mmol) in EtOAc (2 mL).

Following conversion to the product (20 min) and flash column chromatography (1:9 EtOAc/Hex, 1% NEt_3) the pure product was obtained as a yellow solid (107 mg, 90%)

RF (1:9 EtOAc/Hex, 1% NEt_3) = 0.15

IR ν_{max} (cm^{-1}): 3260, 3067, 2972, 2233, 1636, 1364

HRMS (ESI) m/z: $[M + H]^+$ Calcd for $C_{20}H_{20}N_3O$ 318.1606; Found 318.1607

^1H NMR (400 MHz, DMSO-d_6): δ 8.28 (s, 1H), 8.24 (d, J = 7.8 Hz, 1H), 7.94 (dd, J = 8.1, 1.2 Hz, 2H), 7.69 – 7.59 (m, 2H), 7.58 – 7.45 (m, 3H), 5.92 (s, 1H), 1.34 (s, 9H).

^{13}C NMR (101 MHz, DMSO-d_6): δ 153.5, 149.9, 133.7, 129.9, 129.7, 129.4, 129.3, 129.2, 128.0, 127.0, 125.1, 120.5, 119.1, 111.5, 53.3, 30.0.

N-tert-butyl-4-(2-methoxyphenyl)-2-phenyl-1,3-oxazol-5-amine (2h)

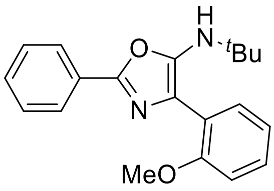

The title compound was prepared according to general procedure **E**, from **1h** (80 mg, 0.30 mmol), $Ca(NTf_2)_2$ (9 mg, 0.015 mmol), nBu_4NPF_6 (6 mg, 0.015 mmol) and tert-butyl isocyanide (29 mg, 0.35 mmol) in EtOAc (1.5 mL).

Following conversion to the product (1h 30 min) and flash column chromatography (1:9 EtOAc/Hex, 1% NEt_3) the pure product was obtained as a yellow oil (86 mg, 90%)

RF (1:9 EtOAc/Hex, 1% NEt_3) = 0.19

IR ν_{max} (cm^{-1}): 3354, 2963, 1597, 1453, 1233, 750

HRMS (ESI) m/z: $[M + H]^+$ Calcd for $C_{20}H_{23}N_2O_2$ 323.1760; Found 323.1760

^1H NMR (400 MHz, DMSO-d_6): δ 7.87 (d, J = 7.2 Hz, 2H), 7.67 (dd, J = 7.7, 1.8 Hz, 1H), 7.54 – 7.47 (m, 2H), 7.45 – 7.39 (m, 1H), 7.31 – 7.24 (m, 1H), 7.12 – 7.07 (m, 1H), 7.04 (td, J = 7.5, 1.0 Hz, 1H), 5.17 (s, 1H), 3.89 (s, 3H), 1.31 (s, 9H).

^{13}C NMR (101 MHz, DMSO-d_6): δ 154.5, 152.4, 150.4, 129.2, 129.1, 127.8, 127.4, 124.6, 121.6, 121.0, 115.9, 111.9, 55.5, 52.7, 30.0.

N-tert-butyl-4-(3-methylphenyl)-2-phenyl-1,3-oxazol-5-amine (2i)

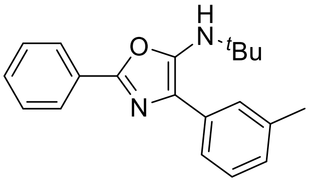

The title compound was prepared according to general procedure **E**, from **1i** (100 mg, 0.35 mmol), $Ca(NTf_2)_2$ (11 mg, 0.018 mmol), nBu_4NPF_6 (7 mg, 0.018 mmol) and tert-butyl isocyanide (35 mg, 0.42 mmol) in EtOAc (2 mL).

Following conversion to the product (20 min) and flash column chromatography (1:5 EtOAc/Hex, 1% NEt_3) the pure product was obtained as a yellow oil which solidifies on standing (101 mg, 93%)

RF (1:5 EtOAc/Hex, 1% NEt_3) = 0.42

^1H NMR (400 MHz, DMSO-d$_6$): δ 7.97 – 7.90 (m, 2H), 7.84 – 7.77 (m, 2H), 7.56 – 7.45 (m, 3H), 7.29 (t, *J* = 7.6 Hz, 1H), 7.05 (d, *J* = 7.5 Hz, 1H), 5.41 (s, 1H), 2.35 (s, 3H), 1.29 (s, 9H).

^{13}C NMR (101 MHz, DMSO-d$_6$): δ 153.8, 148.6, 137.3, 132.3, 129.7, 129.2, 128.2, 127.4, 127.0, 125.9, 125.1, 124.6, 122.5, 53.3, 30.1, 21.3.

*data in accordance with literature[5]

N-tert-butyl-4-(4-methoxyphenyl)-2-phenyl-1,3-oxazol-5-amine (2j)

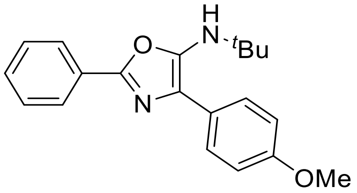

The title compound was prepared according to general procedure **E**, from **1j** (50 mg, 0.18 mmol), Ca(NTf$_2$)$_2$ (6 mg, 0.009 mmol), nBu$_4$NPF$_6$ (4 mg, 0.009 mmol) and tert-butyl isocyanide (18mg, 0.22 mmol) in EtOAc (1 mL).

Following conversion to the product (3h) and flash column chromatography (1:9 EtOAc/Hex, 1% NEt$_3$) the pure product was obtained as a white solid (24 mg, 40%)

RF (1:9 EtOAc/Hex, 1% NEt$_3$) = 0.17

^1H NMR (400 MHz, DMSO-d$_6$): δ 7.96 (d, *J* = 8.9 Hz, 2H), 7.95 – 7.91 (m, 2H), 7.57 – 7.44 (m, 3H), 6.98 (d, *J* = 8.8 Hz, 2H), 5.19 (s, 1H), 3.78 (s, 3H), 1.25 (s, 9H).

^{13}C NMR (101 MHz, DMSO-d$_6$): δ 158.0, 154.3, 147.3, 129.8, 129.2, 127.5, 126.8, 126.1, 125.1, 124.8, 113.7, 55.1, 53.3, 30.1.

*data in accordance with literature[5]

N-tert-butyl-2-phenyl-4-(thiophen-2-yl)-1,3-oxazol-5-amine (2k)

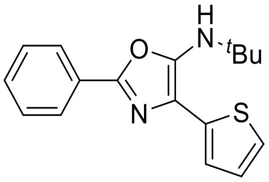

The title compound was prepared according to general procedure **E**, from **1k** (73 mg, 0.27 mmol), Ca(NTf$_2$)$_2$ (8 mg, 0.013 mmol), nBu$_4$NPF$_6$ (5 mg, 0.013 mmol) and tert-butyl isocyanide (26 mg, 0.32 mmol) in EtOAc (1 mL).

Following conversion to the product (1h 30 min) and flash column chromatography (1:9 EtOAc/Hex, 1% NEt$_3$) the pure product was obtained as a brown oil which solidified upon standing (46 mg, 58%)

RF (1:9 EtOAc/Hex, 1% NEt$_3$) = 0.40

IR ν_{max} (cm^{-1}): 3256, 2968, 1648, 1341, 1202, 830

HRMS (ESI) m/z: $[M + H]^+$ Calcd for $C_{17}H_{19}N_2OS$ 299.1218; Found 299.1215

^{1}H NMR (400 MHz, DMSO-d_6): δ ^{1}H NMR (400 MHz,) δ 7.91 (d, *J* = 6.8 Hz, 2H), 7.56 – 7.46 (m, 4H), 7.43 (dd, *J* = 5.1, 1.0 Hz, 1H), 7.10 (dd, *J* = 5.1, 3.6 Hz, 1H), 5.34 (s, 1H), 1.30 (s, 9H).

^{13}C NMR (101 MHz, DMSO-d_6): δ 154.1, 147.2, 134.9, 129.9, 129.2, 127.6, 127.1, 125.2, 124.1, 122.7, 121.9, 53.3, 30.0.

_N_-tert-butyl-4-(naphthalen-2-yl)-2-phenyl-1,3-oxazol-5-amine (2l)

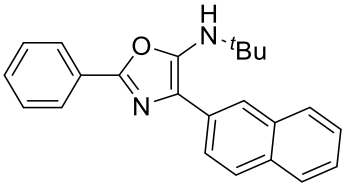

The title compound was prepared according to general procedure **E**, from **1l** (100 mg, 0.31 mmol), $Ca(NTf_2)_2$ (9 mg, 0.016 mmol), nBu_4NPF_6 (6 mg, 0.016 mmol) and tert-butyl isocyanide (31 mg, 0.38 mmol) in EtOAc (2 mL).

Following conversion to the product (15 min) and flash column chromatography (1:5 EtOAc/Hex, 1% NEt_3) the pure product was obtained as a pale yellow solid (96 mg, 90%)

RF (1:5 EtOAc/Hex, 1% NEt_3) = 0.40

^{1}H NMR (400 MHz, DMSO-d_6): δ 8.45 (s, 1H), 8.23 (dd, *J* = 8.7, 1.6 Hz, 1H), 8.01 – 7.92 (m, 4H), 7.89 (d, *J* = 7.8 Hz, 1H), 7.59 – 7.44 (m, 5H), 5.63 (s, 1H), 1.34 (s, 9H).

^{13}C NMR (101 MHz, DMSO-d_6): δ 154.0, 149.2, 133.2, 131.7, 130.0, 129.8, 129.2, 127.9, 127.7, 127.6, 127.4, 126.3, 125.6, 125.2, 124.2, 124.0, 123.4, 53.4, 30.1.

*data in accordance with literature[5]

Amide Variation

2-(4-bromophenyl)-_N_-tert-butyl-4-phenyl-1,3-oxazol-5-amine (3a)

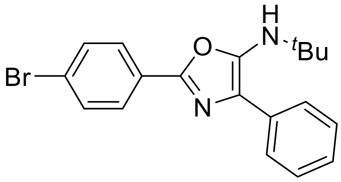

The title compound was prepared according to general procedure **E**, from **1m** (100 mg, 0.29 mmol), $Ca(NTf_2)_2$ (9 mg, 0.014 mmol), nBu_4NPF_6 (6 mg, 0.014 mmol) and tert-butyl isocyanide (29 mg, 0.35 mmol) in EtOAc (2 mL).

Following conversion to the product (15 min) and flash column chromatography (1:9 EtOAc/Hex, 1% NEt_3) the pure product was obtained as a pale-yellow solid (100 mg, 94%)

RF (1:9 EtOAc/Hex, 1% NEt_3) = 0.41

IR ν_{max} (cm^{-1}): 3262, 3057, 2968, 2927, 1631, 1469

HRMS (ESI) m/z: $[M + H]^+$ Calcd for $C_{19}H_{20}BrN_2O$ 371.0759; Found 371.0755

^{1}H NMR (400 MHz, DMSO-d_6): δ 7.97 (d, *J* = 7.3 Hz, 2H), 7.85 (d, *J* = 8.6 Hz, 2H), 7.73 (d, *J* = 8.6 Hz, 2H), 7.41 (t, *J* = 7.8 Hz, 2H), 7.26 – 7.20 (m, 1H), 5.53 (s, 1H), 1.30 (s, 9H).

^{13}C NMR (101 MHz, DMSO-d_6): δ 152.9, 149.0, 132.2, 128.4, 127.0, 126.5, 126.3, 125.3, 124.1, 123.0, 53.2, 30.1

***N*-tert-butyl-2-(4-chlorophenyl)-4-phenyl-1,3-oxazol-5-amine (3b)**

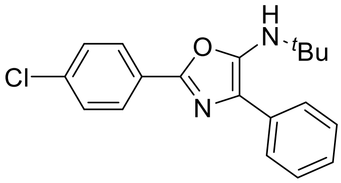

The title compound was prepared according to general procedure **E**, from **1n** (100 mg, 0.33 mmol), $Ca(NTf_2)_2$ (10 mg, 0.017 mmol), nBu_4NPF_6 (6 mg, 0.017 mmol) and tert-butyl isocyanide (33 mg, 0.40 mmol) in EtOAc (2 mL).

Following conversion to the product (10 min) and flash column chromatography (1:9 EtOAc/Hex, 1% NEt_3) the pure product was obtained as a white solid (98 mg, 91%)

RF (1:9 EtOAc/Hex, 1% NEt_3) = 0.45

IR ν_{max} (cm^{-1}): 3262, 3058, 2969, 1633, 1392, 1071

HRMS (ESI) m/z: $[M + H]^+$ Calcd for $C_{19}H_{20}ClN_2O$ 327.1264; Found 327.1252

^{1}H NMR (400 MHz, DMSO-d_6): δ 7.97 (d, *J* = 7.1 Hz, 2H), 7.92 (d, *J* = 8.6 Hz, 2H), 7.59 (d, *J* = 8.6 Hz, 2H), 7.41 (t, *J* = 7.8 Hz, 2H), 7.27 – 7.20 (m, 1H), 5.52 (s, 1H), 1.30 (s, 9H).

^{13}C NMR (101 MHz, DMSO-d_6): δ 152.8, 149.0, 134.3, 132.3, 129.3, 128.4, 126.8, 126.3, 126.2, 125.3, 124.1, 53.2, 30.1.

***N*-tert-butyl-4-phenyl-2-[4-(trifluoromethyl)phenyl]-1,3-oxazol-5-amine (3c)**

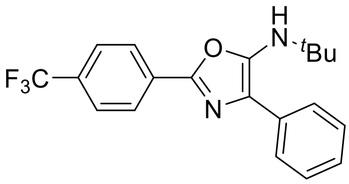

The title compound was prepared according to general procedure **E**, from **1o** (100 mg, 0.30 mmol), $Ca(NTf_2)_2$ (9 mg, 0.015 mmol), nBu_4NPF_6 (6 mg, 0.015 mmol) and tert-butyl isocyanide (30 mg, 0.36 mmol) in EtOAc (2 mL).

Following conversion to the product (40 min) and flash column chromatography (1:9 EtOAc/Hex, 1% NEt_3) the pure product was obtained as a yellow solid (91 mg, 85%)

RF (1:9 EtOAc/Hex, 1% NEt_3) = 0.43

IR ν_{max} (cm^{-1}): 3312, 2971, 2920, 1618, 1322, 1124

HRMS (ESI) m/z: $[M + H]^+$ Calcd for $C_{20}H_{20}F_3N_2O$ 361.1528; Found 361.1525

^{1}H NMR (400 MHz, DMSO-d_6): δ 8.10 (d, *J* = 8.1 Hz, 2H), 7.96 (d, *J* = 7.2 Hz, 2H), 7.88 (d, *J* = 8.4 Hz, 2H), 7.42 (t, *J* = 7.8 Hz, 2H), 7.24 (t, *J* = 7.4 Hz, 1H), 5.71 (s, 1H), 1.33 (s, 9H).

^{13}C NMR (101 MHz, DMSO-d_6): δ 152.0, 149.7, 132.2, 130.8, 129.2 (q, *J* = 31.9 Hz), 128.4, 126.3, 126.2 (d, *J* = 3.4 Hz), 125.5, 125.3, 124.1 (q, *J* = 272.1 Hz).123.5, 53.3, 30.0.

^{19}F NMR (376 MHz, DMSO-d_6): 61.10

_N_-tert-butyl-2-(3,5-difluorophenyl)-4-phenyl-1,3-oxazol-5-amine (3d)

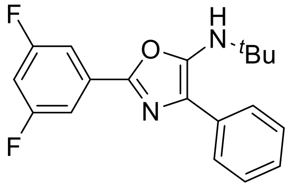

The title compound was prepared according to general procedure **E**, from **1p** (100 mg, 0.33 mmol), $Ca(NTf_2)_2$ (10 mg, 0.016 mmol), nBu_4NPF_6 (6 mg, 0.016 mmol) and tert-butyl isocyanide (33 mg, 0.39 mmol) in EtOAc (2 mL).

Following conversion to the product (45 min) and flash column chromatography (1:9 EtOAc/Hex, 1% NEt_3) the pure product was obtained as an orange solid (98 mg, 91%)

RF (1:9 EtOAc/Hex, 1% NEt_3) = 0.43

IR ν_{max} (cm^{-1}): 3274, 3084, 2972, 1625, 1356, 1120

HRMS (ESI) m/z: $[M + H]^+$ Calcd for $C_{19}H_{19}F_2N_2O$ 329.1465; Found 329.1460

^{1}H NMR (400 MHz, DMSO-d_6): δ 7.95 (dd, *J* = 8.3, 1.1 Hz, 2H), 7.53 – 7.49 (m, 2H), 7.45 – 7.35 (m, 3H), 7.24 (t, *J* = 7.3 Hz, 1H), 5.71 (s, 1H), 1.32 (s, 9H).

^{13}C NMR (101 MHz, DMSO-d_6): δ 164.1 (d, *J* = 13.3 Hz), 161.6 (d, *J* = 12.9 Hz), 151.2 (t, *J* = 3.4 Hz), 149.7, 132.0, 130.3 (t, *J* = 10.6 Hz), 128.4, 126.4, 125.3, 123.5, 108.1 (d, *J* = 7.8 Hz), 107.9 (d, *J* = 7.6 Hz), 105.0 (t, *J* = 26.0 Hz), 53.3, 30.1.

^{19}F NMR (376 MHz, DMSO-d_6): 108.12

_N_-tert-butyl-2-(3-chlorophenyl)-4-phenyl-1,3-oxazol-5-amine (3e)

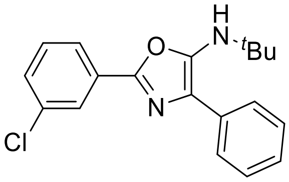

The title compound was prepared according to general procedure **E**, from **1q** (100 mg, 0.33 mmol), $Ca(NTf_2)_2$ (10 mg, 0.017 mmol), nBu_4NPF_6 (6 mg, 0.017 mmol) and tert-butyl isocyanide (33 mg, 0.40 mmol) in EtOAc (2 mL).

Following conversion to the product (1h) and flash column chromatography (1:9 EtOAc/Hex, 1% NEt_3) the pure product was obtained as a pale yellow solid (99 mg, 92%)

RF (1:9 EtOAc/Hex, 1% NEt_3) = 0.38

IR ν_{max} (cm^{-1}): 3269, 3066, 2974, 1627, 1492, 1203

HRMS (ESI) m/z: $[M + H]^+$ Calcd for $C_{19}H_{20}ClN_2O$ 327.1264; Found 327.1259

^1H NMR (400 MHz, DMSO-d_6): δ 7.97 (d, *J* = 7.3 Hz, 2H), 7.61 – 7.51 (m, 2H), 7.41 (t, *J* = 7.8 Hz, 2H), 7.24 (t, *J* = 7.4 Hz, 1H), 5.58 (s, 1H), 1.31 (s, 9H).

^{13}C NMR (101 MHz, DMSO-d_6): δ 152.2, 149.3, 133.9, 132.2, 131.3, 129.5, 129.2, 128.4, 126.4, 125.3, 124.4, 123.9, 123.6, 53.3, 30.1.

N-tert-butyl-2-(4-methoxyphenyl)-4-phenyl-1,3-oxazol-5-amine (3f)

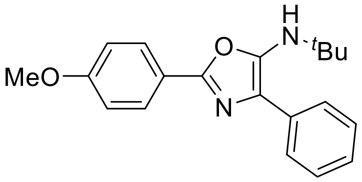

The title compound was prepared according to general procedure **E**, from **1r** (100 mg, 0.34 mmol), $Ca(NTf_2)_2$ (10 mg, 0.017 mmol), nBu_4NPF_6 (7 mg, 0.017 mmol) and tert-butyl isocyanide (34 mg, 0.41 mmol) in EtOAc (2 mL).

Following conversion to the product (30 min) and flash column chromatography (1:5 EtOAc/Hex, 1% NEt_3) the pure product was obtained as a white solid (105 mg, 97%)

RF (1:5 EtOAc/Hex, 1% NEt_3) = 0.19

IR ν_{max} (cm^{-1}): 3258, 3010, 2974, 2839, 1614, 1502

HRMS (ESI) m/z: $[M + H]^+$ Calcd for $C_{20}H_{23}N_2O_2$ 323.1760; Found 323.1759

^1H NMR (400 MHz, DMSO-d_6): δ 8.01 (d, *J* = 7.2 Hz, 2H), 7.87 (d, *J* = 8.9 Hz, 2H), 7.40 (t, *J* = 7.8 Hz, 2H), 7.23 (t, *J* = 7.4 Hz, 1H), 7.09 (d, *J* = 8.9 Hz, 2H), 5.27 (s, 1H), 3.82 (s, 3H), 1.27 (s, 9H).

^{13}C NMR (101 MHz, DMSO-d_6): δ 160.6, 154.4, 148.0, 132.5, 128.3, 126.9, 126.3, 125.3, 125.0, 120.2, 114.6, 55.4, 53. 2, 30.1.

_N_-tert-butyl-2-(furan-2-yl)-4-phenyl-1,3-oxazol-5-amine (3g)

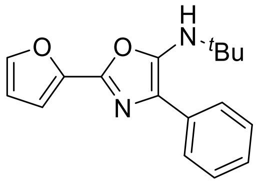

The title compound was prepared according to general procedure **E**, from **1s** (100 mg, 0.39 mmol), $Ca(NTf_2)_2$ (12 mg, 0.019 mmol), nBu_4NPF_6 (7 mg, 0.019 mmol) and tert-butyl isocyanide (39 mg, 0.46 mmol) in EtOAc (2 mL).

Following conversion to the product (20 min) and flash column chromatography (1:9 EtOAc/Hex, 1% NEt_3) the pure product was obtained as an off-white solid (71 mg, 65%)

RF (1:9 EtOAc/Hex, 1% NEt_3) = 0.26

IR ν_{max} (cm^{-1}): 3287, 3118, 2973, 1633, 1497, 1206

HRMS (ESI) m/z: $[M + H]^+$ Calcd for $C_{17}H_{19}N_2O_2$ 283.1447; Found 283.1447

^{1}H NMR (400 MHz, DMSO-d_6): δ 7.98 (d, *J* = 7.3 Hz, 2H), 7.93 – 7.88 (m, 1H), 7.40 (t, *J* = 7.7 Hz, 2H), 7.24 (t, *J* = 7.4 Hz, 1H), 7.05 – 7.03 (m, 1H), 6.70 (dd, *J* = 3.5, 1.8 Hz, 1H), 5.39 (s, 1H), 1.25 (s, 9H).

^{13}C NMR (101 MHz, DMSO-d_6): δ 148.0, 147.6, 144.9, 142.6, 132.1, 128.3, 126.5, 125.4, 124.7, 112.2, 110.4, 53.4, 30.0.

_N_-tert-butyl-4-phenyl-2-(thiophen-2-yl)-1,3-oxazol-5-amine (3h)

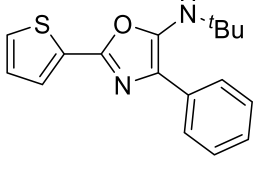

The title compound was prepared according to general procedure **E**, from **1t** (100 mg, 0.36 mmol), $Ca(NTf_2)_2$ (11 mg, 0.018 mmol), nBu_4NPF_6 (7 mg, 0.018 mmol) and tert-butyl isocyanide (36 mg, 0.44 mmol) in EtOAc (2 mL).

Following conversion to the product (2.5 h) and flash column chromatography (1:9 EtOAc/Hex, 1% NEt_3) the pure product was obtained as a yellow solid (96 mg, 86%)

RF (1:9 EtOAc/Hex, 1% NEt_3) = 0.37

IR v_{max} (cm^{-1}): 3268, 3104, 2974, 1633, 1498, 1364

HRMS (ESI) m/z: $[M + H]^+$ Calcd for $C_{17}H_{19}N_2OS$ 299.1218; Found 299.1218

^{1}H NMR (400 MHz, DMSO-d_6): δ 7.97 (d, J = 7.2 Hz, 2H), 7.72 (dd, J = 5.0, 1.0 Hz, 1H), 7.59 (dd, J = 3.6, 1.0 Hz, 1H), 7.40 (t, J = 7.7 Hz, 2H), 7.23 (t, J = 7.4 Hz, 1H), 7.20 (dd, J = 4.9, 3.7 Hz, 1H), 5.40 (s, 1H), 1.26 (s, 9H).

^{13}C NMR (101 MHz, DMSO-d_6): δ 150.7, 148.0, 132.1, 130. 0, 128.5, 128.4, 128.3, 126.6, 126.4, 125.4, 124.8, 53.3, 30.0.

_N_-tert-butyl-2-(2-methylphenyl)-4-phenyl-1,3-oxazol-5-amine (3i)

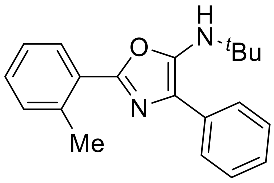

The title compound was prepared according to general procedure **E**, from **1u** (90 mg, 0.33 mmol), $Ca(NTf_2)_2$ (10 mg, 0.016 mmol), nBu_4NPF_6 (6 mg, 0.016 mmol) and tert-butyl isocyanide (32 mg, 0.44 mmol) in EtOAc (2 mL).

Following conversion to the product (20 min) and flash column chromatography (1:9 EtOAc/Hex, 1% NEt_3) the pure product was obtained as an off-white solid (95 mg, 95%)

RF (1:9 EtOAc/Hex, 1% NEt_3) = 0.42

IR v_{max} (cm^{-1}): 3327, 3065, 2969, 1601, 1447, 1365

HRMS (ESI) m/z: $[M + H]^+$ Calcd for $C_{20}H_{23}N_2O$ 307.1810; Found 307.1807

^{1}H NMR (400 MHz, DMSO-d_6): δ 8.00 (d, J = 7.3 Hz, 2H), 7.93 – 7.87 (m, 1H), 7.41 (t, J = 7.7 Hz, 2H), 7.38 – 7.31 (m, 3H), 7.23 (t, J = 7.4 Hz, 1H), 5.41 (s, 1H), 2.70 (s, 3H), 1.30 (s, 9H).

^{13}C NMR (101 MHz, DMSO-d_6): δ 154.3, 148.2, 136.0, 132.6, 131.7, 129.4, 128.3, 127.5, 126.3, 126.2, 126.2, 125.3, 124.0, 53.2, 30.1, 21.9.

Isocyanide Variation

2,4-diphenyl-_N_-[4-(trifluoromethyl)phenyl]-1,3-oxazol-5-amine (4a)

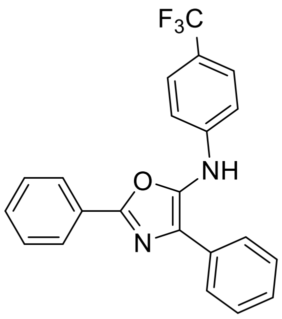

The title compound was prepared according to general procedure **E**, from **1a** (100 mg, 0.41 mmol), $Ca(NTf_2)_2$ (12 mg, 0.021 mmol), nBu_4NPF_6 (8 mg, 0.021 mmol) and 1-isocyano-4-(trifluoromethyl)benzene (85 mg, 0.50 mmol) in EtOAc (2 mL).

Following conversion to the product (20 min) and flash column chromatography (1:20 EtOAc/Hex, 1% NEt_3) the pure product was obtained as an orange oil (129 mg, 82%)

RF (1:5 EtOAc/Hex, 1% NEt_3) = 0.48

IR ν_{max} (cm^{-1}): 3369, 3061, 2973, 1613, 1320, 830

HRMS (ESI) m/z: $[M + H]^+$ Calcd for $C_{22}H_{16}F_3N_2O$ 381.1215; Found 381.1215

^1H NMR (400 MHz, DMSO-d_6): δ 9.30 (s, 1H), 8.09 – 7.96 (m, 2H), 7.87 (d, *J* = 7.3 Hz, 2H), 7.62 – 7.52 (m, 5H), 7.44 (t, *J* = 7.7 Hz, 2H), 7.32 (t, *J* = 7.4 Hz, 1H), 6.96 (d, *J* = 8.6 Hz, 2H).

^{13}C NMR (101 MHz, DMSO-d_6): δ 157.2, 148.3, 141.8, 131.2, 131.2, 130.1, 129.7, 129.3, 128.3, 127.3, 126.3, 126.2, 125.3 (q, *J* = 270.8 Hz), 120.2 (q, *J* = 32.3 Hz), 114.5, 46.2.

^{19}F NMR (376 MHz, DMSO-d_6): 59.56

4-[(2,4-diphenyl-1,3-oxazol-5-yl)amino]benzonitrile (4b)

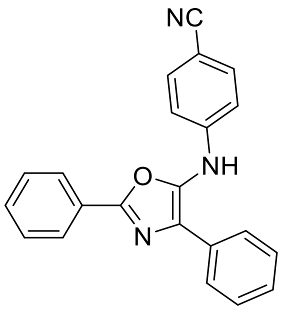

The title compound was prepared according to general procedure **E**, from **1a** (100 mg, 0.41 mmol), $Ca(NTf_2)_2$ (12 mg, 0.021 mmol), nBu_4NPF_6 (8 mg, 0.021 mmol) and 4-isocyanobenzonitrile (64 mg, 0.50 mmol) in EtOAc (2 mL).

Following conversion to the product (30 min) and flash column chromatography (10 to 20% EtOAc/Hex, 1% NEt_3) the pure product was obtained as an off-white solid (120 mg, 86%)

RF (1:5 EtOAc/Hex, 1% NEt_3) = 0.27

IR ν_{max} (cm^{-1}): 3287, 3060, 2219, 1601, 1512, 1328, 1173, 690

HRMS (ESI) m/z: $[M - H]^+$ Calcd for $C_{22}H_{14}N_3O$ 336.1137; Found 336.1149

^1H NMR (400 MHz, DMSO-d_6): δ 9.49 (s, 1H), 8.06 – 7.98 (m, 2H), 7.84 (d, *J* = 7.3 Hz, 2H), 7.64 (d, *J* = 8.8 Hz, 2H), 7.59 – 7.52 (m, 3H), 7.44 (t, *J* = 7.7 Hz, 2H), 7.33 (t, *J* = 7.4 Hz, 1H), 6.93 (d, *J* = 8.7 Hz, 2H).

^{13}C NMR (101 MHz, DMSO-d_6): δ 156.9, 148.5, 140.7, 134.0, 130.8, 130.5, 129.7, 129.2, 128.8, 127.9, 126.8, 125.9, 125.7, 119.7, 114.5, 100.9.

***N*-(4-bromophenyl)-2,4-diphenyl-1,3-oxazol-5-amine (4c)**

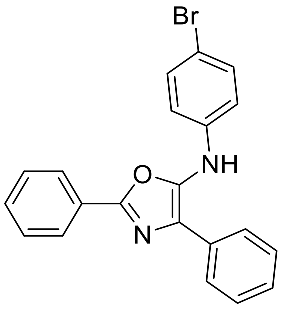

The title compound was prepared according to general procedure **E**, from **1a** (60 mg, 0.24 mmol), $Ca(NTf_2)_2$ (7 mg, 0.012 mmol), nBu_4NPF_6 (5 mg, 0.012 mmol) and 1-bromo-4-isocyanobenzene (53 mg, 0.29 mmol) in EtOAc (1 mL).

Following conversion to the product (45 min) and flash column chromatography (1:9 EtOAc/Hex, 1% NEt_3) the pure product was obtained as an orange oil (77 mg, 82%)

RF (1:9 EtOAc/Hex, 1% NEt_3) = 0.42

^1H NMR (400 MHz, DMSO-d_6): δ 8.94 (s, 1H), 8.03 – 7.98 (m, 2H), 7.86 (d, *J* = 7.3 Hz, 2H), 7.56 – 7.53 (m, 3H), 7.43 (t, *J* = 7.7 Hz, 2H), 7.37 (d, *J* = 8.8 Hz, 2H), 7.31 (t, *J* = 7.4 Hz, 1H), 6.78 (d, *J* = 8.9 Hz, 2H).

^{13}C NMR (101 MHz, DMSO-d_6): δ 156.3, 143.6, 142.2, 132.1, 130.8, 130.6, 129.2, 128.9, 128.7, 127.6, 126.9, 125.7, 125.6, 116.2, 110.7.

*data in accordance with literature[5]

***N*-(3-methoxyphenyl)-2,4-diphenyl-1,3-oxazol-5-amine (4d)**

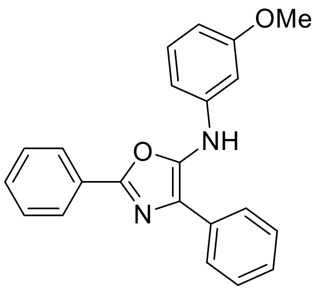

The title compound was prepared according to general procedure **E**, from **1a** (100 mg, 0.41 mmol), $Ca(NTf_2)_2$ (12 mg, 0.021 mmol), nBu_4NPF_6 (8 mg, 0.021 mmol) and 1-isocyano-3-methoxybenzene (64 mg, 0.50 mmol) in EtOAc (2 mL).

Following conversion to the product (15 min) and flash column chromatography (1:9 EtOAc/Hex, 1% NEt_3) the pure product was obtained as an orange oil (72 mg, 51%)

RF (1:5 EtOAc/Hex, 1% NEt_3) = 0.37

IR ν_{max} (cm^{-1}): 3298, 2922, 1595, 1331, 1202, 840

HRMS (ESI) m/z: $[M + H]^+$ Calcd for $C_{22}H_{19}N_2O_2$ 343.1447; Found 343.1440

^1H NMR (400 MHz, DMSO-d_6): δ 8.76 (s, 1H), 8.03 – 7.97 (m, 2H), 7.88 (d, *J* = 7.3 Hz, 2H), 7.59 – 7.50 (m, 3H), 7.43 (t, *J* = 7.7 Hz, 2H), 7.30 (t, *J* = 7.4 Hz, 1H), 7.11 (t, *J* = 8.1 Hz, 1H), 6.46 – 6.28 (m, 1H), 3.66 (s, 3H).

^{13}C NMR (101 MHz, DMSO-d_6): δ 160.4, 156.2, 145.5, 142.6, 131.0, 130.6, 130.3, 129.2, 128.7, 128.6, 127.5, 127.0, 125.7, 106.8, 105.0, 100.2, 54.9.

***N*-(2-chloro-4-methylphenyl)-2,4-diphenyl-1,3-oxazol-5-amine (4e)**

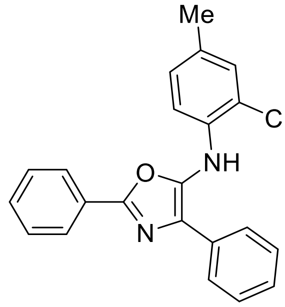

The title compound was prepared according to general procedure **E**, from **1a** (100 mg, 0.41 mmol), $Ca(NTf_2)_2$ (12 mg, 0.021 mmol), nBu_4NPF_6 (8 mg, 0.021 mmol) and 2-chloro-1-isocyano-4-methylbenzene (75 mg, 0.50 mmol) in EtOAc (2 mL).

Following conversion to the product (15 min) and flash column chromatography (1:9 EtOAc/Hex, 1% NEt_3) the pure product was obtained as an orange oil (139 mg, 93%)

RF (1:5 EtOAc/Hex, 1% NEt_3) = 0.56

IR ν_{max} (cm^{-1}): 3300, 2920, 1640, 1506, 1283, 810

HRMS (ESI) m/z: $[M + H]^+$ Calcd for $C_{22}H_{18}ClN_2O$ 361.1108; Found 361.1109

^{1}H NMR (400 MHz, DMSO-d_6): δ δ 8.25 (s, 1H), 8.02 (dd, *J* = 6.5, 3.0 Hz, 2H), 7.87 (d, *J* = 7.5 Hz, 2H), 7.59 – 7.51 (m, 3H), 7.42 (t, *J* = 7.7 Hz, 2H), 7.30 (t, *J* = 7.3 Hz, 1H), 7.25 (s, 1H), 6.92 (dd, *J* = 8.3, 1.2 Hz, 1H), 6.55 (d, *J* = 8.3 Hz, 1H), 2.19 (s, 3H).

^{13}C NMR (101 MHz, DMSO-d_6): δ 156.9, 142.2, 138.3, 130.8, 130.7, 130.5, 130.1, 130.0, 129.2, 128.8, 128.6, 127.7, 126.9, 125.8, 125.6, 119.0, 115.0, 19.7.

***N*-benzyl-2,4-diphenyl-1,3-oxazol-5-amine (4f)**

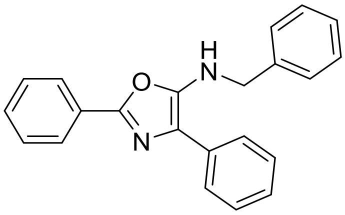

The title compound was prepared according to general procedure **E**, from **1a** (100 mg, 0.41 mmol), $Ca(NTf_2)_2$ (12 mg, 0.021 mmol), nBu_4NPF_6 (8 mg, 0.021 mmol) and benzyl isocyanide (58 mg, 0.50 mmol) in EtOAc (2 mL).

Following conversion to the product (1h 15 min) and flash column chromatography (1:20 EtOAc/Hex, 1% NEt_3) the pure product was obtained as a yellow oil (75 mg, 55%)

RF (1:9 EtOAc/Hex, 1% NEt_3) = 0.53

^{1}H NMR (400 MHz, DMSO-d_6): δ 7.82 (d, *J* = 7.2 Hz, 2H), 7.76 (d, *J* = 7.3 Hz, 2H), 7.50 – 7.43 (m, 4H), 7.43 – 7.36 (m, 3H), 7.34 (t, *J* = 7.6 Hz, 2H), 7.27 – 7.21 (m, 2H), 7.17 (t, *J* = 7.4 Hz, 1H), 4.53 (d, *J* = 6.0 Hz, 2H).

^{13}C NMR (101 MHz, DMSO-d_6): δ 150.6, 150.3, 140.2, 132.8, 129.0, 129.0, 128.5, 128.4, 127.5, 127.2, 127.0, 125.0, 124.5, 124.2, 113.9, 47.4.

*data in accordance with literature[5]

***N*-cyclohexyl-2,4-diphenyl-1,3-oxazol-5-amine (4g)**

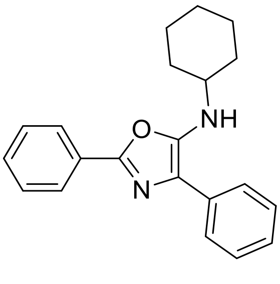

The title compound was prepared according to general procedure **E**, from **1a** (100 mg, 0.41 mmol), $Ca(NTf_2)_2$ (12 mg, 0.021 mmol), nBu_4NPF_6 (8 mg, 0.021 mmol) and cyclohexyl isocyanide (54 mg, 0.50 mmol) in EtOAc (2 mL).

Following conversion to the product (5h) and flash column chromatography (1:9 EtOAc/Hex, 1% NEt_3) the pure product was obtained as an orange oil (82 mg, 62%)

RF (1:9 EtOAc/Hex, 1% NEt_3) = 0.42

^{1}H NMR (400 MHz, DMSO-d_6): δ 7.89 (d, *J* = 7.2 Hz, 2H), 7.80 (d, *J* = 7.2 Hz, 2H), 7.50 (t, *J* = 7.4 Hz, 2H), 7.47 – 7.36 (m, 3H), 7.18 (t, *J* = 7.4 Hz, 1H), 6.07 (d, *J* = 7.2 Hz, 1H), 3.47 – 3.37 (m, 1H), 2.00 – 1.90 (m, 2H), 1.80 – 1.68 (m, 2H), 1.64 – 1.52 (m, 1H), 1.46 – 1.24 (m, 4H), 1.23 – 1.08 (m, 1H).

^{13}C NMR (101 MHz, DMSO-d_6): δ 151.2, 150.1, 132.8, 129.2, 129.1, 128.4, 127.4, 125.3, 124.7, 124.6, 116.5, 53.9, 33.4, 25.4, 24.8.

*data in accordance with literature[5]

6. Calcium catalyzed synthesis of 5-aminothiazoles

***N*-tert-butyl-2,4-diphenyl-1,3-thiazol-5-amine (6a)**

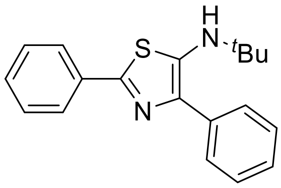

The title compound was prepared according to general procedure **F**, from **5a** (50 mg, 0.19 mmol), $Ca(NTf_2)_2$ (12 mg, 0.019 mmol), nBu_4NPF_6 (8 mg, 0.019 mmol) and tert-butyl isocyanide (19 mg, 0.23 mmol) in EtOAc (1 mL).

Following conversion to the product (30 min) and flash column chromatography (1:20 EtOAc/Hex, 1% NEt_3) the pure product was obtained as a yellow oil (50 mg, 83%)

RF (1:5 EtOAc/Hex) = 0.57

^{1}H NMR (400 MHz, DMSO-d_6): δ 8.10 (d, *J* = 7.1 Hz, 2H), 7.89 (d, *J* = 7.1 Hz, 2H), 7.53 – 7.36 (m, 5H), 7.28 (t, *J* = 7.4 Hz, 1H), 4.97 (s, 1H), 1.22 (s, 9H).

^{13}C NMR (101 MHz, DMSO-d_6): δ 155.9, 142.2, 142.1, 135.2, 133.7, 129.4, 129.1, 128.2, 127.6, 126.9, 125.3, 53.2, 28.9.

*data in accordance with literature[5]

4-(4-bromophenyl)-*N*-tert-butyl-2-phenyl-1,3-thiazol-5-amine (6b)

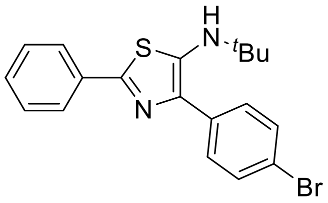

The title compound was prepared according to general procedure **F**, from **5b** (50 mg, 0.15 mmol), $Ca(NTf_2)_2$ (9 mg, 0.015 mmol), nBu_4NPF_6 (6 mg, 0.015 mmol) and tert-butyl isocyanide (15 mg, 0.18 mmol) in EtOAc (0.8 mL).

Following conversion to the product (15 min) and flash column chromatography (2% EtOAc/Hex, 1% NEt_3) the pure product was obtained as a yellow oil (44 mg, 76%)

RF (1:5 EtOAc/Hex) = 0.70

IR ν_{max} (cm^{-1}): 3326, 3063, 2968, 1520, 1479, 1384, 1200, 1069, 829

HRMS (ESI) m/z: $[M + H]^+$ Calcd for $C_{19}H_{20}BrN_2S$ 387.0531; Found 387.0532

^1H NMR (400 MHz, DMSO-d_6): δ 8.10 (d, *J* = 8.6 Hz, 2H), 7.93 – 7.87 (m, 2H), 7.61 (d, *J* = 8.6 Hz, 2H), 7.52 – 7.39 (m, 3H), 5.08 (s, 1H), 1.21 (s, 9H).

^{13}C NMR (101 MHz, DMSO-d_6): δ 156.4, 142.7, 141.2, 134.4, 133.6, 131.1, 129.6, 129.6, 129.2, 125.4, 119.7, 53.3, 28.8.

2-(4-bromophenyl)-*N*-tert-butyl-4-phenyl-1,3-thiazol-5-amine (6c)

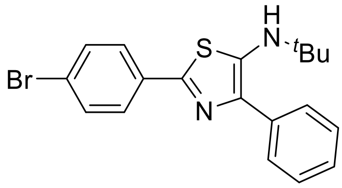

The title compound was prepared according to general procedure **F**, from **5c** (50 mg, 0.15 mmol), $Ca(NTf_2)_2$ (9 mg, 0.015 mmol), nBu_4NPF_6 (6 mg, 0.015 mmol) and tert-butyl isocyanide (15 mg, 0.18 mmol) in EtOAc (0.8 mL).

Following conversion to the product (15 min) and flash column chromatography (2% EtOAc/Hex, 1% NEt_3) the pure product was obtained as a yellow oil (51 mg, 89%)

RF (1:5 EtOAc/Hex) = 0.59

IR ν_{max} (cm^{-1}): 3373, 3057, 2968, 1523, 1487, 1384, 1202, 1069, 826

HRMS (ESI) m/z: $[M + H]^+$ Calcd for $C_{19}H_{20}BrN_2S$ 387.0531; Found 387.0539

^1H NMR (400 MHz, DMSO-d_6): δ 8.11 – 8.03 (m, 2H), 7.83 (d, *J* = 8.6 Hz, 2H), 7.66 (d, *J* = 8.6 Hz, 2H), 7.43 (t, *J* = 7.7 Hz, 2H), 7.29 (t, *J* = 7.3 Hz, 1H), 5.09 (s, 1H), 1.22 (s, 9H).

^{13}C NMR (101 MHz, DMSO-d_6): δ 154.2, 142.7, 141.9, 135.1, 132.9, 132.1, 128.3, 127.6, 127.1, 126.9, 122.4, 53.2, 28.9.

***N*-cyclohexyl-2,4-diphenyl-1,3-thiazol-5-amine (6d)**

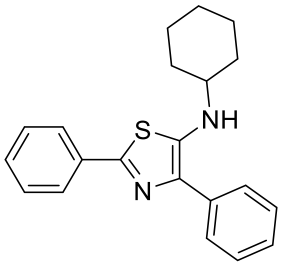

The title compound was prepared according to general procedure **F**, from **5a** (50 mg, 0.19 mmol), $Ca(NTf_2)_2$ (12 mg, 0.019 mmol), nBu_4NPF_6 (8 mg, 0.019 mmol) and cyclohexyl isocyanide (26 mg, 0.23 mmol) in EtOAc (1 mL).

Following conversion to the product (1 h) and flash column chromatography (2% EtOAc/Hex, 1% NEt_3) the pure product was obtained as a yellow oil (41 mg, 63%)

RF (1:5 EtOAc/Hex) = 0.46

^{1}H NMR (400 MHz, DMSO-d_6): δ 7.89 (d, *J* = 7.2 Hz, 2H), 7.81 (d, *J* = 7.2 Hz, 2H), 7.47 – 7.40 (m, 4H), 7.36 (t, *J* = 7.3 Hz, 1H), 7.25 (t, *J* = 7.4 Hz, 1H), 5.90 (d, *J* = 7.0 Hz, 1H), 3.03 – 2.90 (m, 1H), 2.10 – 1.93 (m, 2H), 1.80 – 1.67 (m, 2H), 1.61 – 1.53 (m, 1H), 1.44 – 1.08 (m, 5H).

^{13}C NMR (101 MHz, DMSO-d_6): δ 150.0, 146.9, 135.3, 133.9, 133.3, 129.1, 128.7, 128.4, 126.8, 126.1, 124.8, 59.1, 32.4, 25.4, 24.6.

*data in accordance with literature[5]

4-(4-bromophenyl)-*N*-cyclohexyl-2-phenyl-1,3-thiazol-5-amine (6e)

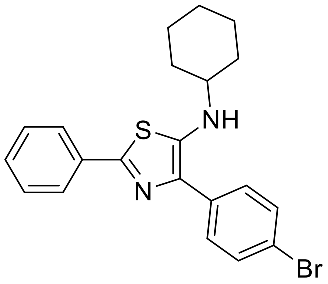

The title compound was prepared according to general procedure **F**, from **5b** (50 mg, 0.15 mmol), $Ca(NTf_2)_2$ (9 mg, 0.015 mmol), nBu_4NPF_6 (6 mg, 0.015 mmol) and cyclohexyl isocyanide (20 mg, 0.18 mmol) in EtOAc (0.8 mL).

Following conversion to the product (45 min) and flash column chromatography (2% EtOAc/Hex, 1% NEt_3) the pure product was obtained as an orange oil (37 mg, 60%)

RF (1:5 EtOAc/Hex) = 0.59

IR ν_{max} (cm^{-1}): 3276, 3060, 2927, 2952, 1590, 1481, 1347, 1068, 827

HRMS (ESI) m/z: $[M + H]^+$ Calcd for $C_{21}H_{22}BrN_2S$ 413.0687; Found 413.0681

^{1}H NMR (400 MHz, DMSO-d_6): δ 7.85 (d, *J* = 8.6 Hz, 2H), 7.83 – 7.78 (m, 2H), 7.60 (d, *J* = 8.6 Hz, 2H), 7.44 (t, *J* = 7.4 Hz, 2H), 7.37 (t, *J* = 7.3 Hz, 1H), 6.04 (d, *J* = 6.8

Hz, 1H), 3.03 – 2.90 (m, 1H), 2.06 – 1.97 (m, 2H), 1.77 – 1.69 (m, 2H), 1.65 – 1.51 (m, 1H), 1.41 – 1.14 (m, 5H).

^{13}C NMR (101 MHz, DMSO-d$_6$): δ 150.3, 147.6, 134.5, 133.7, 132.1, 131.3, 129.1, 128.8, 128.7, 124.9, 118.7, 59.2, 32.3, 25.3, 24.6.

2-(4-bromophenyl)-*N*-cyclohexyl-4-phenyl-1,3-thiazol-5-amine (6f)

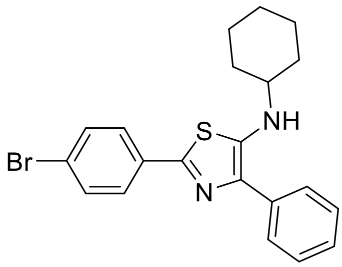

The title compound was prepared according to general procedure **F**, from **5c** (50 mg, 0.15 mmol), $Ca(NTf_2)_2$ (9 mg, 0.015 mmol), nBu_4NPF_6 (6 mg, 0.015 mmol) and cyclohexyl isocyanide (20 mg, 0.18 mmol) in EtOAc (0.8 mL).

Following conversion to the product (30 min) and column chromatography (2% EtOAc/Hex, 1% NEt_3) the pure product was obtained as a yellow oil (40 mg, 65%)

RF (1:5 EtOAc/Hex) = 0.68

IR ν_{max} (cm^{-1}): 3369, 2927, 2851, 1252, 1487, 1361, 1088, 971

HRMS (ES) m/z: [M + H]$^+$ Calcd for $C_{21}H_{22}BrN_2S$ 413.0687; Found 413.0698

^{1}H NMR (400 MHz, DMSO-d$_6$): δ 7.89 – 7.85 (m, 2H), 7.74 (d, *J* = 8.6 Hz, 2H), 7.61 (d, *J* = 8.6 Hz, 2H), 7.42 (t, *J* = 7.8 Hz, 2H), 7.25 (t, *J* = 7.4 Hz, 1H), 6.01 (d, *J* = 7.0 Hz, 1H), 3.01 – 2.90 (m, 1H), 2.05 – 1.91 (m, 2H), 1.80 – 1.66 (m, 2H), 1.62 – 1.51 (m, 1H), 1.45 – 1.08 (m, 5H).

^{13}C NMR (101 MHz, DMSO-d$_6$): δ 148.3, 147.5, 135.1, 133.2, 133.1, 132.0, 128.4, 126.8, 126.6, 126.2, 121.5, 59.2, 32.3, 25.3, 24.6.

7. Catalyst Turnover Studies

Procedure

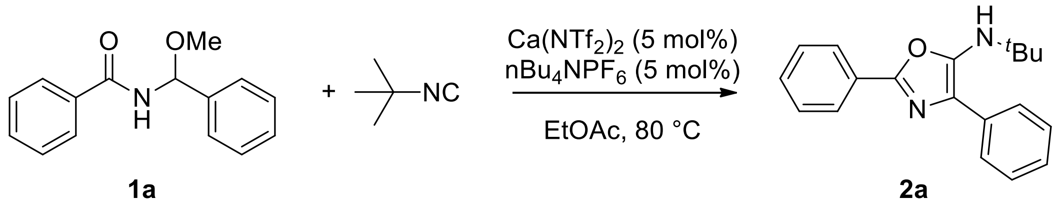

Study was carried out using **1a** (0.2 mmol), $Ca(NTf_2)_2$ (0.010 mmol), nBu_4NPF_6 (0.01 mmol) and *tert*-butylisocyanide (0.24 mmol) in EtOAc (1 mL). The reaction was stirred at 80 °C for 20 minutes. A 100 μL aliquot was taken, diluted in DMSO-d_6 (0.5 mL) using 1,3,5-trimethoxybenzne as the internal standard. Sequential additions every 20 minutes for 1h 40 min with the NMR yield determined prior to each addition.

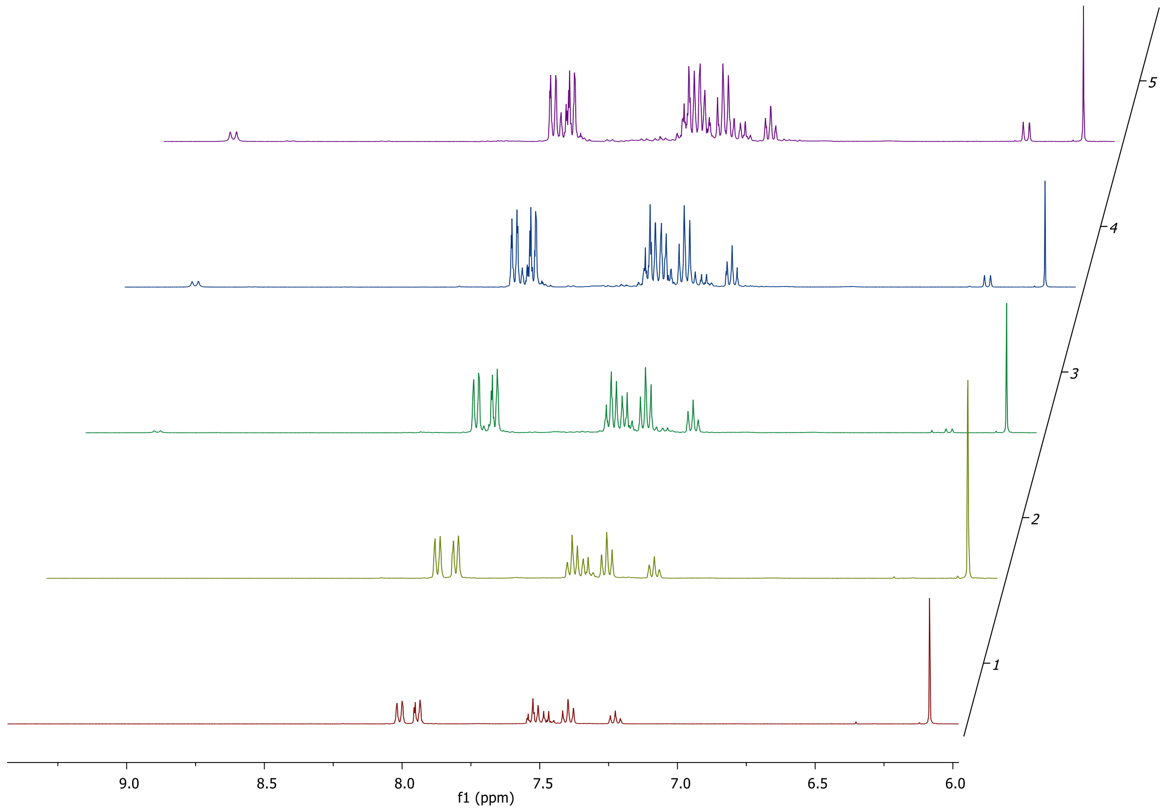

Stacked spectra (first run at bottom, run 5 at the top). Appearance of doublet at 9.18 ppm (N-H) and 6.30 ppm (C-H) denotes starting material indicating reaction slowing.

Isolated mass: 116 mg (over 5 runs), when mass used in NMR yields is added in this equates to 51% yield over 5 runs.

Something worth noting is an 82% yield after 3 runs.

Tables

Run	mmol of product in NMR sample	mmol product remaining	moles total
1	0.016053	0.16053	0.16053
2	0.034675	0.312075	0.328128
3	0.05538	0.44304	0.493768
4	0.058753	0.411271	0.517379
5	0.059005	0.35403	0.518891

mmol of product on the graph denotes total mmol scaled up to the amount in the reaction and the amounts removed in previous aliquots.

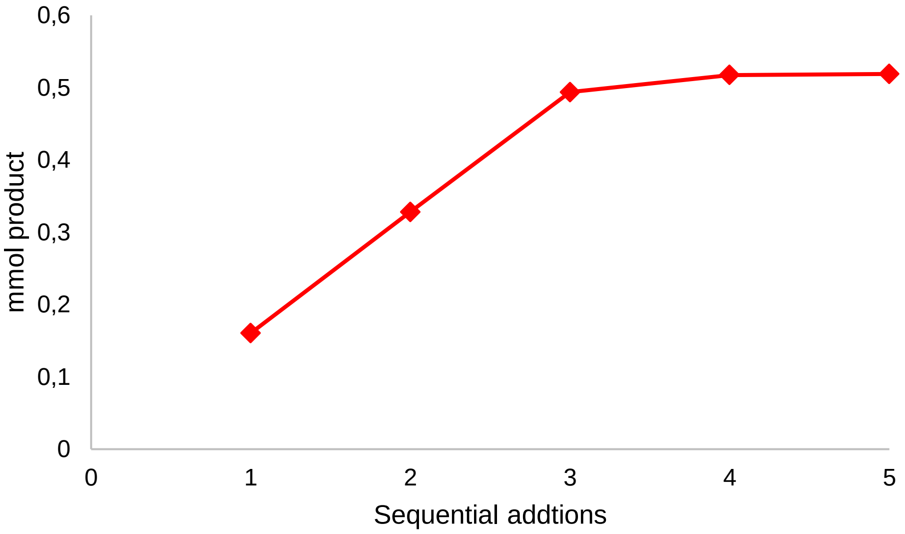

Figure 1. Reaction monitored by ^{1}H NMR using 1,3,5-trimethoxybenzene as an internal standard

Graph can also be plotted to show the decrease in overall yield as sequential additions are added

Run	Percentage yield / %
1	80.12779
2	81.89177
3	81.9206
4	64.56182
5	51.8891

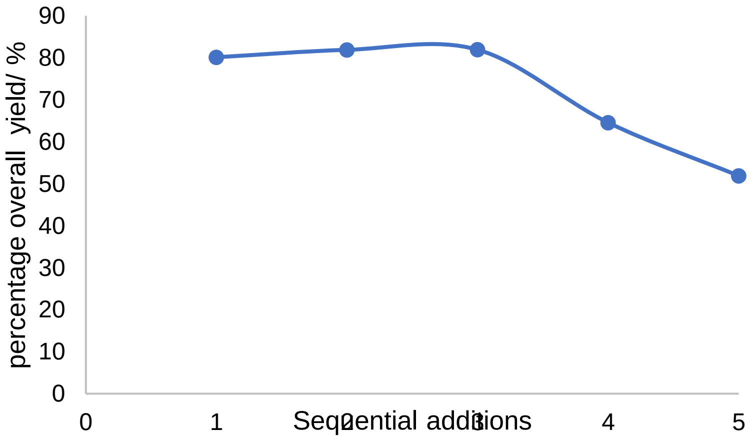

References

1. J. Halli, K. Hofman, T. Beisel and G. Manolikakes, *Eur. J. Org. Chem.*, 2015, **2015**, 4624-4627.
2. M. Li, B. Luo, Q. Liu, Y. Hu, A. Ganesan, P. Huang and S. Wen, *Org. Lett.*, 2014, **16**, 10-13.
3. A. R. Katritzky, J. Pernak, W. Q. Fan and F. Saczewski, *J. Org. Chem*, 1991, **56**, 4439-4443.
4. R. Erbea, S. Panossian and C. Giordano, *Synthesis*, 1977, **1977**, 250-252.
5. T. Soeta, K. Tamura and Y. Ukaji, *Tetrahedron*, 2014, **70**, 3005-3010.

8. COPIES OF SPECTRA

Figure S.1. *N*-(Methoxy(phenyl)methyl)benzamide (1a)

^{1}H NMR (400 MHz, DMSO-d_6)

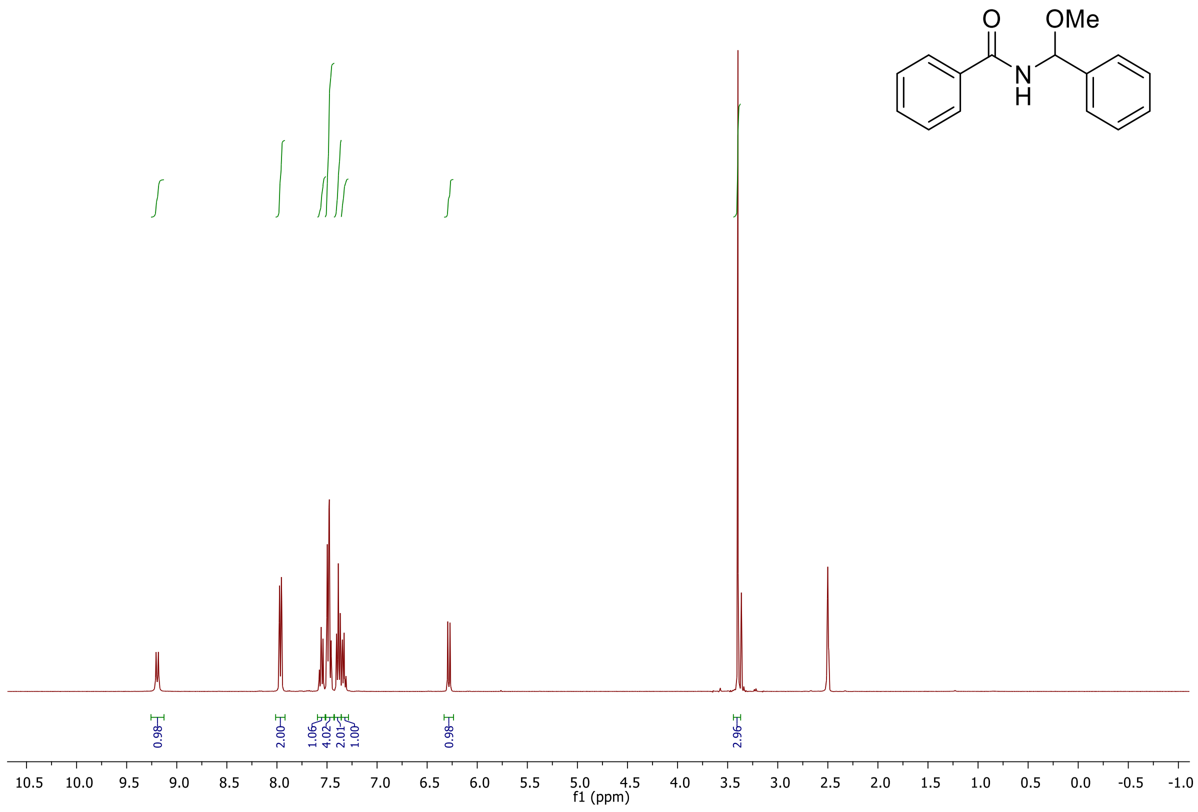

$^{13}C\{^{1}H\}$ NMR (101 MHz, DMSO-d_6)

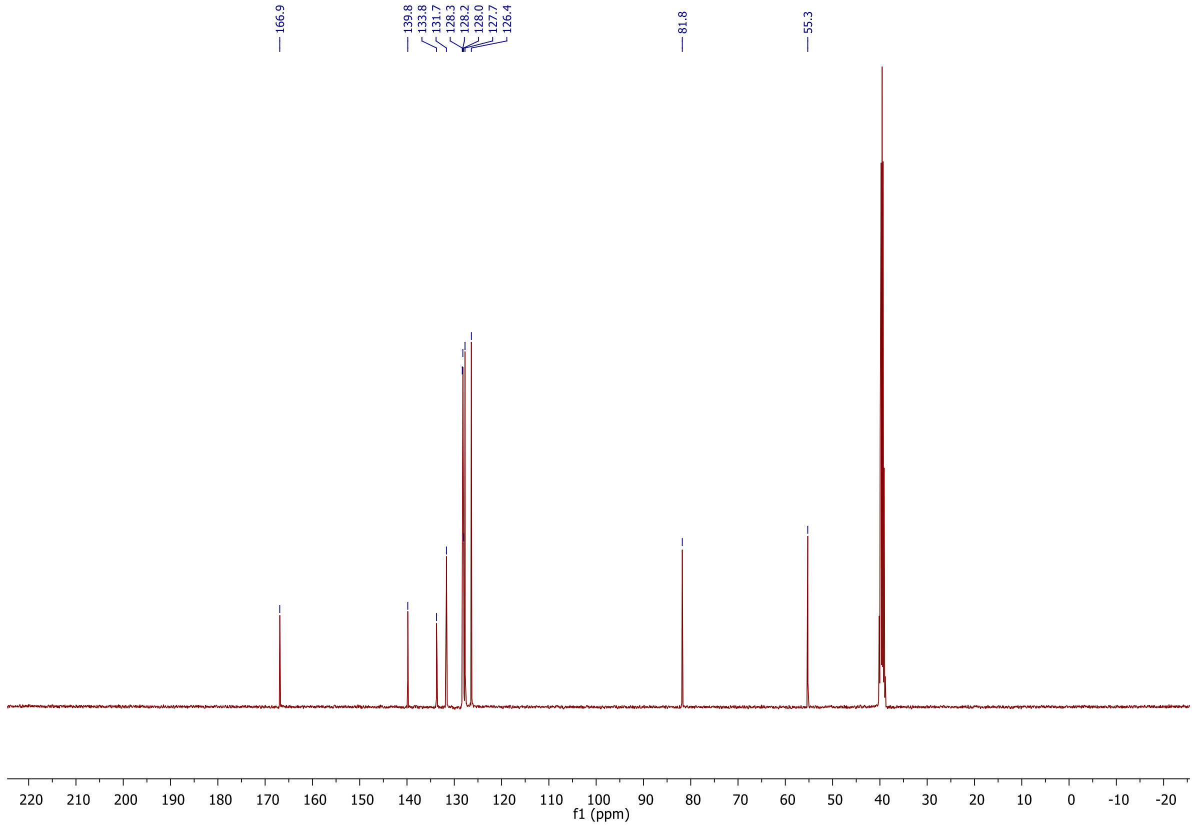

Figure S.2. ***N*-[(4-bromophenyl)(methoxy)methyl]benzamide (1b)**

^{1}H NMR (400 MHz, DMSO-d_6)

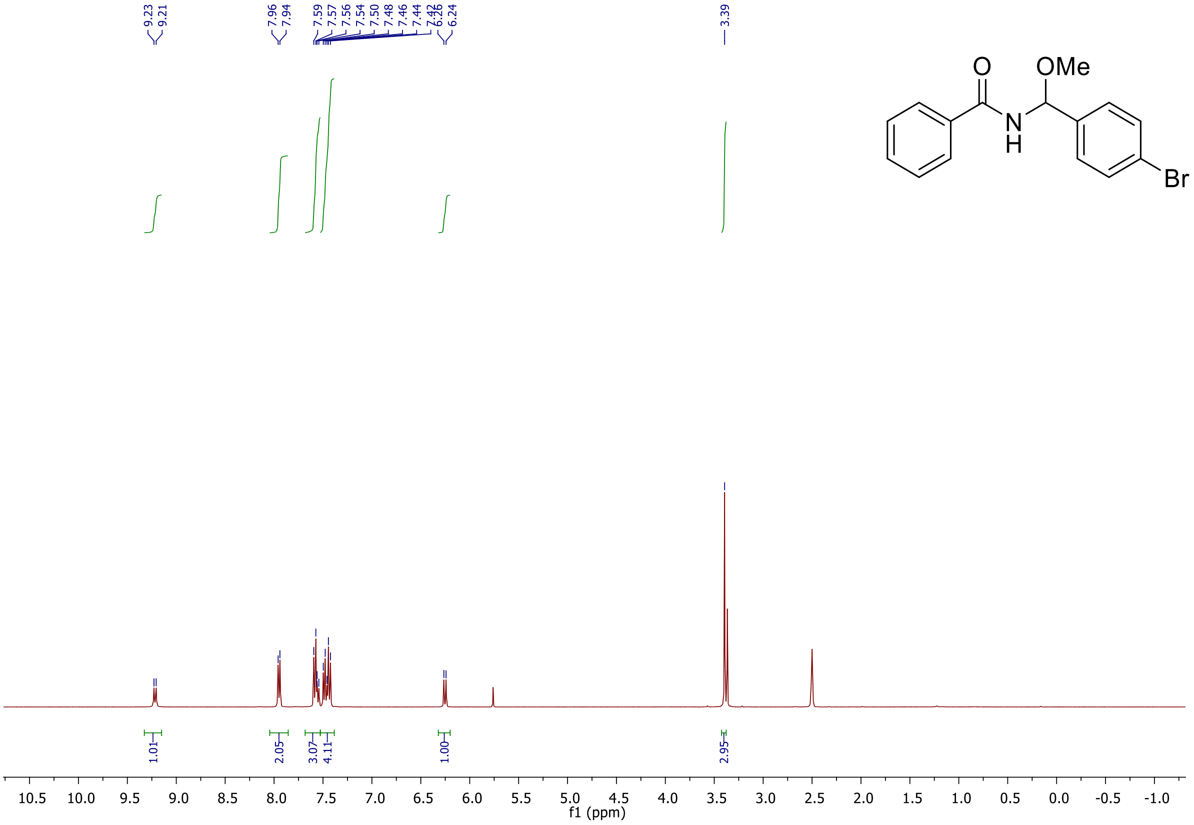

$^{13}C\{^{1}H\}$ NMR (101 MHz, DMSO-d_6)

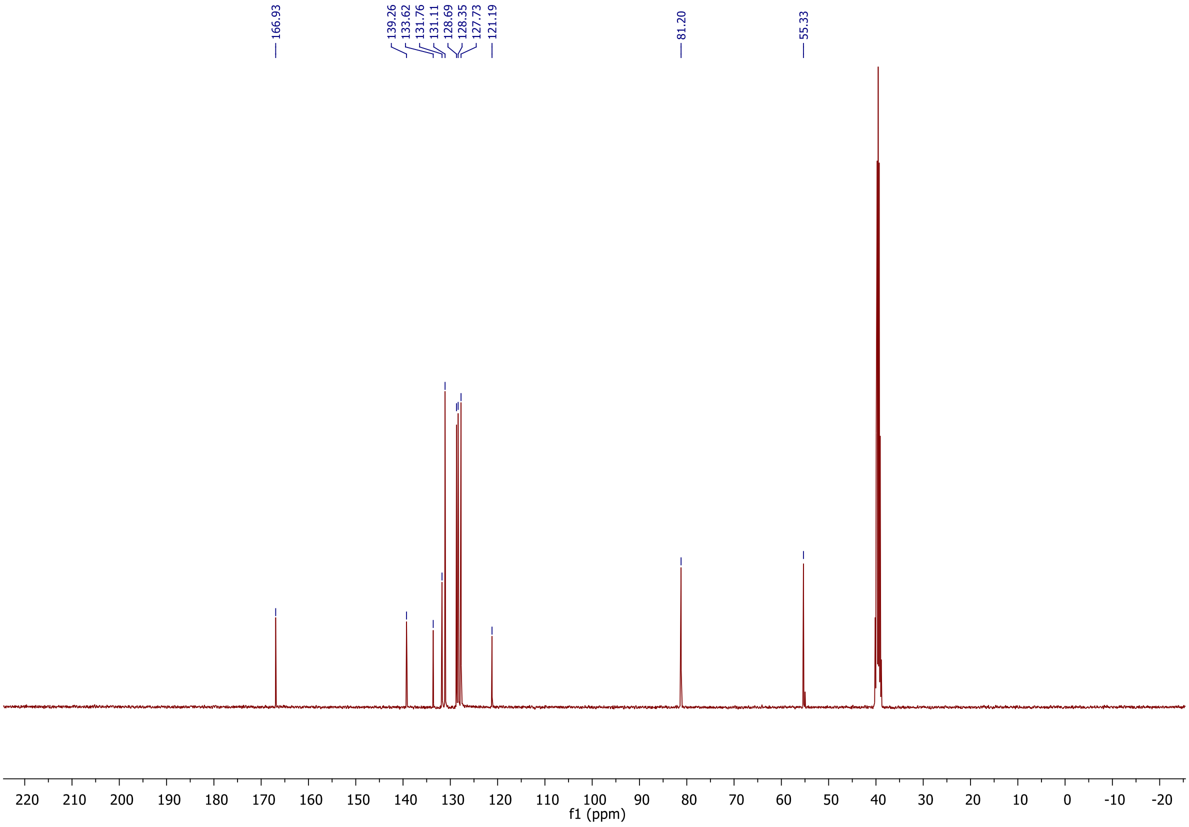

Figure S.3. ***N*-{methoxy[4-(trifluoromethyl)phenyl]methyl}benzamide (1c)**

^{1}H NMR (400 MHz, DMSO-d_6)

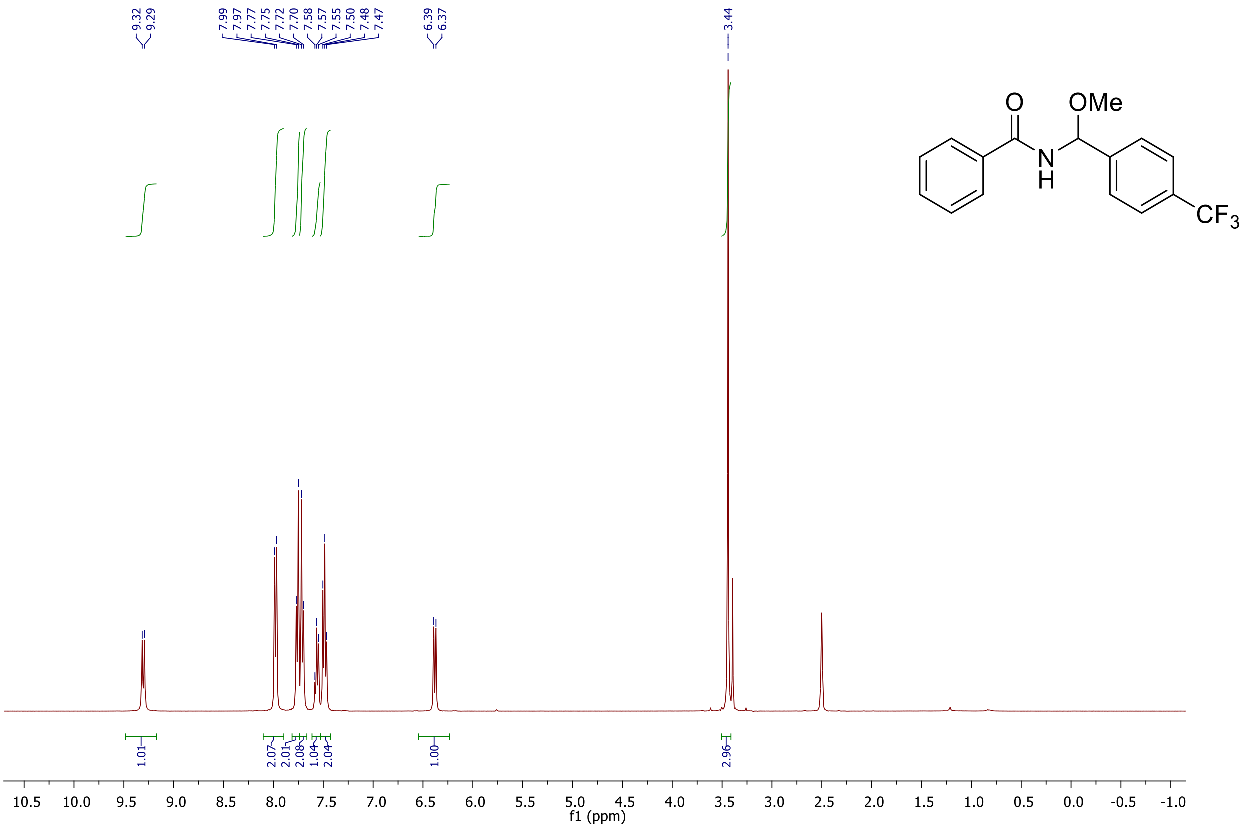

$^{13}C\{^{1}H\}$ NMR (101 MHz, DMSO-d_6)

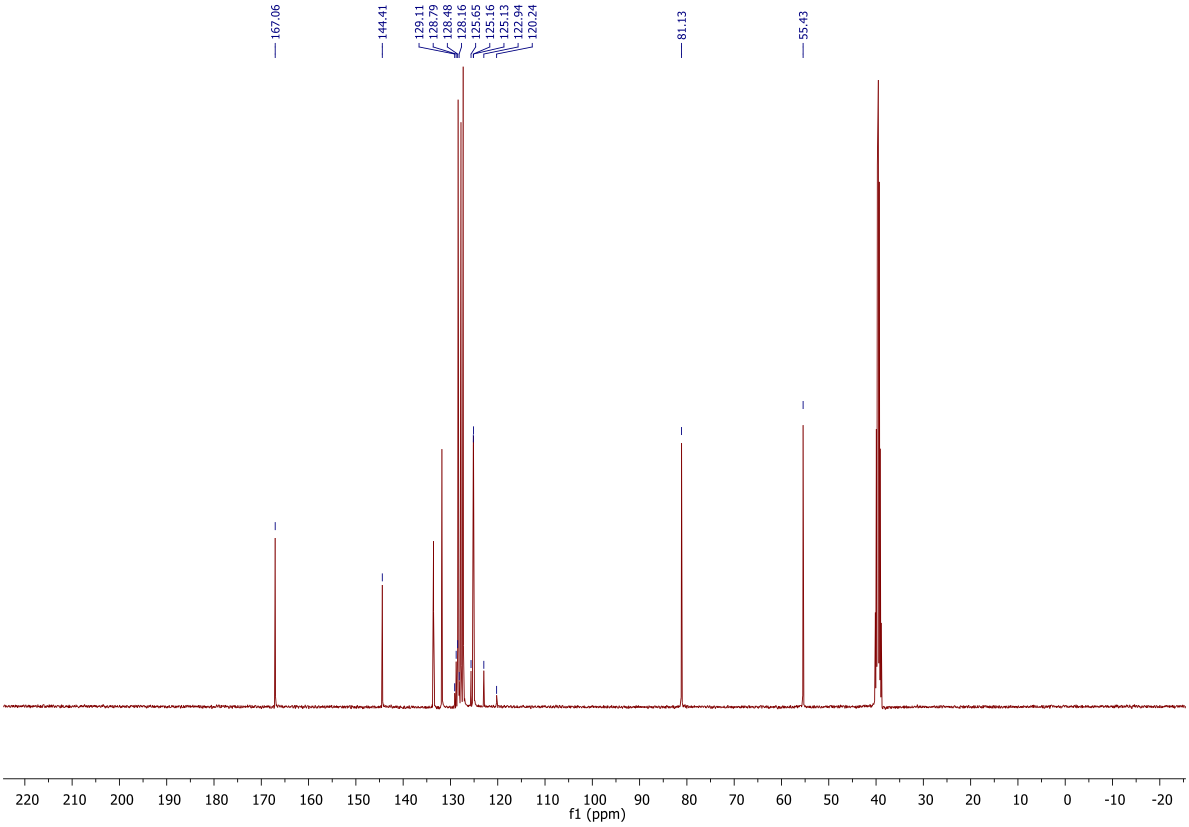

Figure S.4. *N*-[(4-chlorophenyl)(methoxy)methyl]benzamide (1d)

^{1}H NMR (400 MHz, DMSO-d_6)

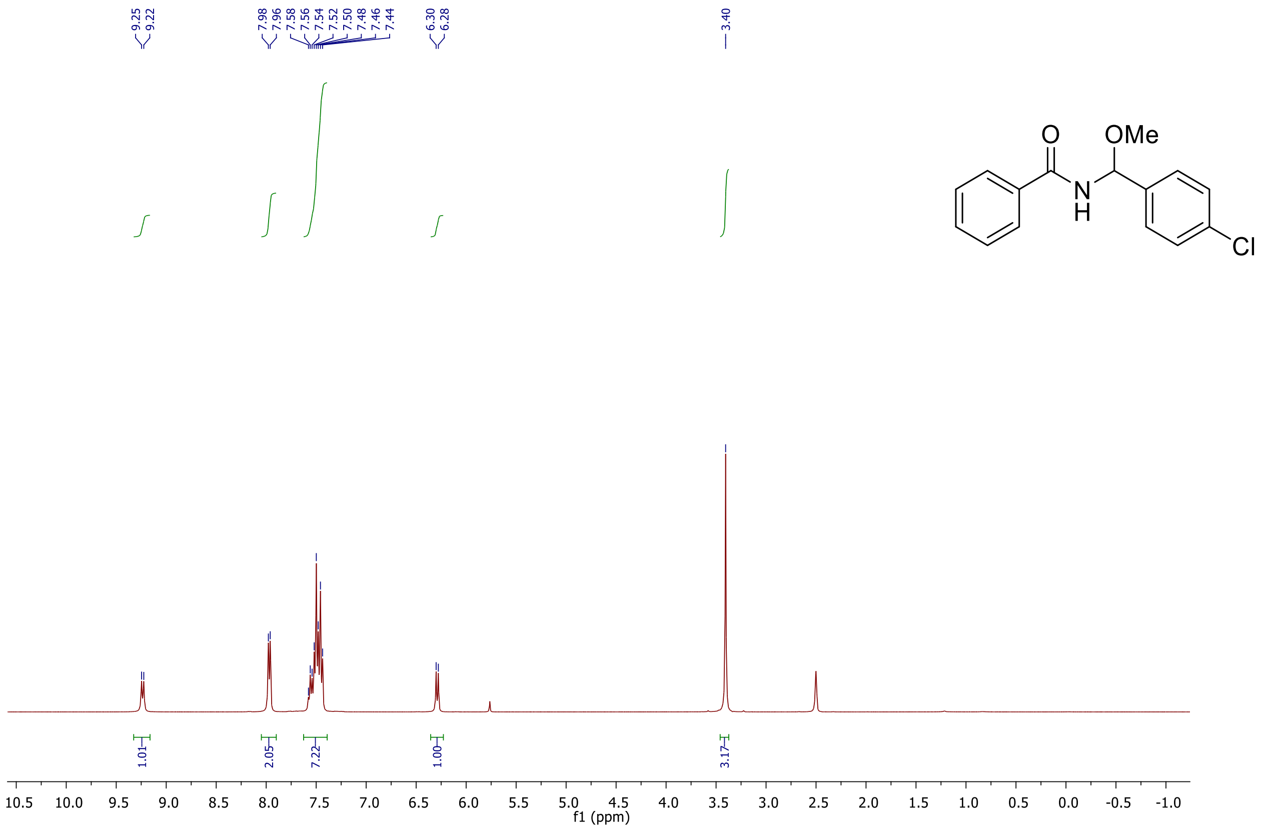

$^{13}C\{^{1}H\}$ NMR (101 MHz, DMSO-d_6)

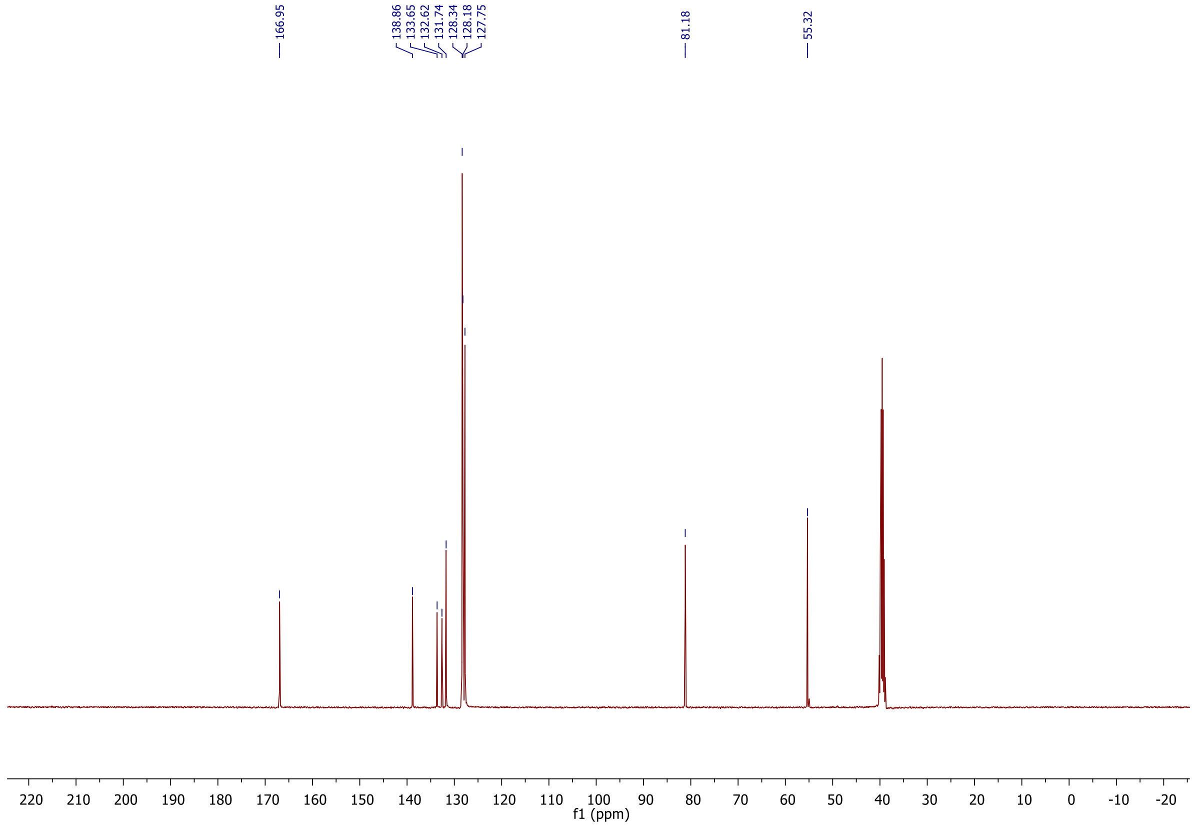

Figure S.5. ***N*-[methoxy(4-nitrophenyl)methyl]benzamide (1e)**

^{1}H NMR (400 MHz, DMSO-d_6)

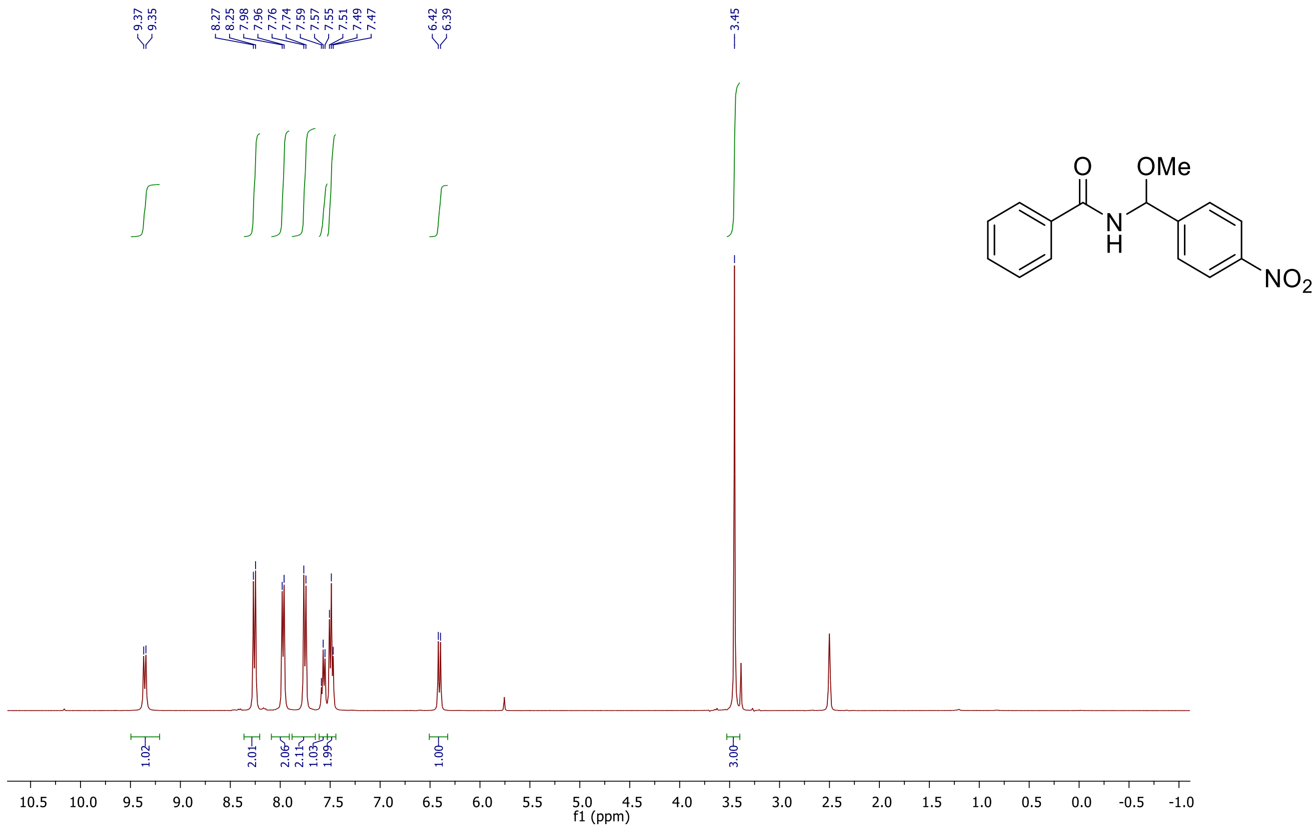

$^{13}C\{^{1}H\}$ NMR (101 MHz, DMSO-d_6)

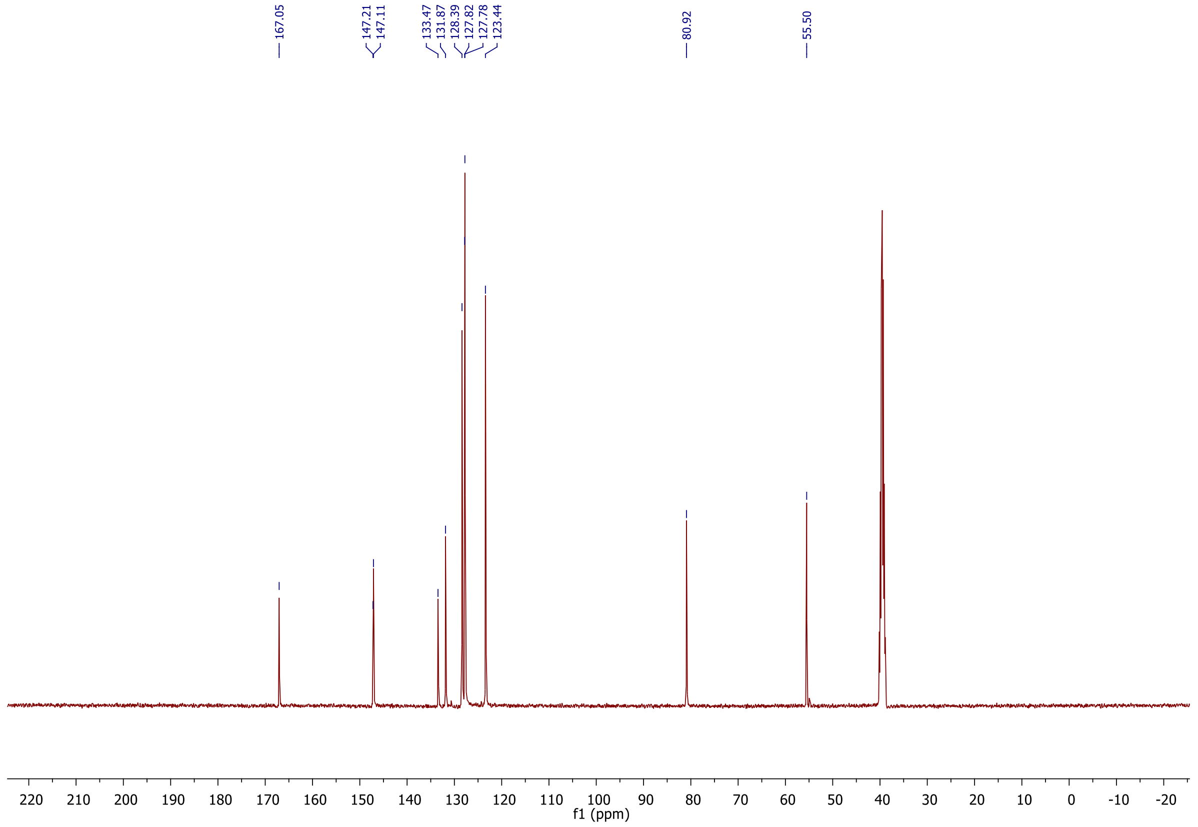

Figure S.6. ***N*-{(4-cyanophenyl)[(propan-2-yl)oxy]methyl}benzamide (1f)**

^{1}H NMR (400 MHz, DMSO-d_6)

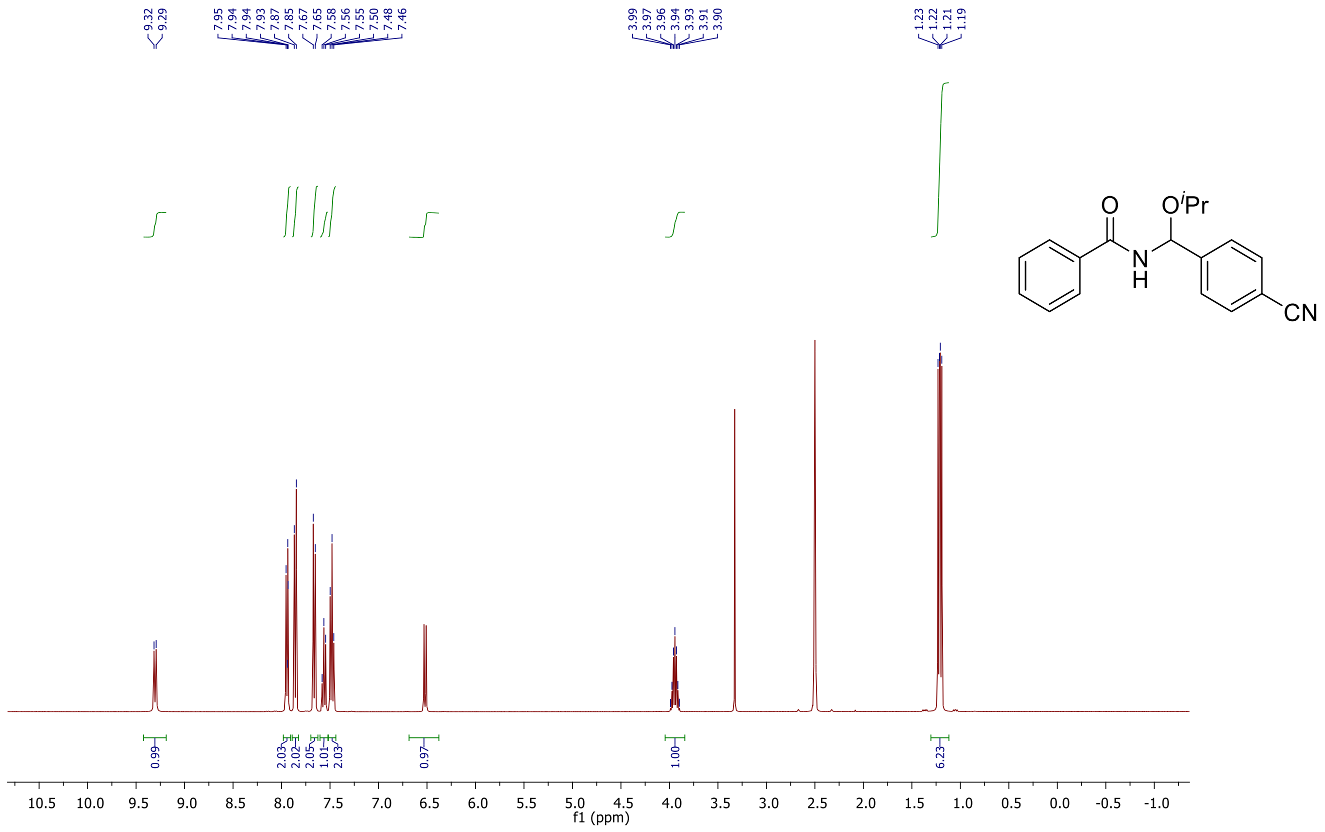

$^{13}C\{^{1}H\}$ NMR (101 MHz, DMSO-d$_6$)

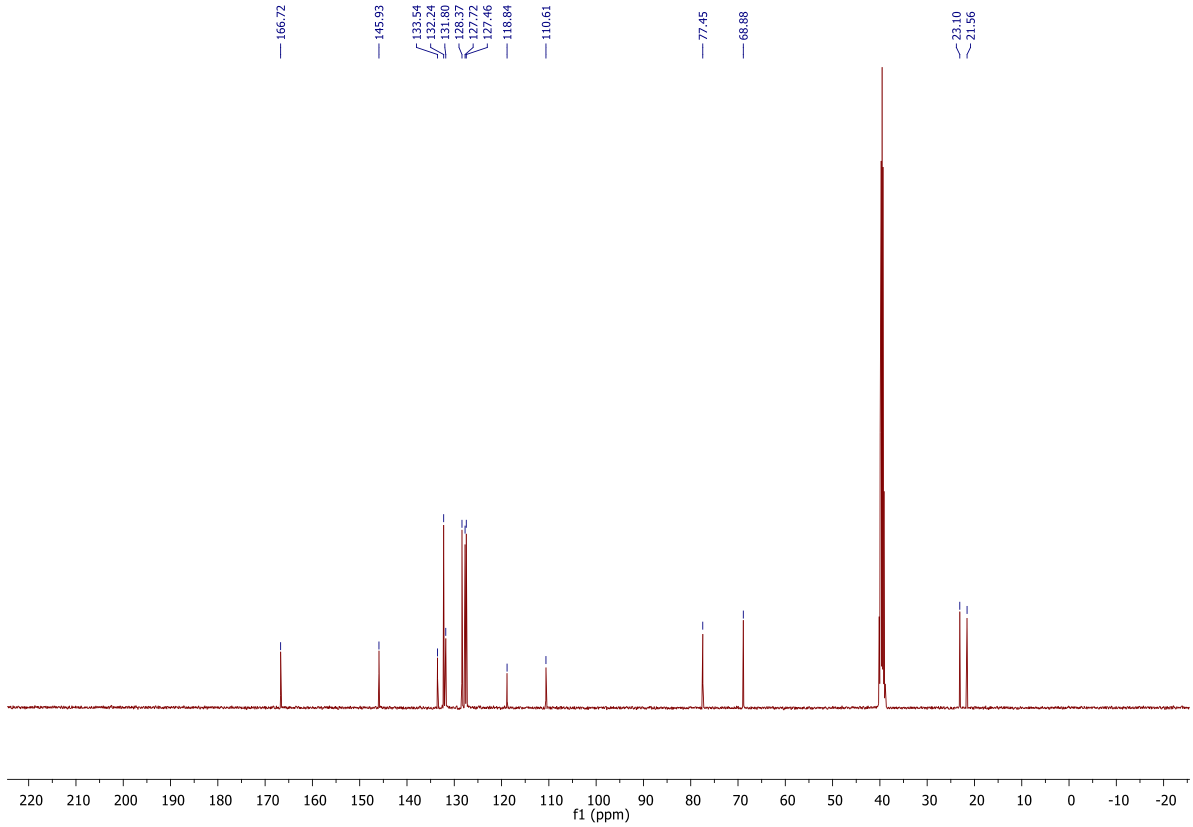

Figure S.7. *N*-[(3-cyanophenyl)(methoxy)methyl]benzamide (1g)

^{1}H NMR (400 MHz, DMSO-d$_6$)

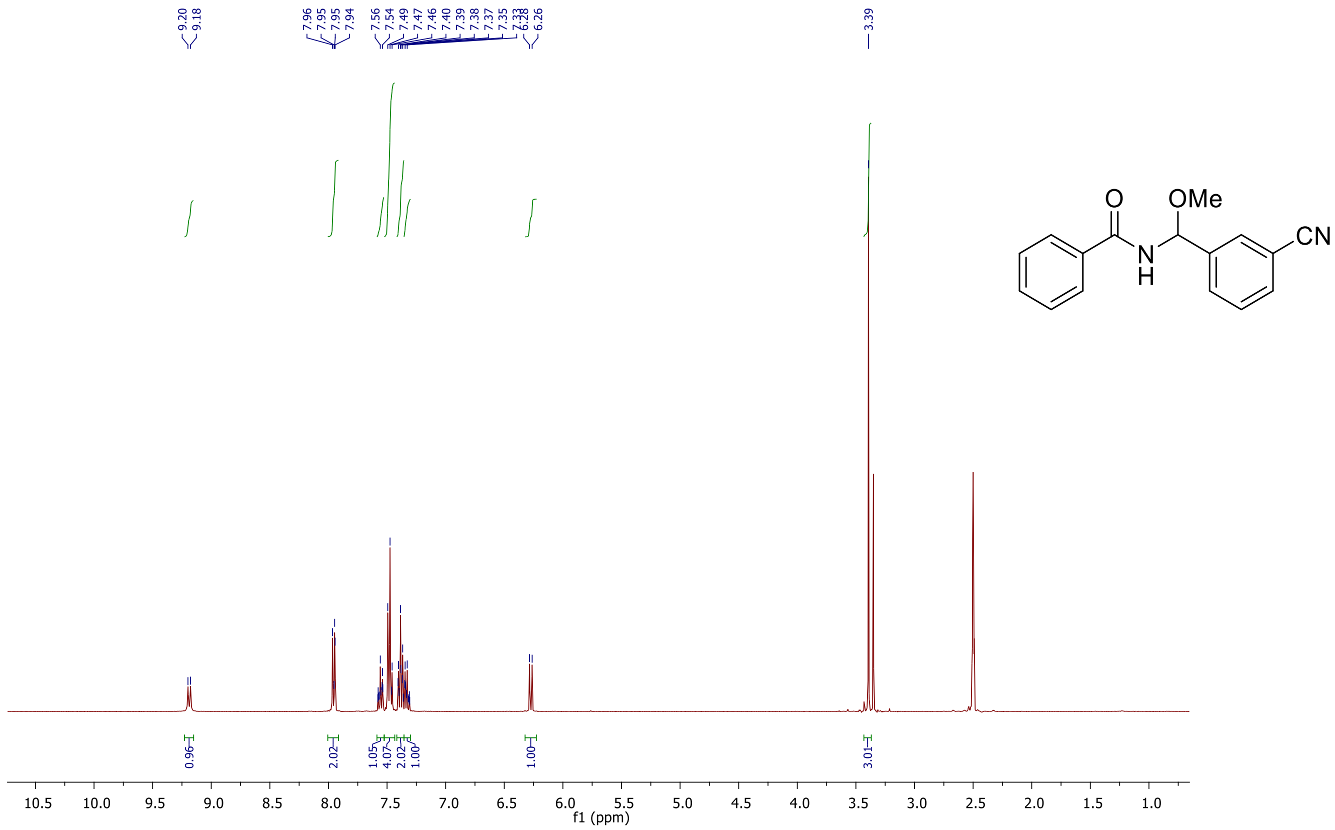

$^{13}C\{^{1}H\}$ NMR (101 MHz, DMSO-d_6)

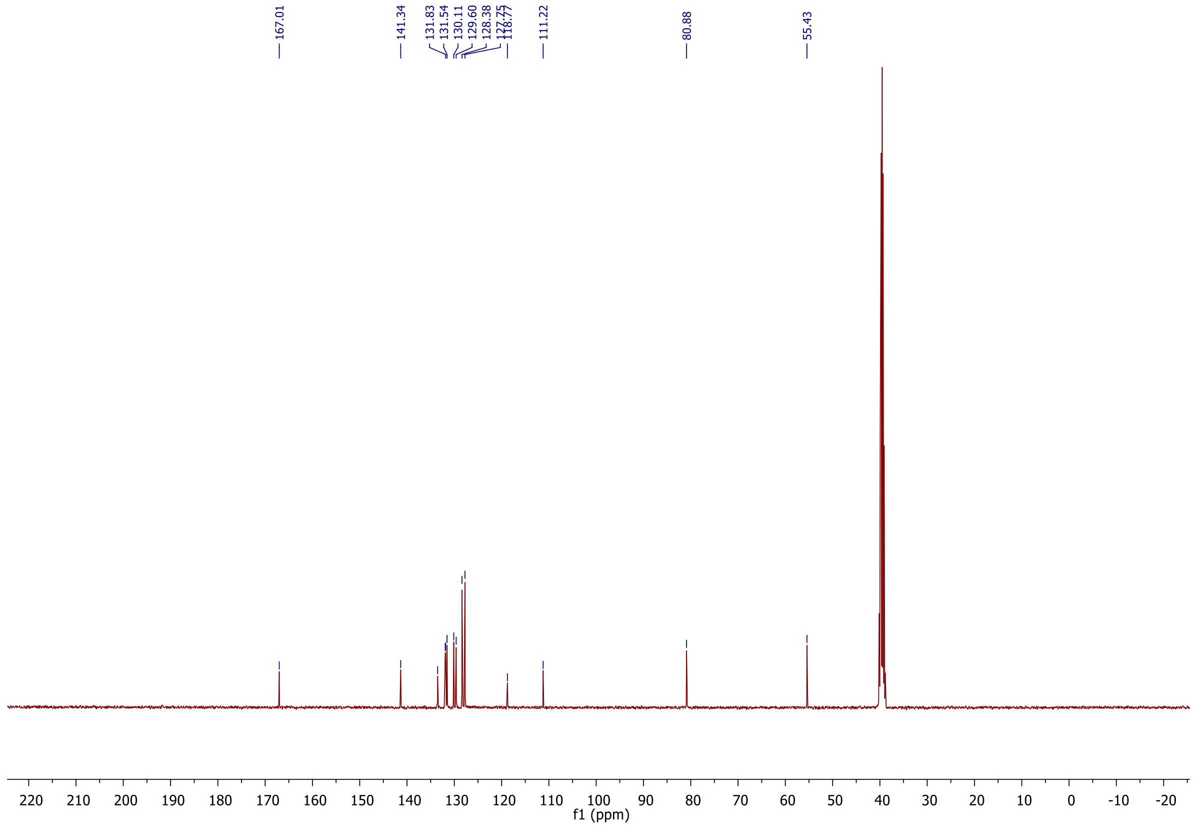

Figure S.8. *N*-[methoxy(2-methoxyphenyl)methyl]benzamide (1h)

^{1}H NMR (400 MHz, DMSO-d_6)

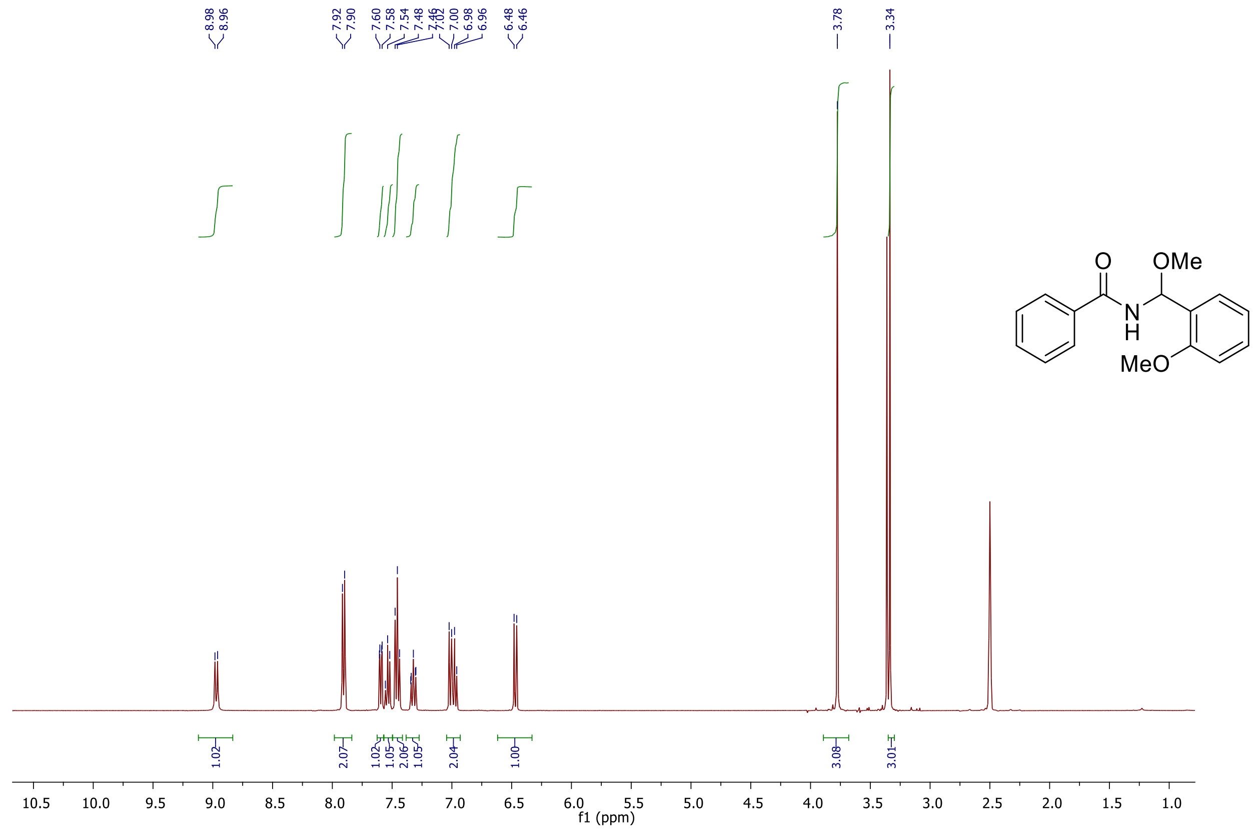

$^{13}C\{^{1}H\}$ NMR (101 MHz, DMSO-d$_6$)

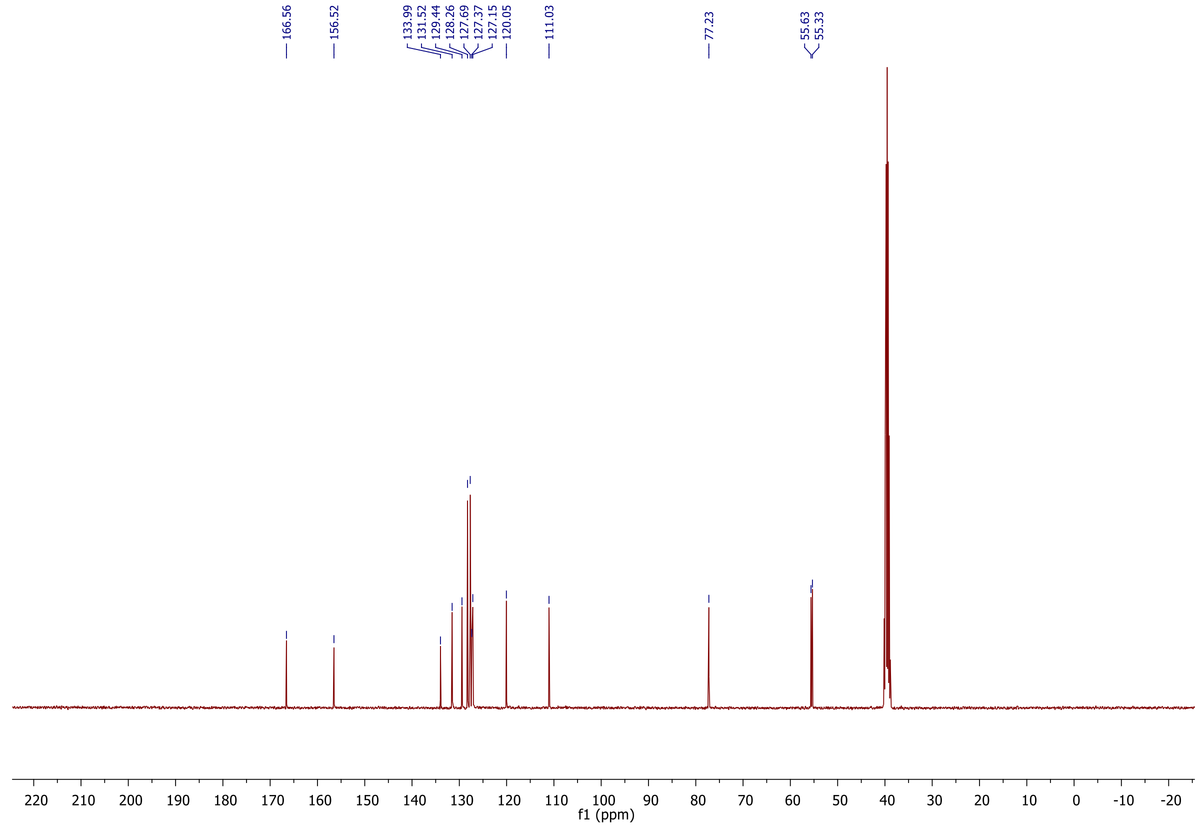

Figure S.9. *N*-{(3-methylphenyl)[(propan-2-yl)oxy]methyl}benzamide (1i)

^{1}H NMR (400 MHz, DMSO-d$_6$)

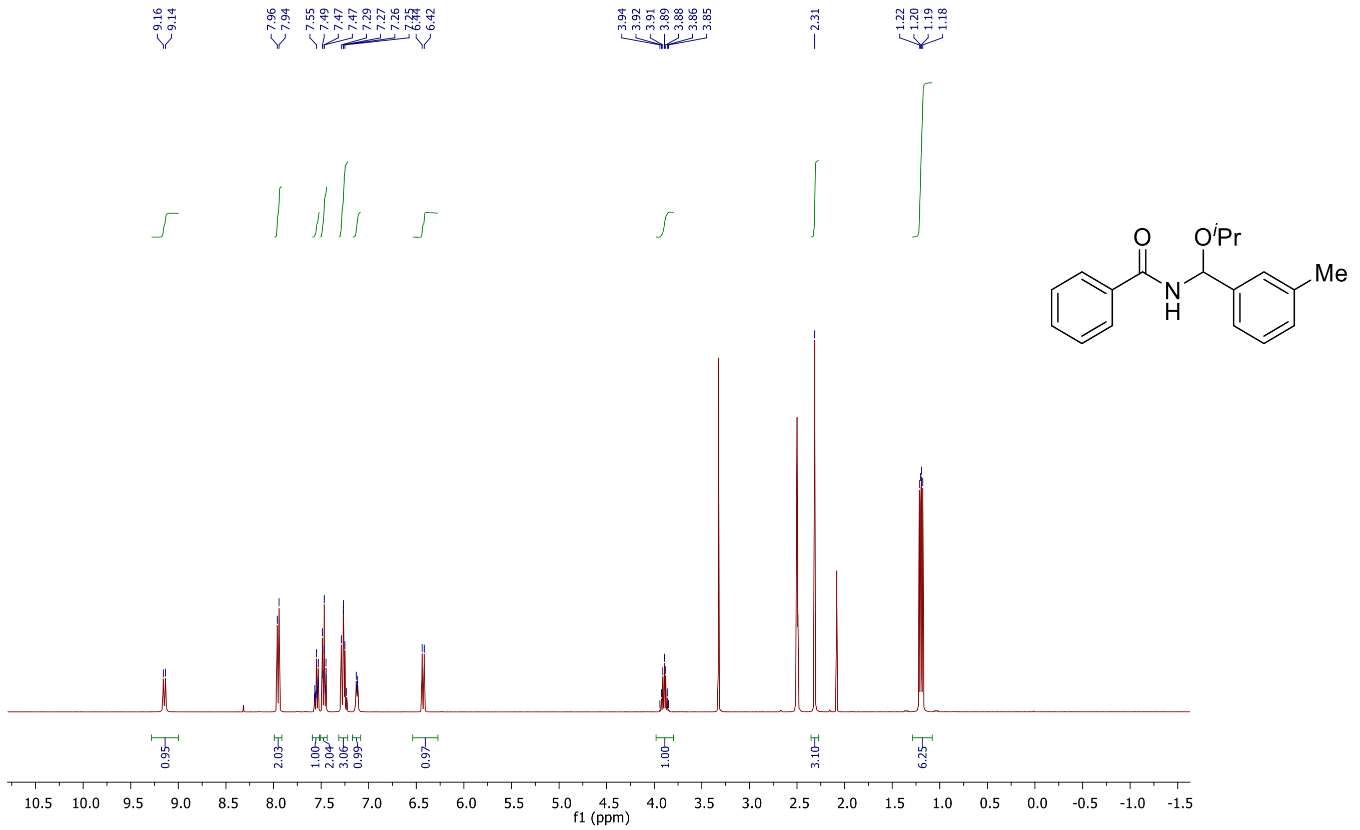

$^{13}C\{^1H\}$ NMR (101 MHz, DMSO-d_6)

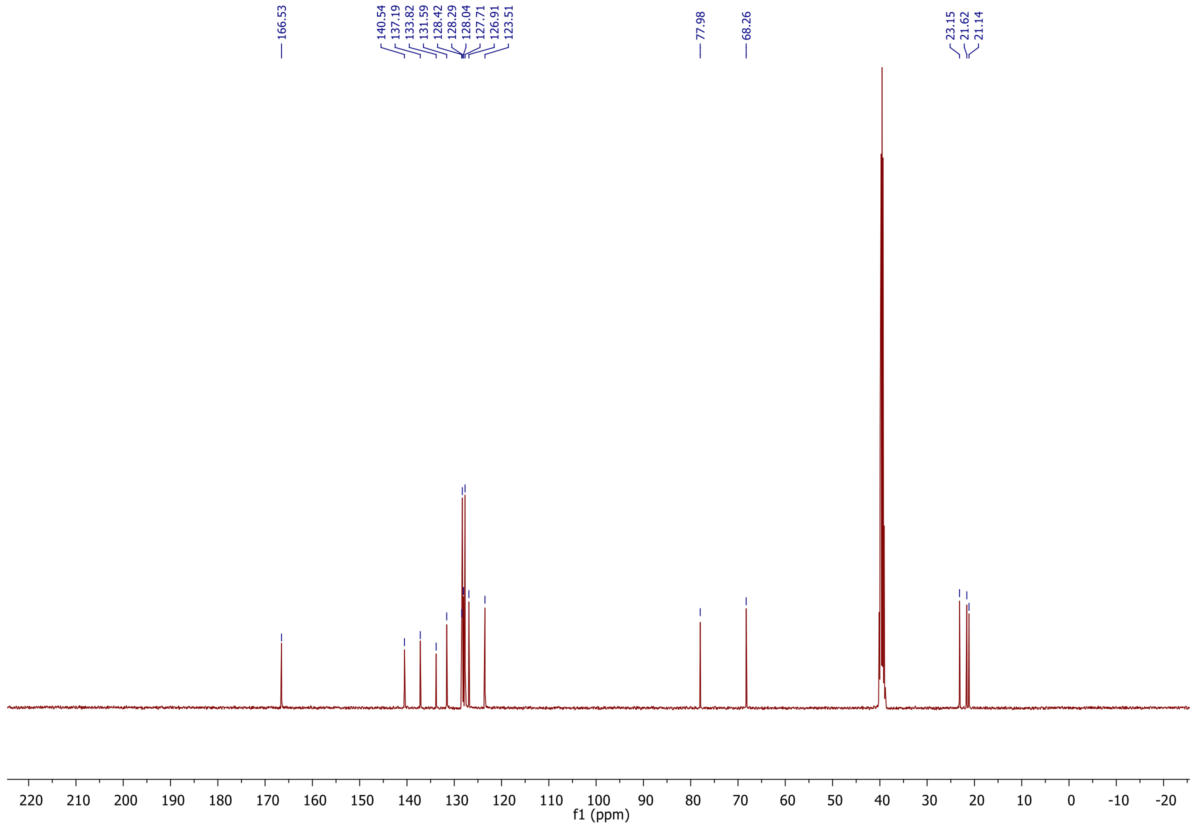

Figure S.10. *N*-[methoxy(4-methoxyphenyl)methyl]benzamide (1j)

^1H NMR (400 MHz, DMSO-d_6)

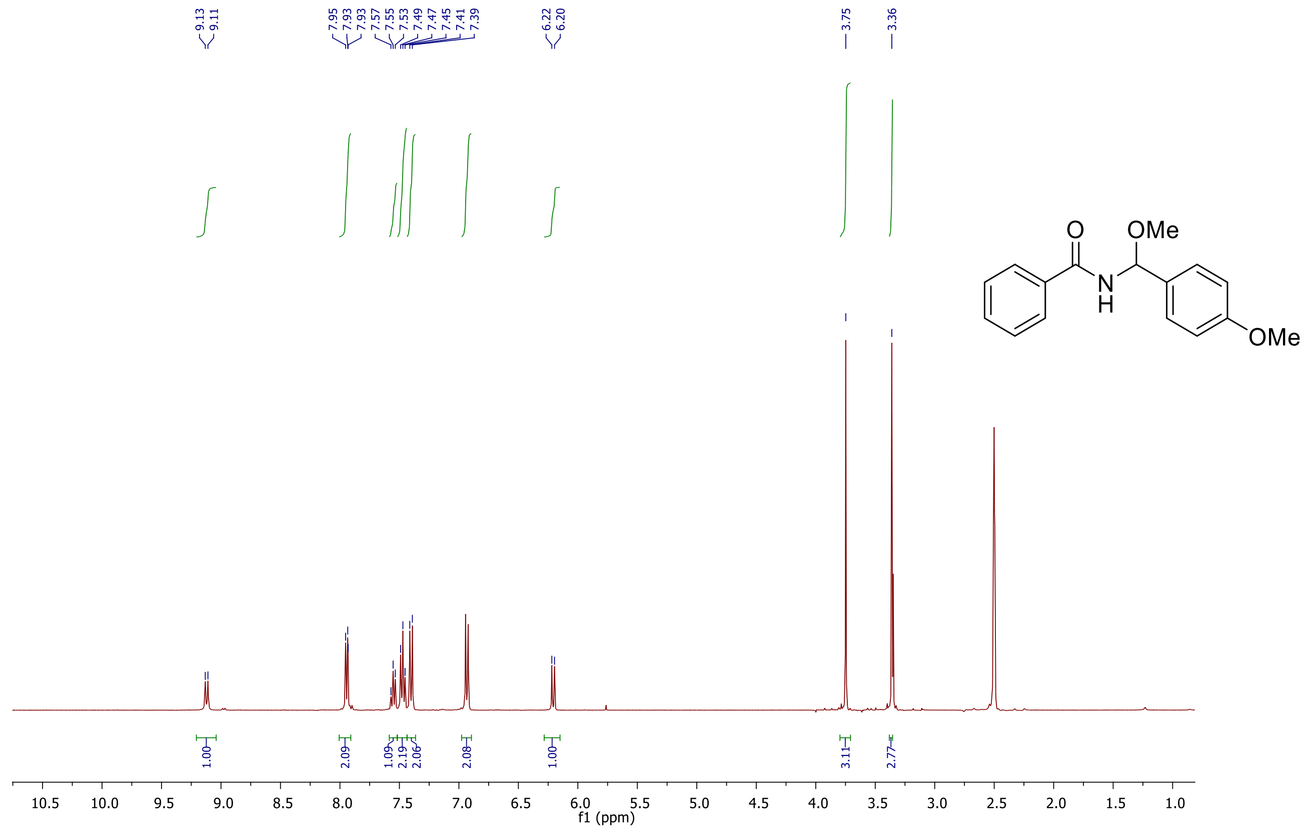

$^{13}C\{^1H\}$ NMR (101 MHz, DMSO-d$_6$)

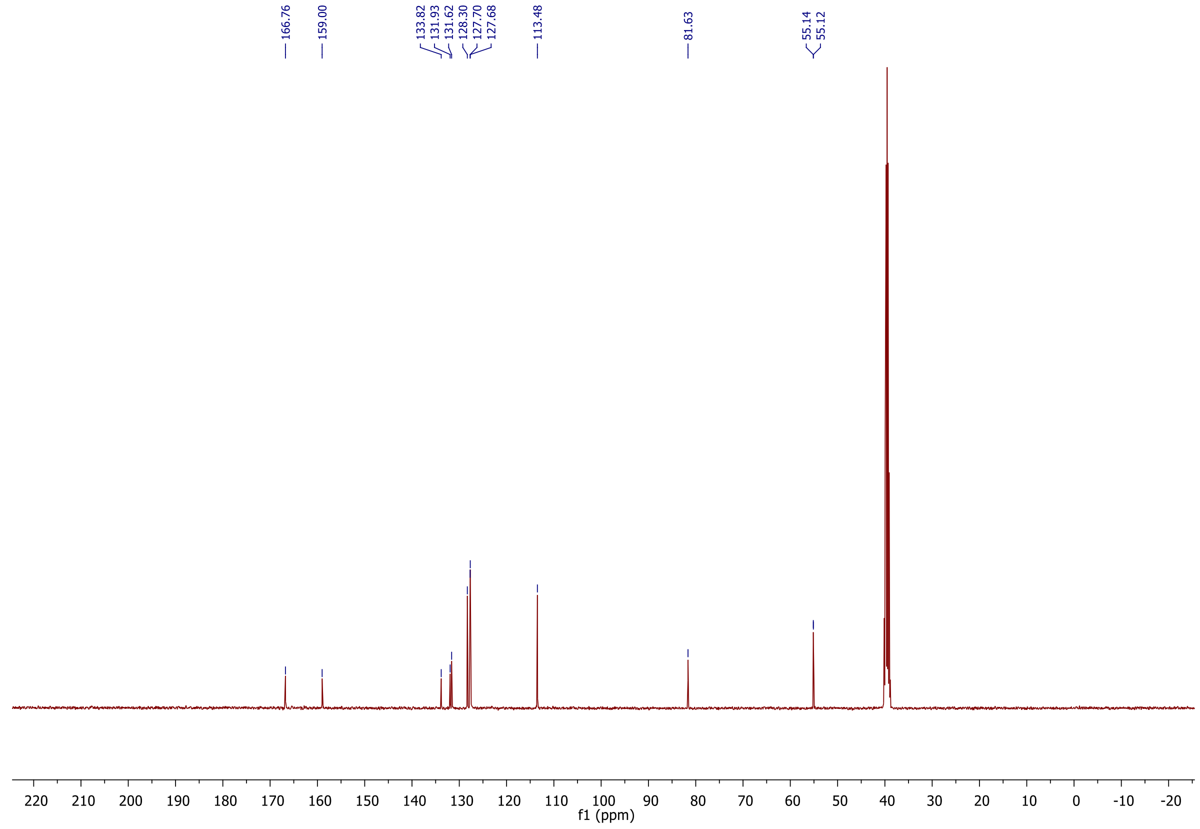

Figure S.11. ***N*-{[(propan-2-yl)oxy](thiophen-2-yl)methyl}benzamide (1k)**

^1H NMR (400 MHz, DMSO-d$_6$)

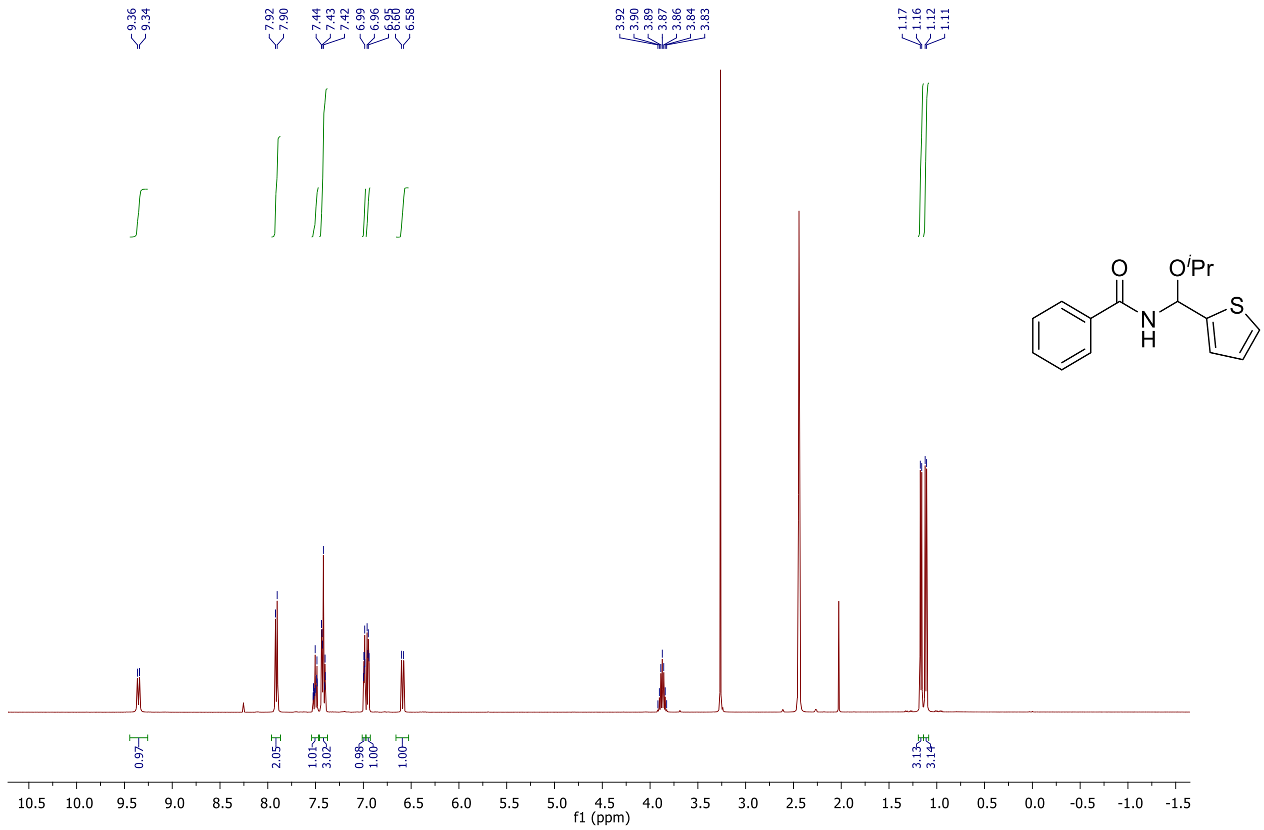

$^{13}C\{^{1}H\}$ NMR (101 MHz, DMSO-d_6)

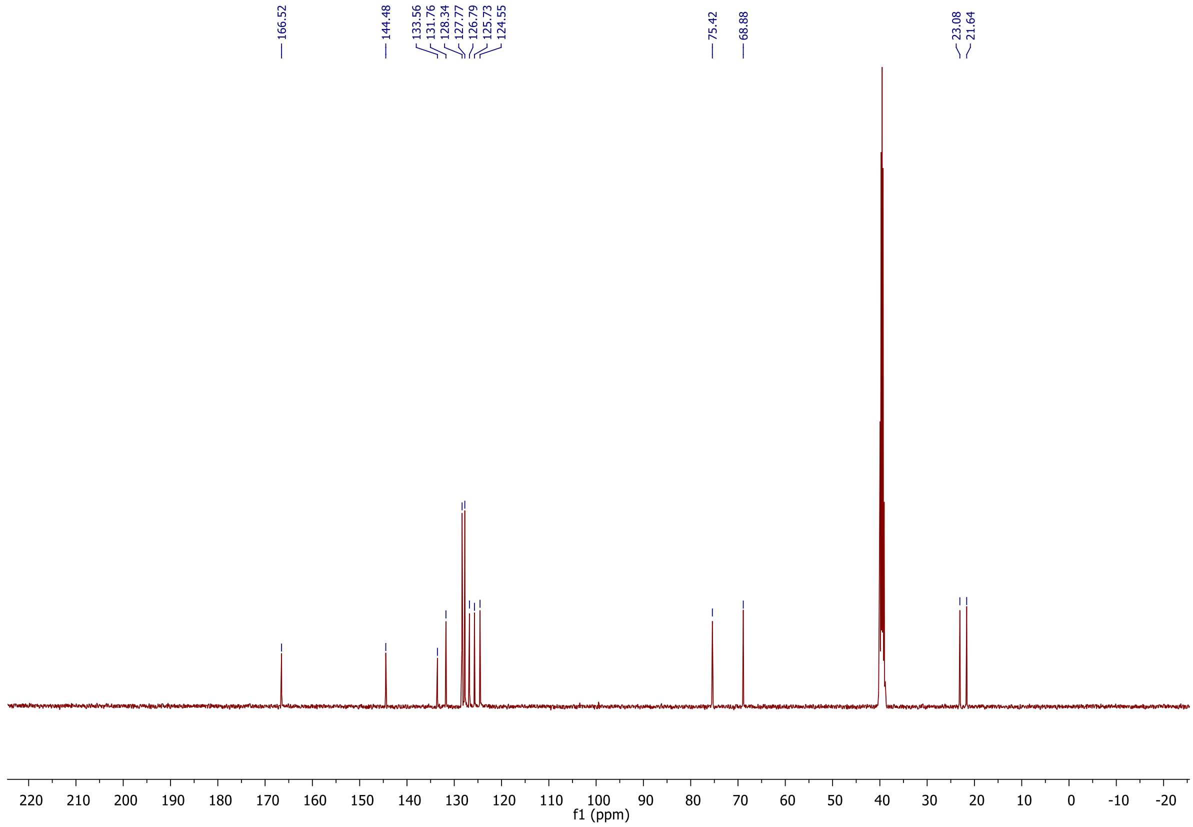

Figure S.12. ***N*-{(naphthalen-2-yl)[(propan-2-yl)oxy]methyl}benzamide (1l)**

^{1}H NMR (400 MHz, DMSO-d_6)

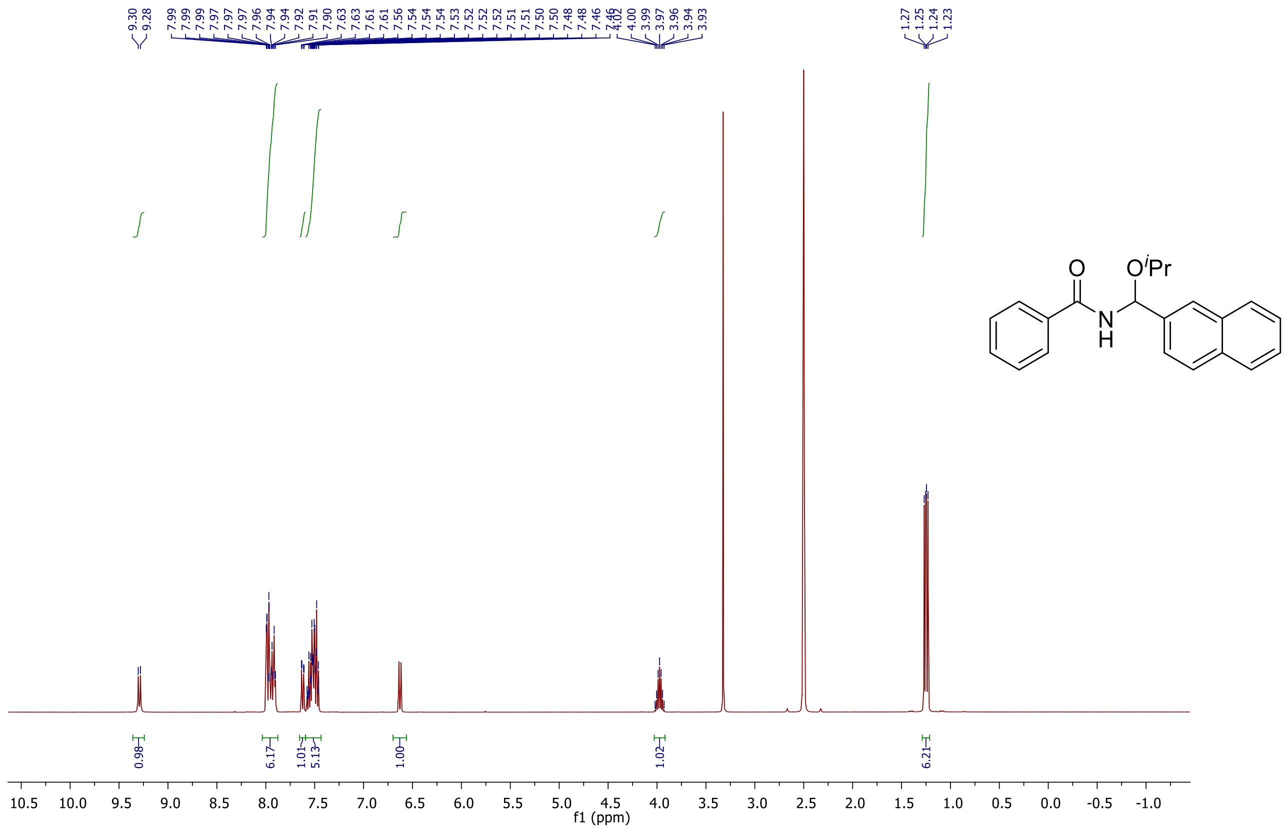

$^{13}C\{^1H\}$ NMR (101 MHz, DMSO-d$_6$)

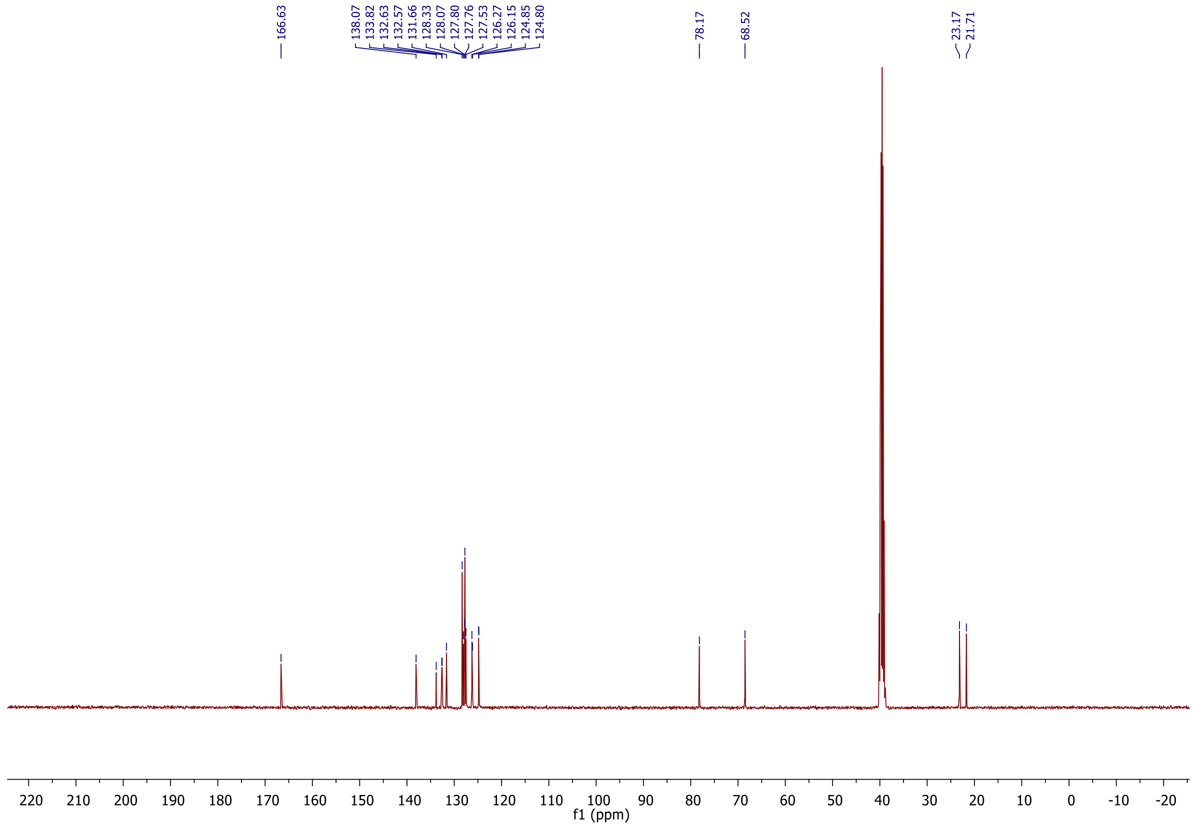

Figure S.13. 4-bromo-*N*-{phenyl[(propan-2-yl)oxy]methyl}benzamide (1m)

^1H NMR (400 MHz, DMSO-d$_6$)

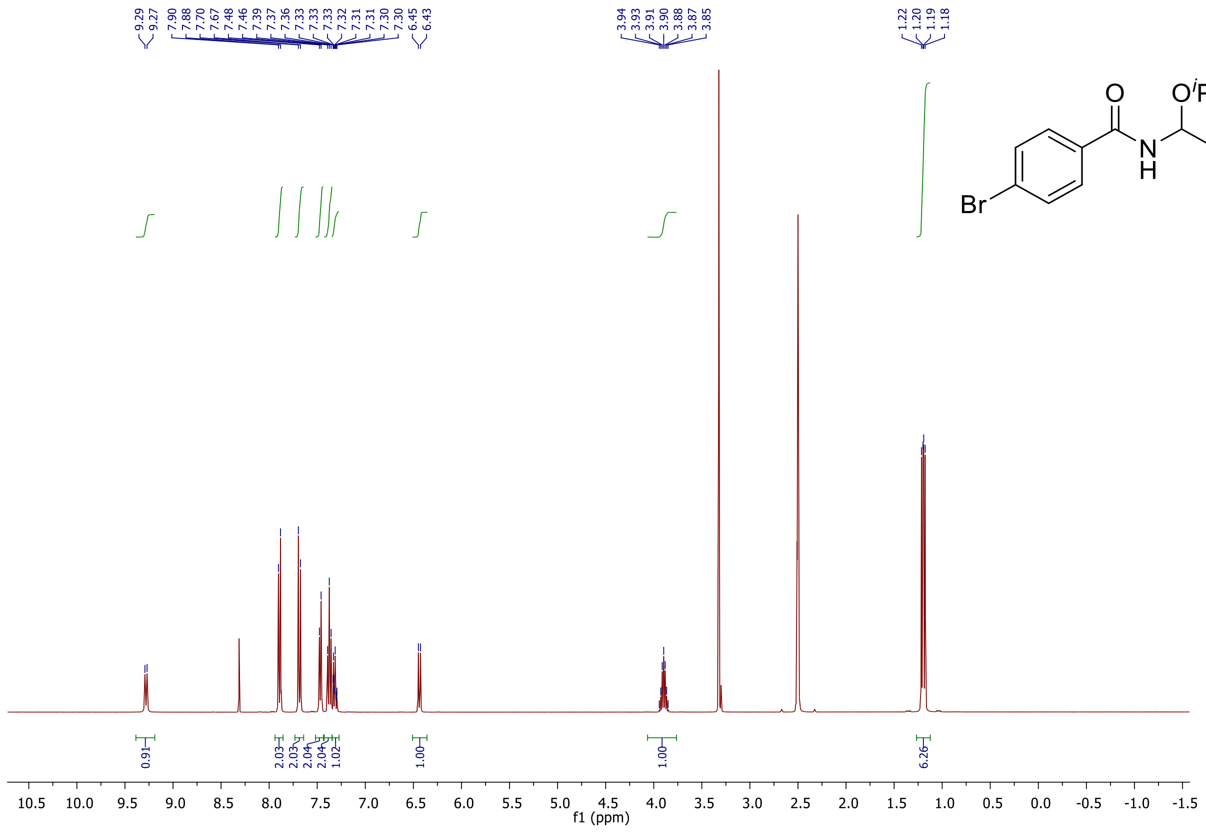

$^{13}C\{^1H\}$ NMR (101 MHz, DMSO-d$_6$)

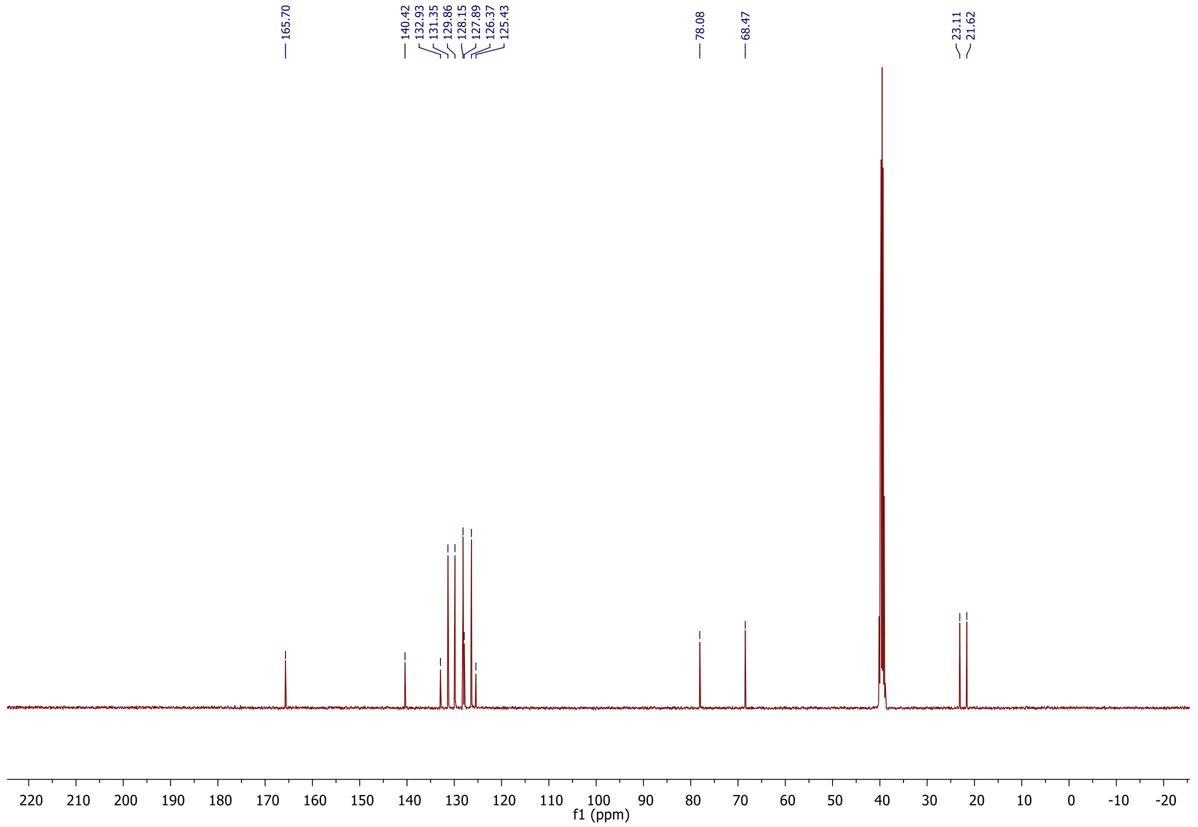

Figure S.14. 4-chloro-*N*-{phenyl[(propan-2-yl)oxy]methyl}benzamide (1n)

^1H NMR (400 MHz, DMSO-d$_6$)

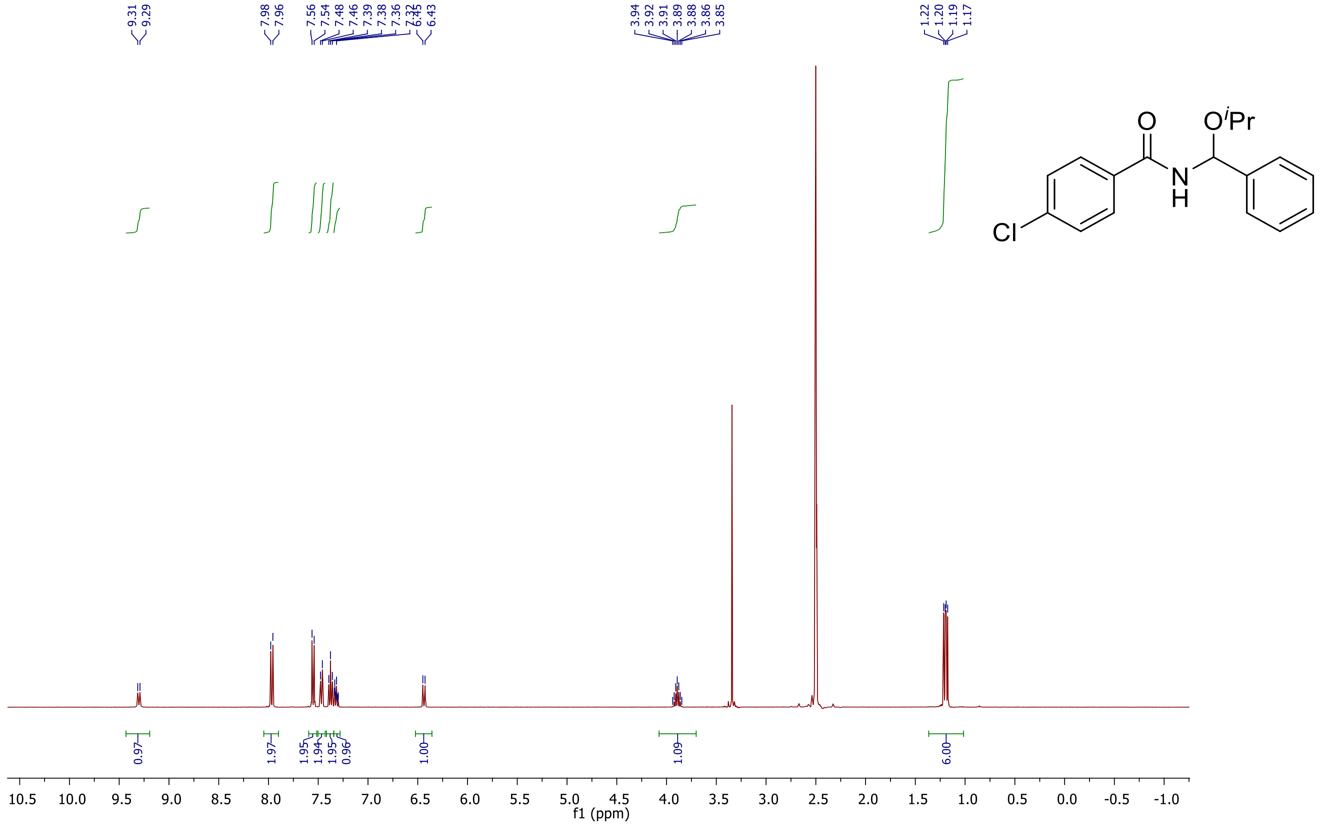

$^{13}C\{^{1}H\}$ NMR (101 MHz, DMSO-d$_6$)

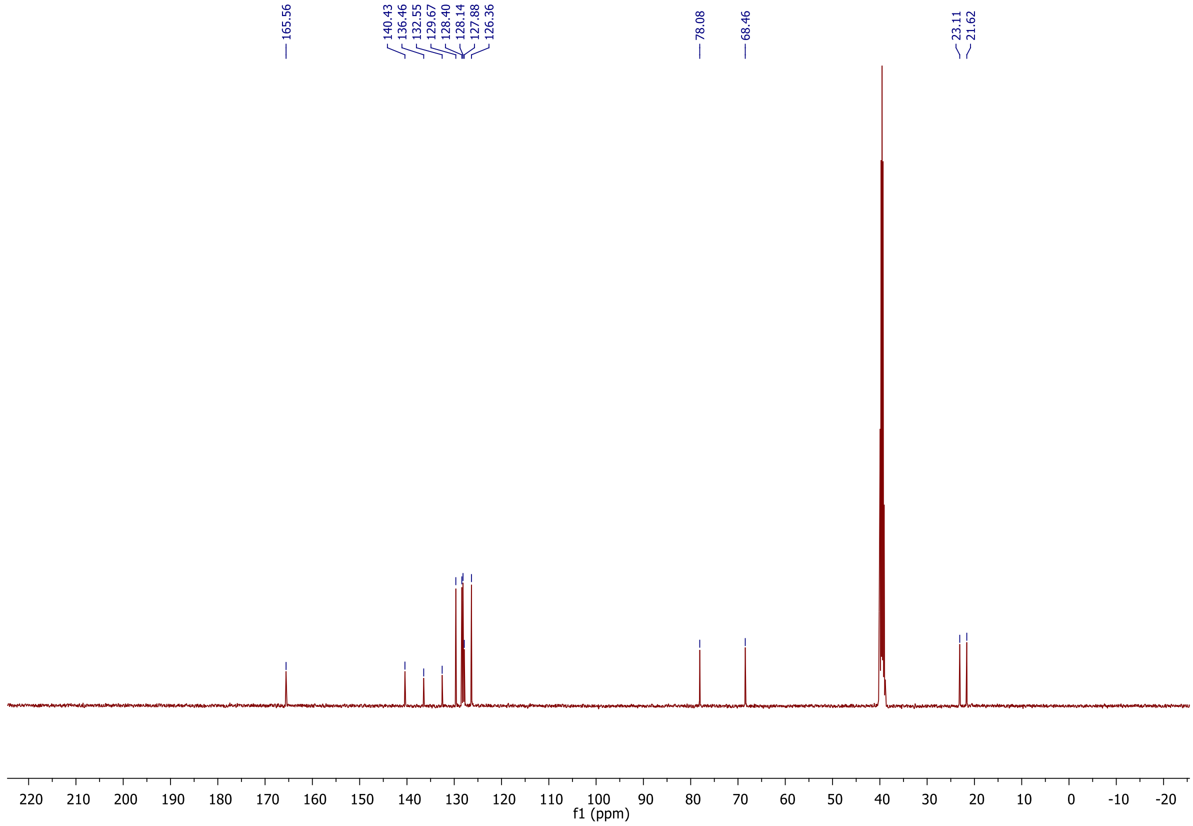

Figure S.15. ***N*-{phenyl[(propan-2-yl)oxy]methyl}-4-(trifluoromethyl)benzamide (1o)**

^{1}H NMR (400 MHz, DMSO-d$_6$)

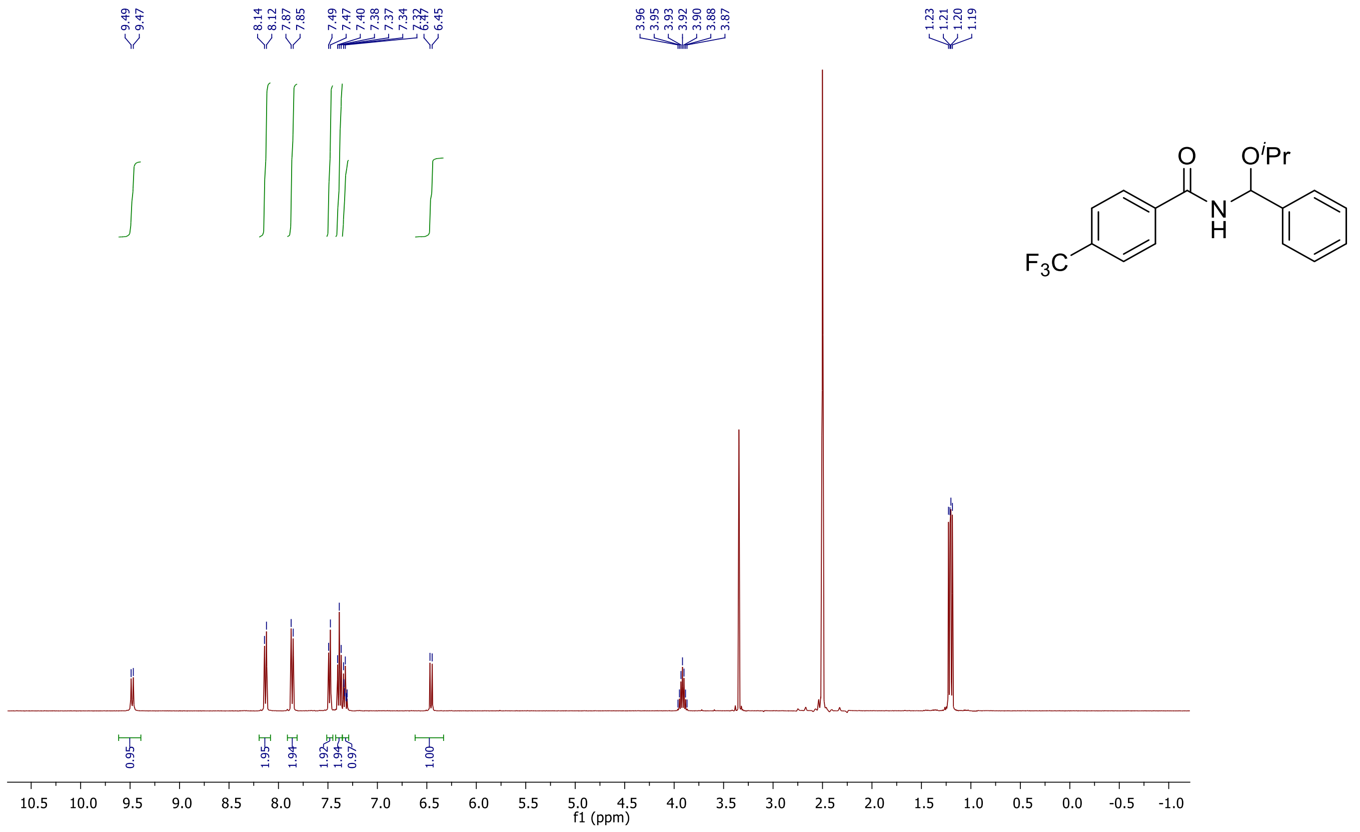

$^{13}C\{^{1}H\}$ NMR (101 MHz, DMSO-d$_6$)

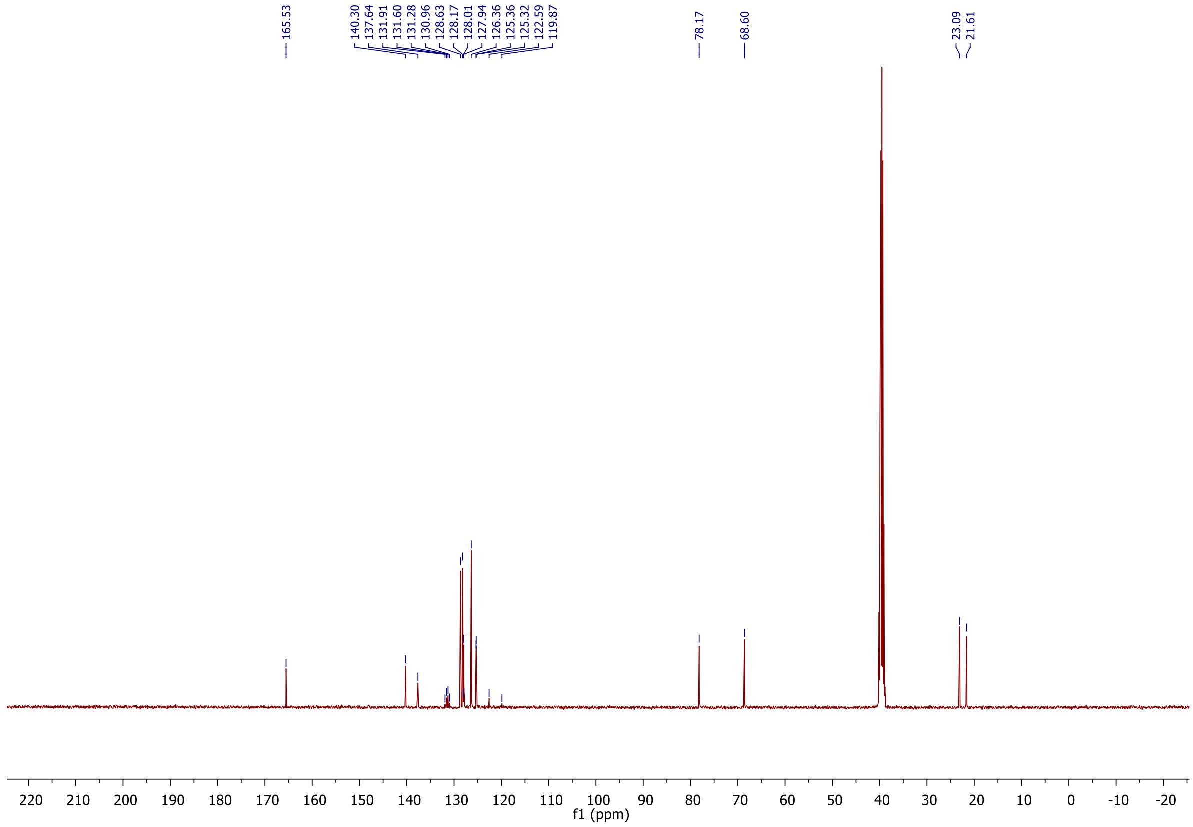

Figure S.16. 3,5-difluoro-*N*-{phenyl[(propan-2-yl)oxy]methyl}benzamide (1p)

^{1}H NMR (400 MHz, DMSO-d$_6$)

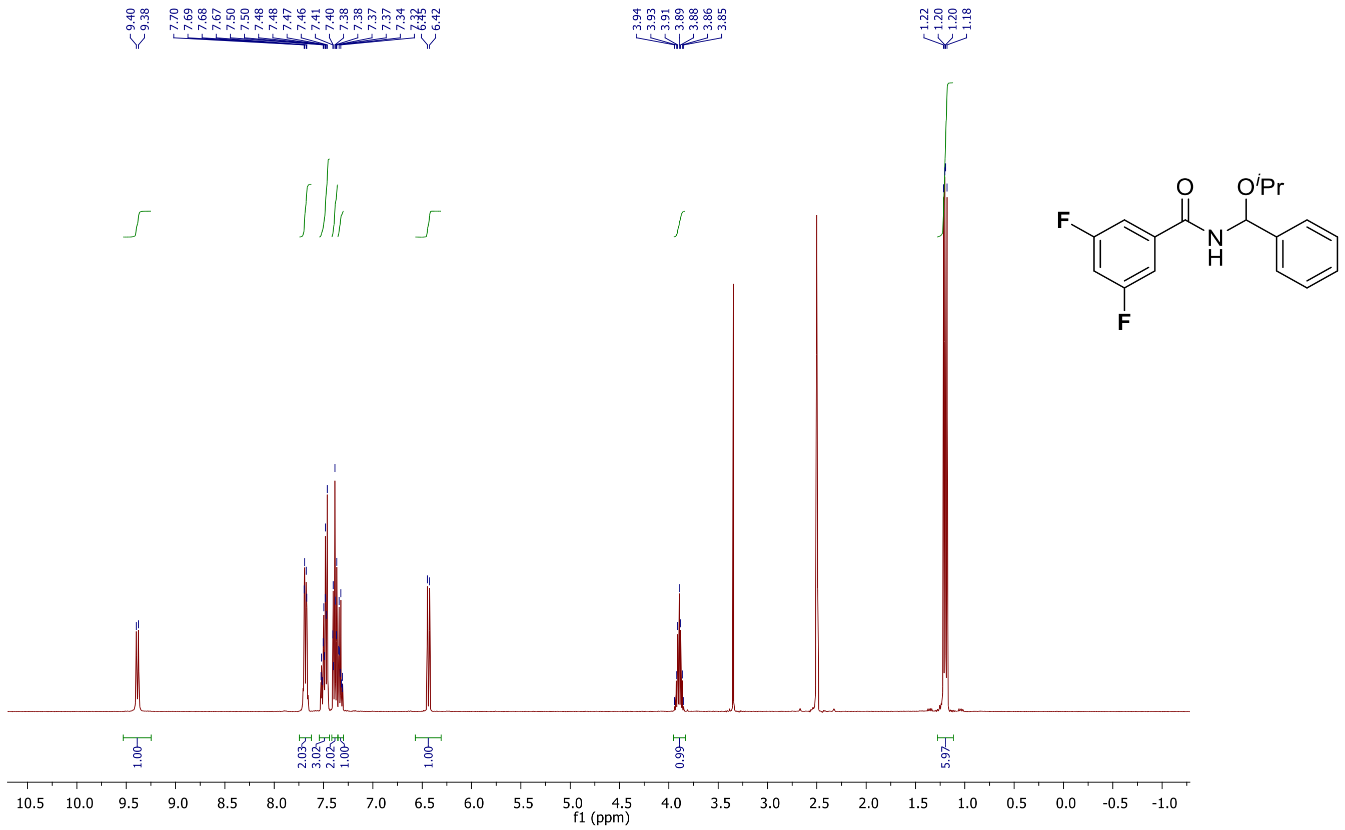

$^{13}C\{^{1}H\}$ NMR (101 MHz, DMSO-d$_6$)

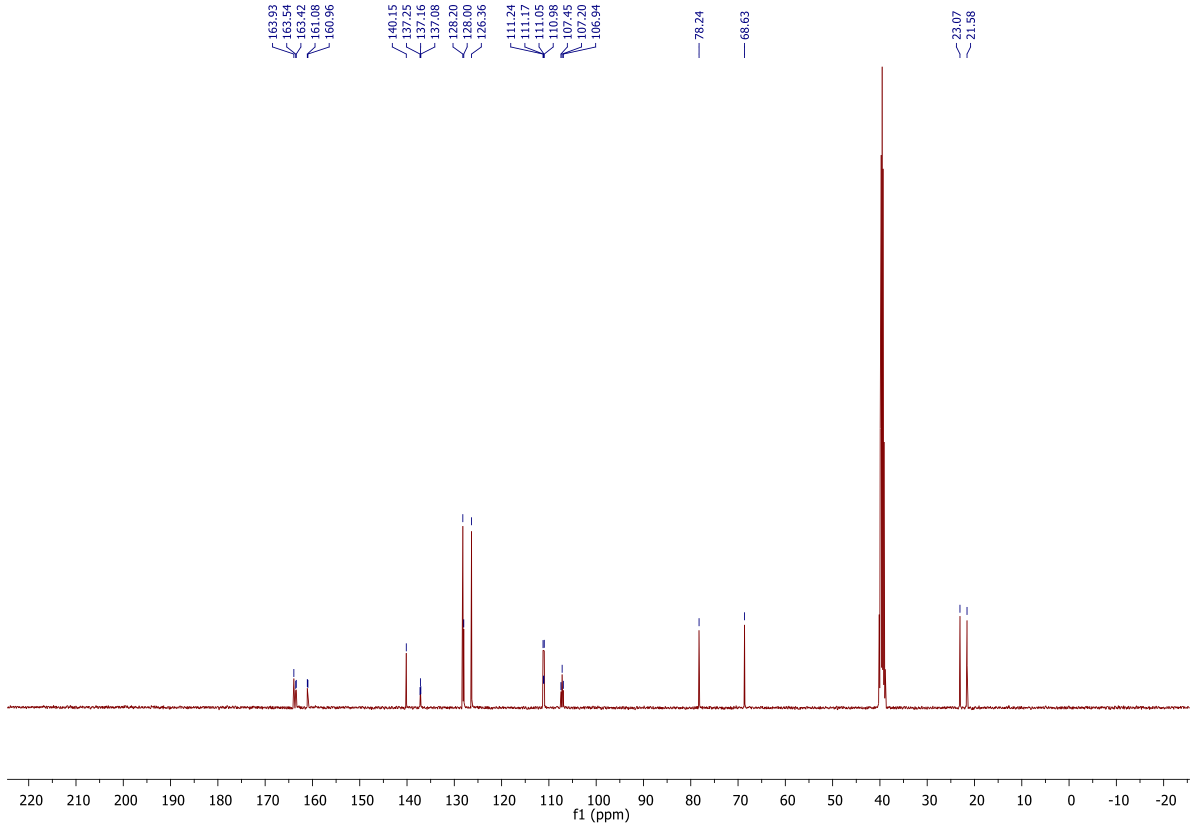

Figure S.17. 3-chloro-*N*-{phenyl[(propan-2-yl)oxy]methyl}benzamide (1q)

^{1}H NMR (400 MHz, DMSO-d$_6$)

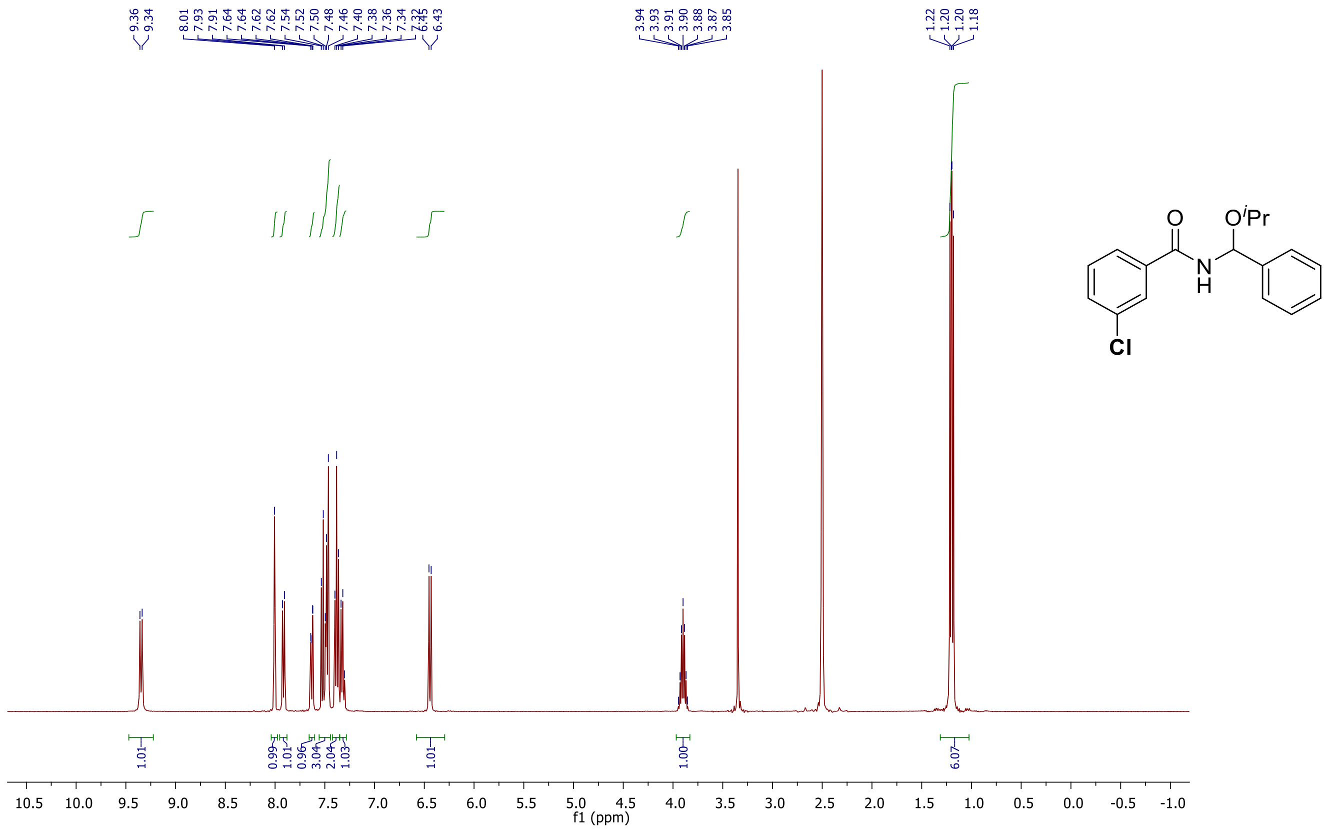

$^{13}C\{^{1}H\}$ NMR (101 MHz, DMSO-d$_6$)

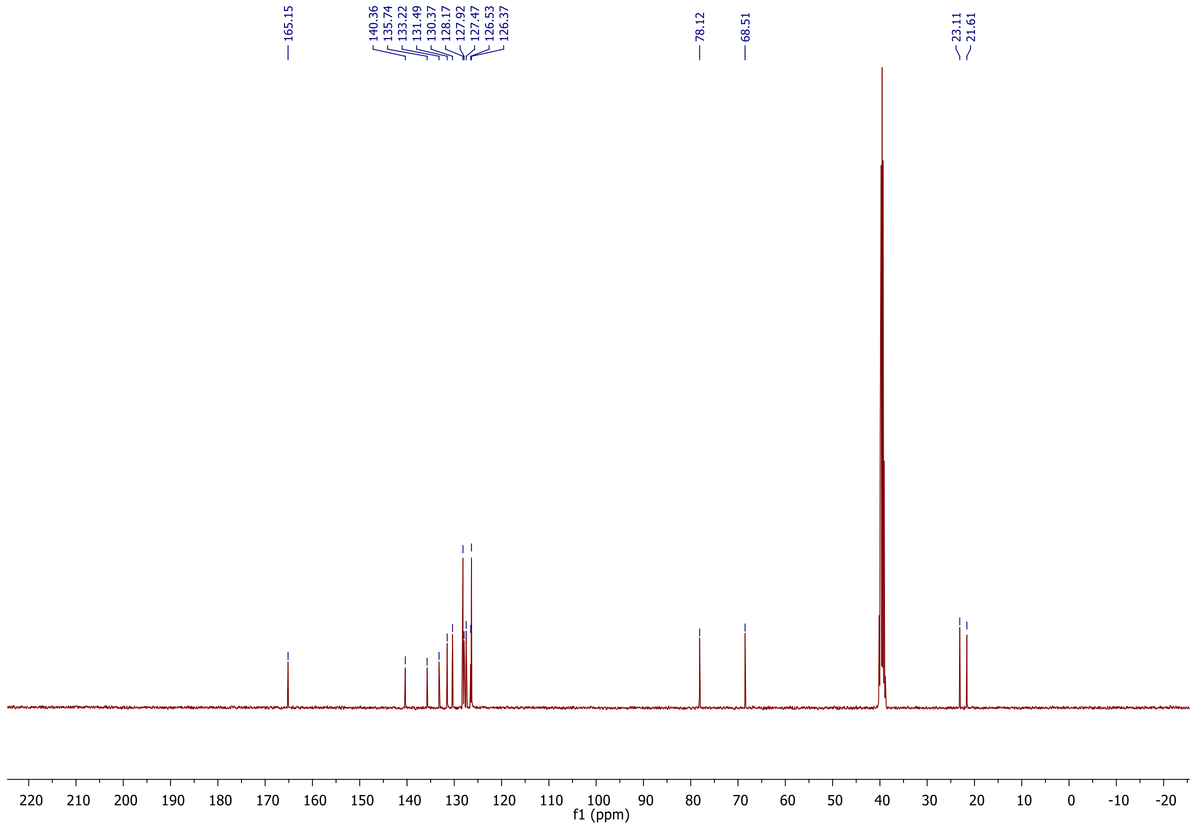

Figure S.18. 4-methoxy-*N*-{phenyl[(propan-2-yl)oxy]methyl}benzamide (1r)

^{1}H NMR (400 MHz, DMSO-d$_6$)

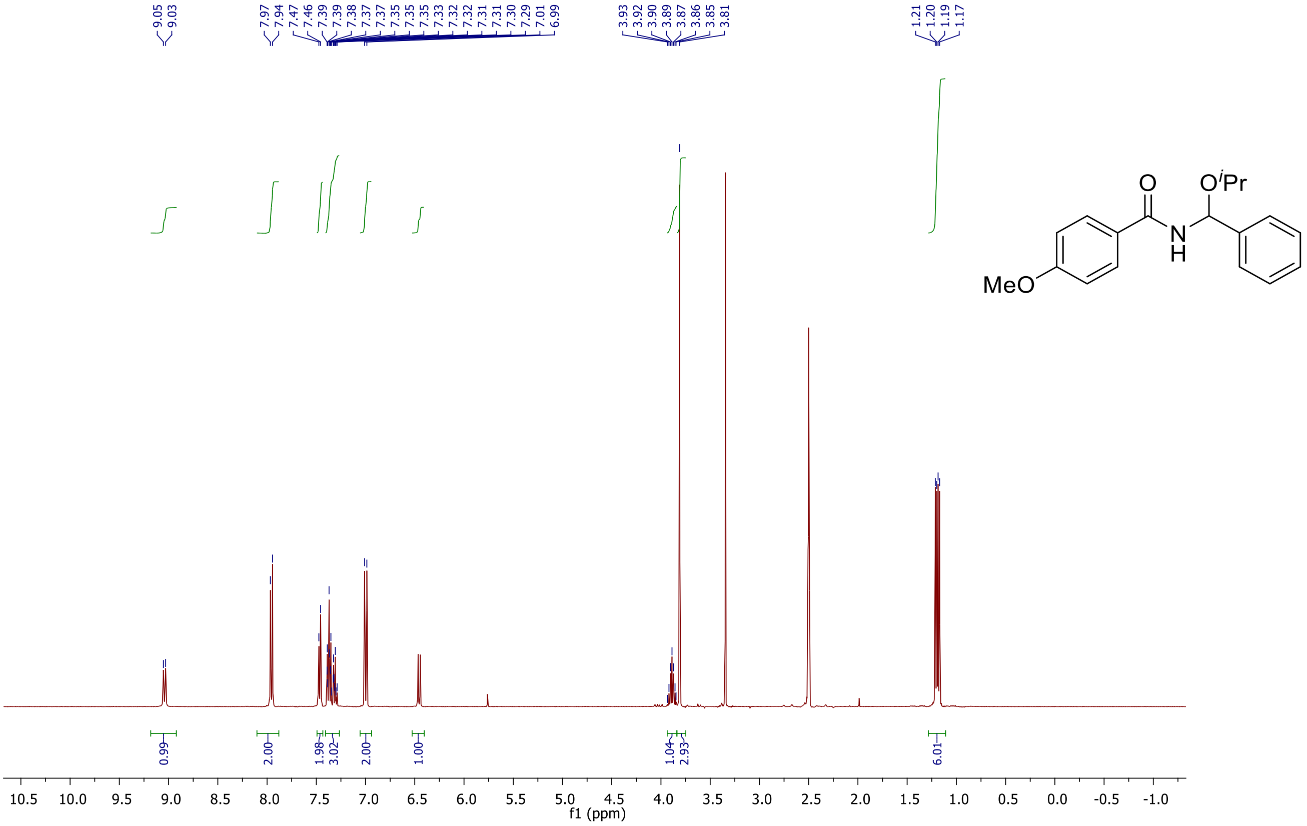

$^{13}C\{^{1}H\}$ NMR (101 MHz, DMSO-d_6)

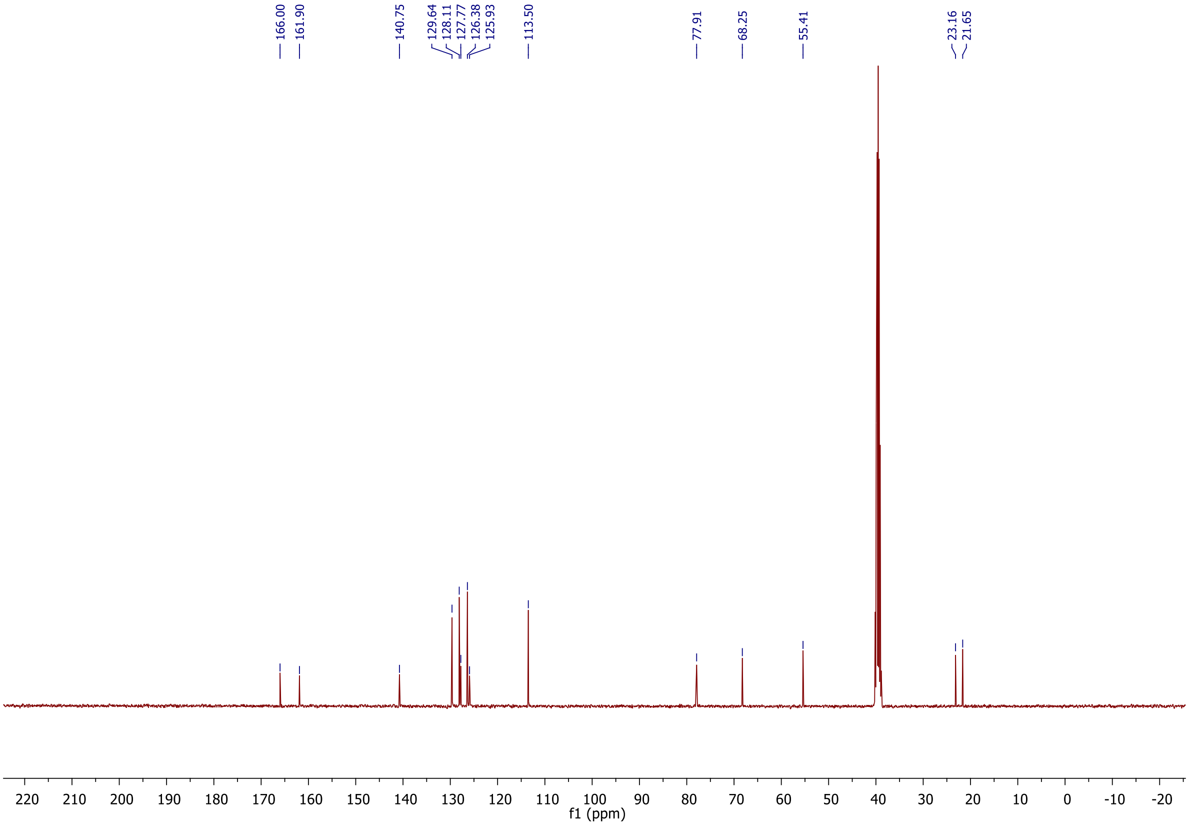

Figure S.19. *N*-{phenyl[(propan-2-yl)oxy]methyl}furan-2-carboxamide (1s)

^{1}H NMR (400 MHz, DMSO-d_6)

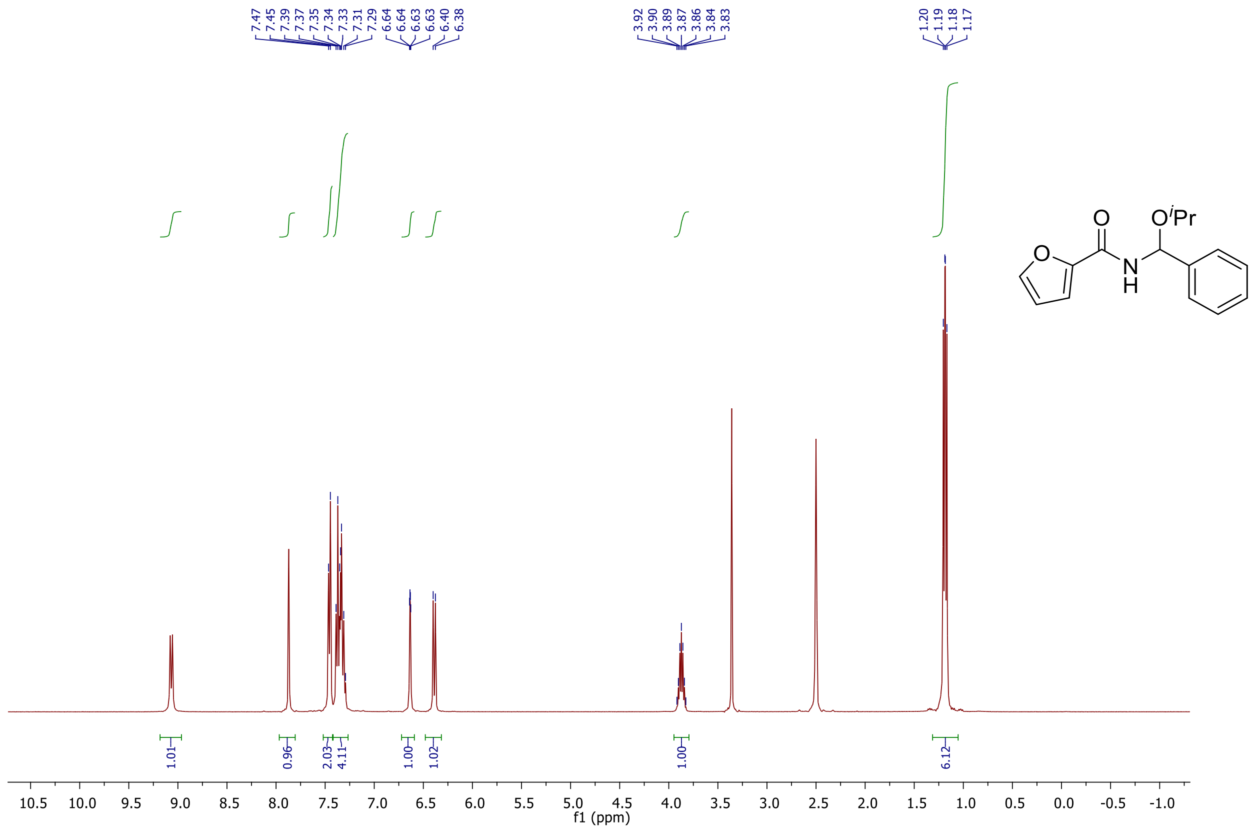

$^{13}C\{^1H\}$ NMR (101 MHz, DMSO-d$_6$)

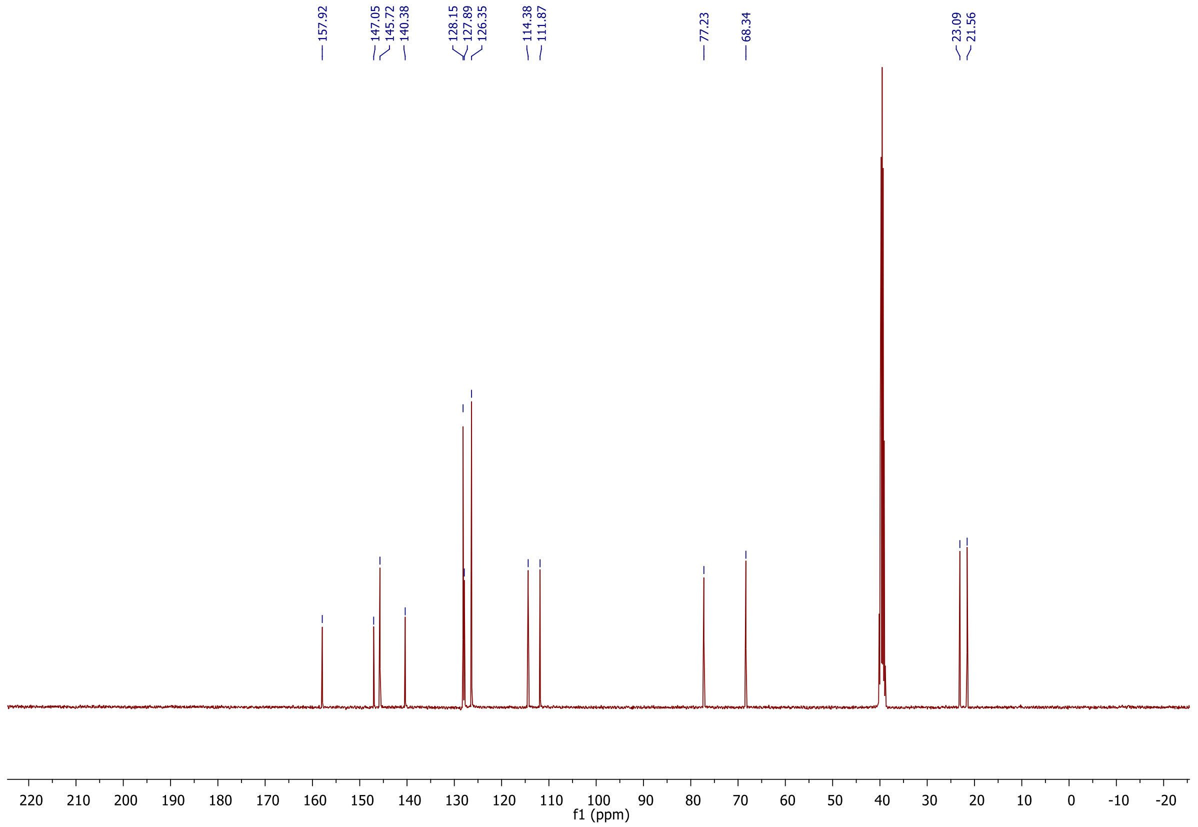

Figure S.20. ***N*-{phenyl[(propan-2-yl)oxy]methyl}thiophene-2-carboxamide (1t)**

^{1}H NMR (400 MHz, DMSO-d$_6$)

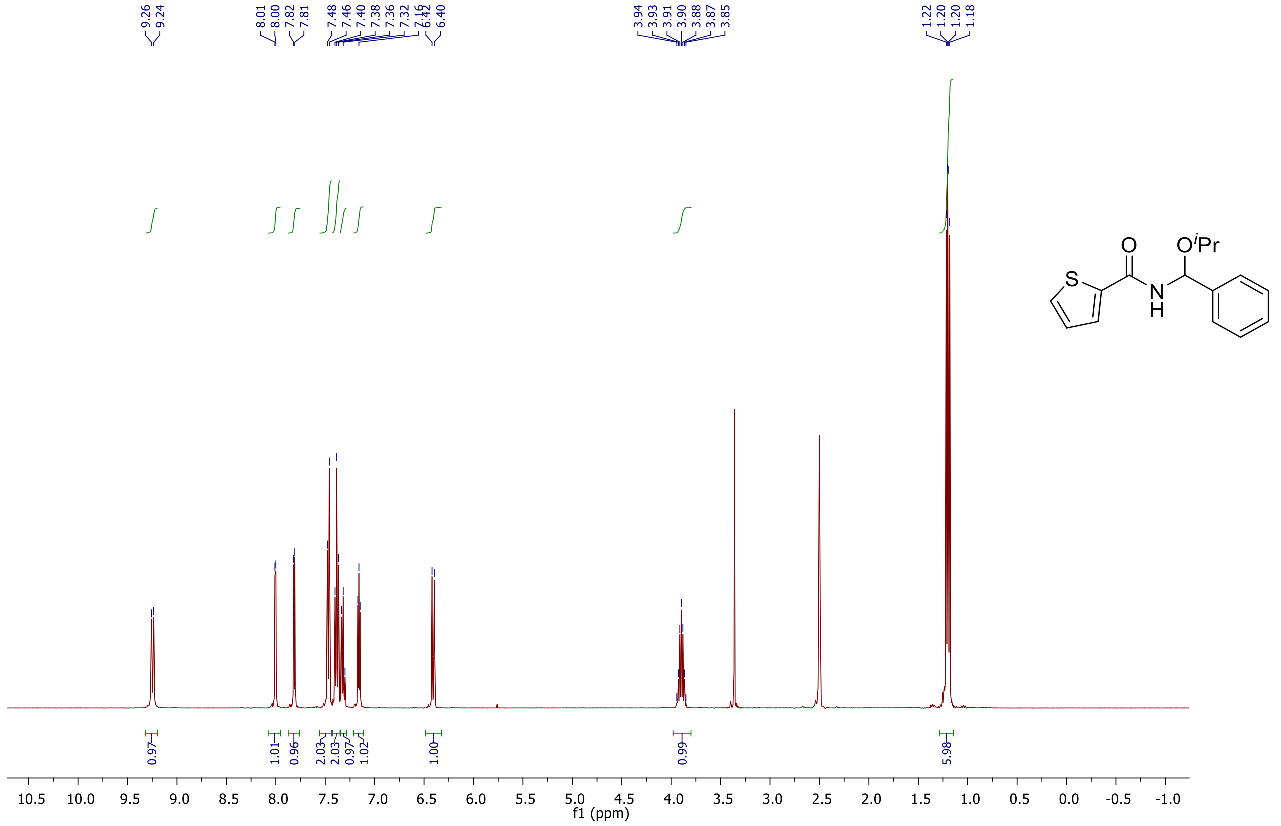

$^{13}C\{^{1}H\}$ NMR (101 MHz, DMSO-d$_6$)

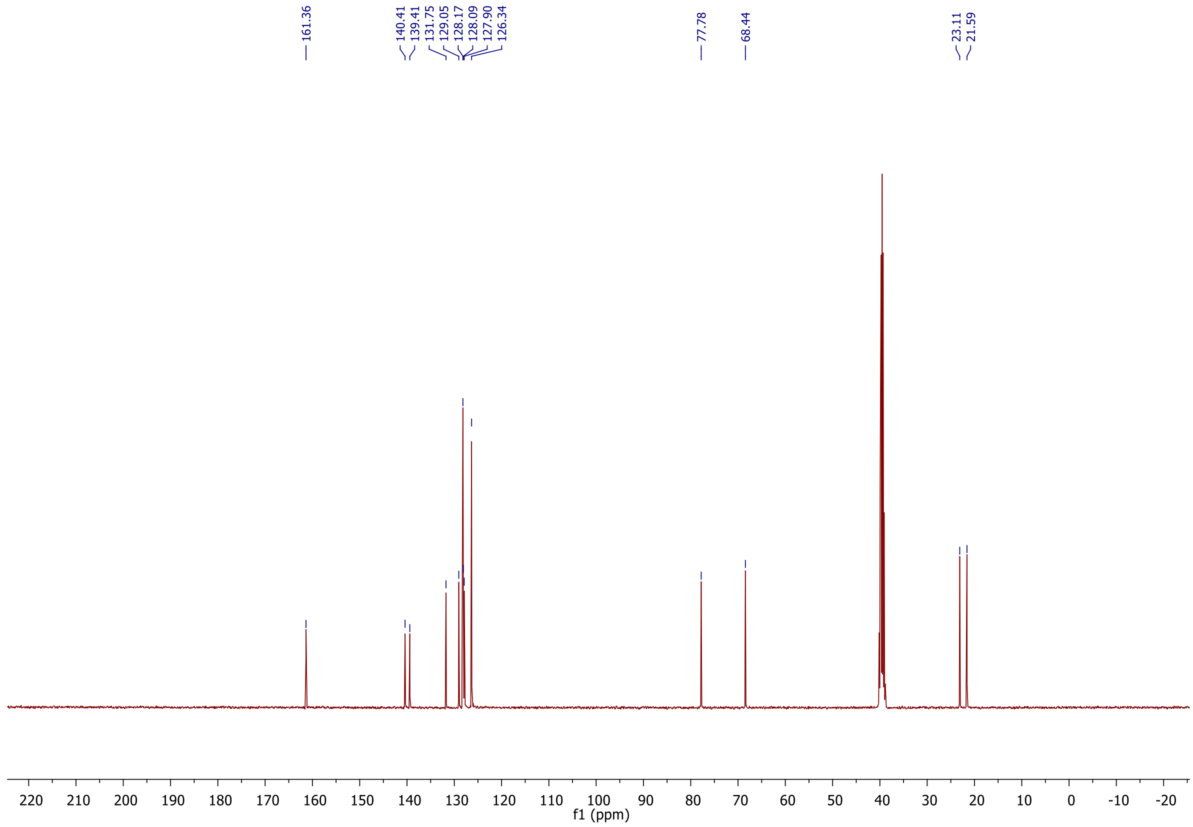

Figure S.21. 2-methyl-*N*-{phenyl[(propan-2-yl)oxy]methyl}benzamide (1u)

^{1}H NMR (400 MHz, DMSO-d$_6$)

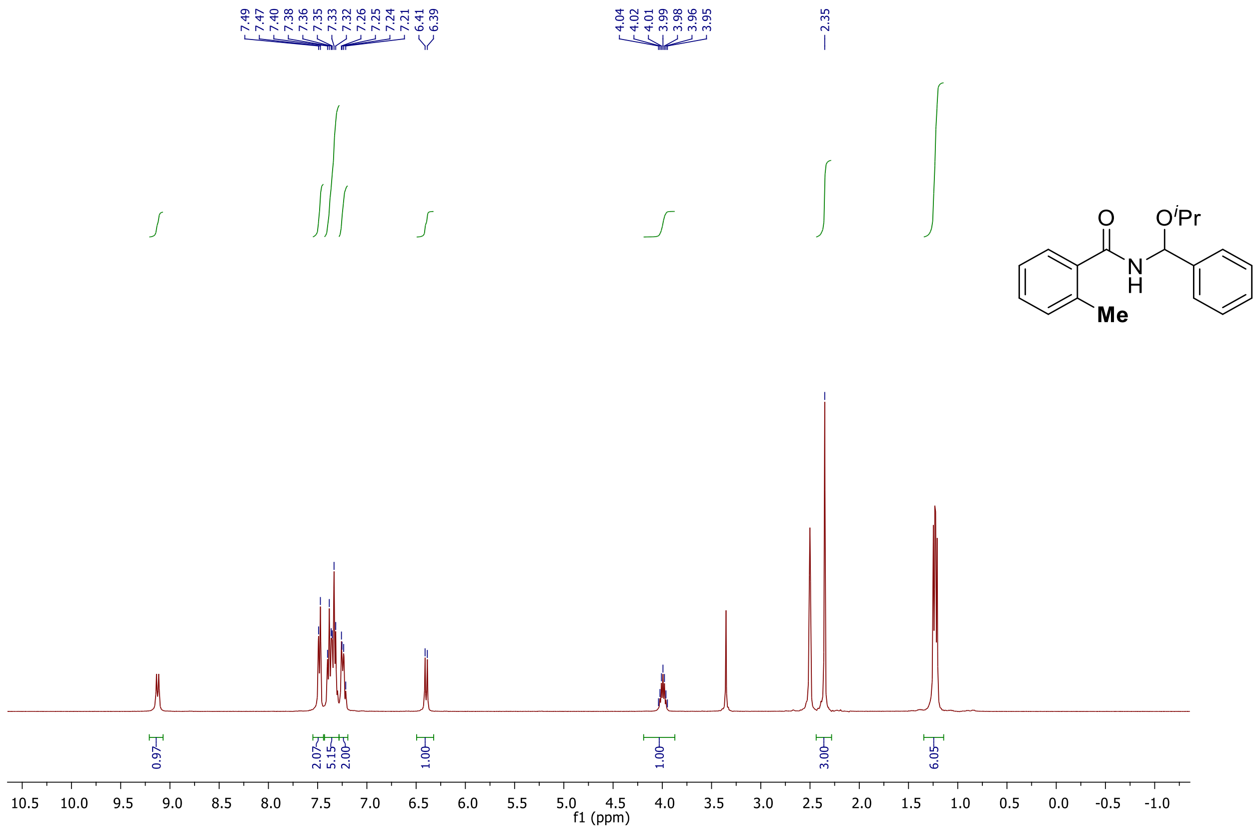

$^{13}C\{^{1}H\}$ NMR (101 MHz, DMSO-d$_6$)

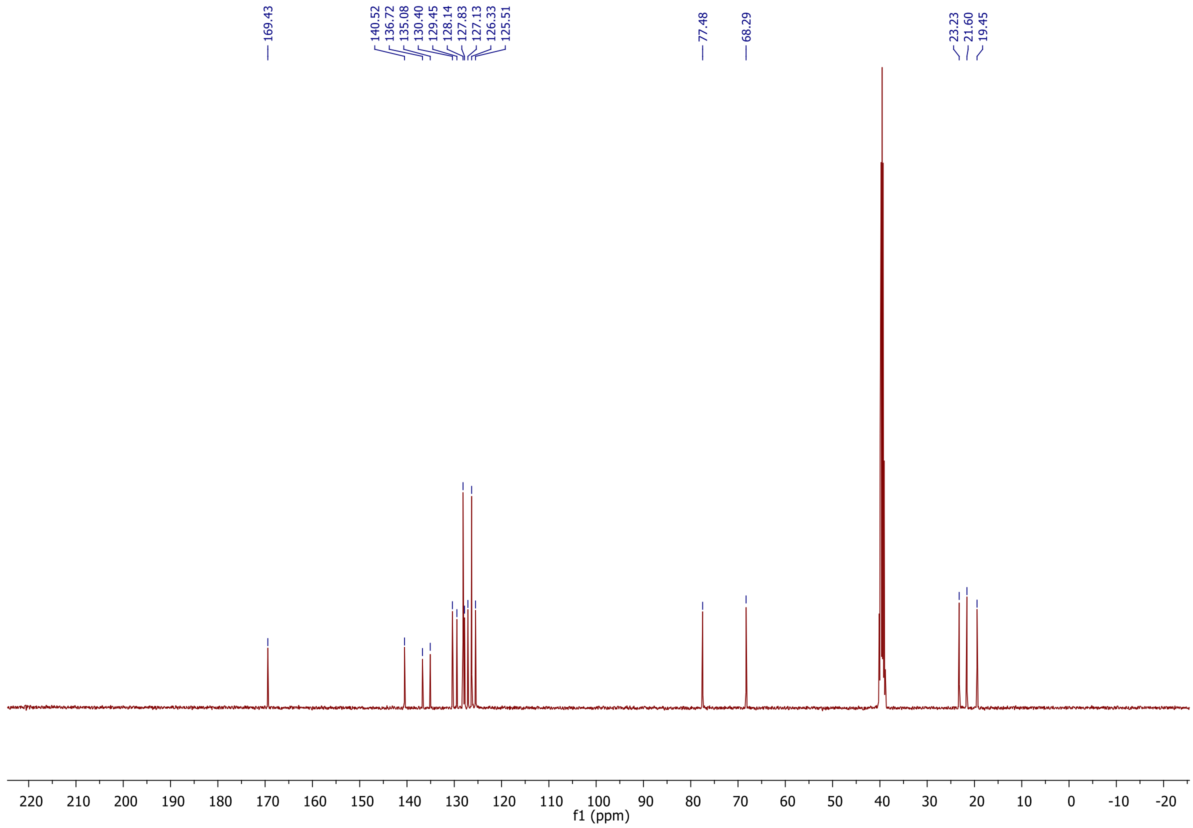

Figure S.22. *N*-[methoxy(phenyl)methyl]benzenecarbothioamide (5a)

^{1}H NMR (400 MHz, DMSO-d$_6$)

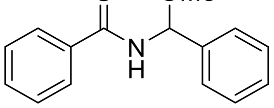

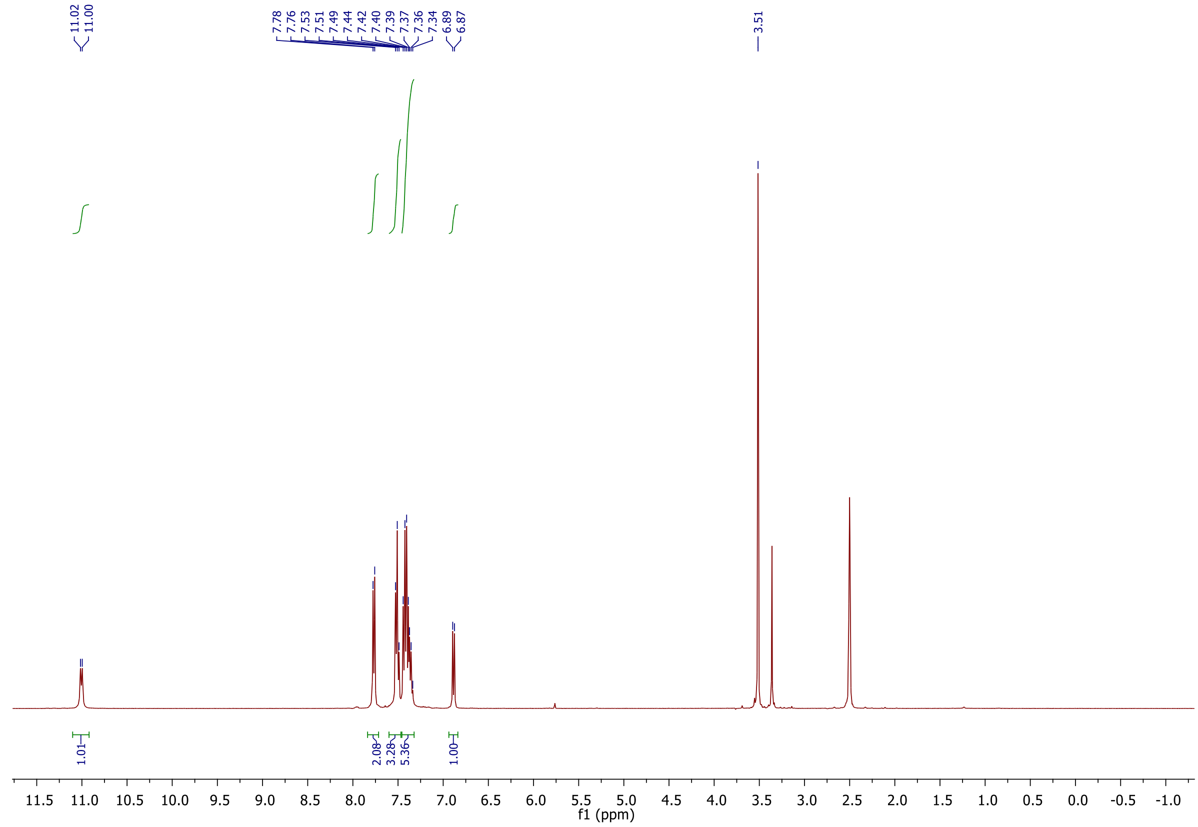

$^{13}C\{^{1}H\}$ NMR (101 MHz, DMSO-d6)

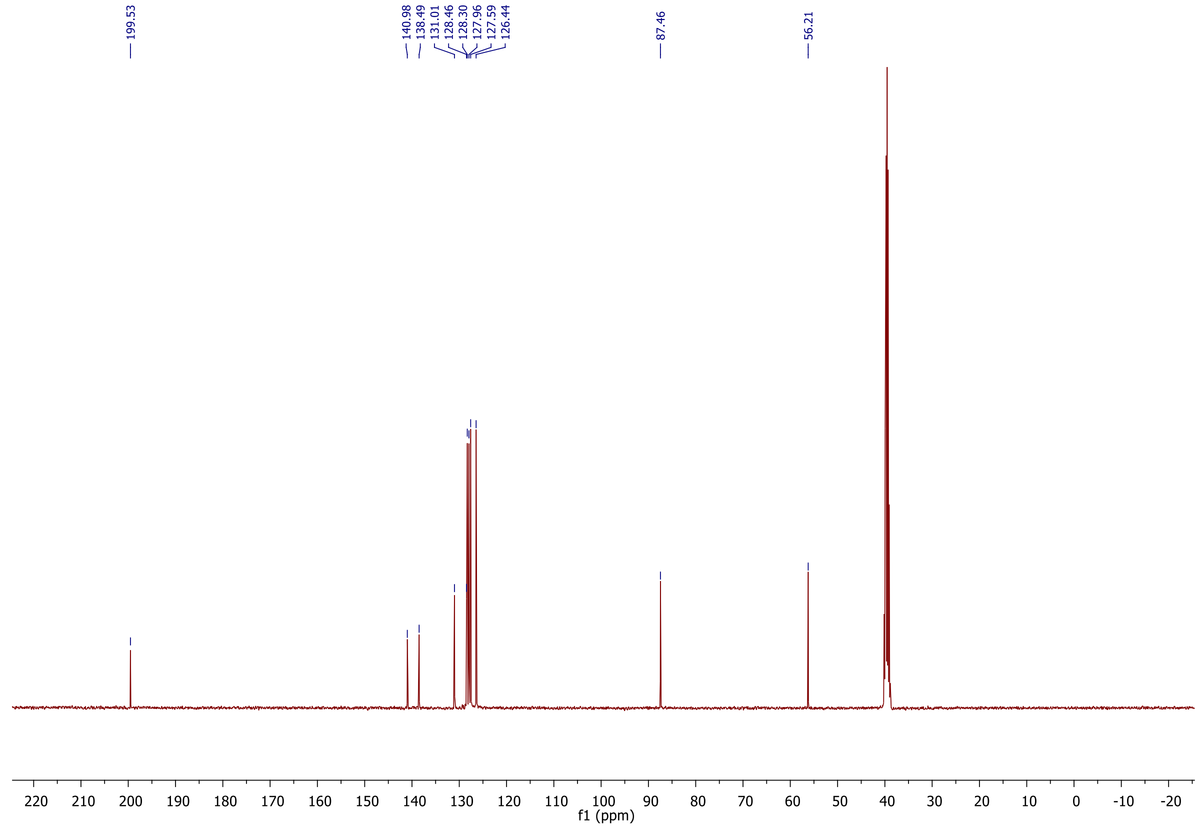

Figure S.23. *N*-[(4-bromophenyl)(methoxy)methyl]benzenecarbothioamide (5b)

^{1}H NMR (400 MHz, DMSO-d6)

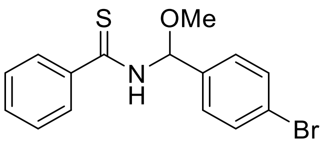

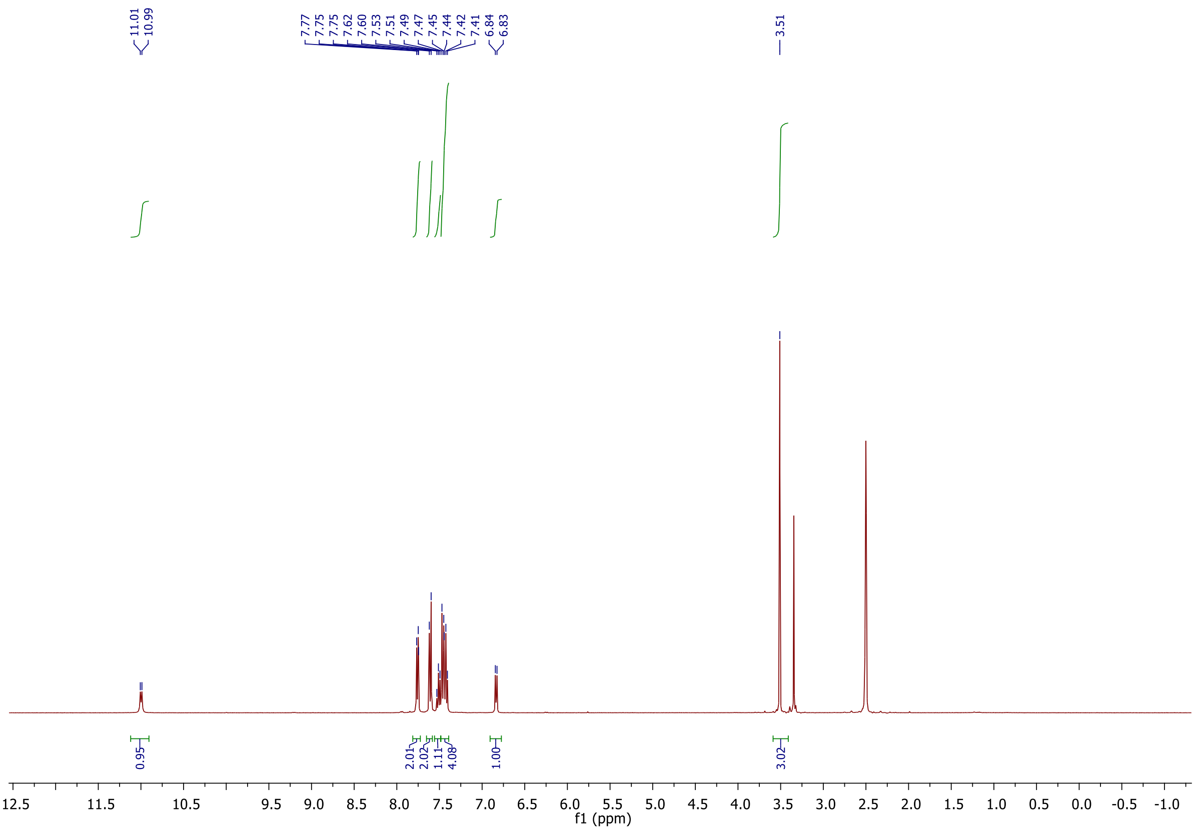

$^{3}C\{^{1}H\}$ NMR (101 MHz, DMSO-d_6)

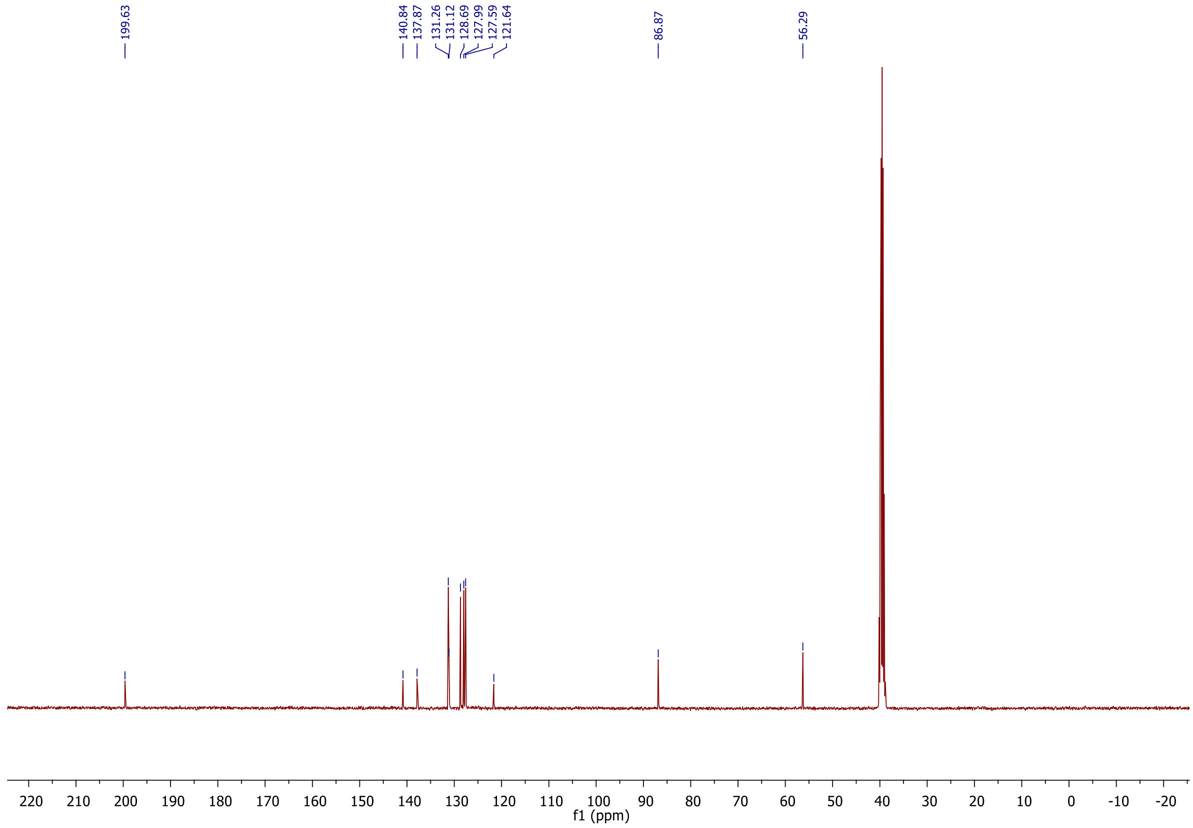

Figure S.24. 4-bromo-*N*-[methoxy(phenyl)methyl]benzene-1-carbothioamide (5c)

^{1}H NMR (400 MHz, DMSO-d_6)

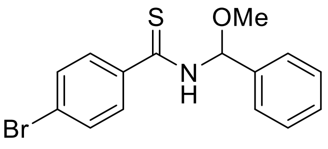

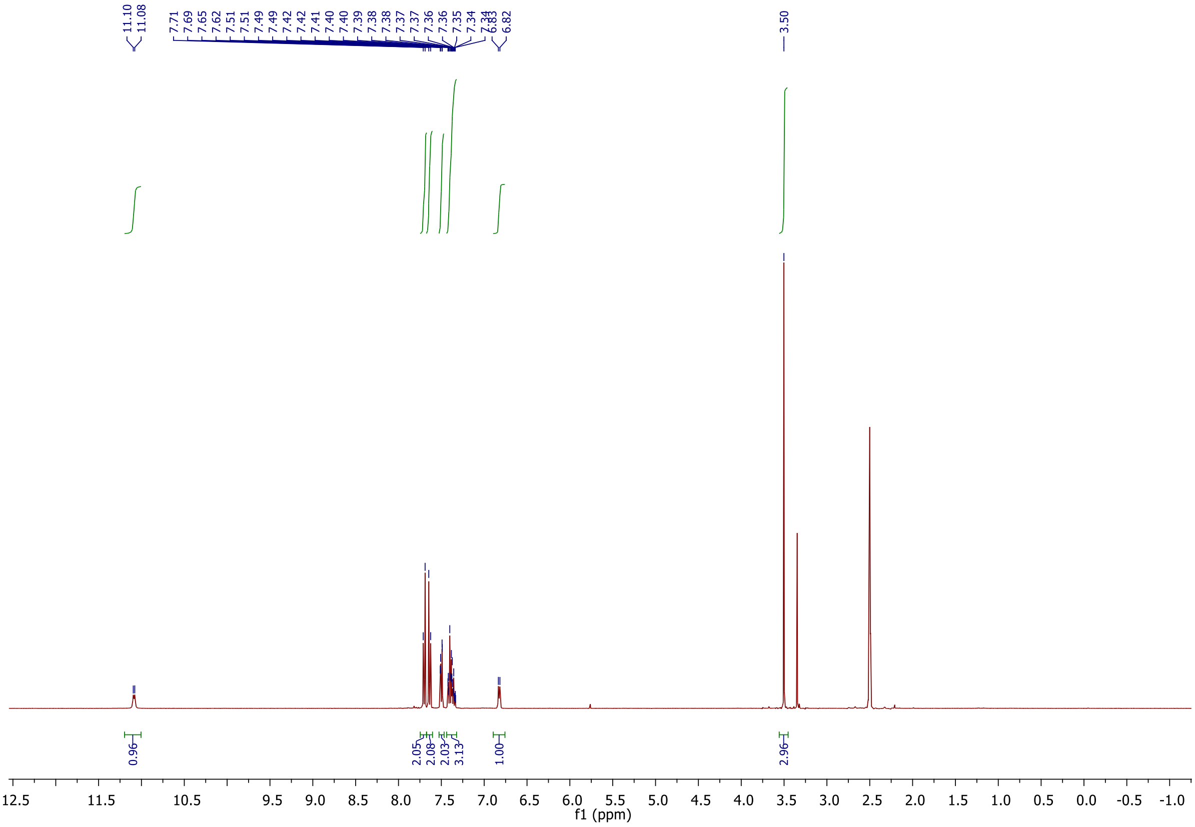

$^{13}C\{^{1}H\}$ NMR (101 MHz, DMSO-d6)

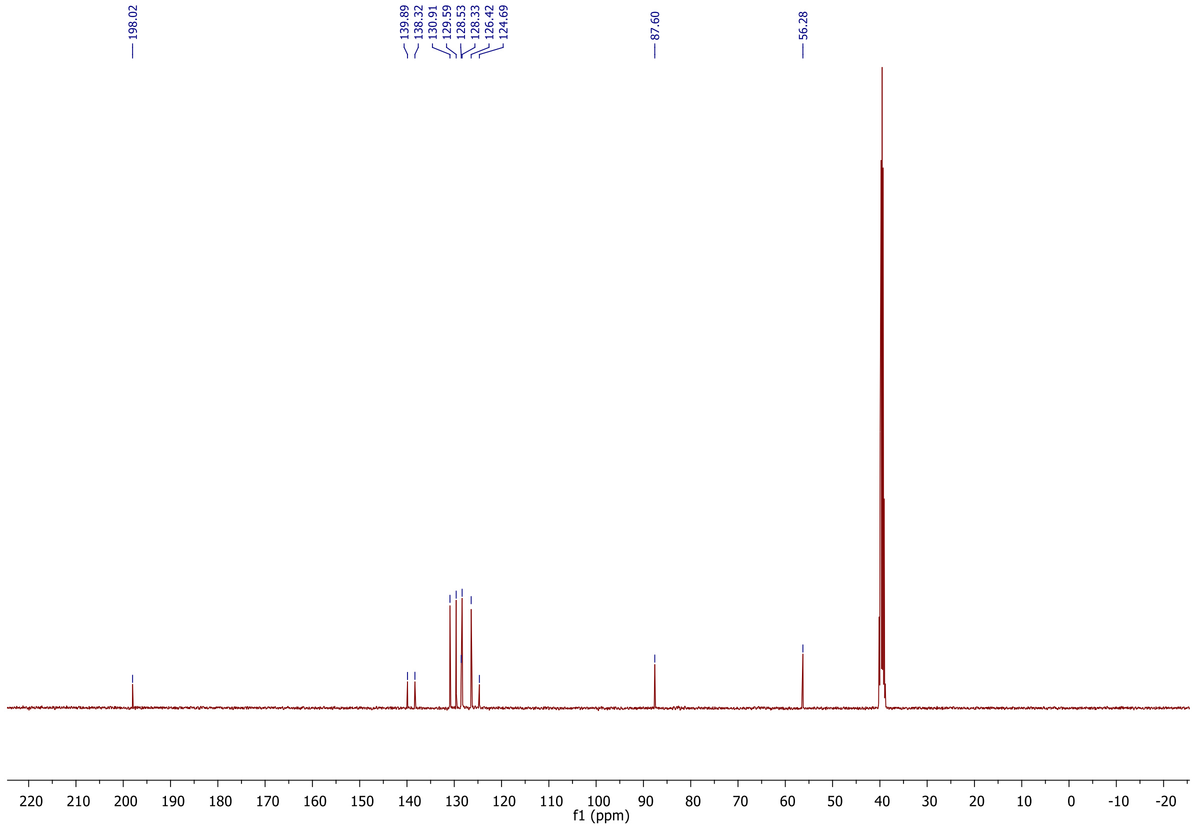

Figure S.25. ***N*-(*tert*-butyl)-2,4-diphenyloxazol-5-amine (2a)**

^{1}H NMR (400 MHz, DMSO-d6)

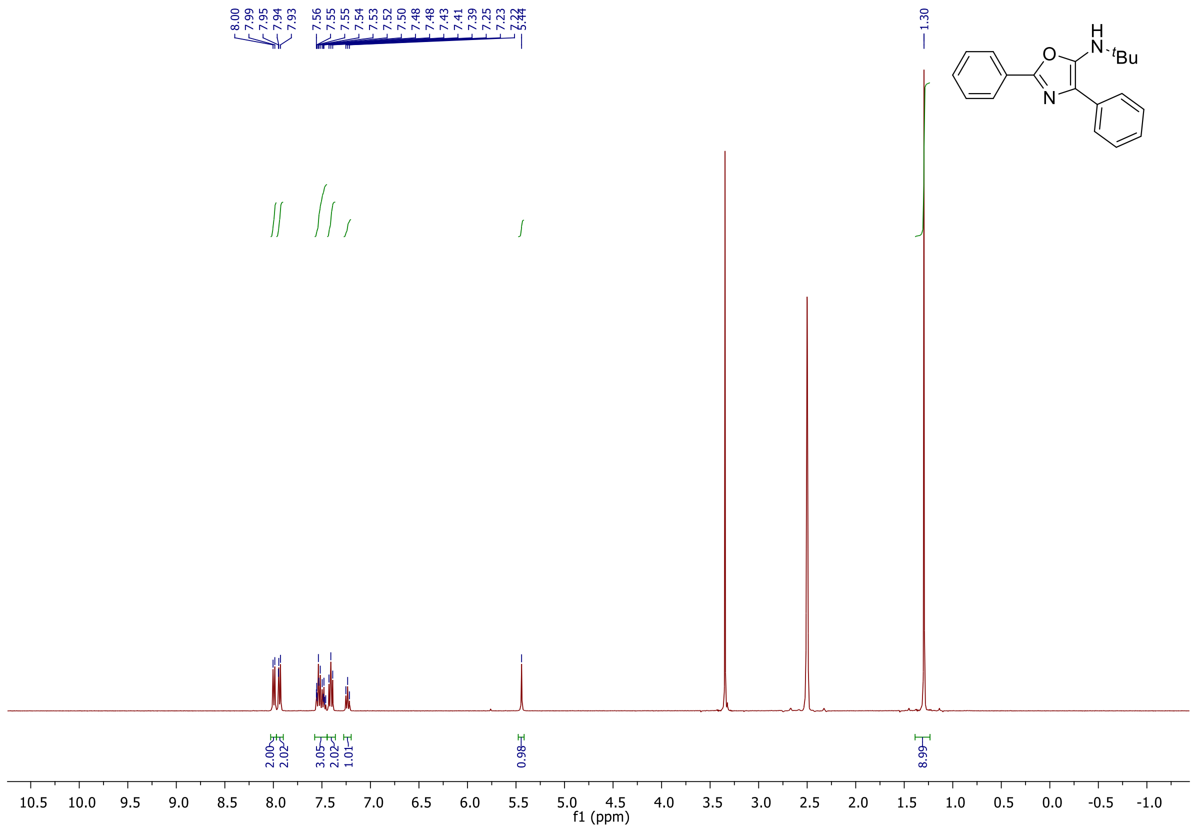

$^{13}C\{^{1}H\}$ NMR (101 MHz, DMSO-d_6)

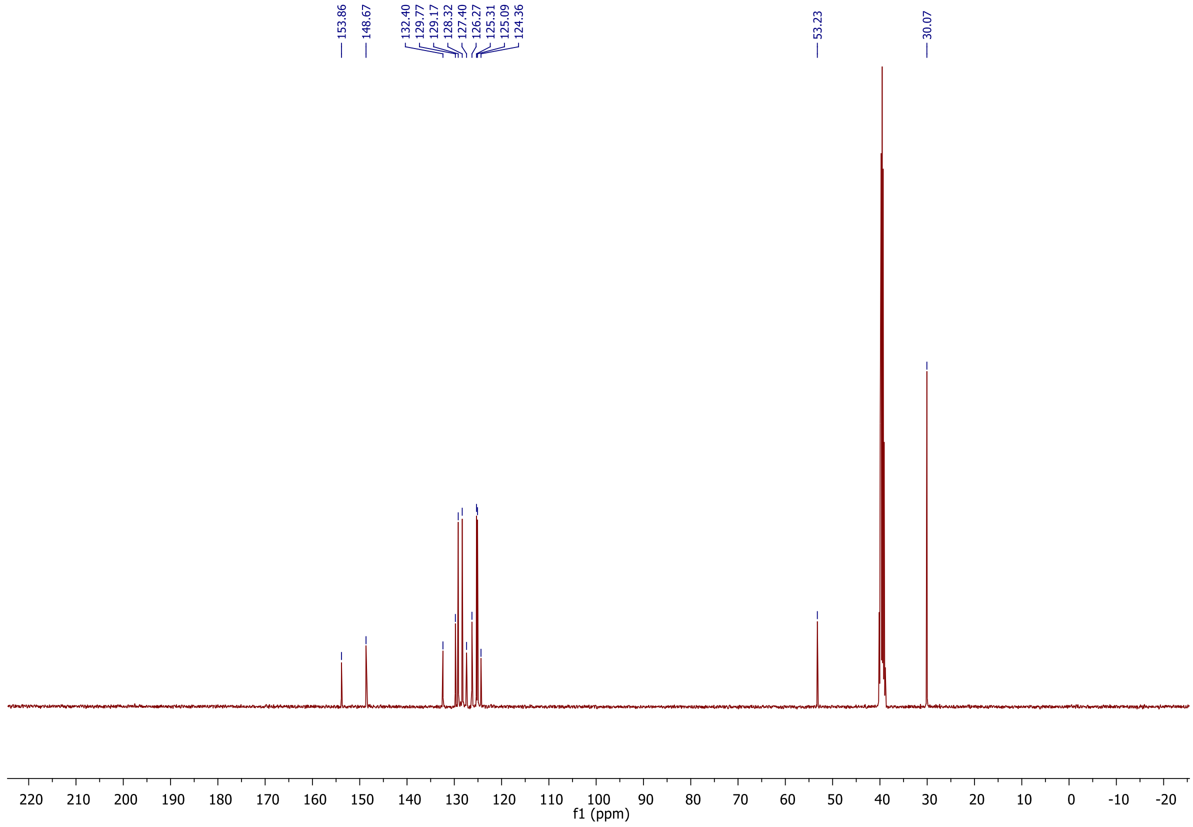

Figure S.26. 4-(4-bromophenyl)-*N*-(*tert*-butyl)-2-phenyloxazol-5-amine (2b)

^1H NMR (400 MHz, DMSO-d$_6$)

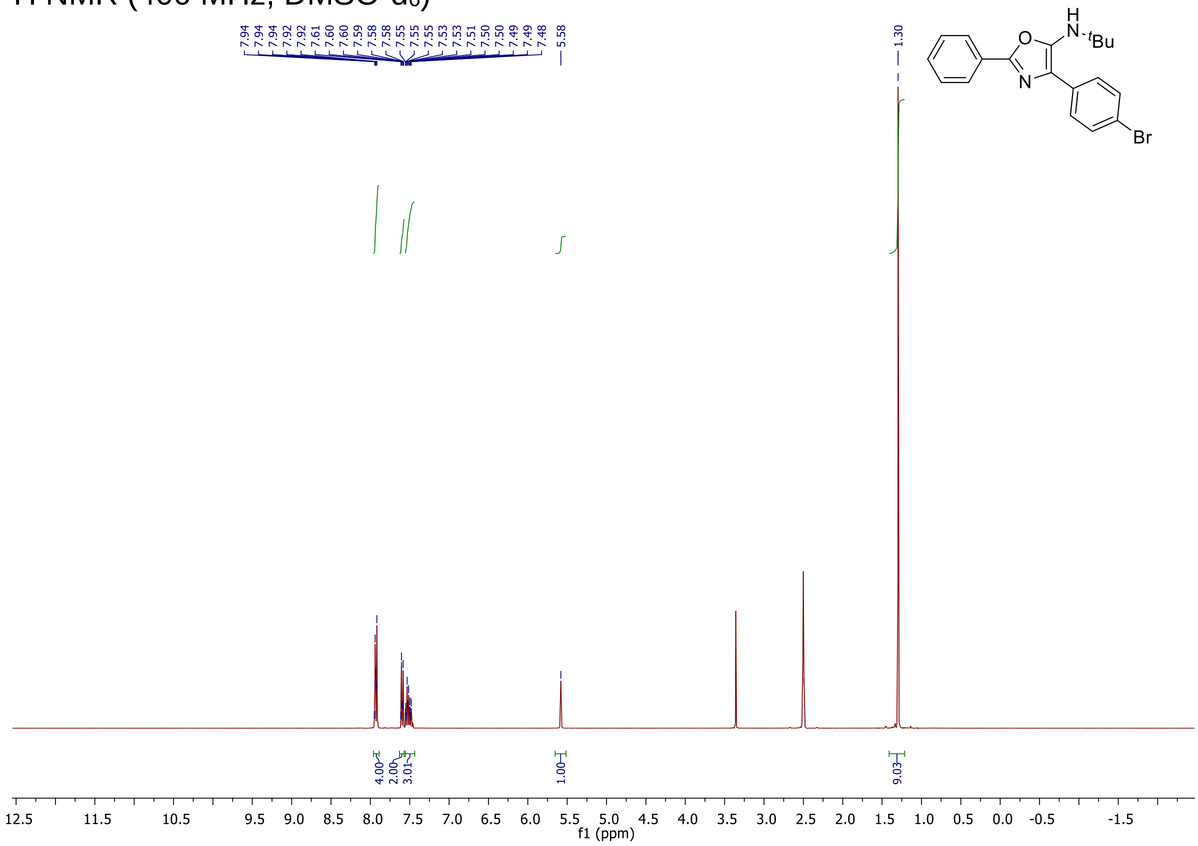

$^{13}C\{^1H\}$ NMR (101 MHz, DMSO-d$_6$)

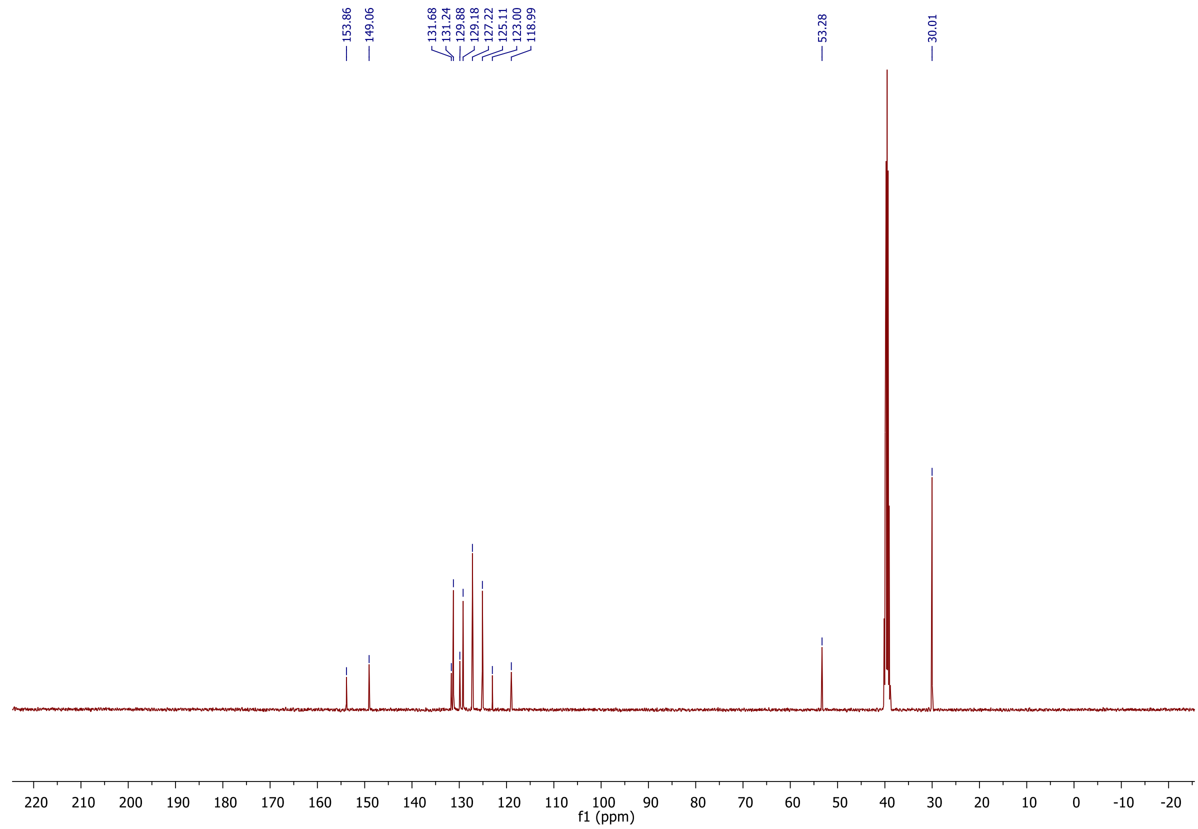

Figure S.27. ***N-tert*-butyl-2-phenyl-4-[4-(trifluoromethyl)phenyl]-1,3-oxazol-5-amine (2c)**

^{1}H NMR (400 MHz, DMSO-d$_6$)

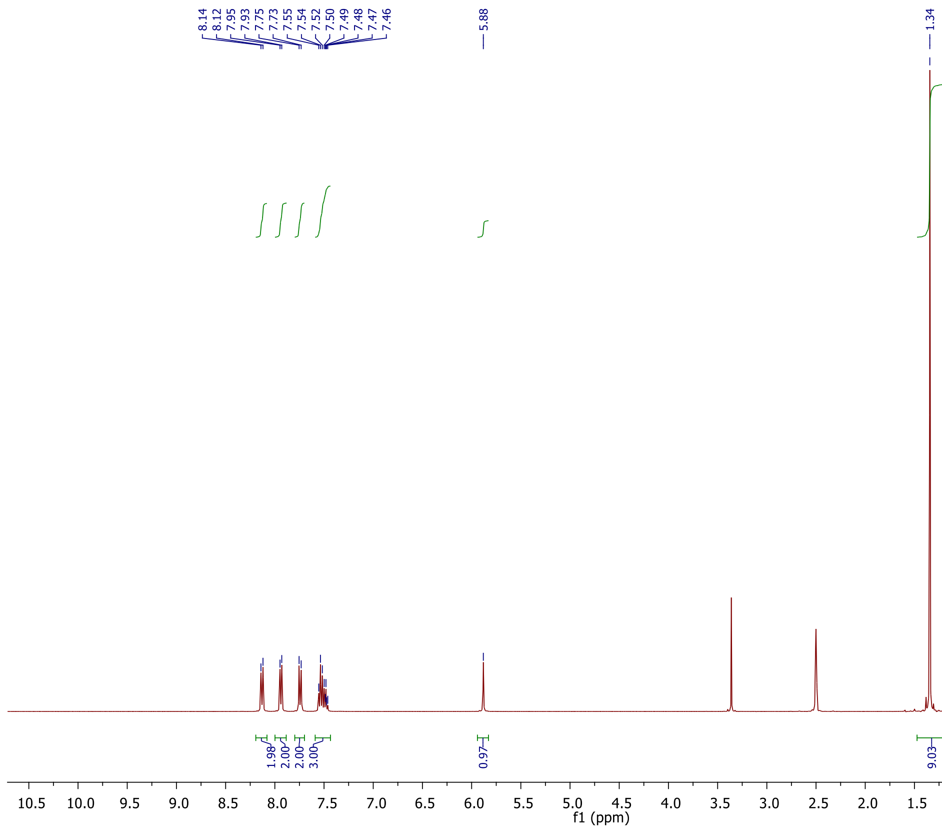

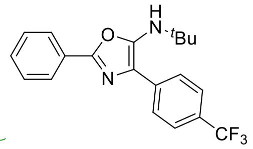

$^{13}C\{^{1}H\}$ NMR (101 MHz, DMSO-d$_6$)

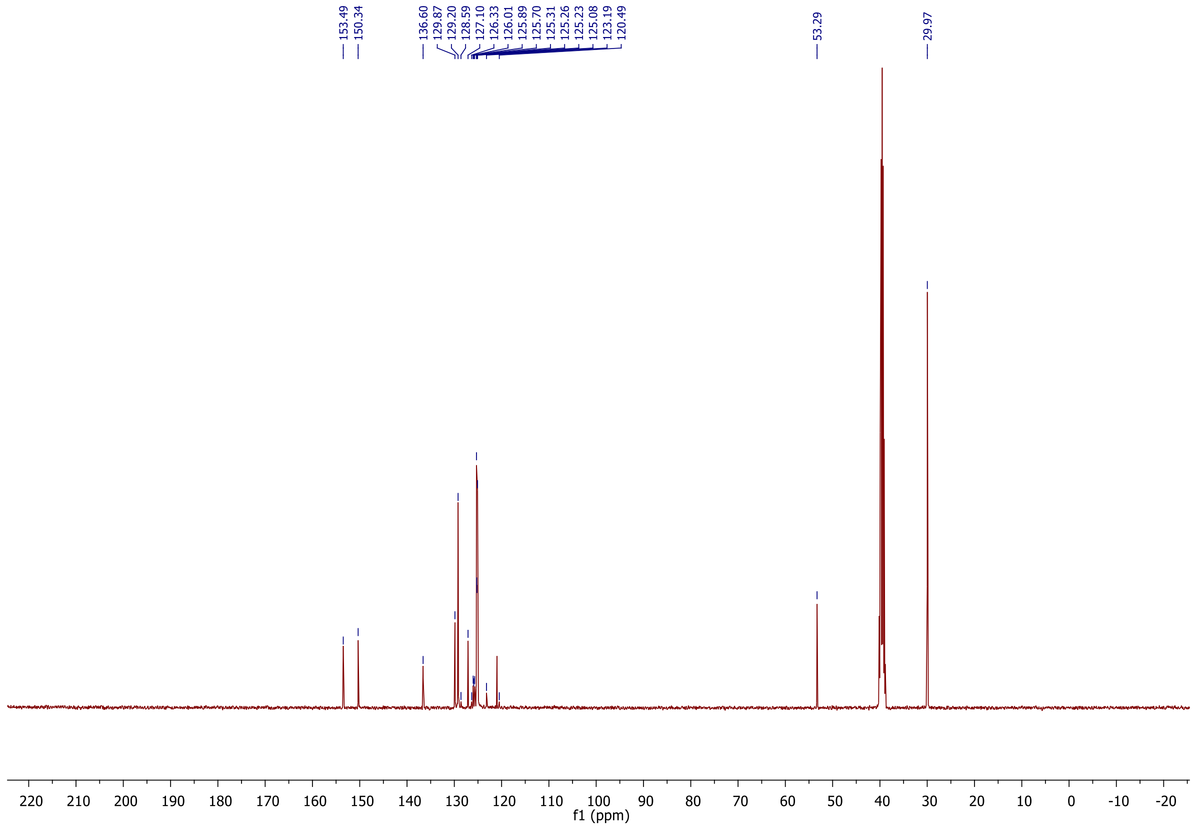

Figure S.28. ***N-tert*-butyl-4-(4-chlorophenyl)-2-phenyl-1,3-oxazol-5-amine (2d)**

^{1}H NMR (400 MHz, DMSO-d_6)

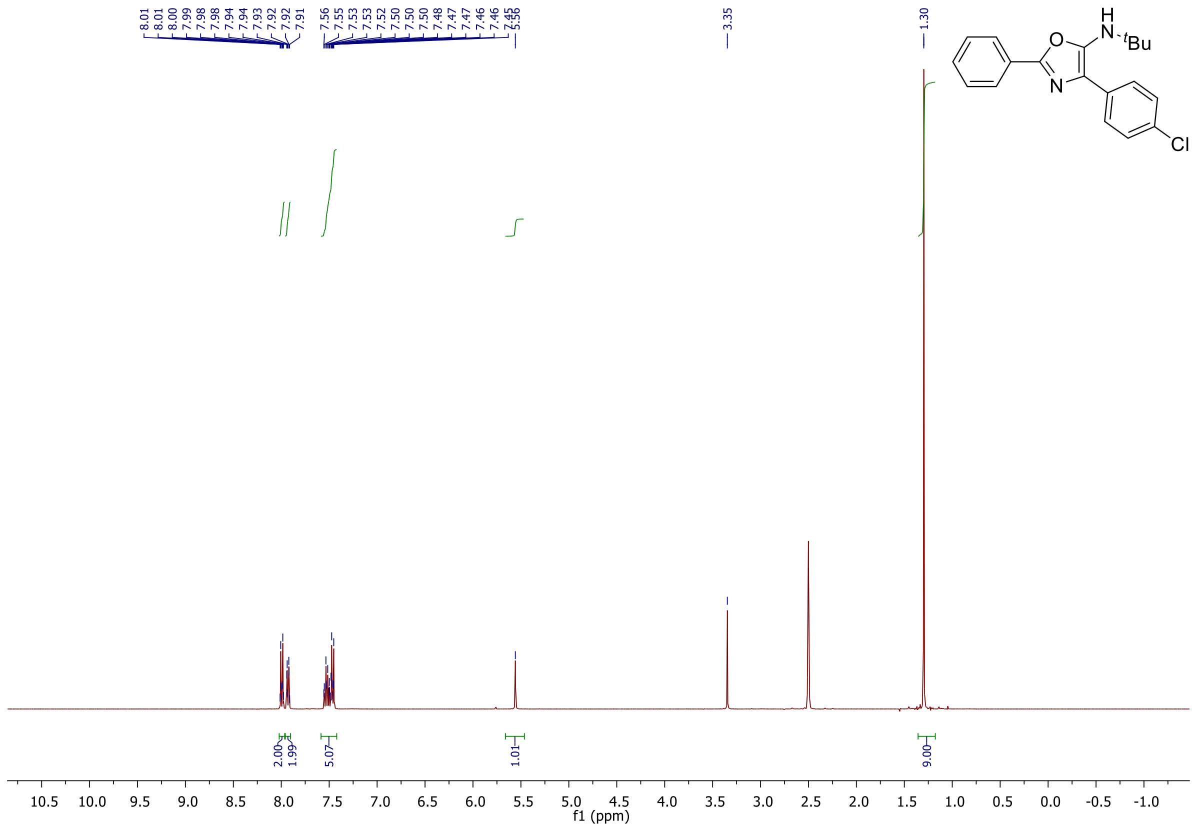

^{13}C{^{1}H} NMR (101 MHz, DMSO-d_6)

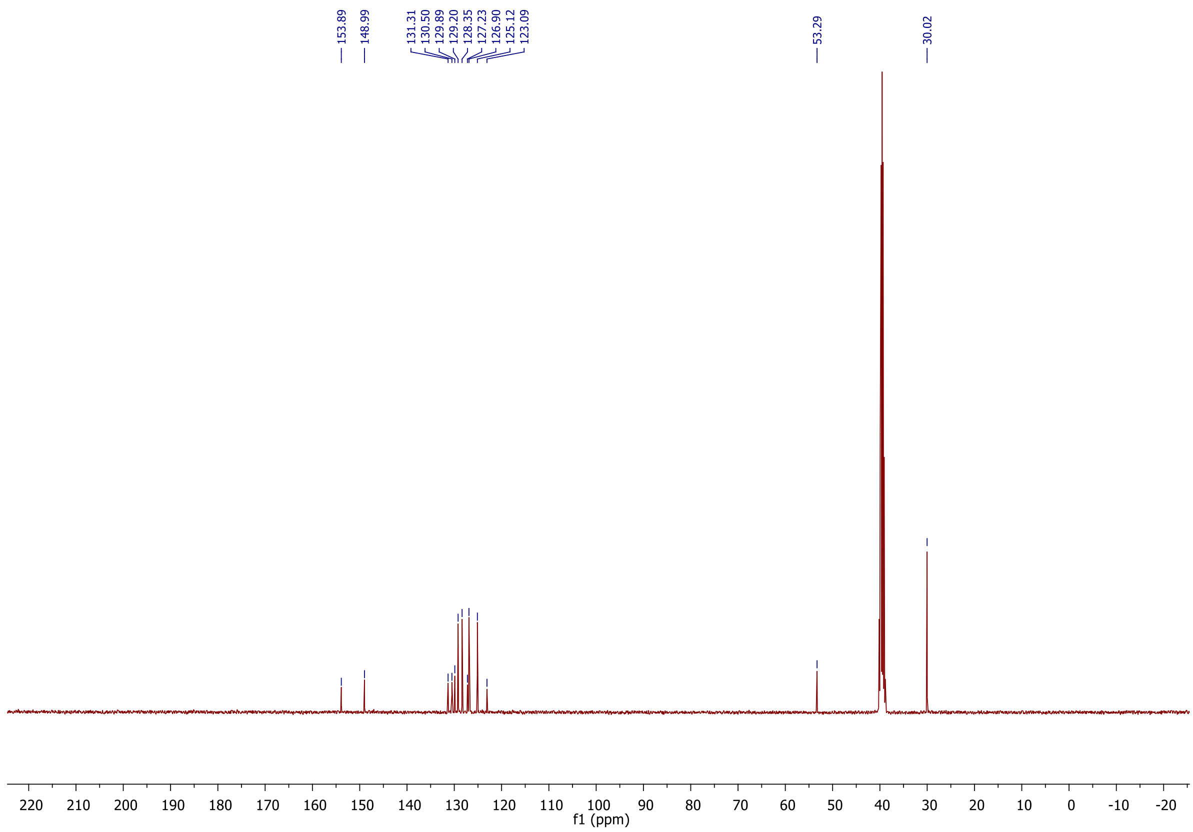

Figure S.29. *N*-tert-butyl-4-(4-nitrophenyl)-2-phenyl-1,3-oxazol-5-amine (2e)

^{1}H NMR (400 MHz, DMSO-d$_6$)

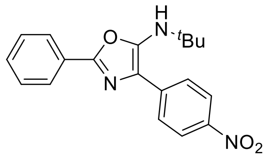

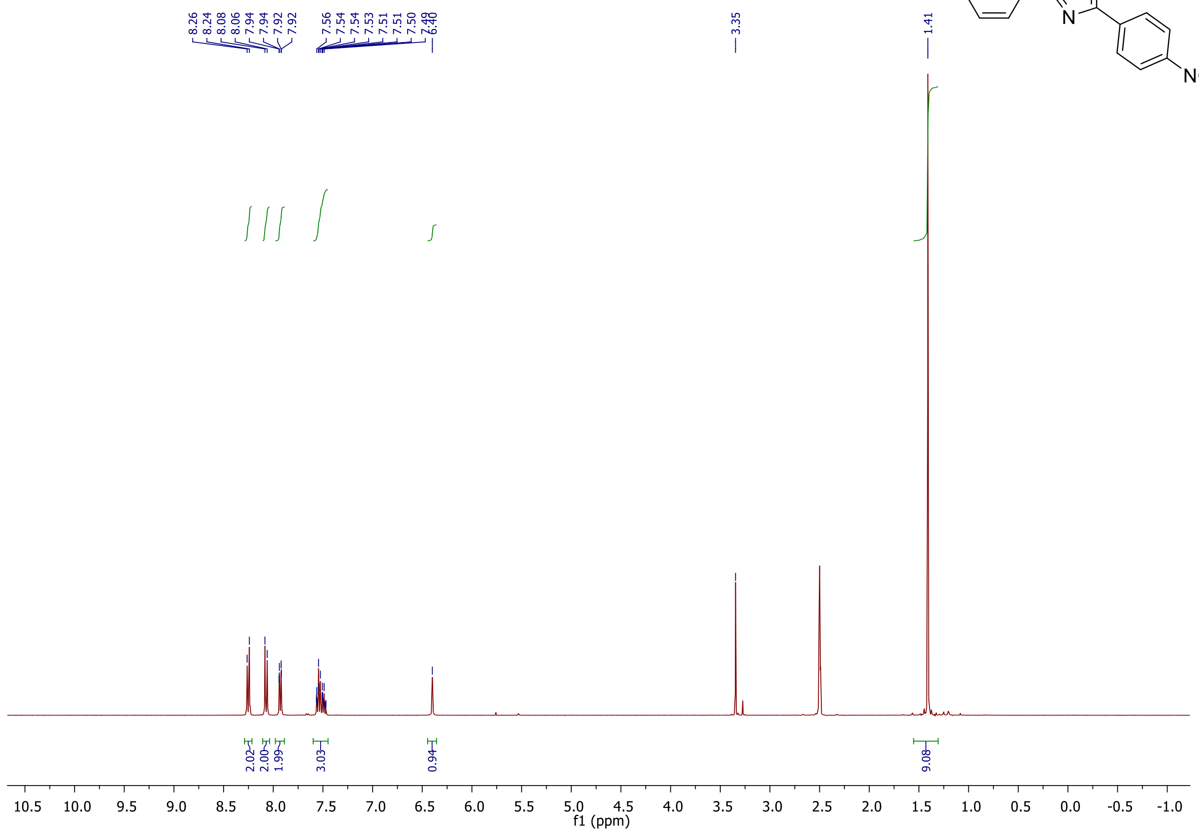

^{13}C{^{1}H} NMR (101 MHz, DMSO-d$_6$)

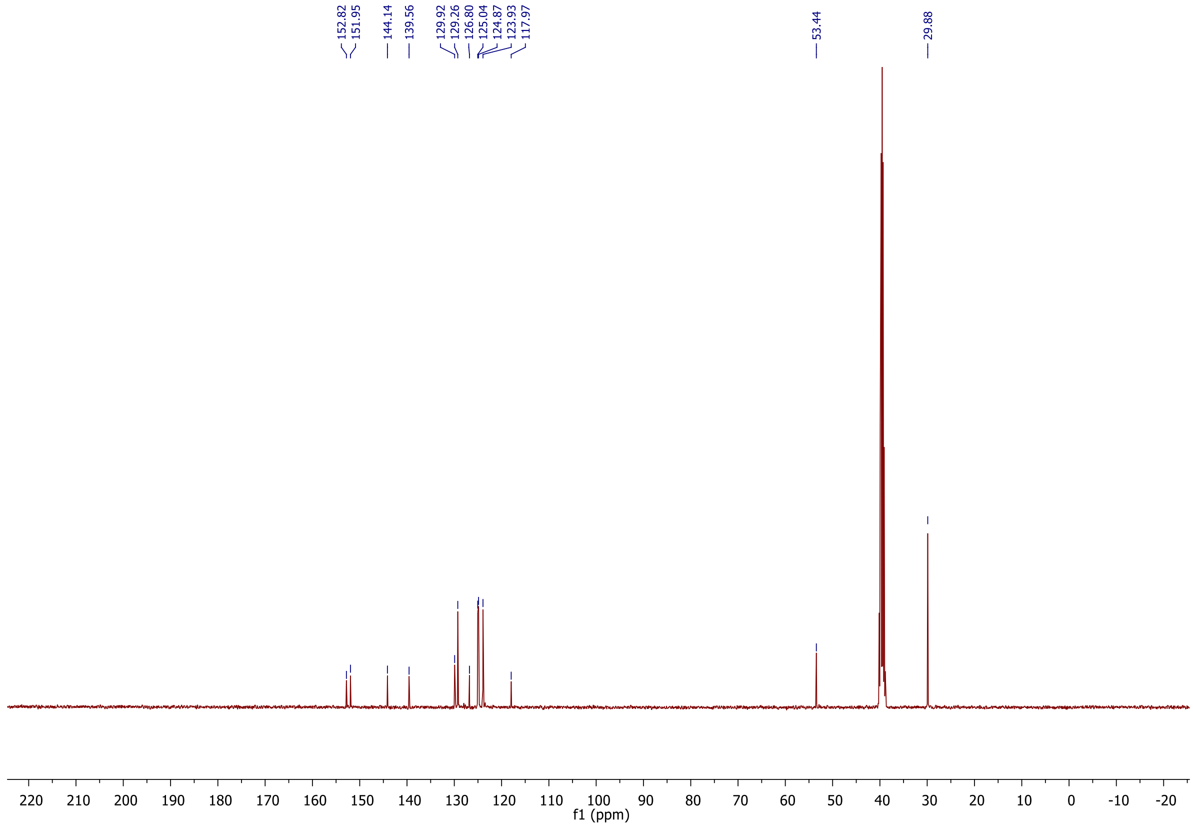

Figure S.30. 4-[5-(*tert*-butylamino)-2-phenyl-1,3-oxazol-4-yl]benzonitrile (2f)

^{1}H NMR (400 MHz, DMSO-d$_6$)

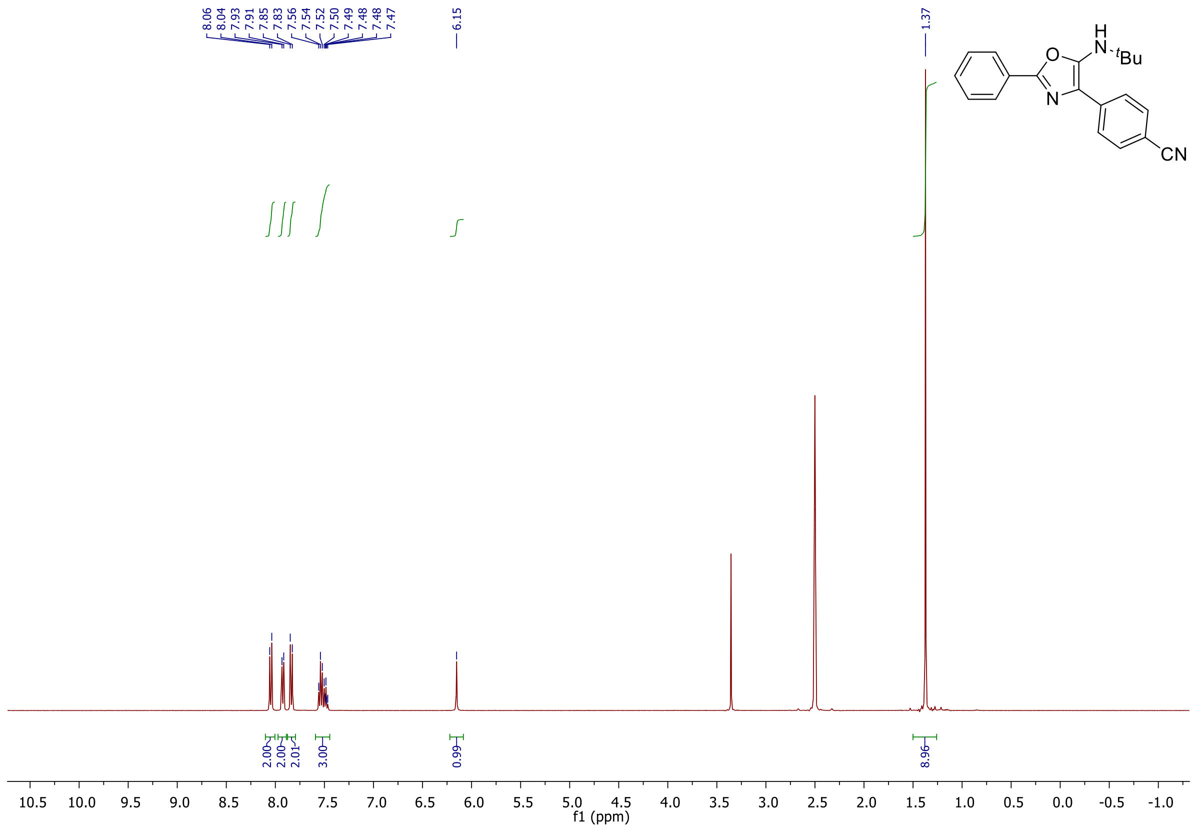

^{13}C{^{1}H} NMR (101 MHz, DMSO-d$_6$)

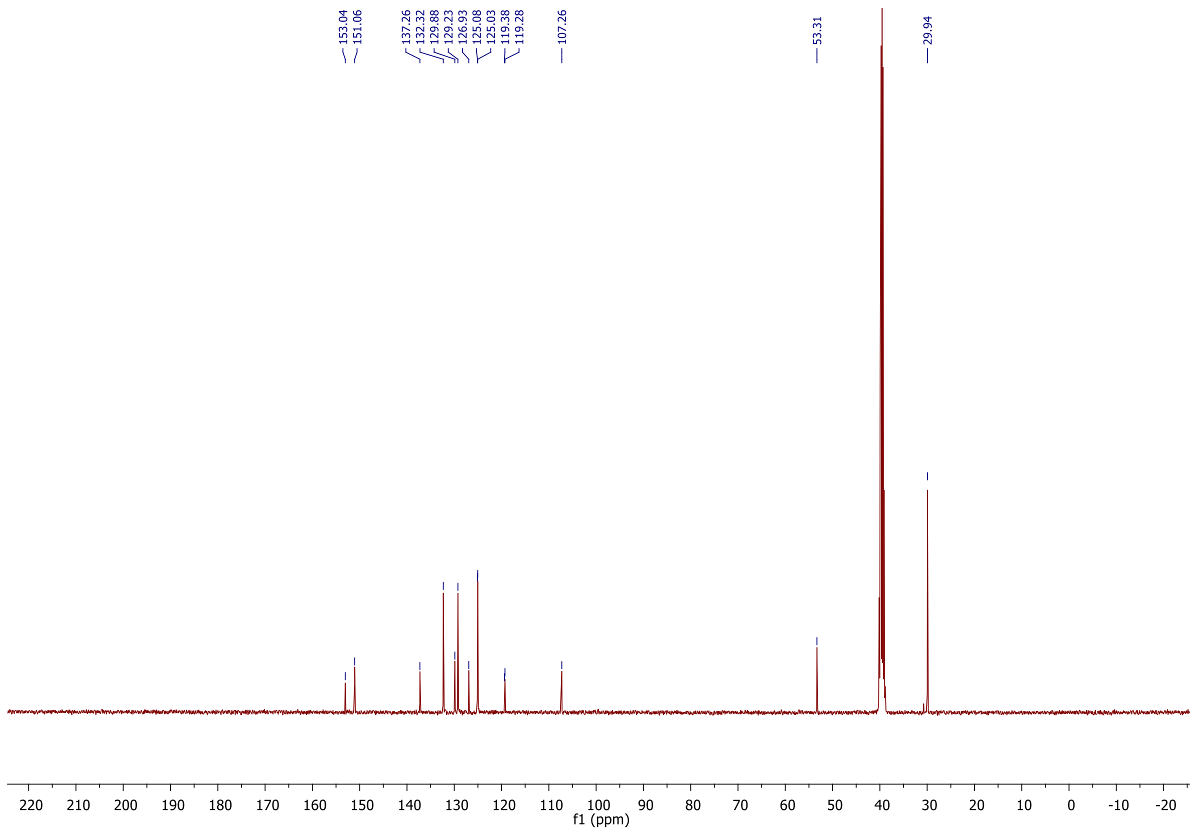

Figure S.31. 3-[5-(*tert*-butylamino)-2-phenyl-1,3-oxazol-4-yl]benzonitrile (2g)

^{1}H NMR (400 MHz, DMSO-d$_6$)

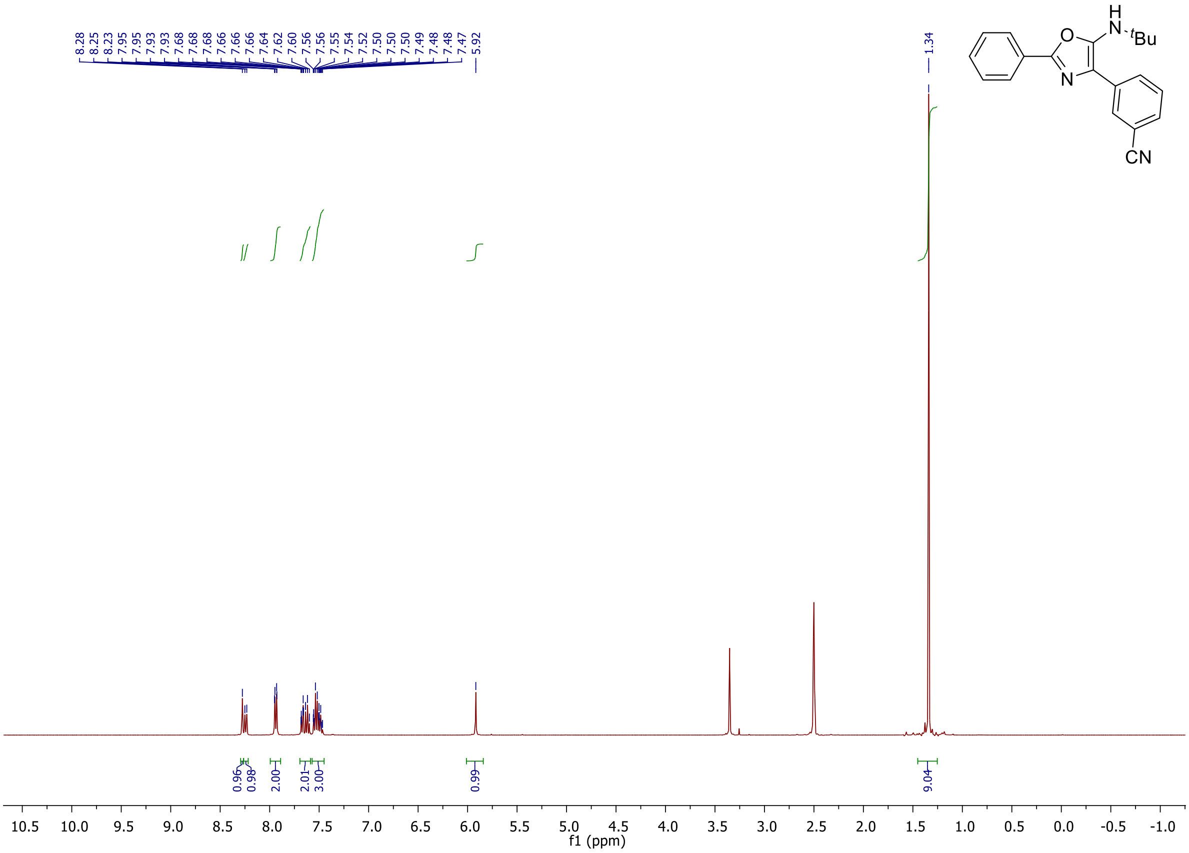

^{13}C{^{1}H} NMR (101 MHz, DMSO-d$_6$)

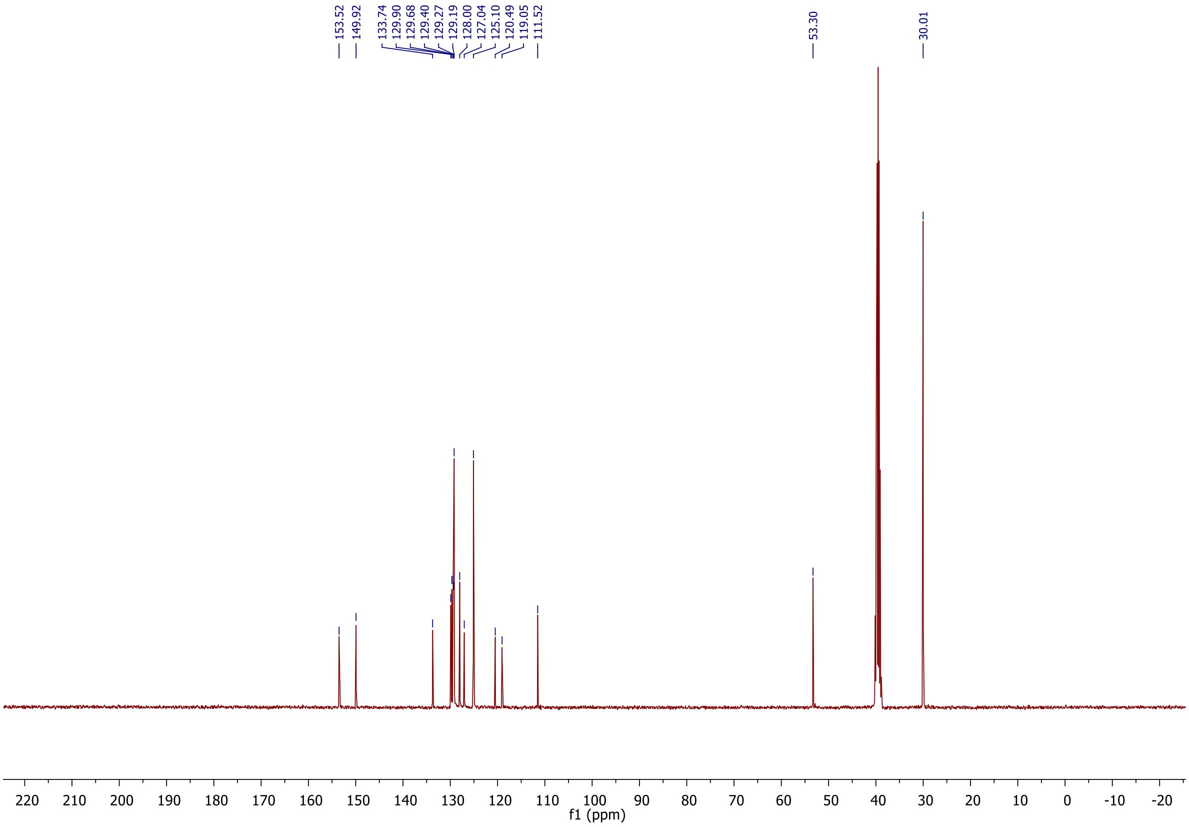

Figure S.32. *N-tert*-butyl-4-(2-methoxyphenyl)-2-phenyl-1,3-oxazol-5-amine (2h)

^{1}H NMR (400 MHz, DMSO-d$_6$)

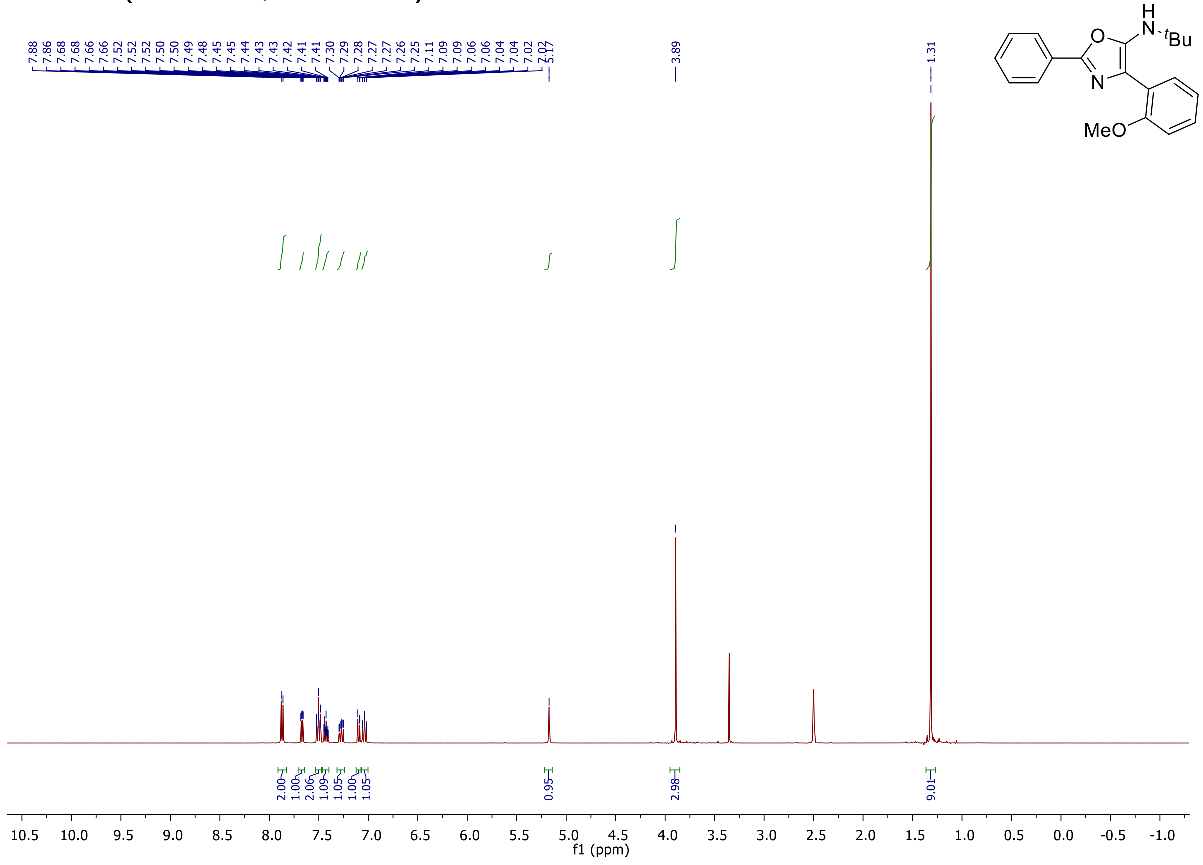

^{13}C{^{1}H} NMR (101 MHz, DMSO-d$_6$)

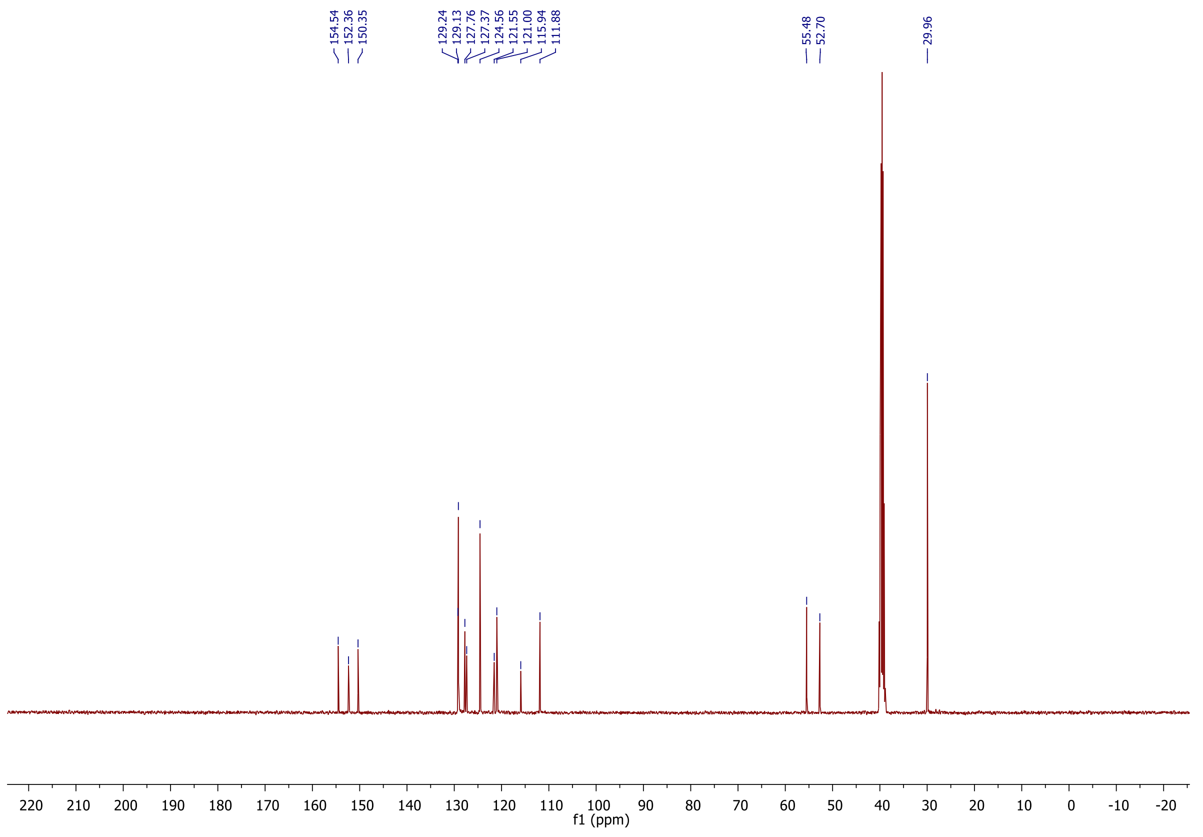

Figure S.33. *N-tert*-butyl-4-(3-methylphenyl)-2-phenyl-1,3-oxazol-5-amine (2i)

^{1}H NMR (400 MHz, DMSO-d$_6$)

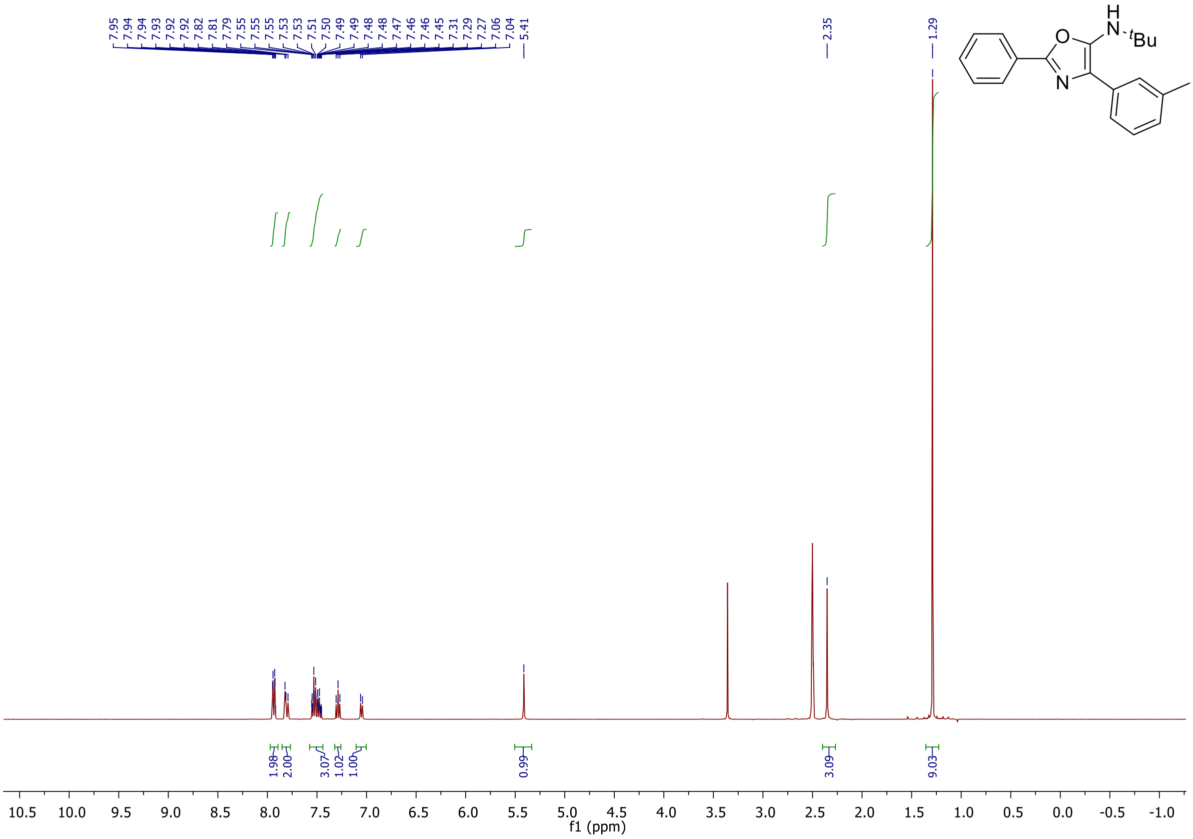

^{13}C{^{1}H} NMR (101 MHz, DMSO-d$_6$)

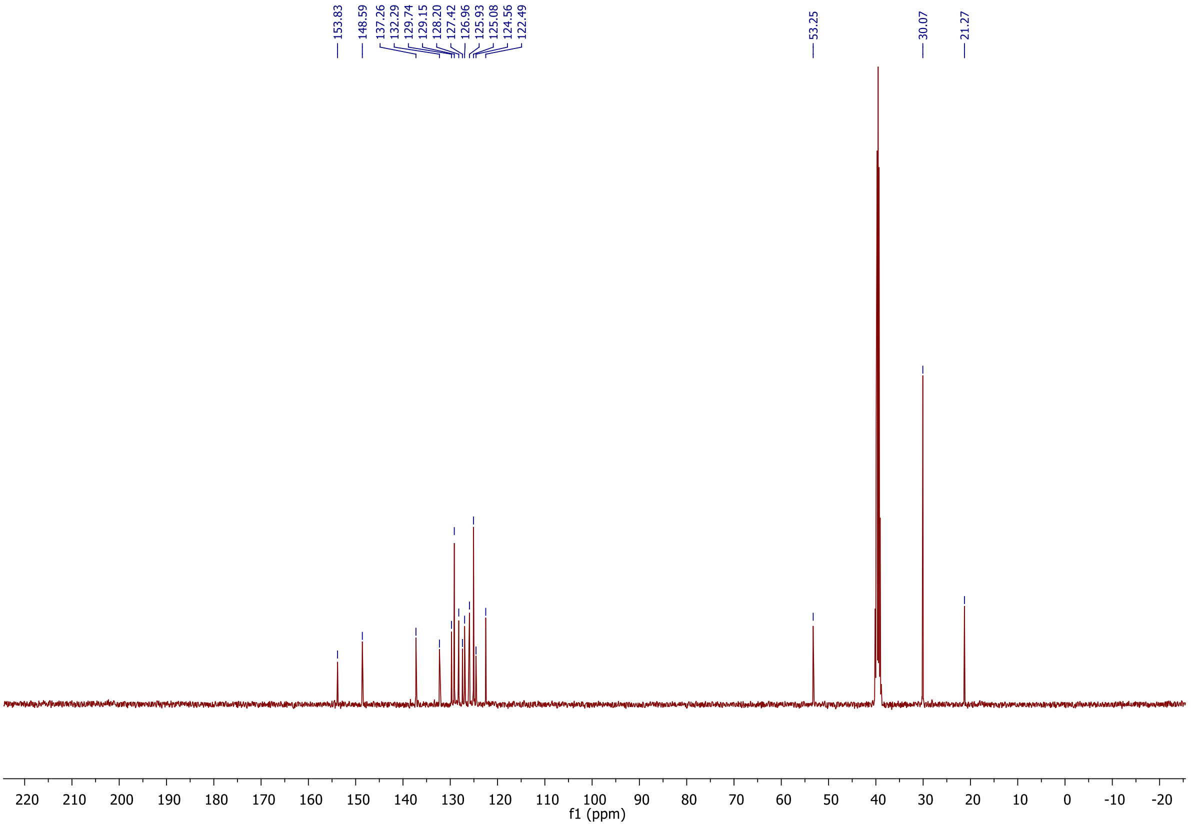

Figure S.34. ***N-tert*-butyl-4-(4-methoxyphenyl)-2-phenyl-1,3-oxazol-5-amine (2j)**

^{1}H NMR (400 MHz, DMSO-d$_6$)

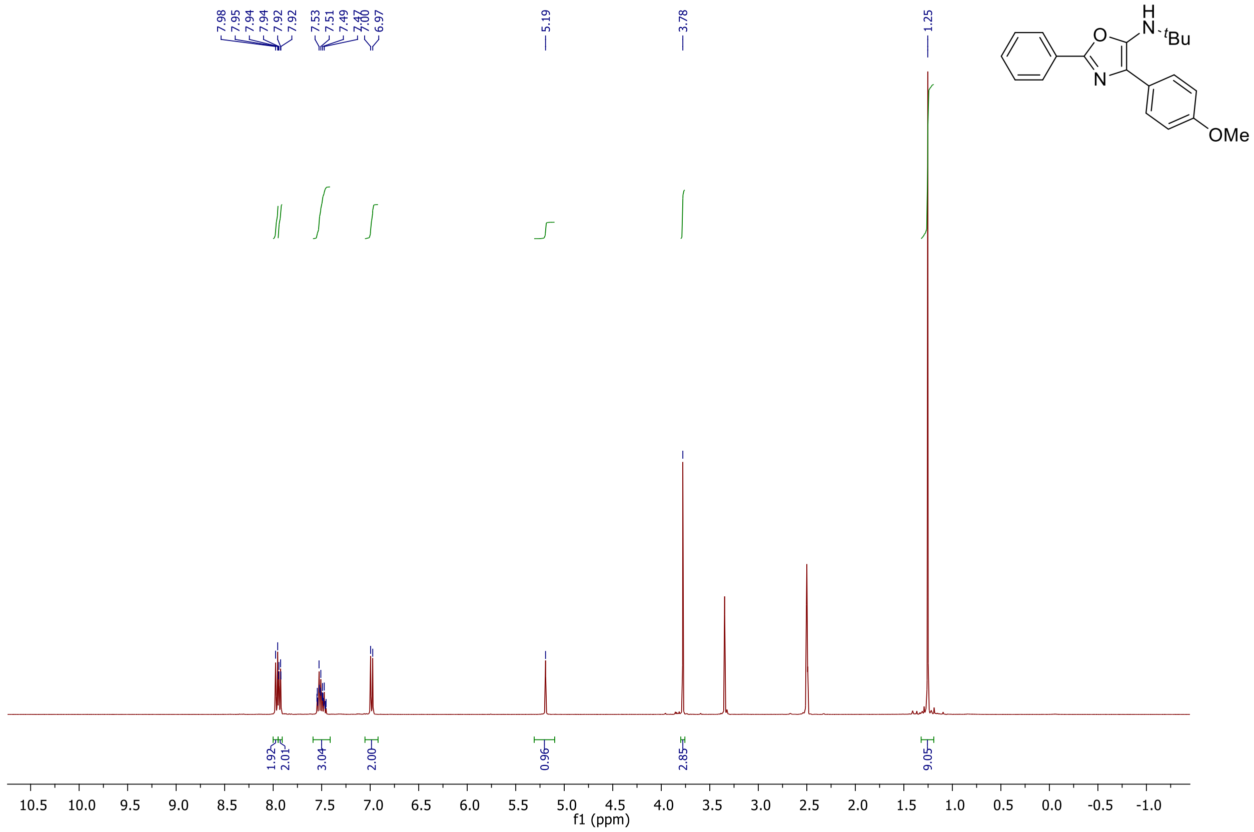

^{13}C{^{1}H} NMR (101 MHz, DMSO-d$_6$)

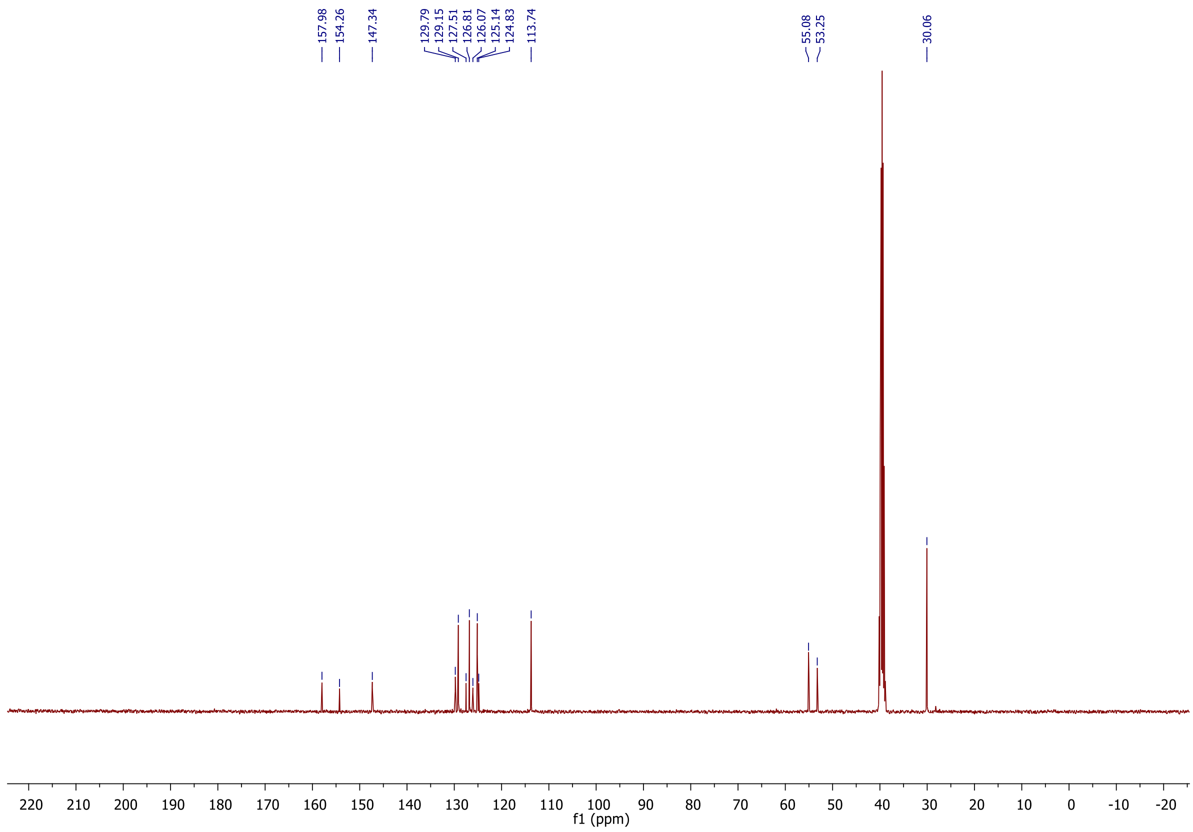

Figure S.35. ***N-tert*-butyl-2-phenyl-4-(thiophen-2-yl)-1,3-oxazol-5-amine (2k)**

^{1}H NMR (400 MHz, DMSO-d6)

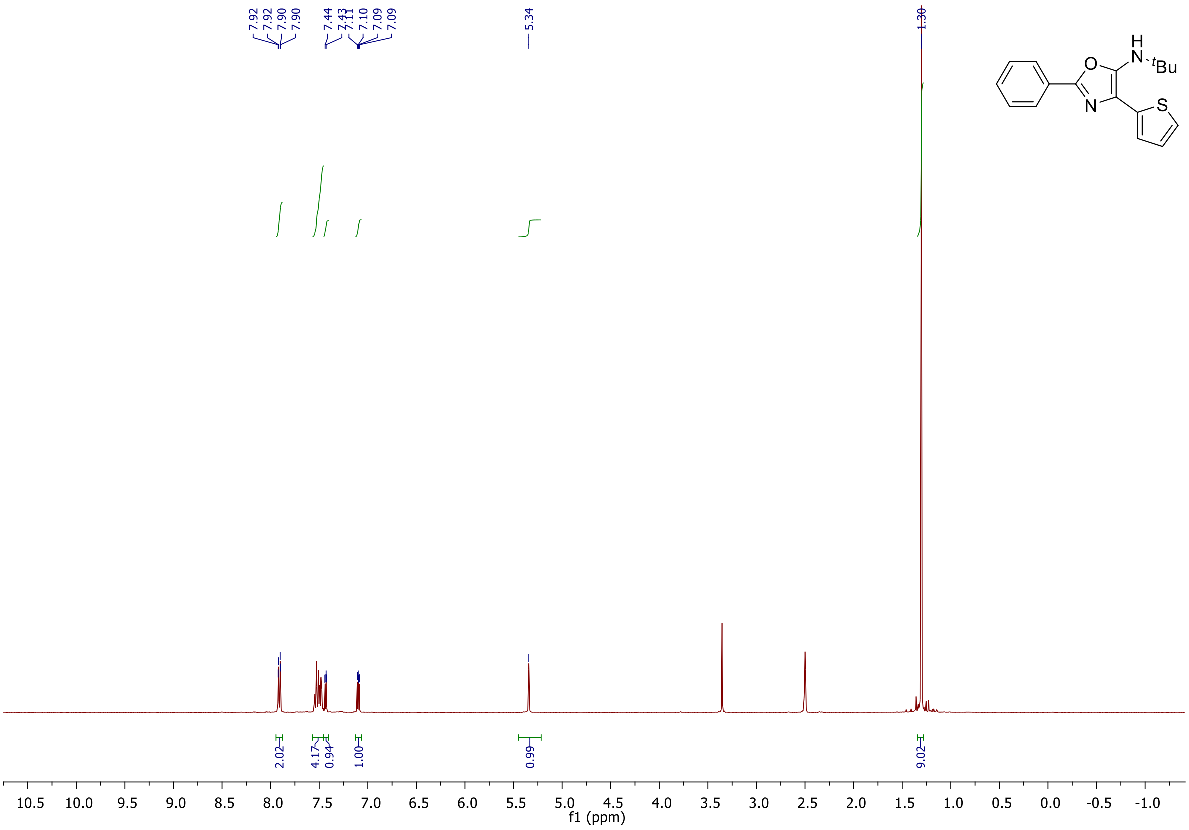

^{13}C{^{1}H} NMR (101 MHz, DMSO-d6)

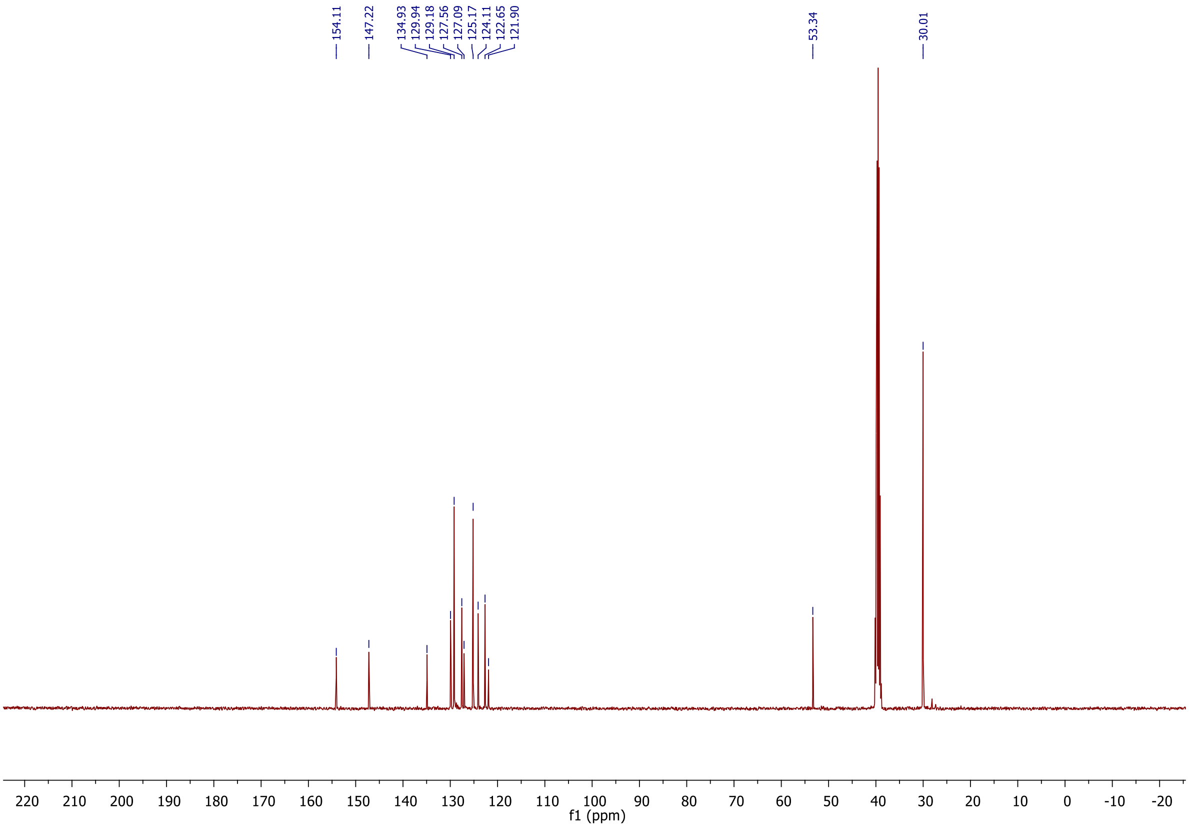

Figure S.36. ***N-tert*-butyl-4-(naphthalen-2-yl)-2-phenyl-1,3-oxazol-5-amine (2l)**

^{1}H NMR (400 MHz, DMSO-d$_6$)

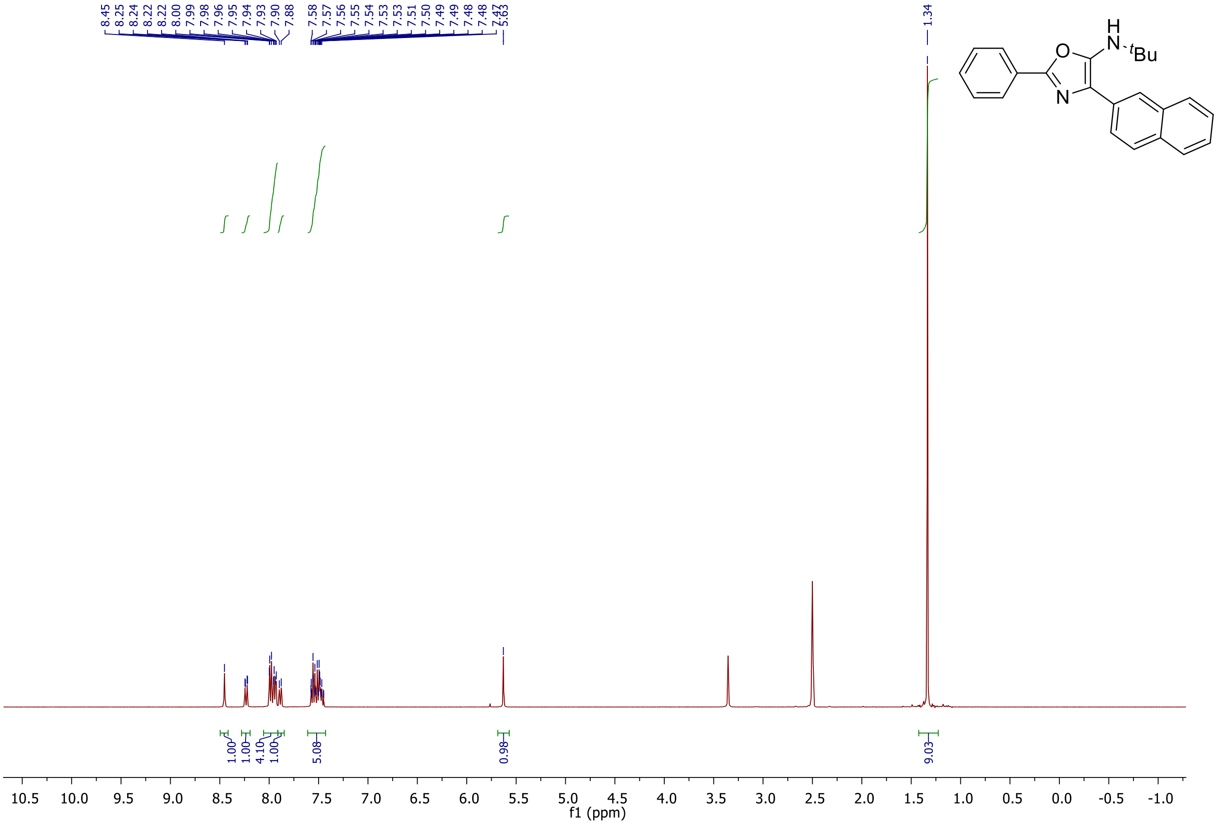

^{13}C{^{1}H} NMR (101 MHz, DMSO-d$_6$)

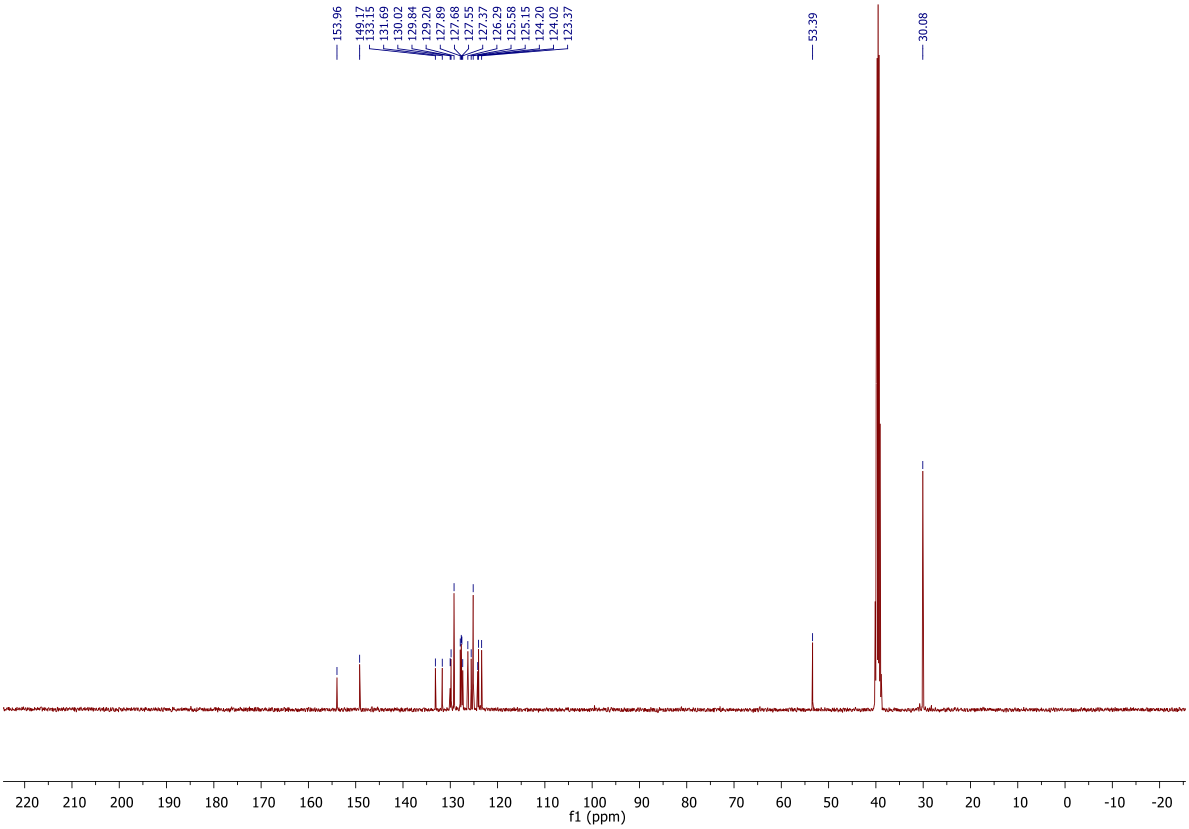

Figure S.37. 2-(4-bromophenyl)-*N-tert*-butyl-4-phenyl-1,3-oxazol-5-amine (3a)

^{1}H NMR (400 MHz, DMSO-d$_6$)

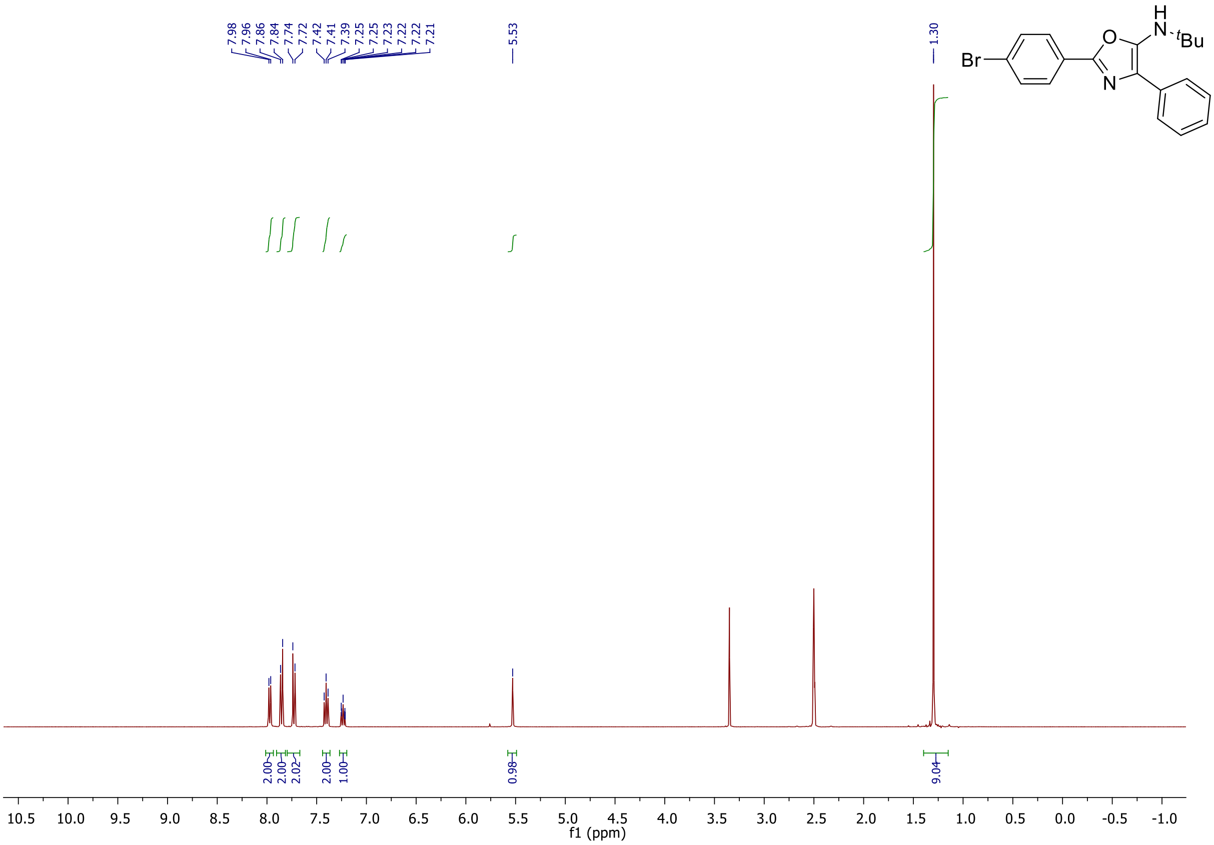

^{13}C{^{1}H} NMR (101 MHz, DMSO-d$_6$)

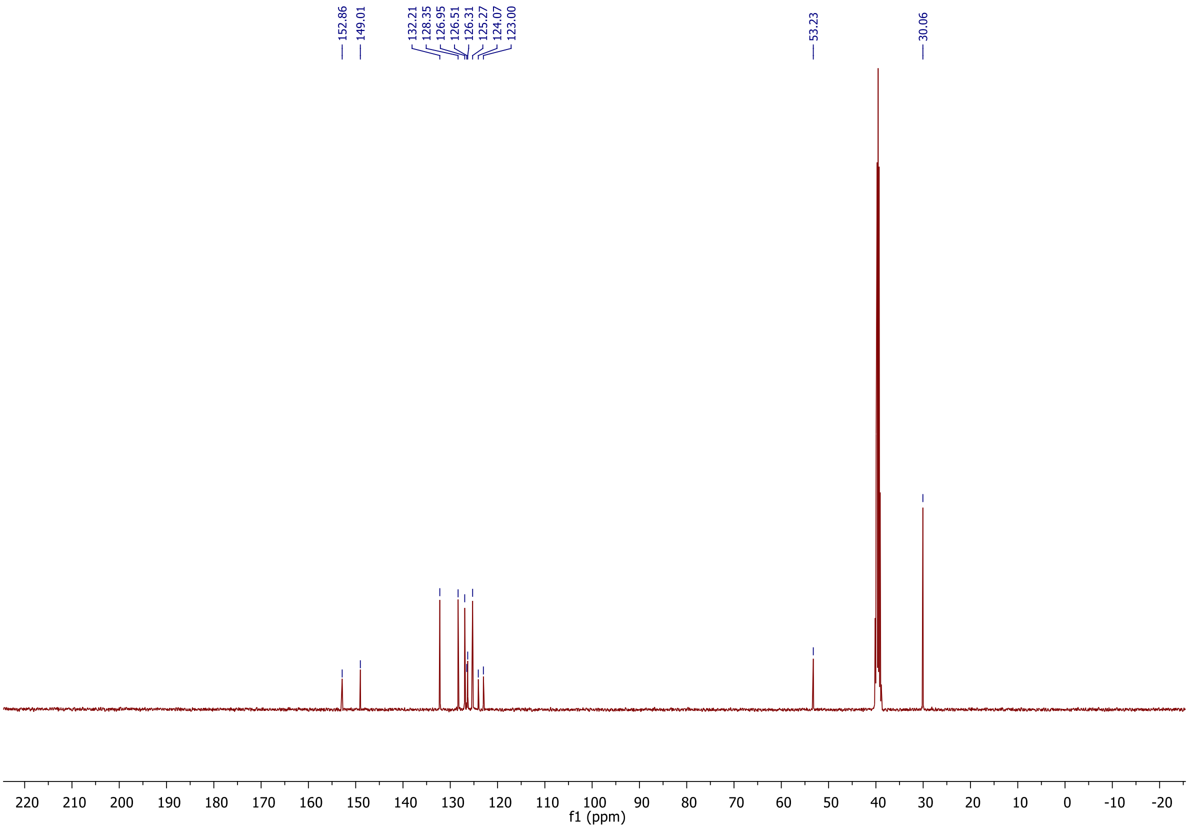

Figure S.38. ***N-tert*-butyl-2-(4-chlorophenyl)-4-phenyl-1,3-oxazol-5-amine (3b)**

^{1}H NMR (400 MHz, DMSO-d$_6$)

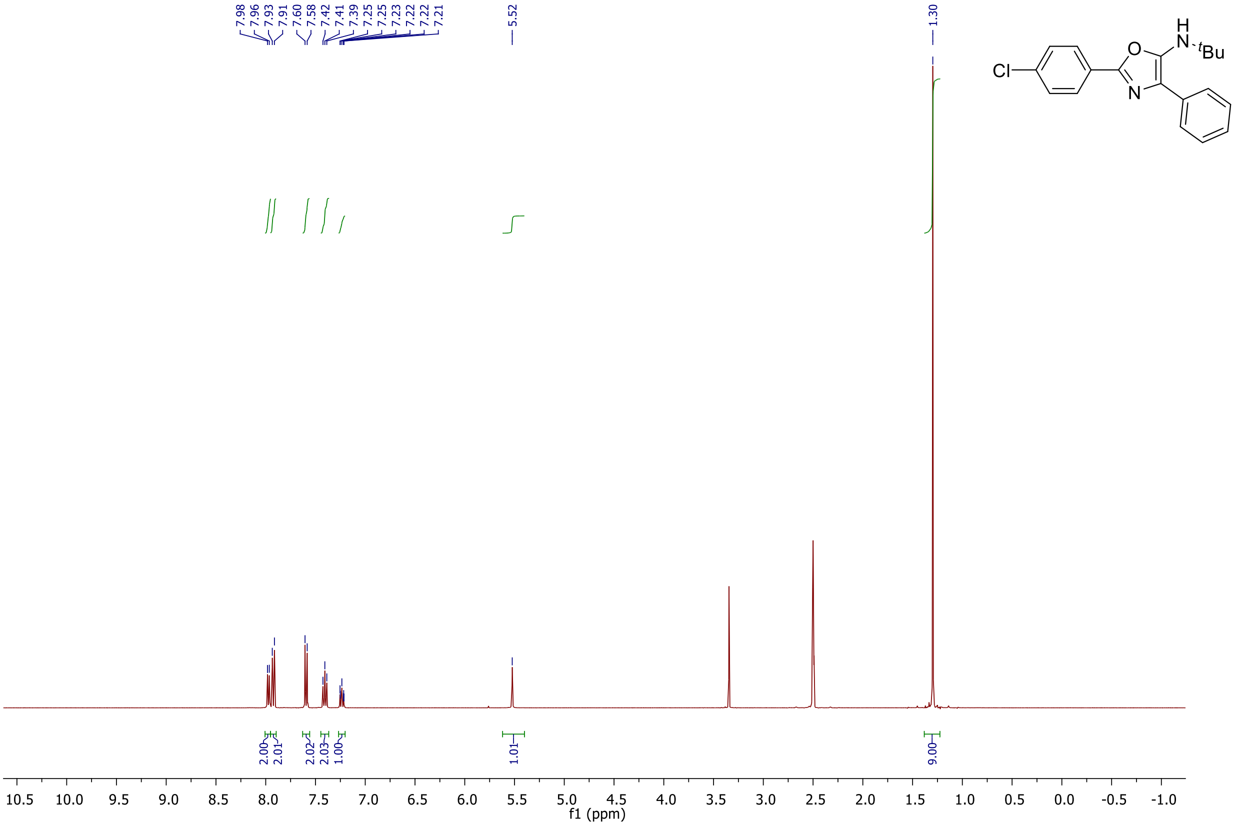

^{13}C{^{1}H} NMR (101 MHz, DMSO-d$_6$)

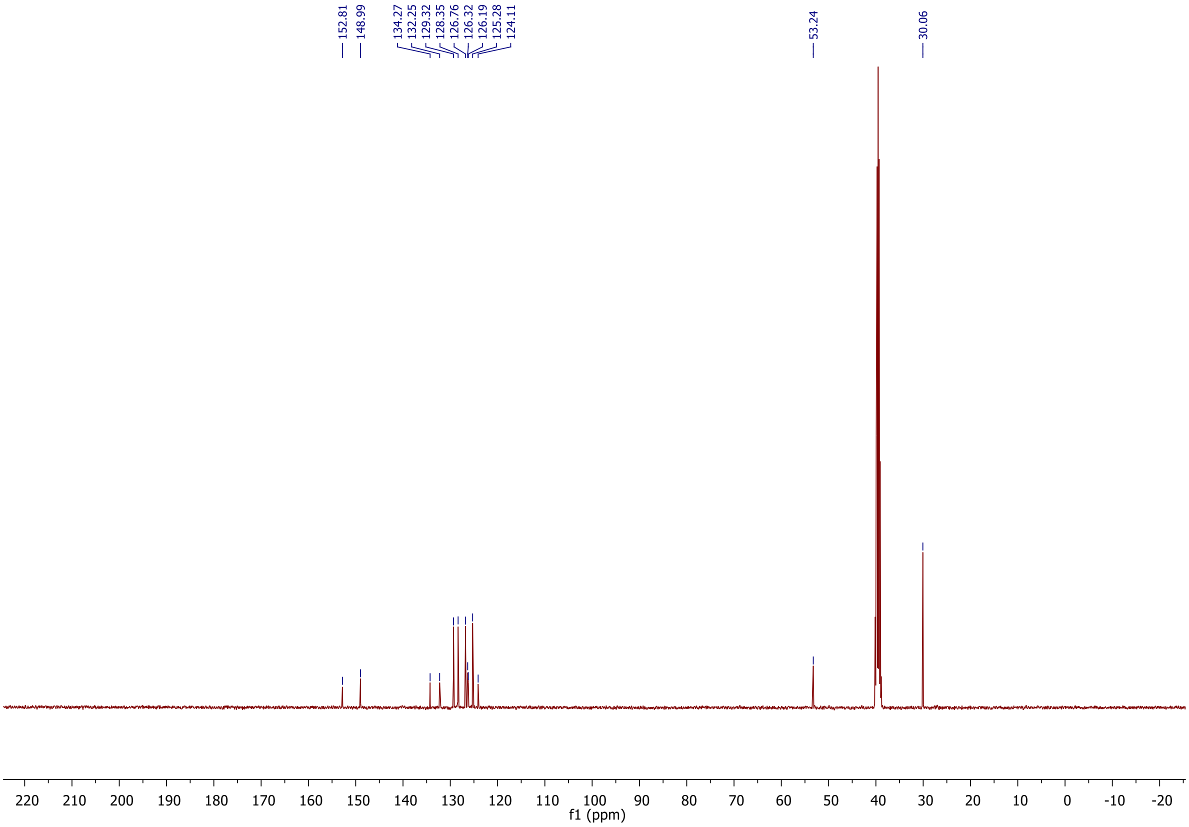

Figure S.39. ***N-tert*-butyl-4-phenyl-2-[4-(trifluoromethyl)phenyl]-1,3-oxazol-5-amine (3c)**

^{1}H NMR (400 MHz, DMSO-d$_6$)

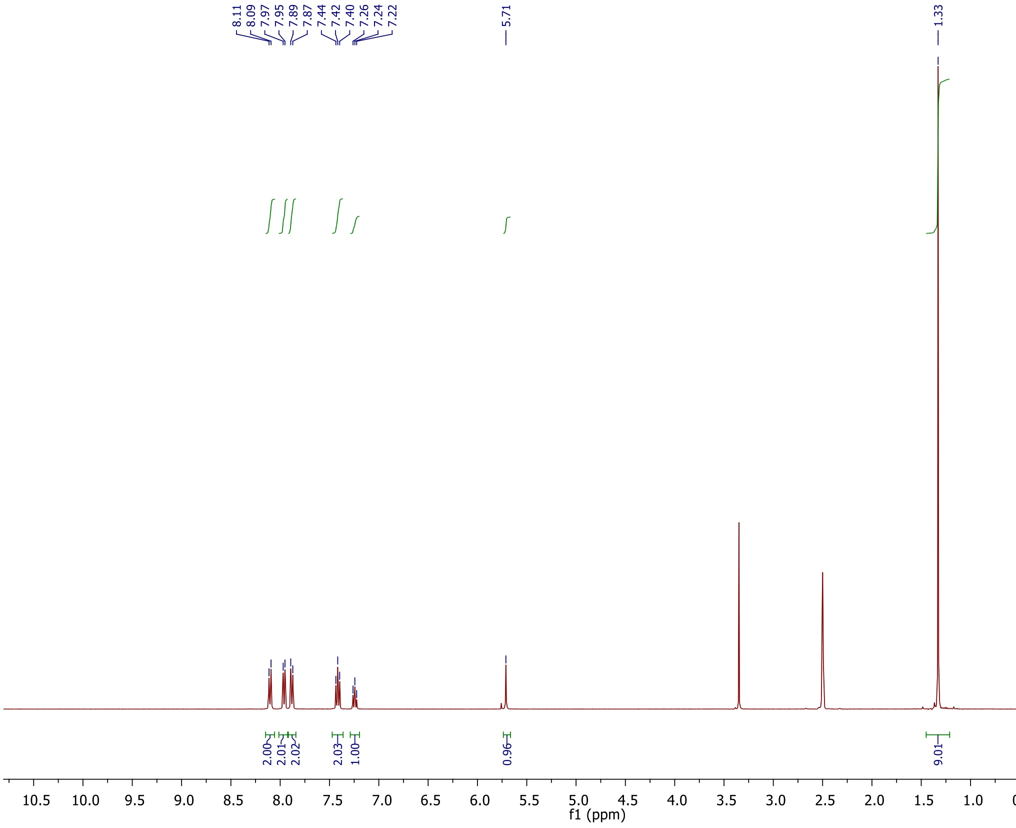

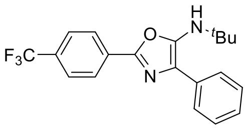

^{13}C{^{1}H} NMR (101 MHz, DMSO-d$_6$)

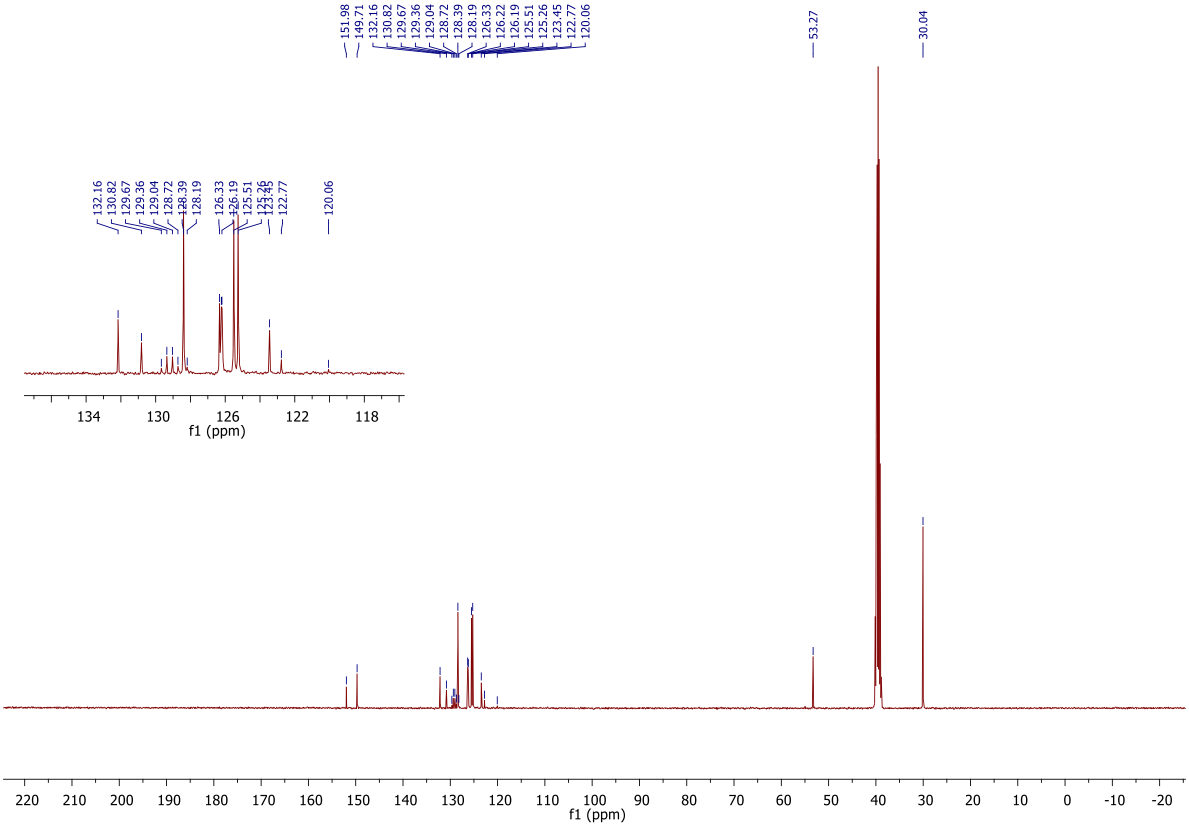

Figure S.40. ***N-tert*-butyl-2-(3,5-difluorophenyl)-4-phenyl-1,3-oxazol-5-amine (3d)**

^{1}H NMR (400 MHz, DMSO-d$_6$)

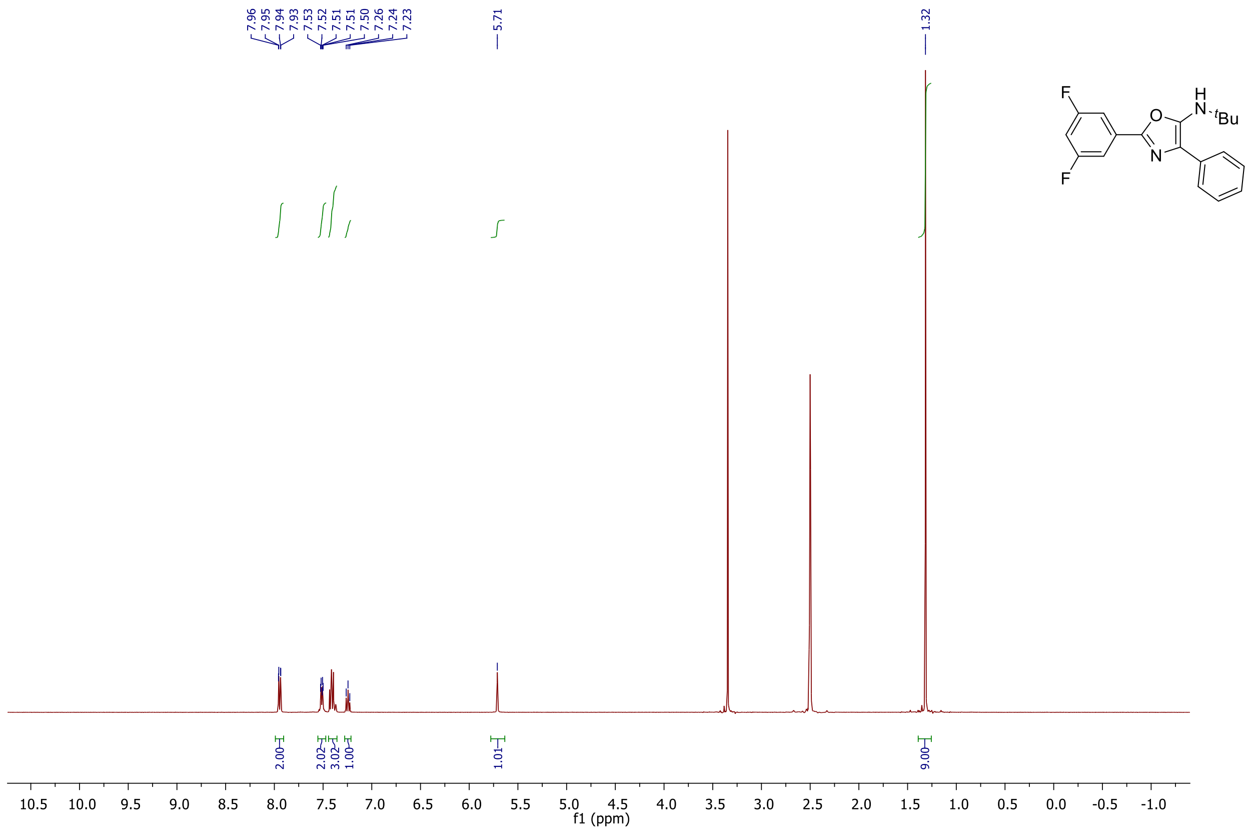

^{13}C{^{1}H} NMR (101 MHz, DMSO-d$_6$)

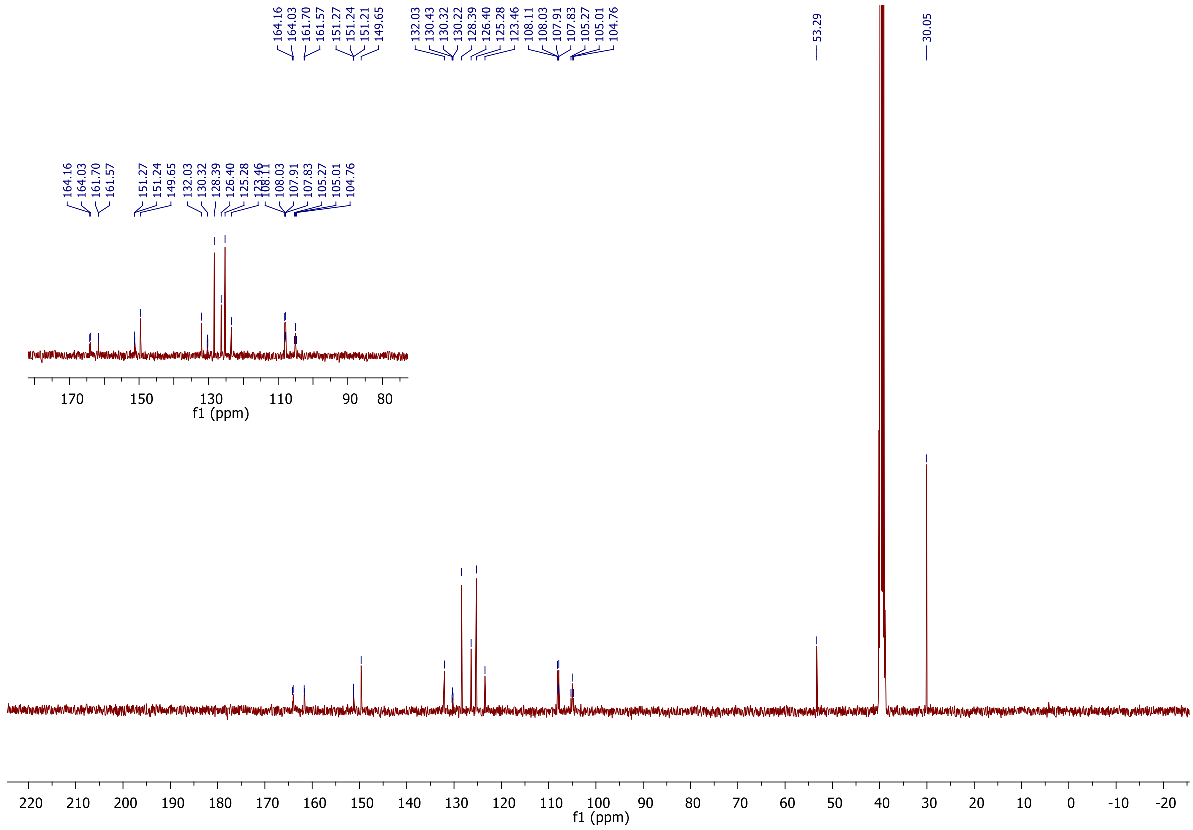

Figure S.41. ***N-tert*-butyl-2-(3-chlorophenyl)-4-phenyl-1,3-oxazol-5-amine (3e)**

^{1}H NMR (400 MHz, DMSO-d$_6$)

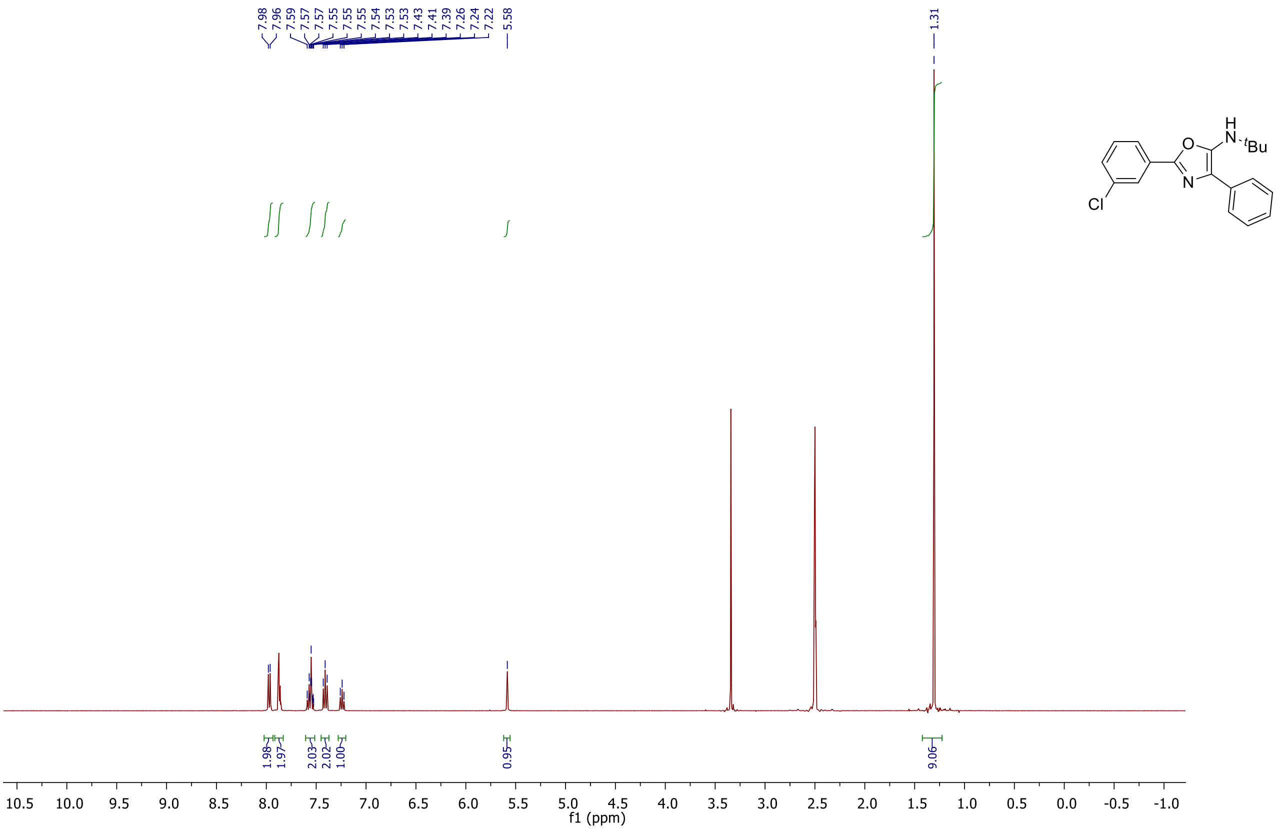

^{13}C{^{1}H} NMR (101 MHz, DMSO-d$_6$)

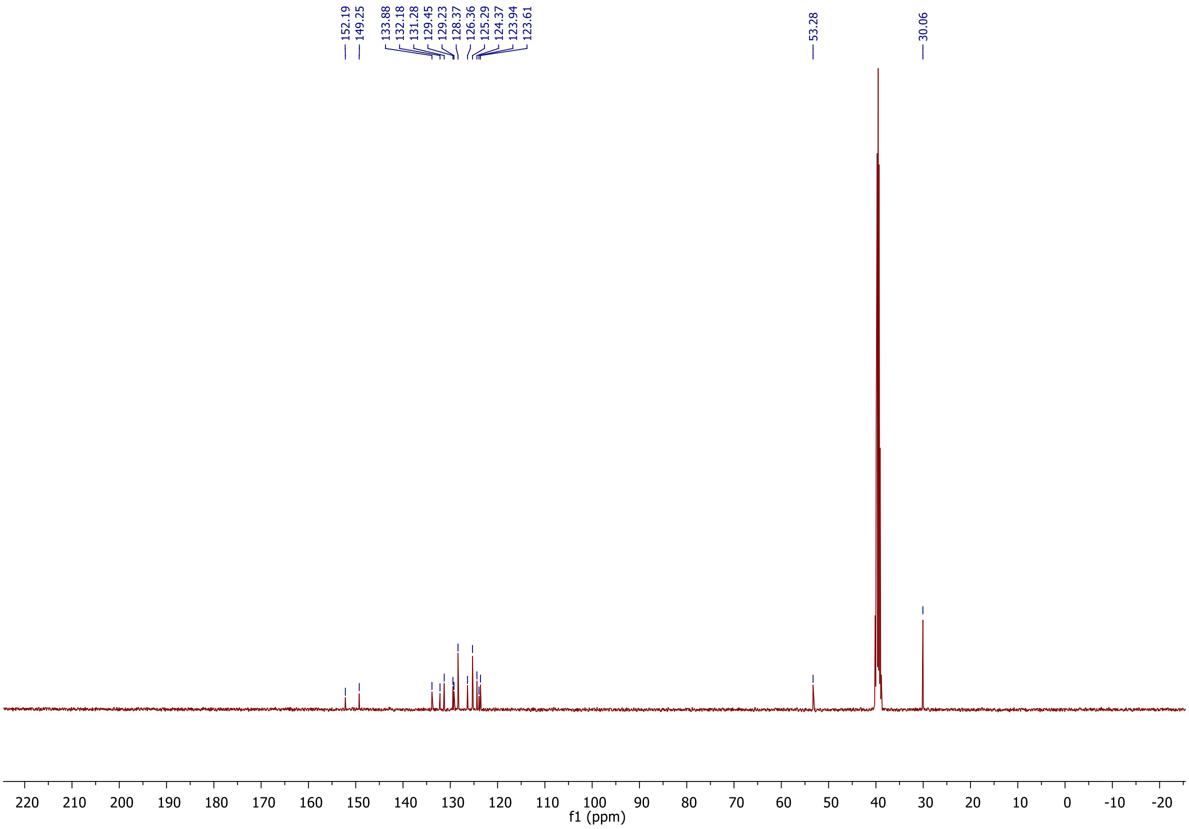

Figure S.42. *N-tert*-butyl-2-(4-methoxyphenyl)-4-phenyl-1,3-oxazol-5-amine (3f)

^{1}H NMR (400 MHz, DMSO-d$_6$)

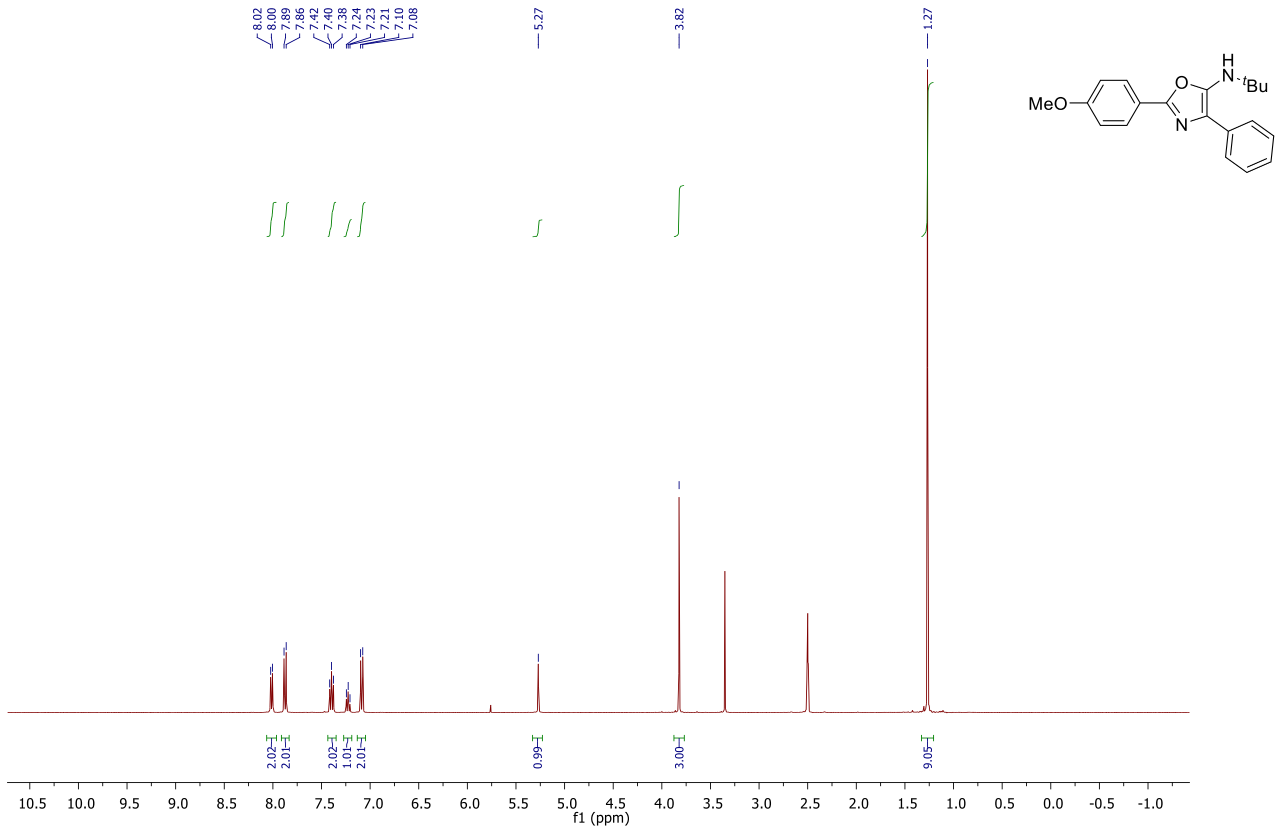

$^{13}C\{^{1}H\}$ NMR (101 MHz, DMSO-d$_6$)

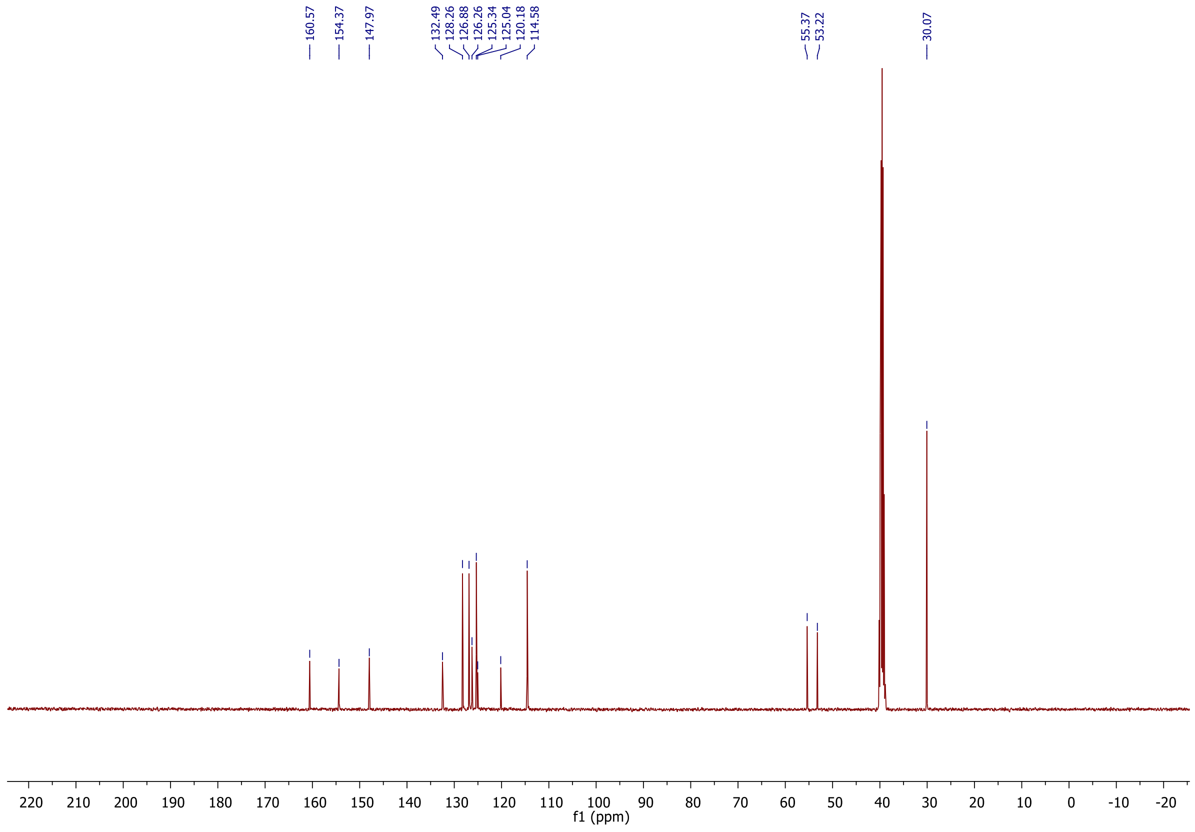

Figure S.43. *N-tert*-butyl-2-(furan-2-yl)-4-phenyl-1,3-oxazol-5-amine (3g)

^{1}H NMR (400 MHz, DMSO-d$_6$)

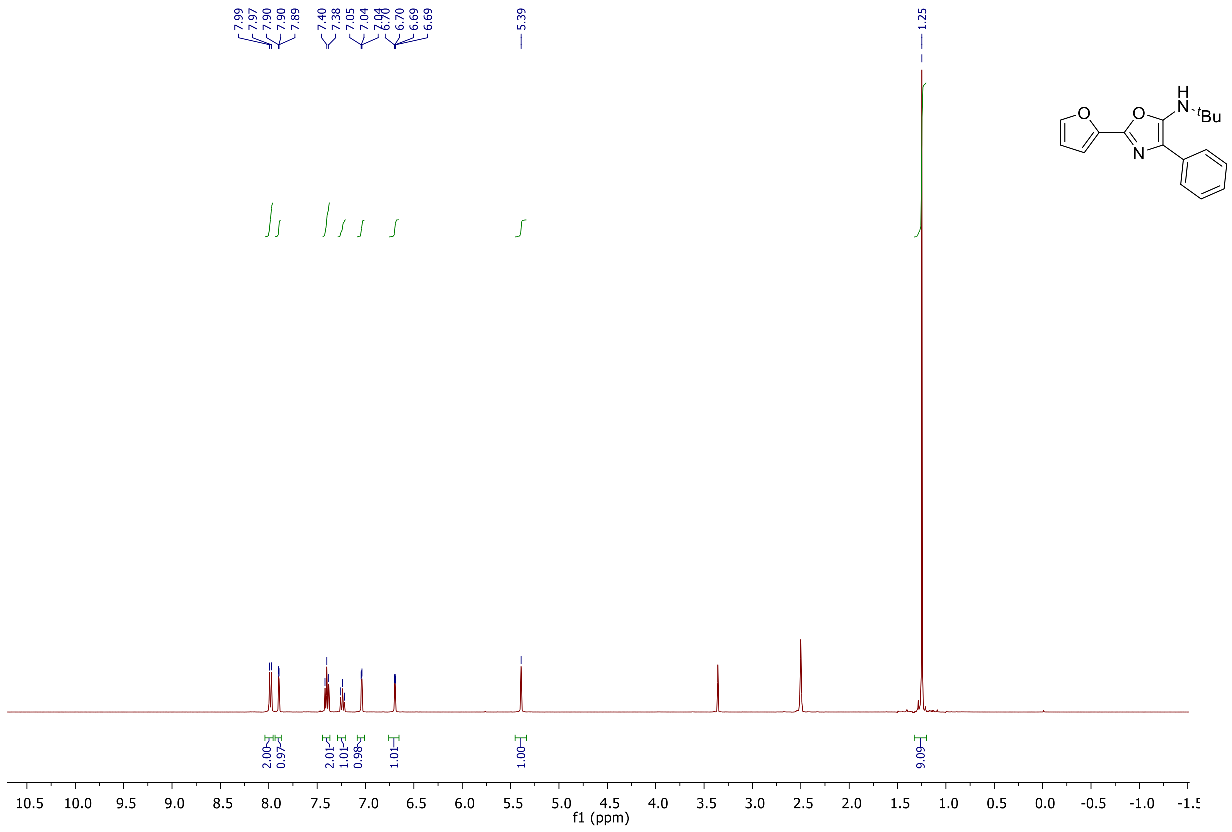

^{13}C{^{1}H} NMR (101 MHz, DMSO-d$_6$)

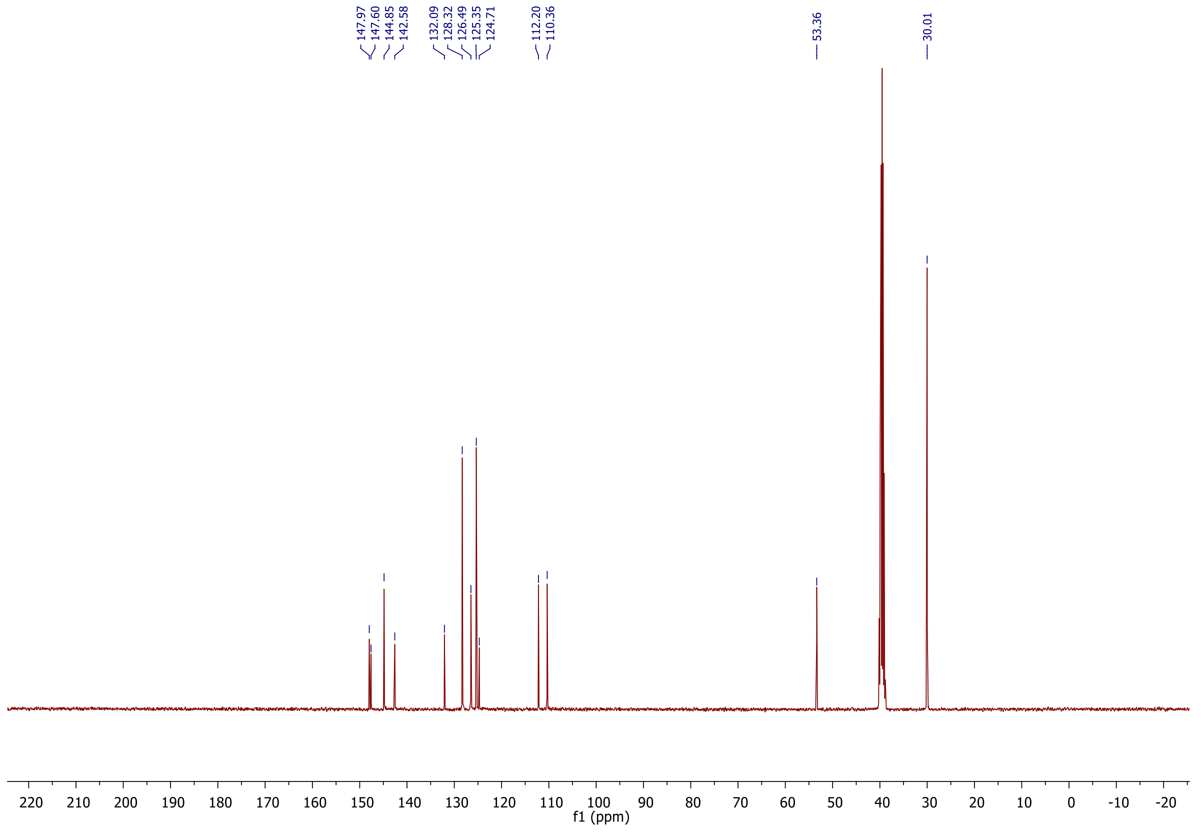

Figure S.44. ***N-tert*-butyl-4-phenyl-2-(thiophen-2-yl)-1,3-oxazol-5-amine (3h)**

^{1}H NMR (400 MHz, DMSO-d$_6$)

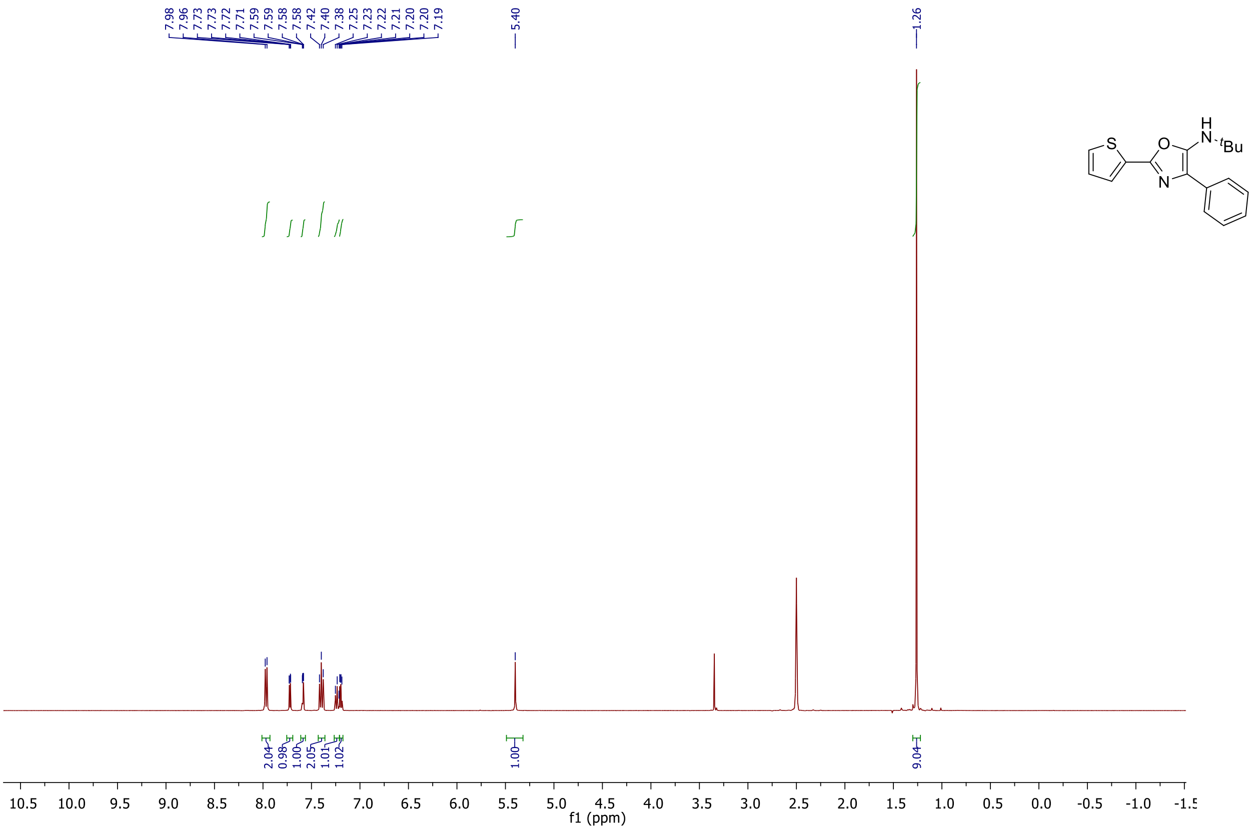

^{13}C{^{1}H} NMR (101 MHz, DMSO-d$_6$)

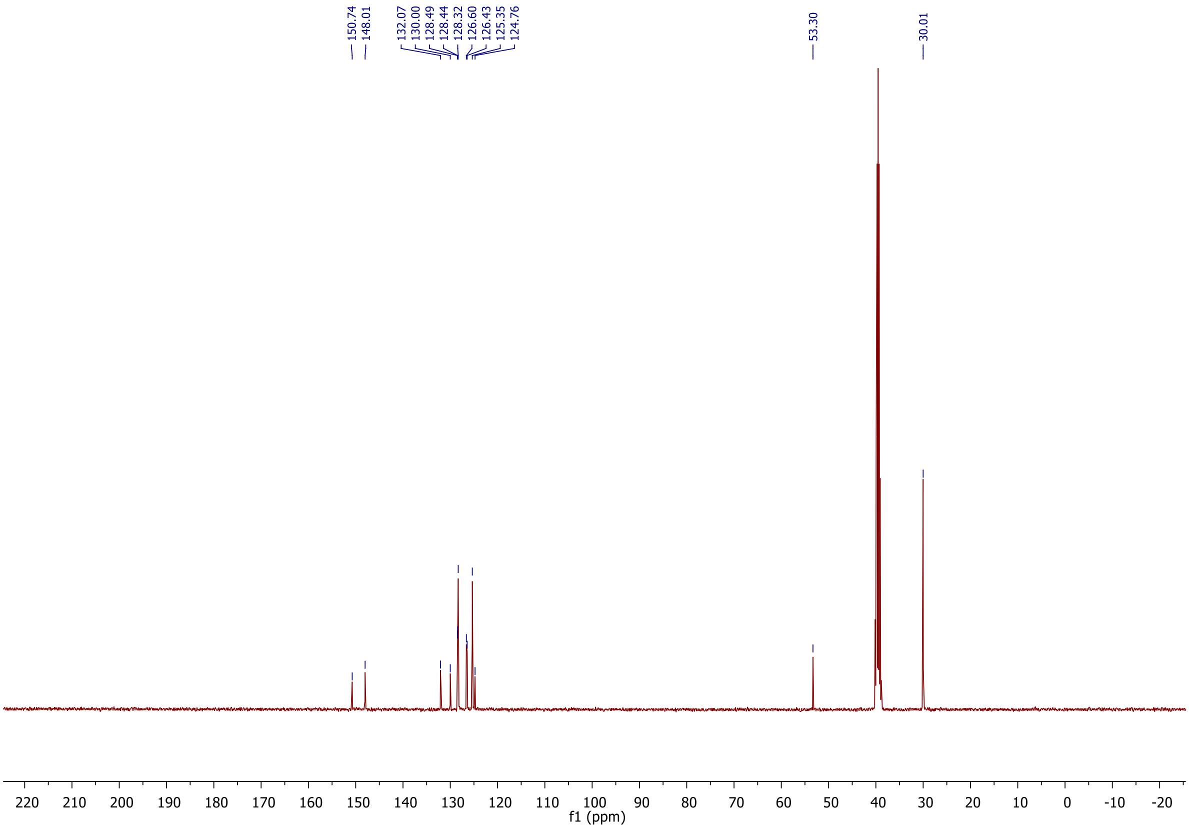

Figure S.45. *N-tert*-butyl-2-(2-methylphenyl)-4-phenyl-1,3-oxazol-5-amine (3i)

^{1}H NMR (400 MHz, DMSO-d$_6$)

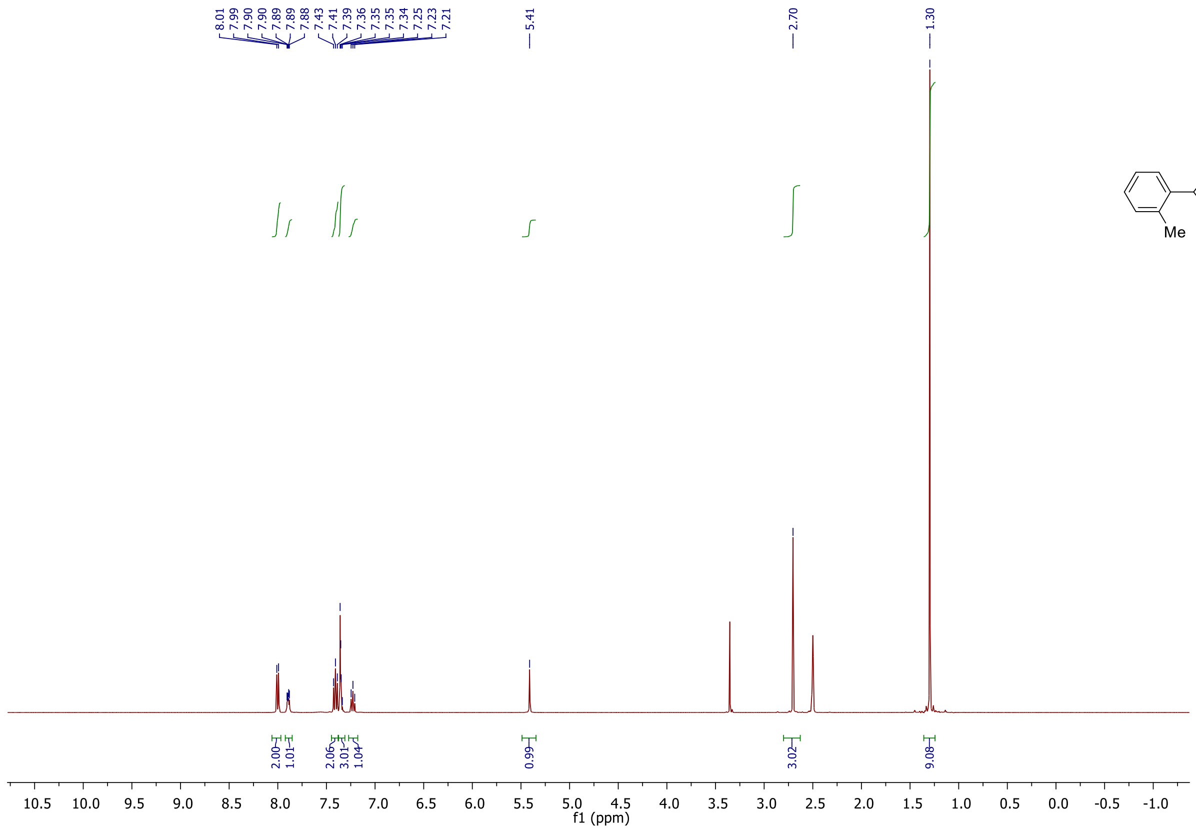

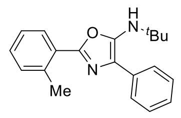

$^{13}C\{^{1}H\}$ NMR (101 MHz, DMSO-d$_6$)

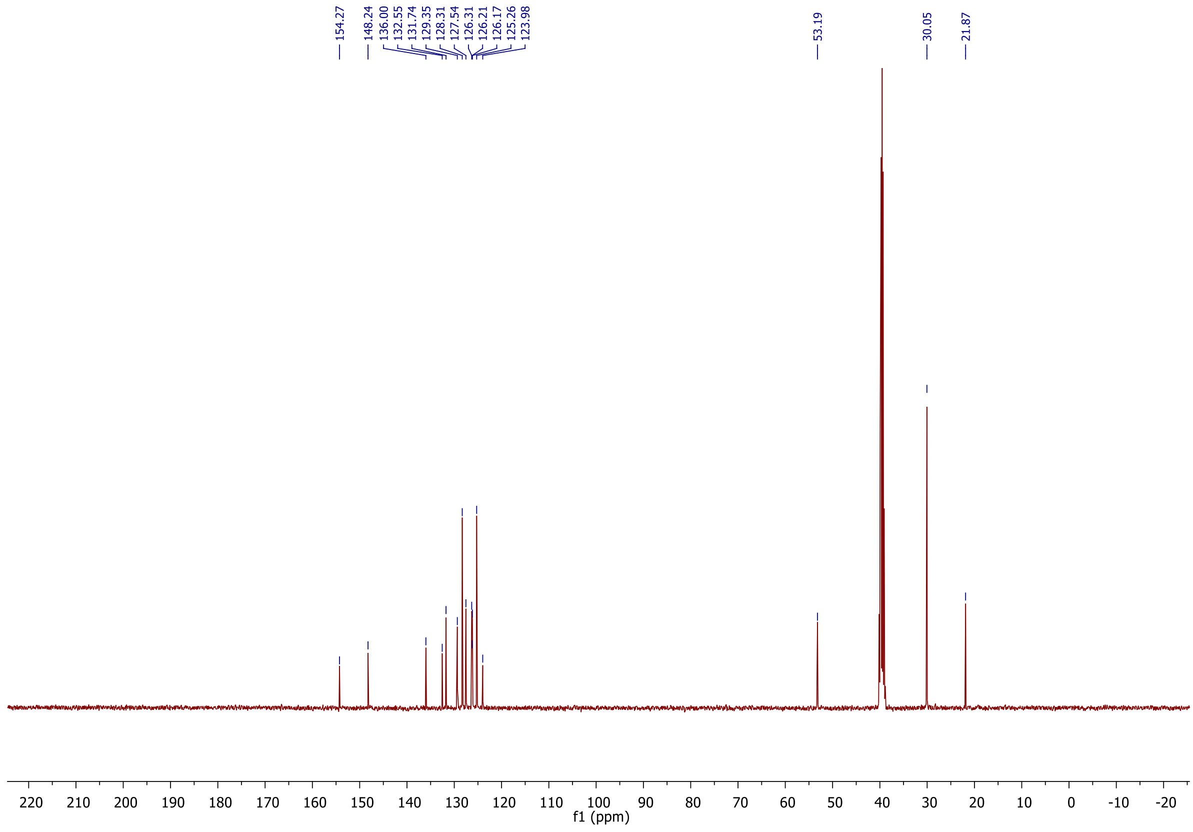

Figure S.46. 2,4-diphenyl-*N*-[4-(trifluoromethyl)phenyl]-1,3-oxazol-5-amine (4a)

^{1}H NMR (400 MHz, DMSO-d$_6$)

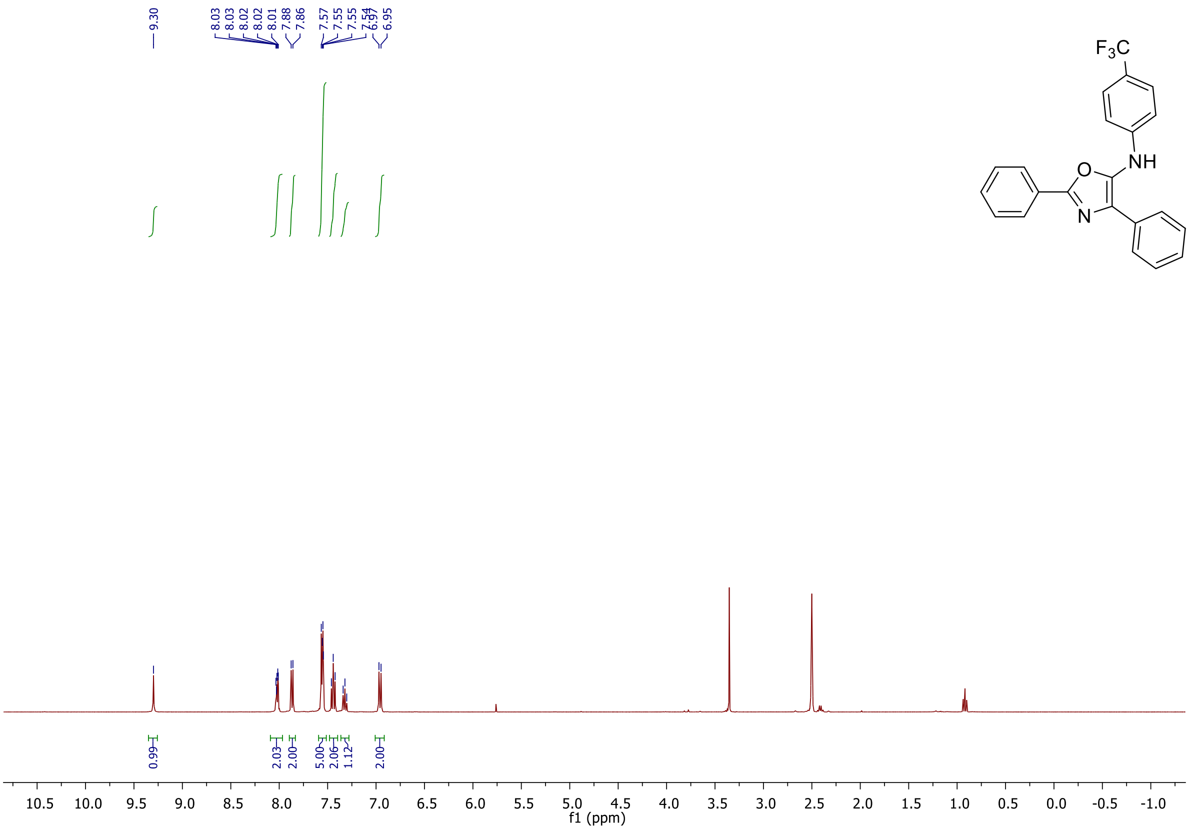

^{13}C{^{1}H} NMR (101 MHz, DMSO-d$_6$)

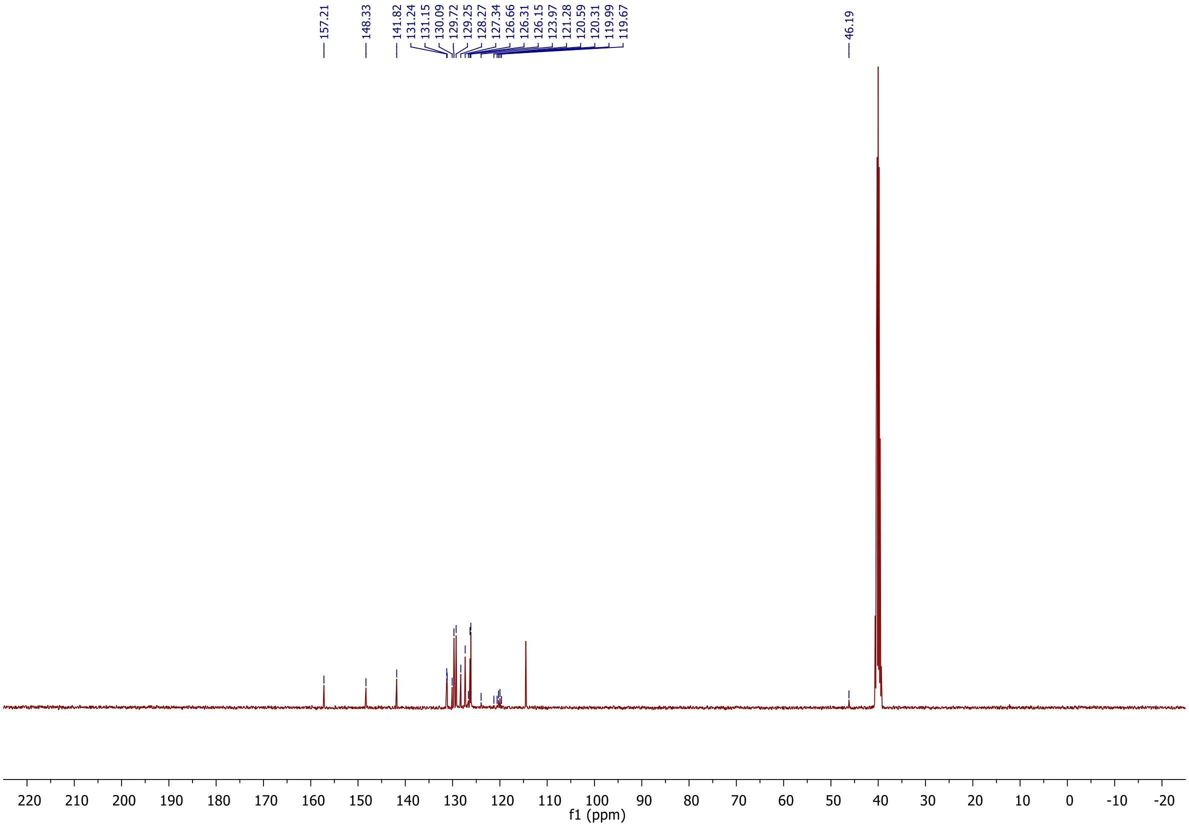

Figure S.47. 4-[(2,4-diphenyl-1,3-oxazol-5-yl)amino]benzonitrile (4b)

^{1}H NMR (400 MHz, DMSO-d_6)

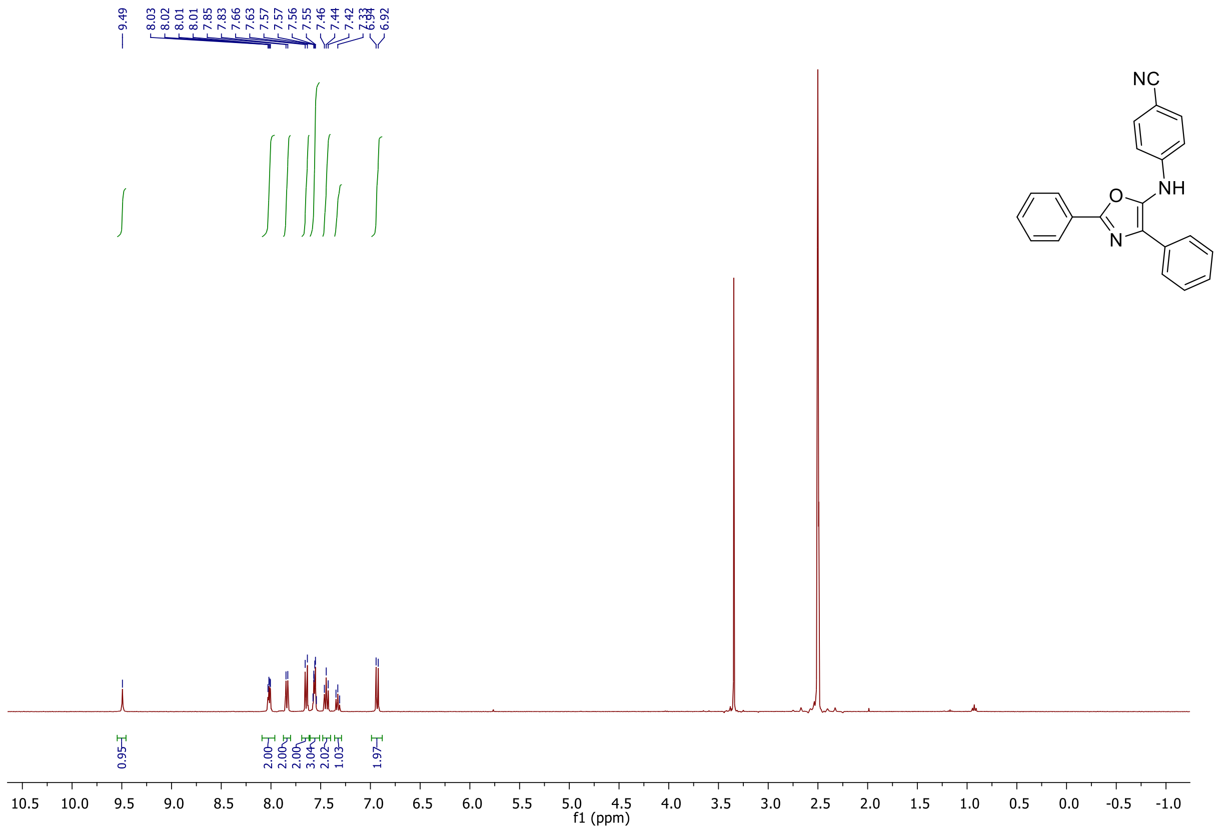

$^{13}C\{^{1}H\}$ NMR (101 MHz, DMSO-d_6)

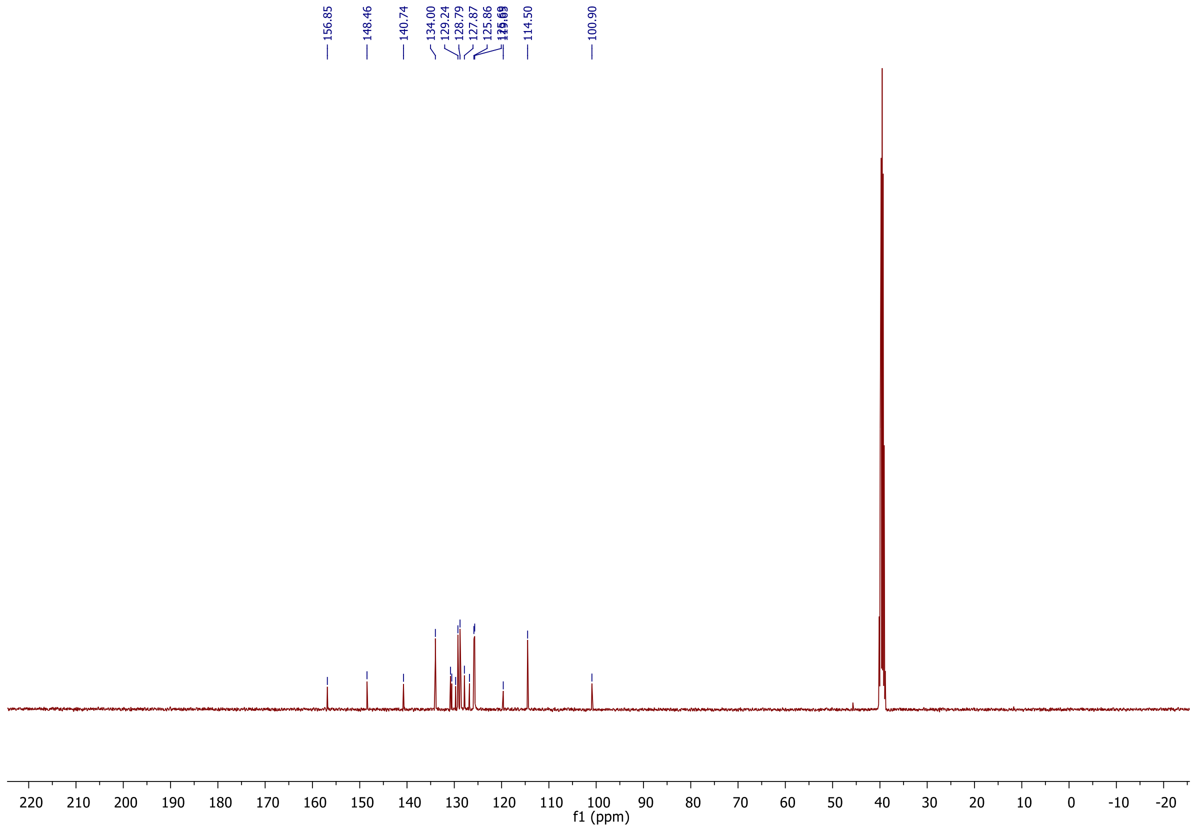

Figure S.48. *N*-(4-bromophenyl)-2,4-diphenyl-1,3-oxazol-5-amine (4c)

^{1}H NMR (400 MHz, DMSO-d$_6$)

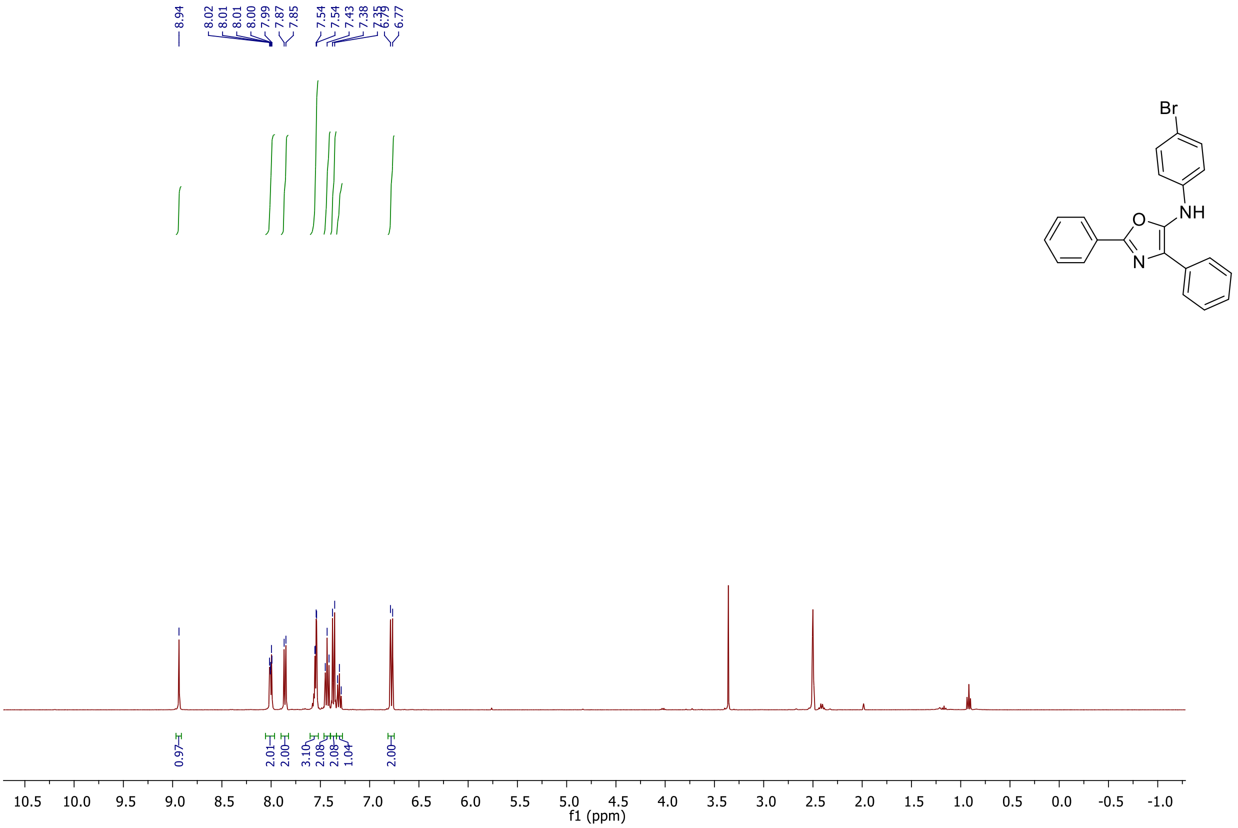

^{13}C{^{1}H} NMR (101 MHz, DMSO-d$_6$)

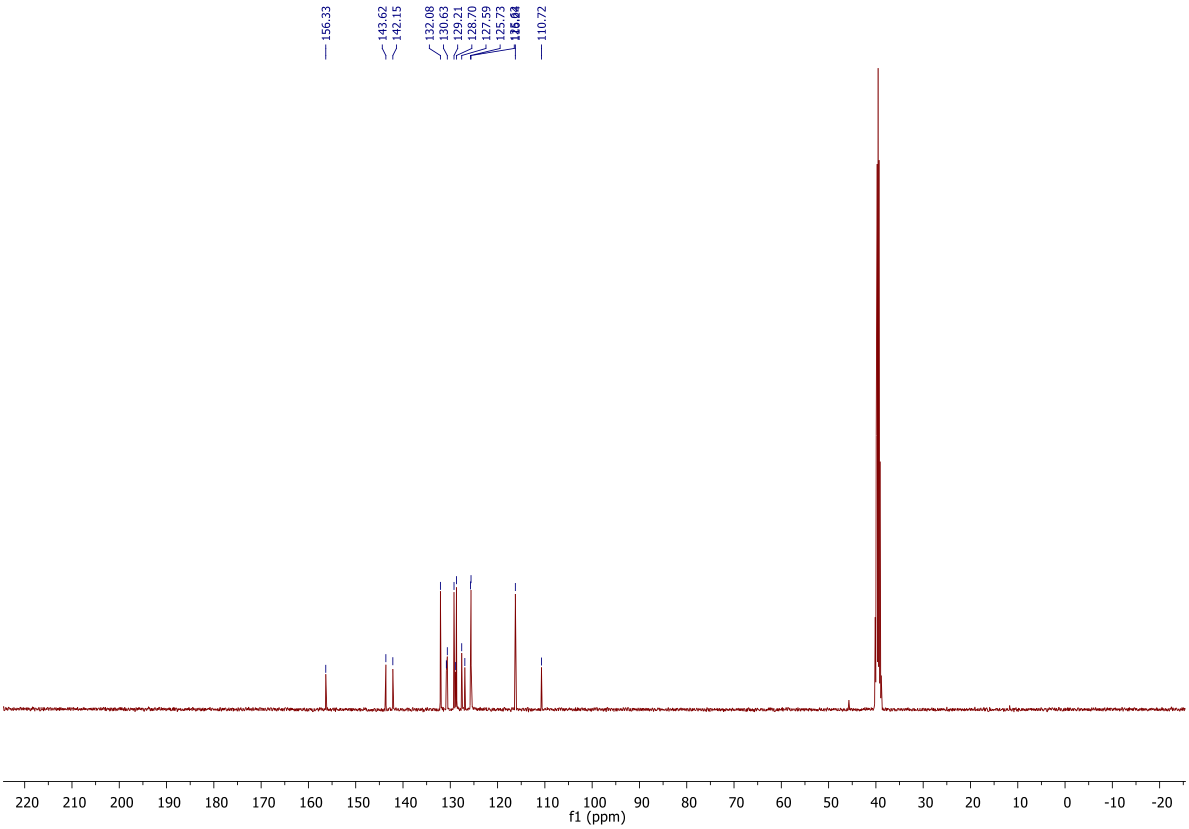

Figure S.49. *N*-(3-methoxyphenyl)-2,4-diphenyl-1,3-oxazol-5-amine (4d)

^{1}H NMR (400 MHz, DMSO-d$_6$)

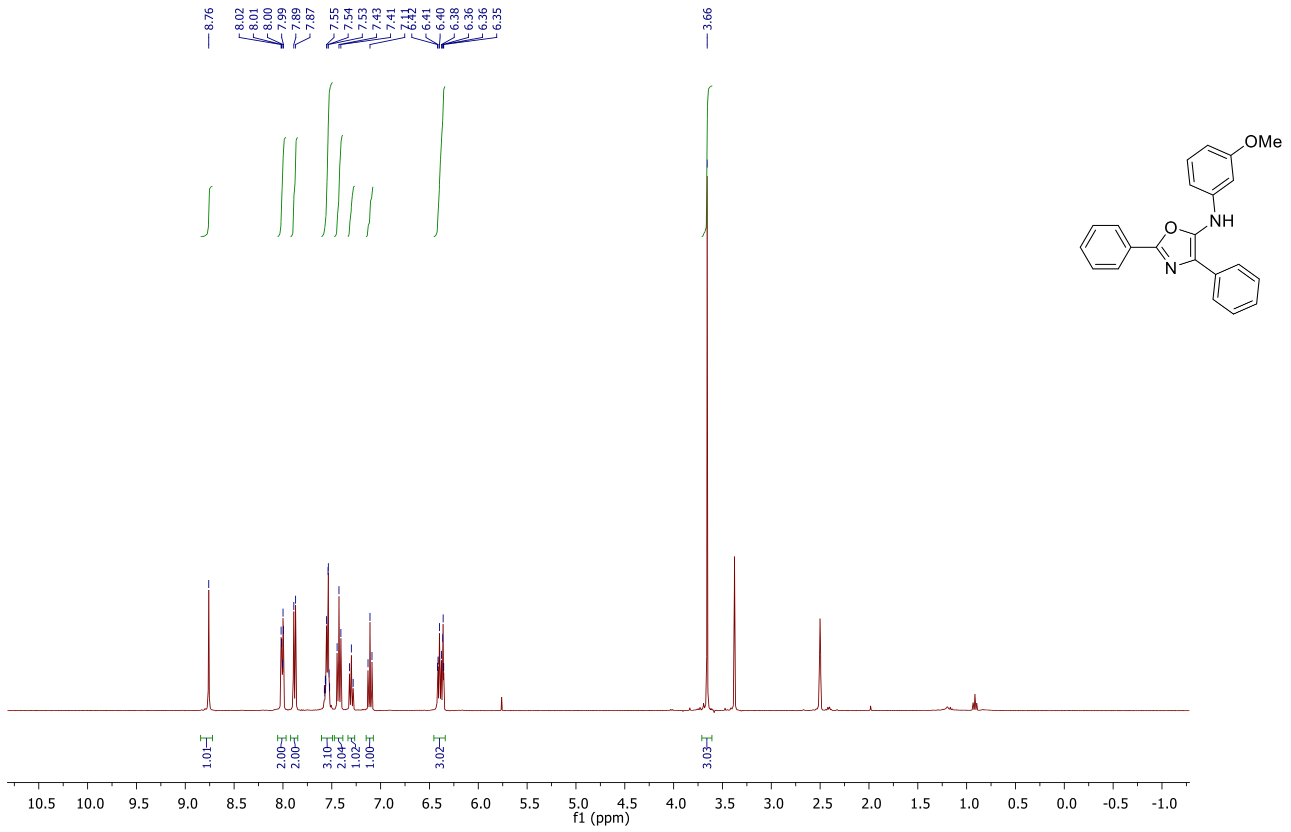

^{13}C{^{1}H} NMR (101 MHz, DMSO-d$_6$)

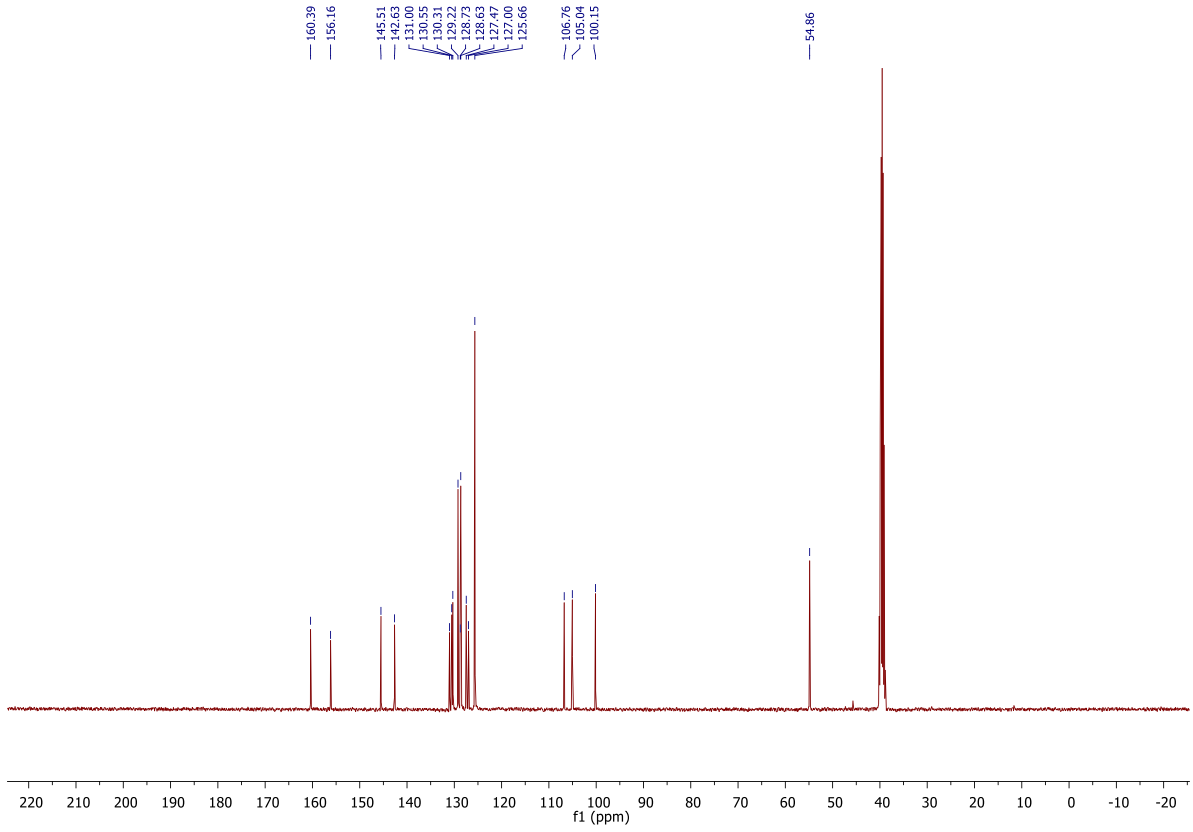

Figure S.50. ***N*-(2-chloro-4-methylphenyl)-2,4-diphenyl-1,3-oxazol-5-amine (4e)**

^{1}H NMR (400 MHz, DMSO-d$_6$)

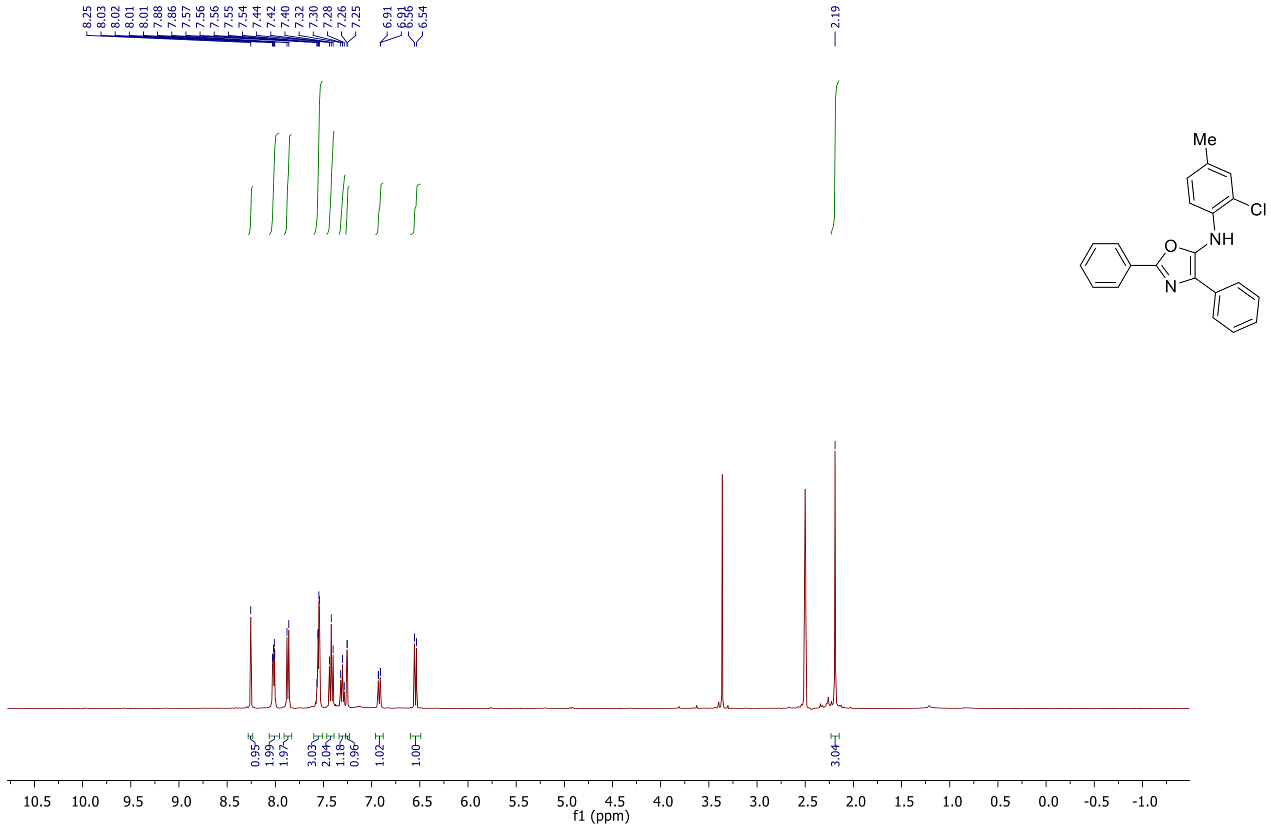

^{13}C{^{1}H} NMR (101 MHz, DMSO-d$_6$)

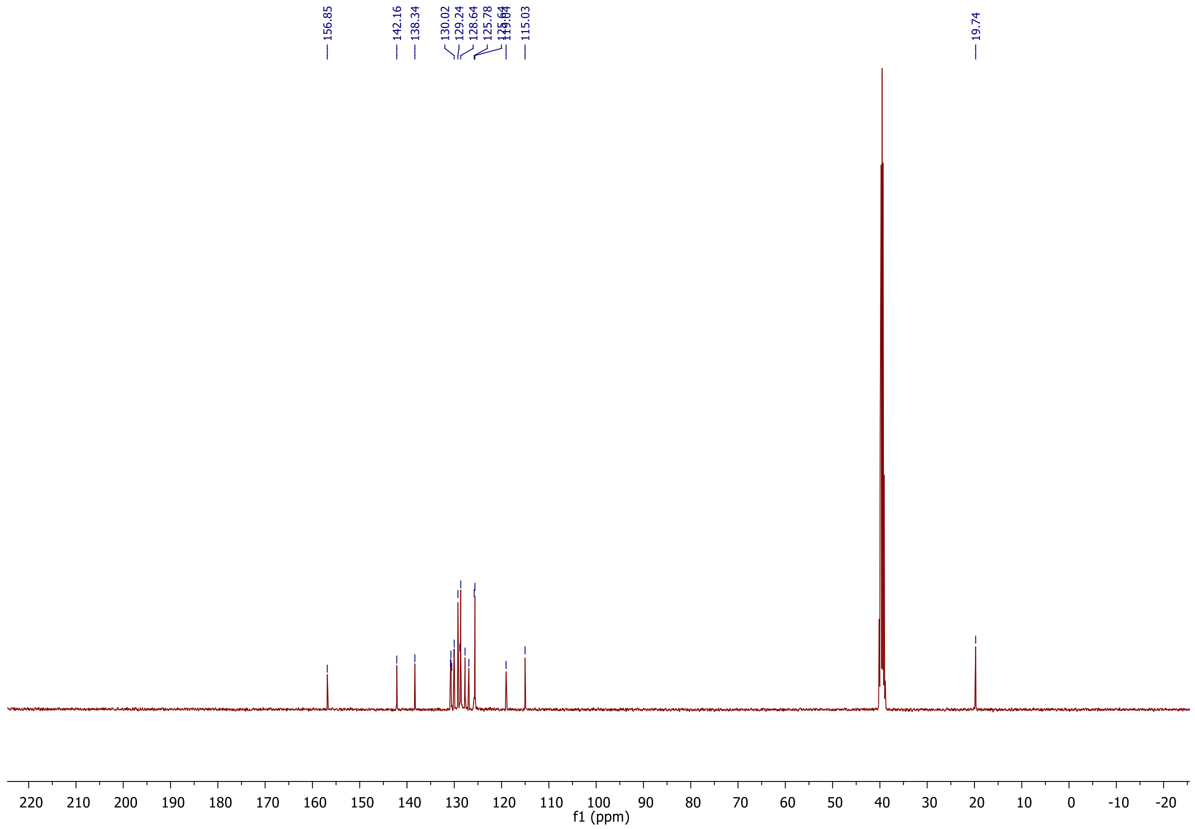

Figure S.51. *N*-benzyl-2,4-diphenyl-1,3-oxazol-5-amine (4f)

^{1}H NMR (400 MHz, DMSO-d$_6$)

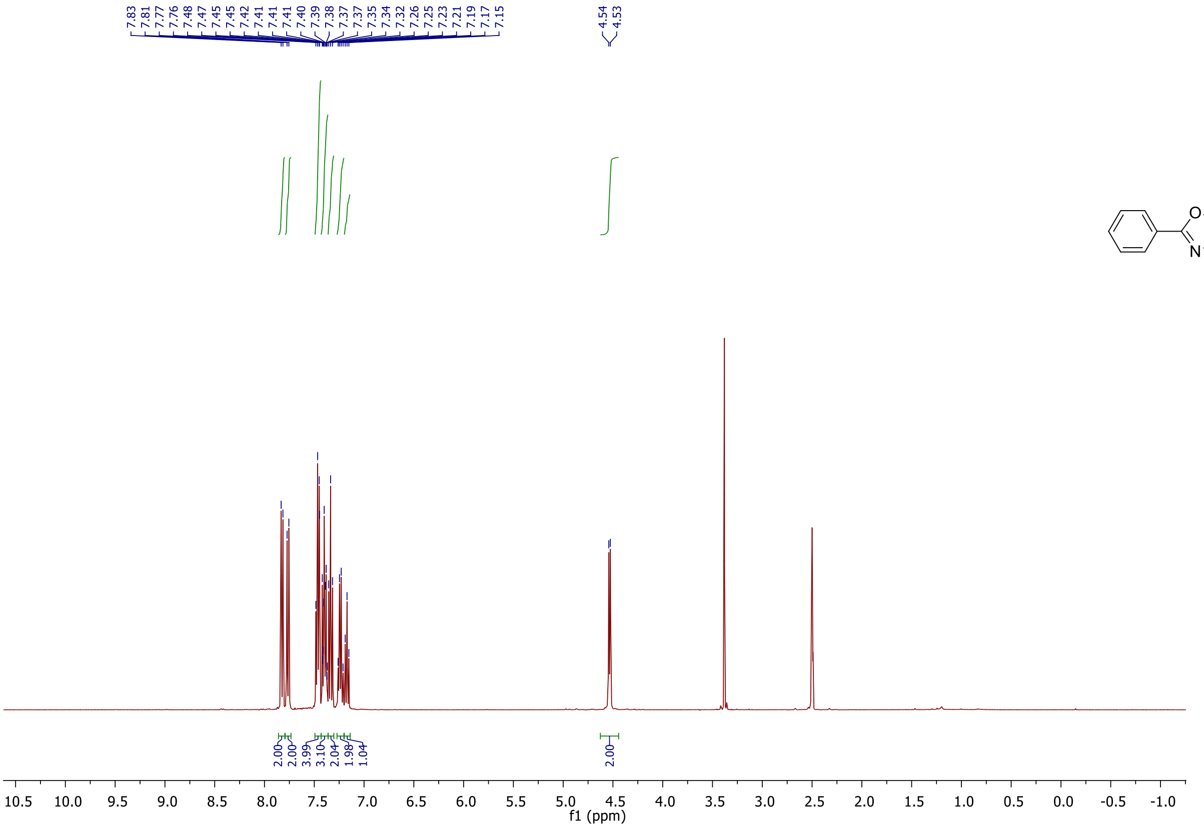

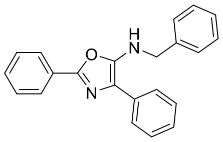

$^{13}C\{^{1}H\}$ NMR (101 MHz, DMSO-d$_6$)

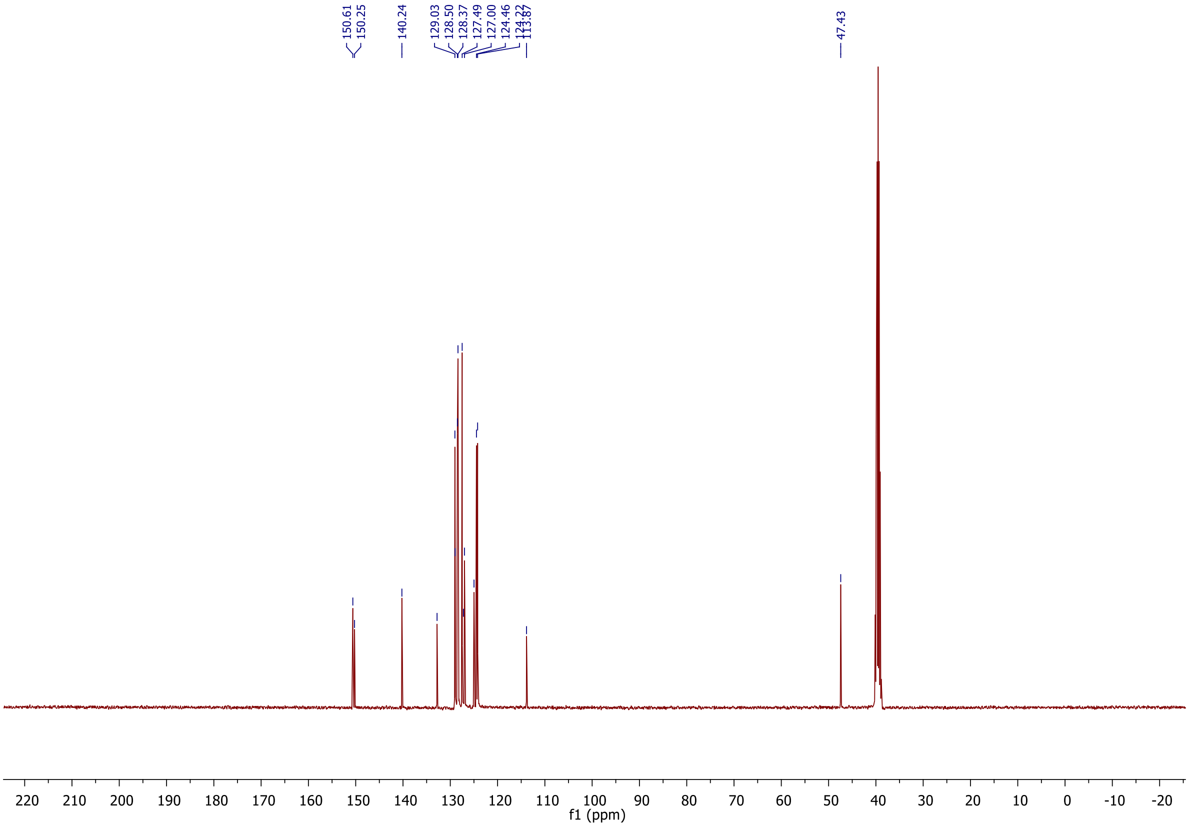

Figure S.52. *N*-cyclohexyl-2,4-diphenyl-1,3-oxazol-5-amine (4g)

^{1}H NMR (400 MHz, DMSO-d$_6$)

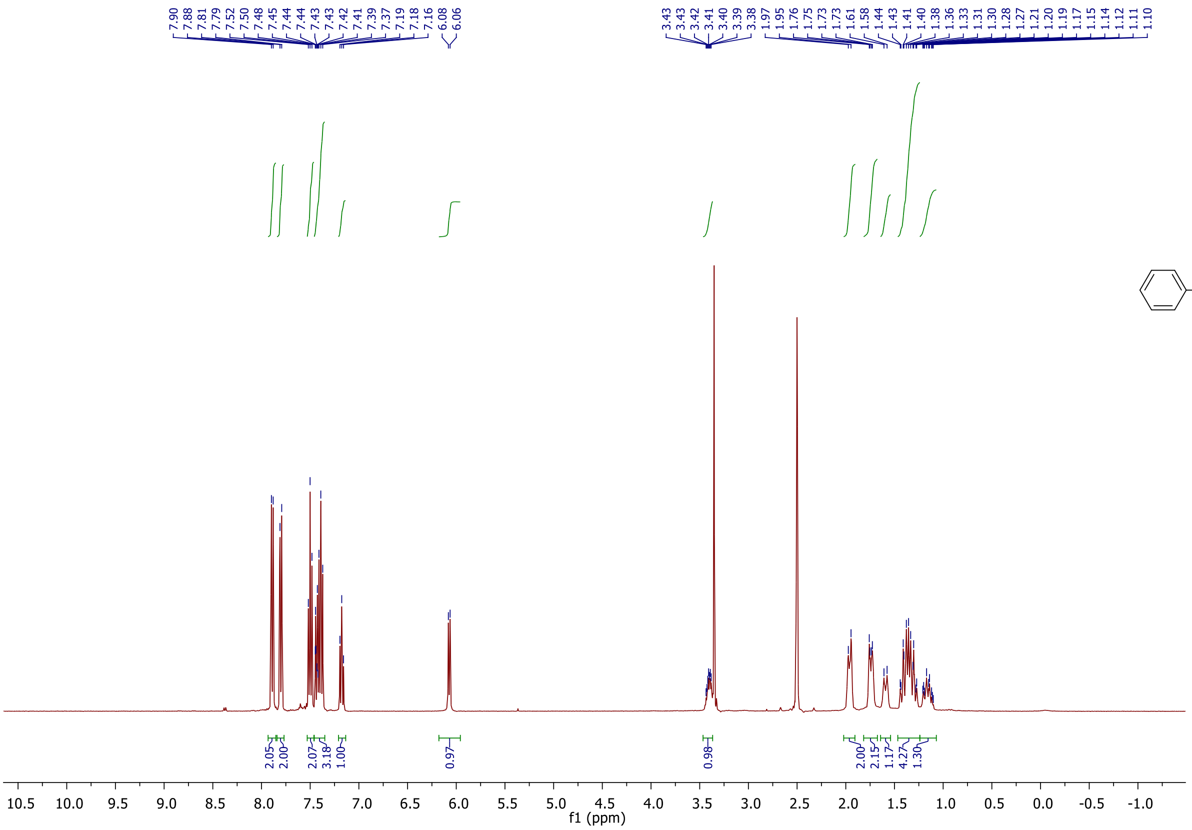

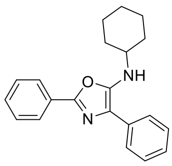

^{13}C{^{1}H} NMR (101 MHz, DMSO-d$_6$)

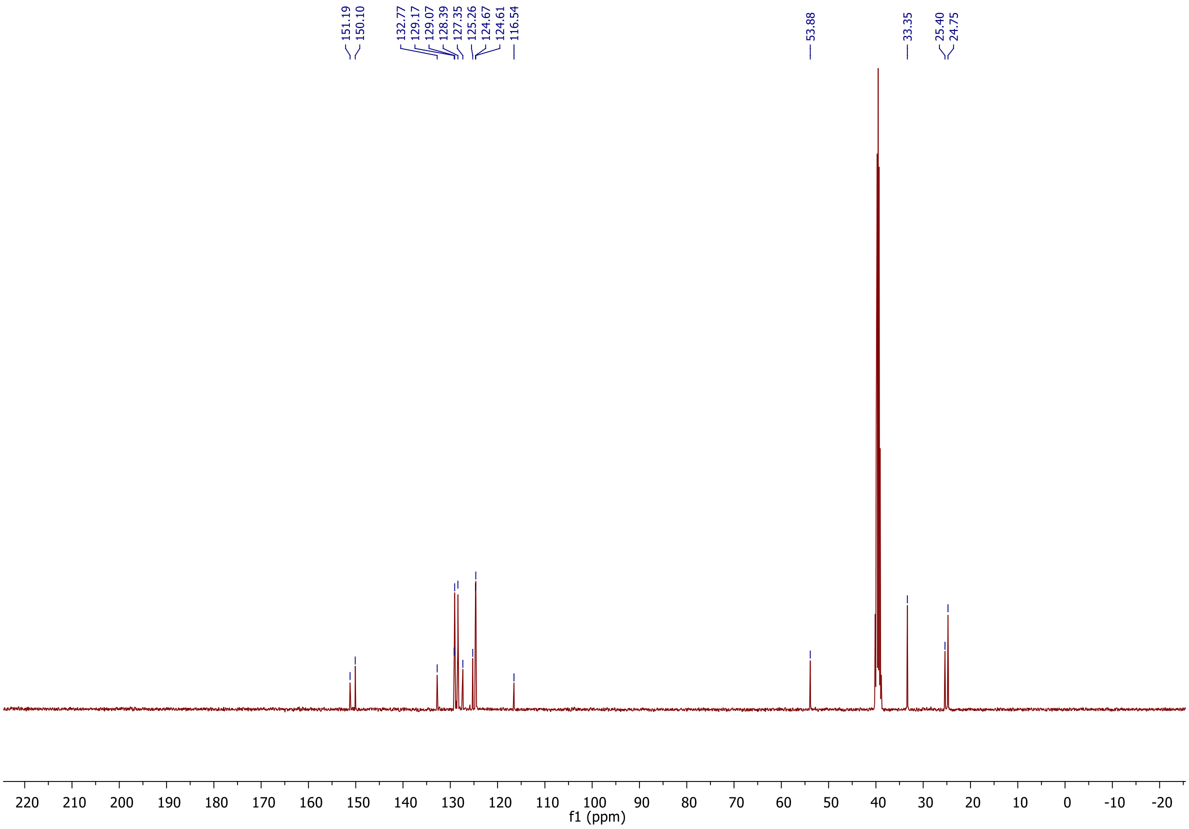

Figure S.53. *N*-tert-butyl-2,4-diphenyl-1,3-thiazol-5-amine (6a)

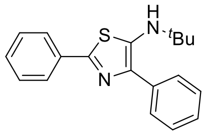

^{1}H NMR (400 MHz, DMSO-d$_6$)

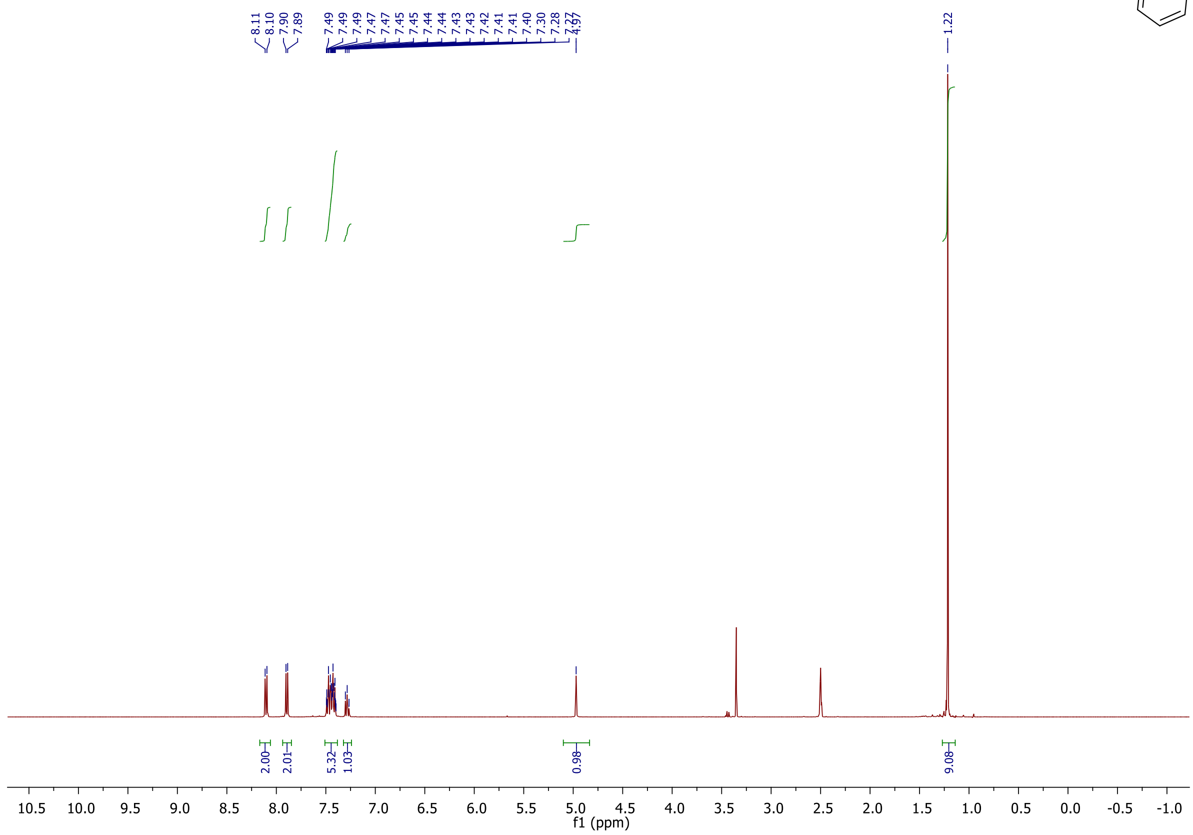

^{13}C{^{1}H} NMR (101 MHz, DMSO-d$_6$)

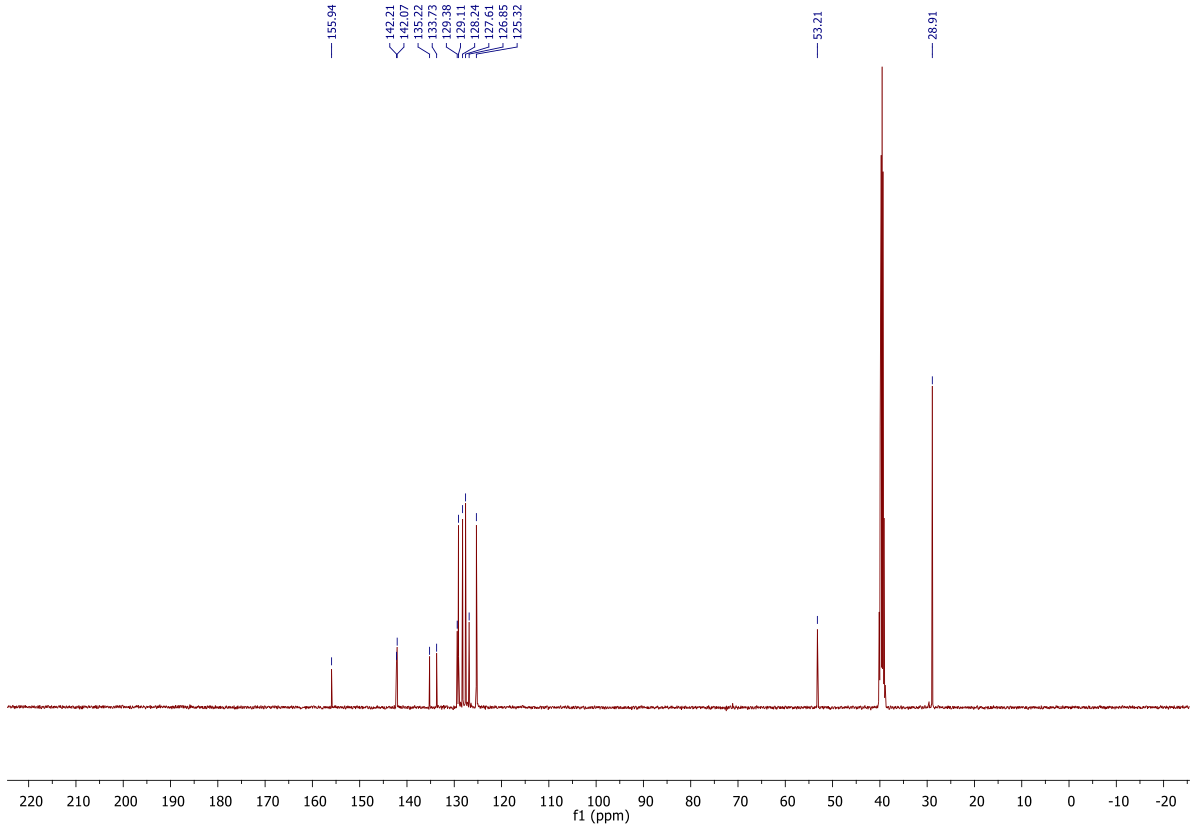

Figure S.54. 4-(4-bromophenyl)-*N*-*tert*-butyl-2-phenyl-1,3-thiazol-5-amine (6b)

^{1}H NMR (400 MHz, DMSO-d$_6$)

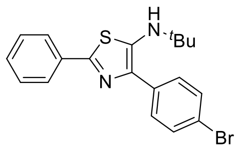

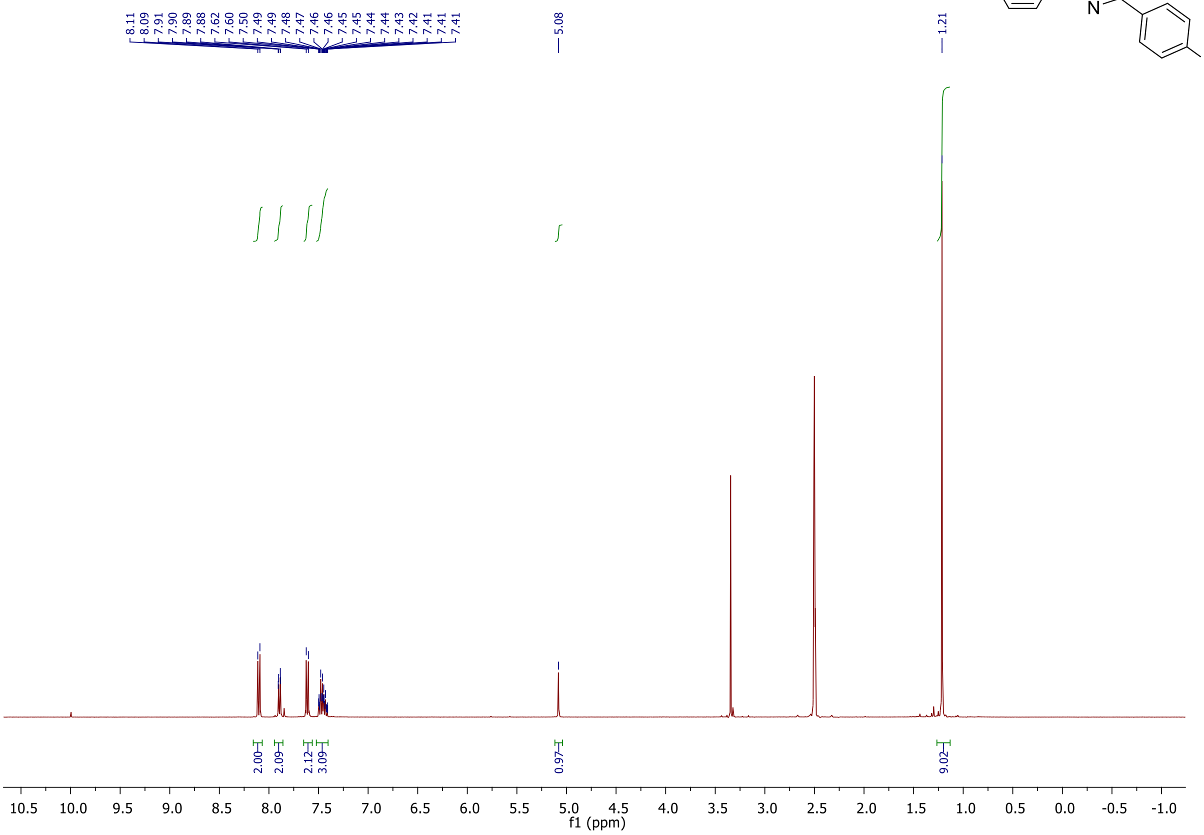

^{13}C{^{1}H} NMR (101 MHz, DMSO-d$_6$)

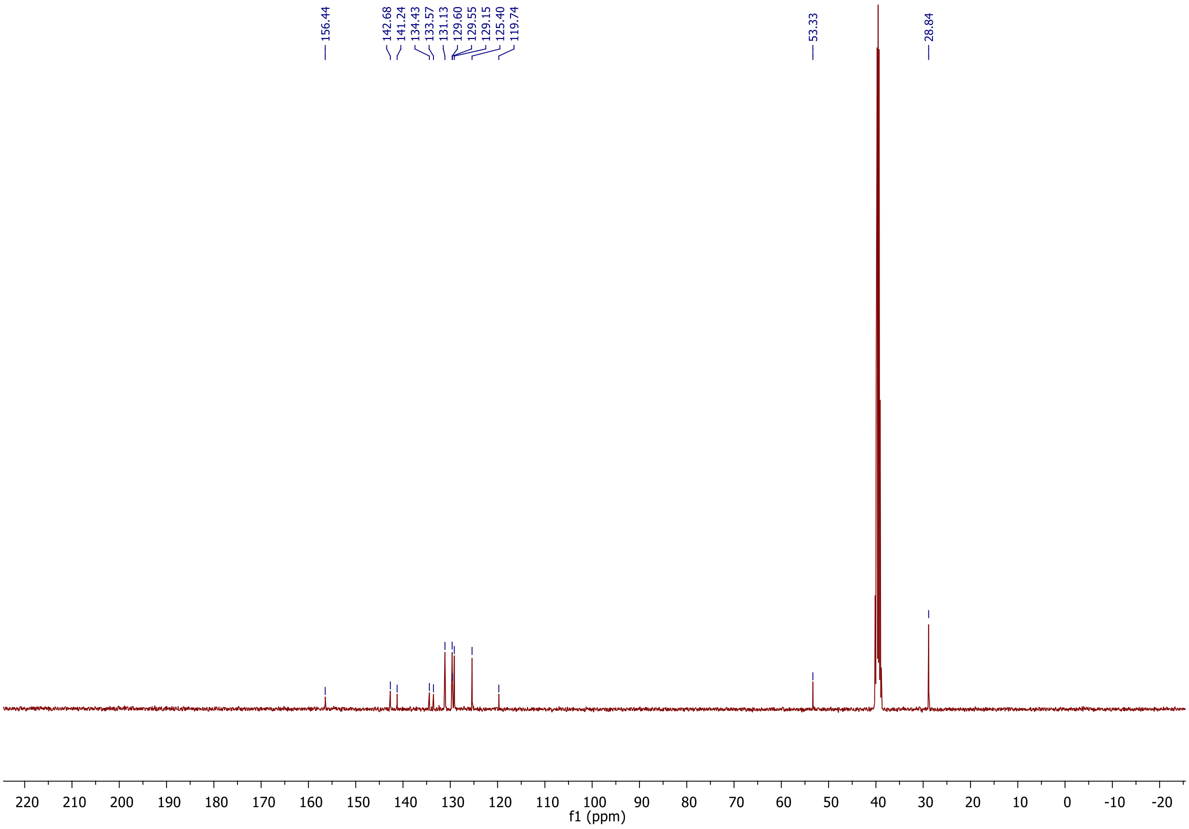

Figure S.55. 2-(4-bromophenyl)-*N-tert*-butyl-4-phenyl-1,3-thiazol-5-amine (6c)

^{1}H NMR (400 MHz, DMSO-d$_6$)

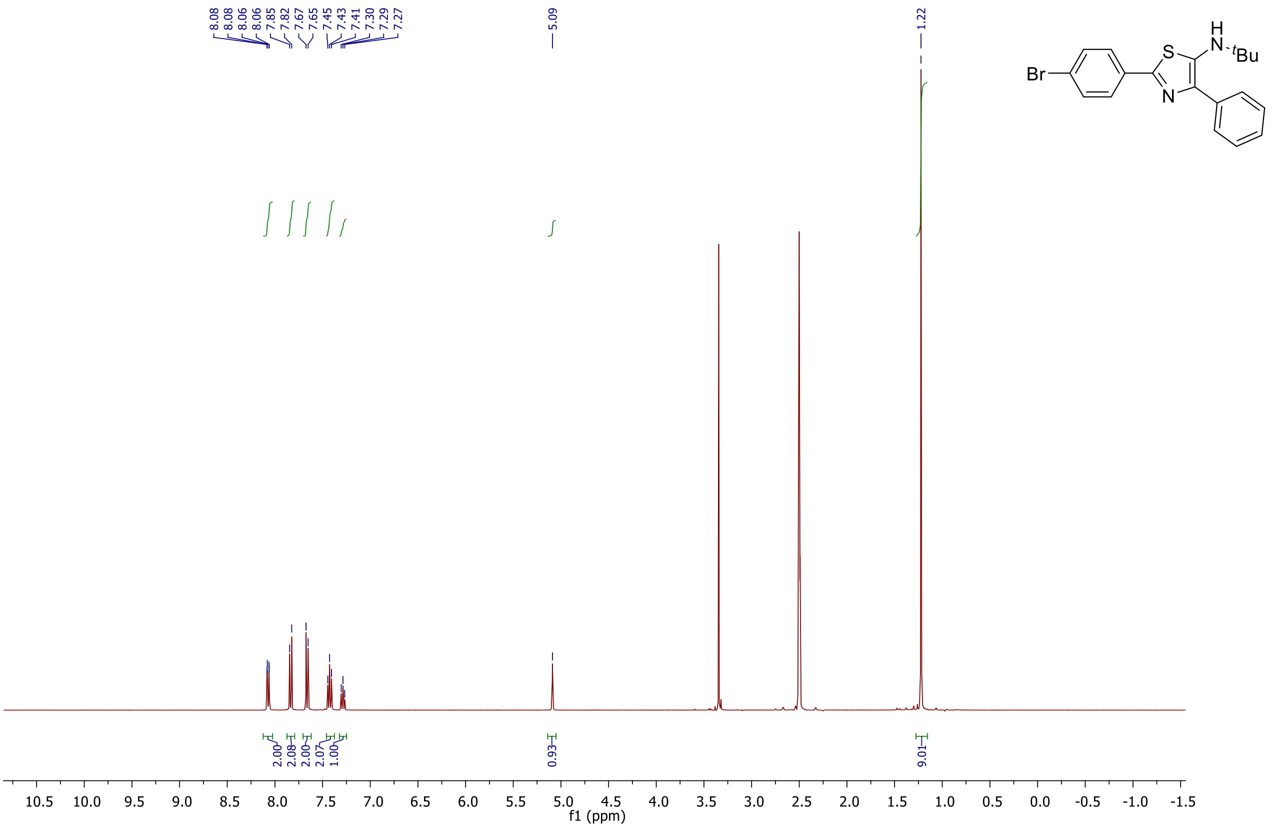

^{13}C{^{1}H} NMR (101 MHz, DMSO-d$_6$)

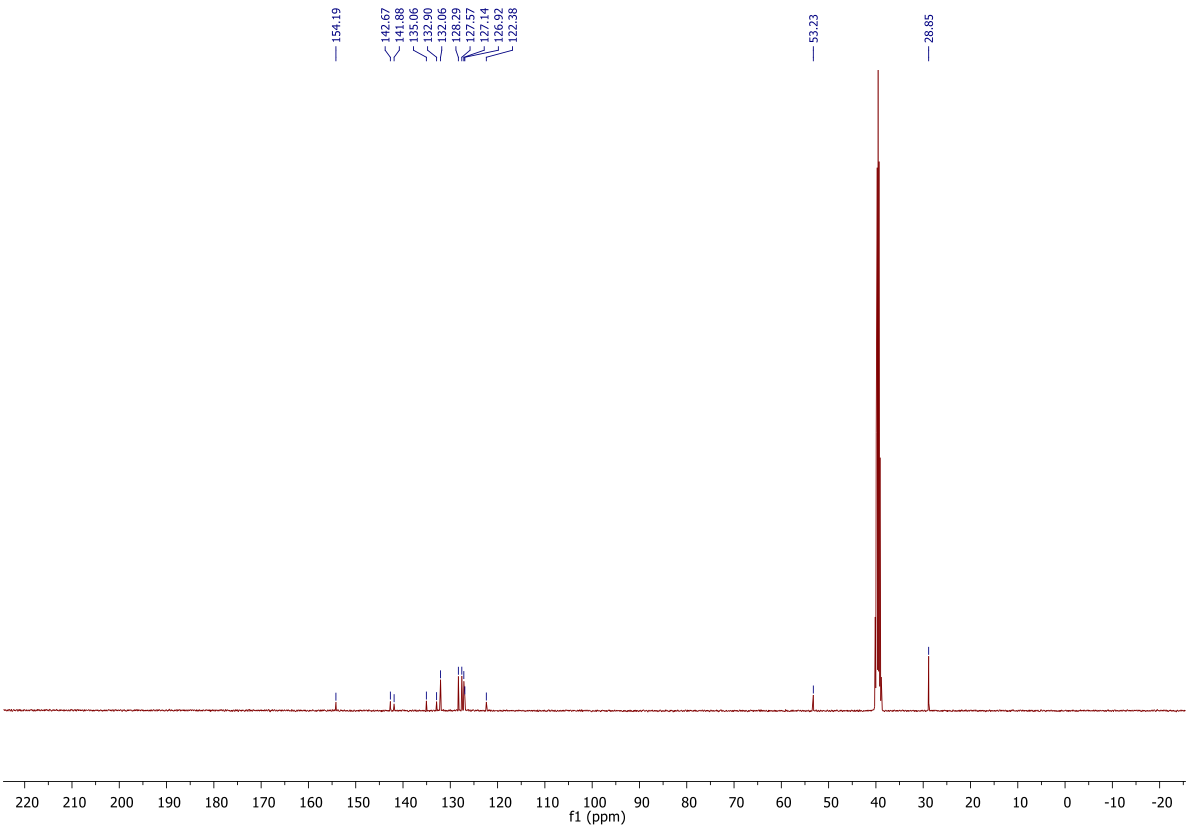

Figure S.56. *N*-cyclohexyl-2,4-diphenyl-1,3-thiazol-5-amine (6d)

^{1}H NMR (400 MHz, DMSO-d$_6$)

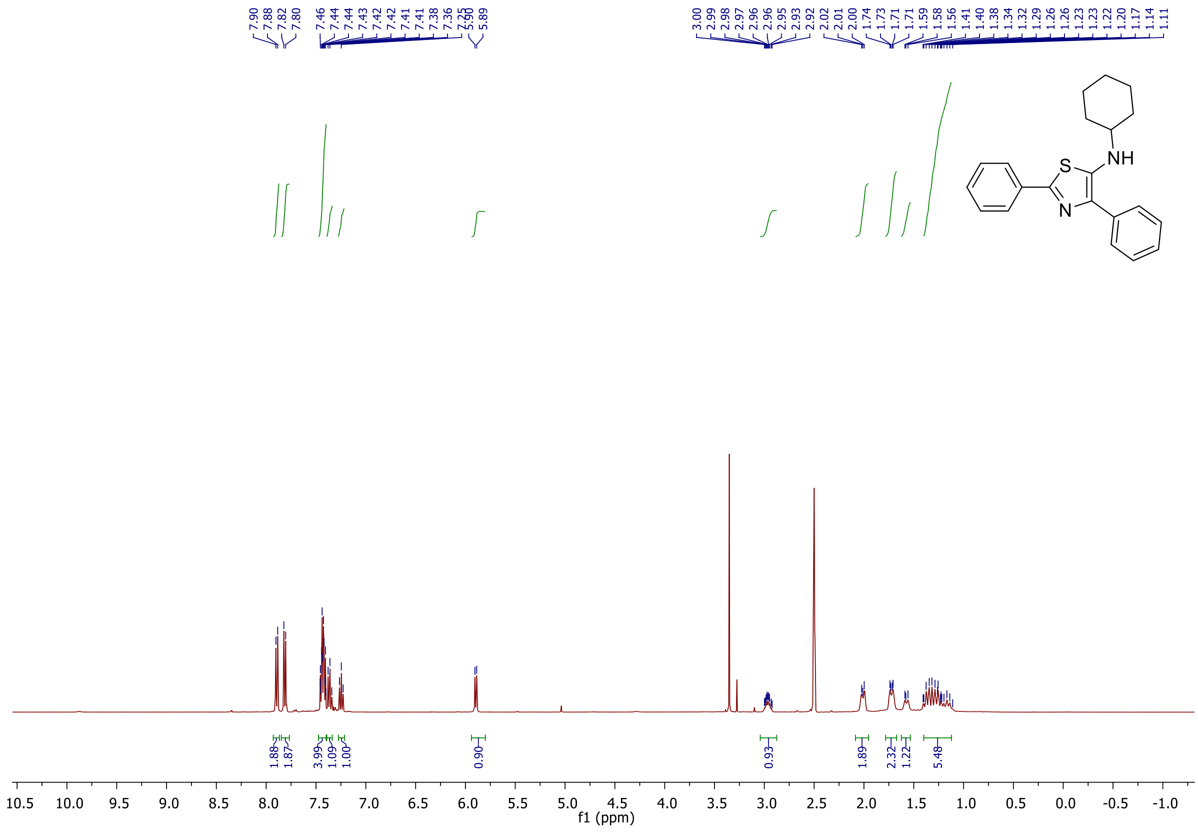

$^{13}C\{^{1}H\}$ NMR (101 MHz, DMSO-d$_6$)

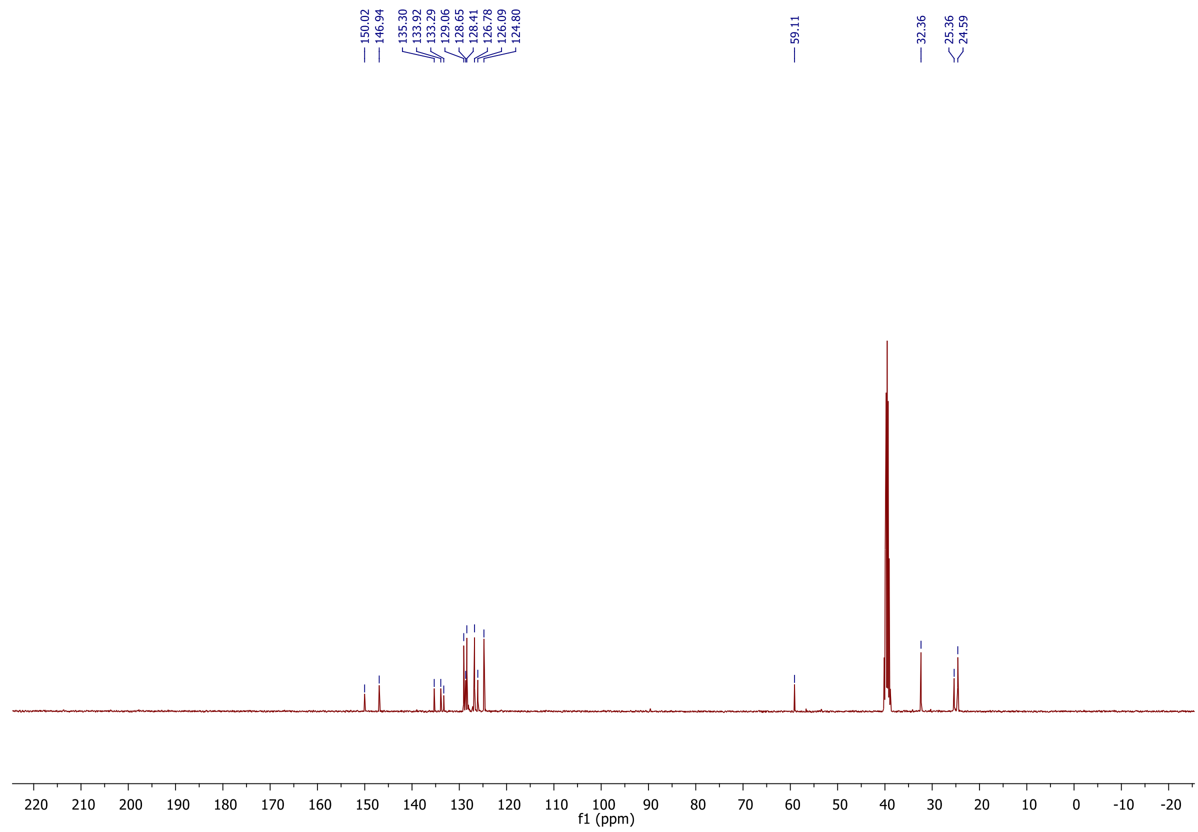

Figure S.57. 4-(4-bromophenyl)-*N*-cyclohexyl-2-phenyl-1,3-thiazol-5-amine (6e)

^{1}H NMR (400 MHz, DMSO-d$_6$)

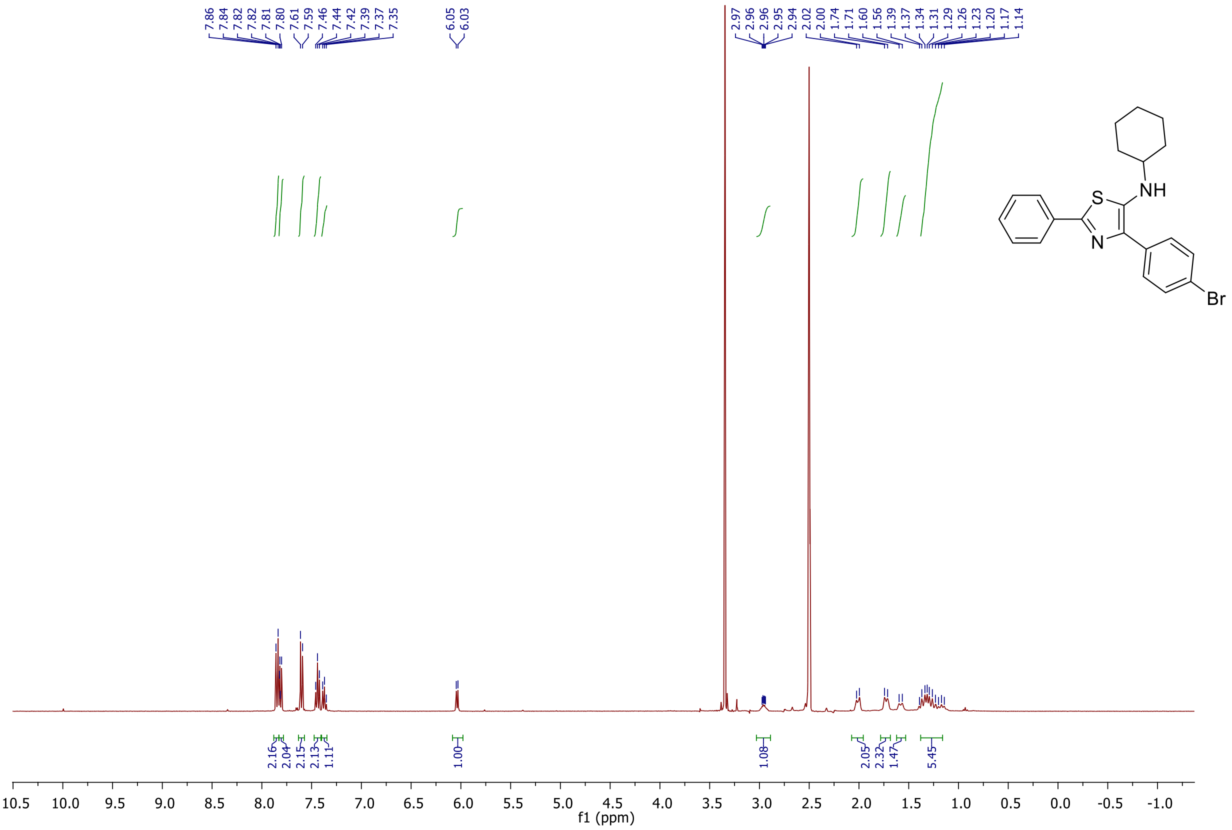

^{13}C{^{1}H} NMR (101 MHz, DMSO-d$_6$)

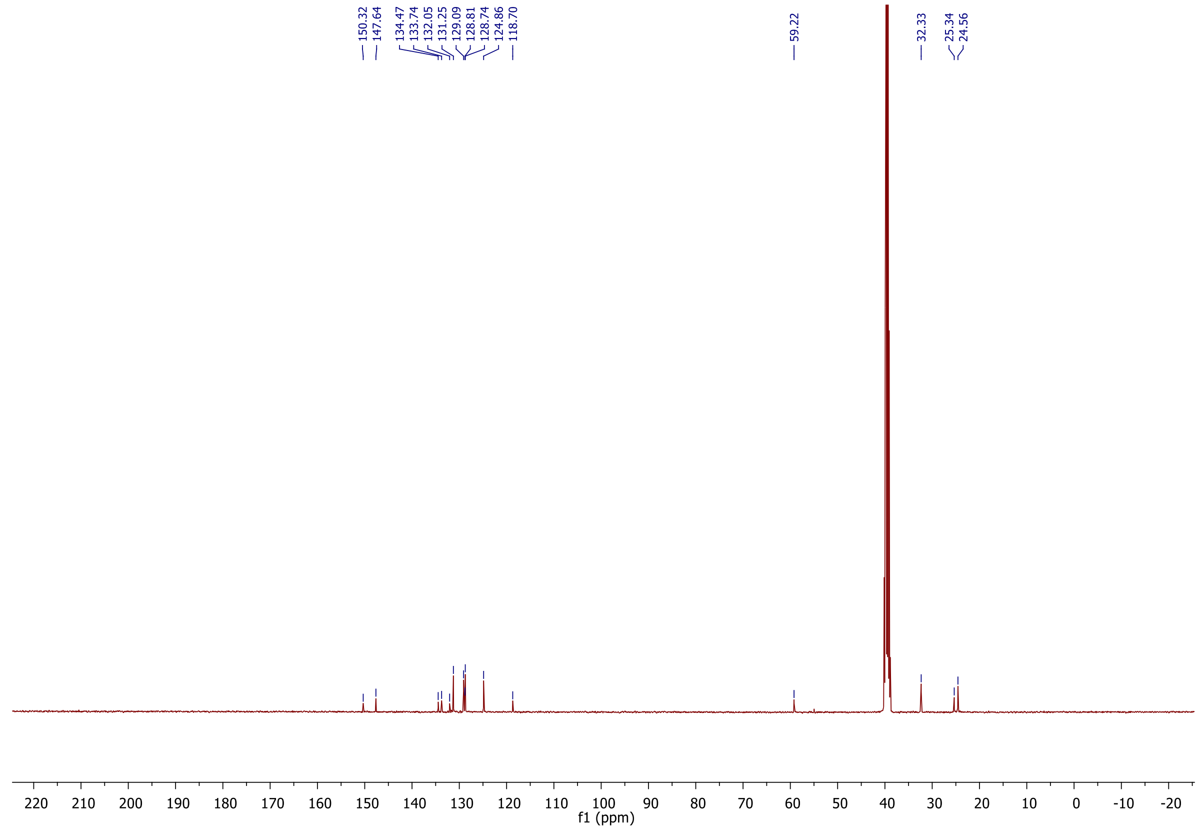

Figure S.58. 2-(4-bromophenyl)-*N*-cyclohexyl-4-phenyl-1,3-thiazol-5-amine (6f)

^{1}H NMR (400 MHz, DMSO-d$_6$)

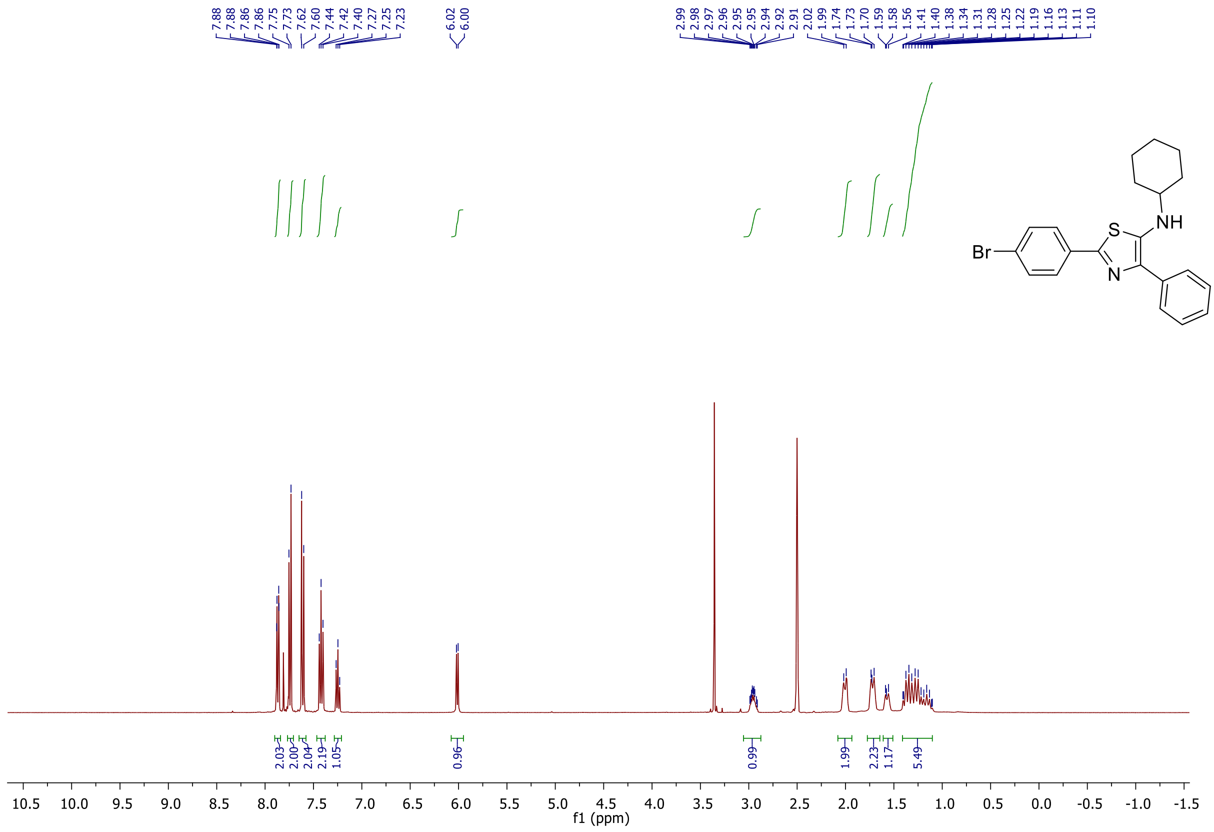

^{13}C{^{1}H} NMR (101 MHz, DMSO-d$_6$)

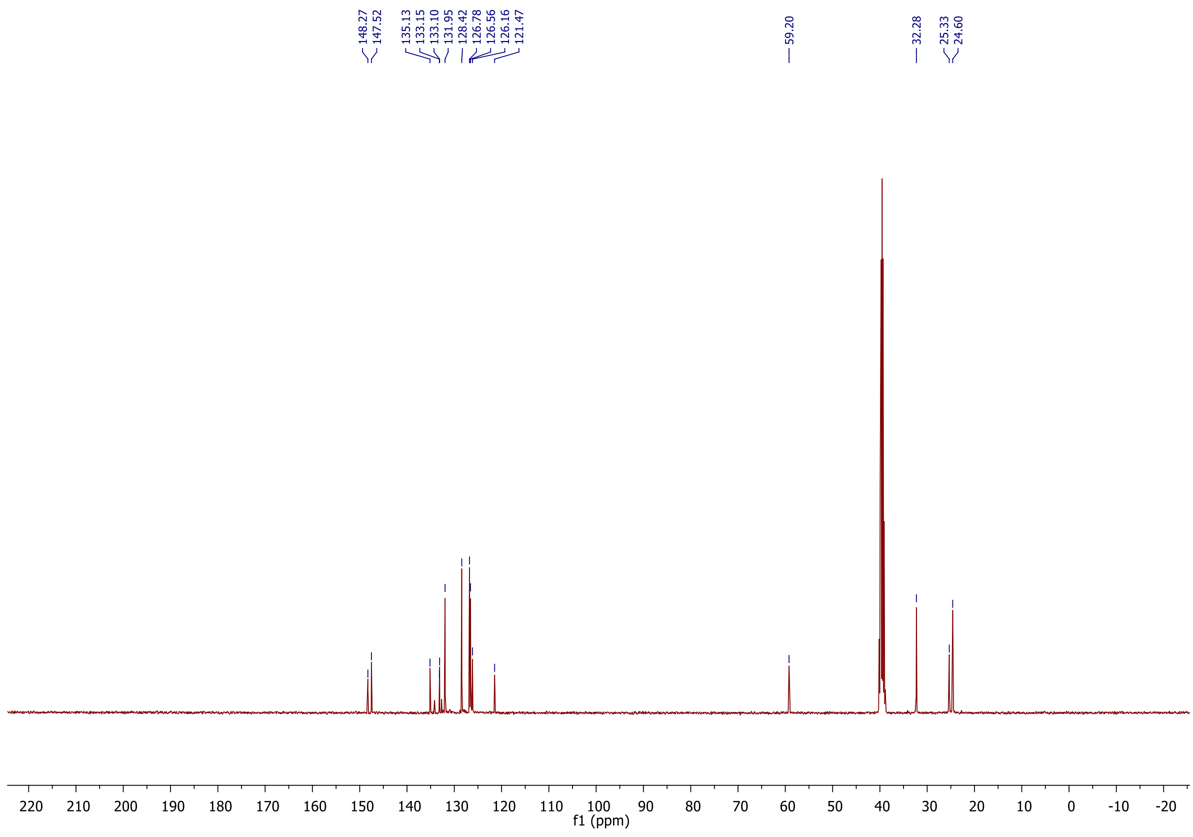

Supplement: Supplementary file 1 — Supplementary [file CSSC-14-1696-s001.pdf]
